# Abstracts from the 8th Drug Hypersensitivity Meeting (DHM)

**DOI:** 10.1186/s13601-018-0217-8

**Published:** 2018-08-21

## Thursday 19 April 2018

### Lessons from drug induced anaphylaxis

#### O01 Evidence of an IgG-induced neutrophil activation pathway during human drug-induced anaphylaxis

##### Luc De Chaisemartin^1^, Friederike Jönsson^2^, Vanessa Granger^1^, Aurélie Gouel-Chéron^2^, Caitlin Gillis^2^, Fadia Dib^3^, Pascale Nicaise-Roland^1^, Christelle Ganneau^4^, Marie-Thérèse Guinnepain^5^, Michel Aubier^6^, Sylvie Bay^4^, Catherine Neukirch^6^, Florence Tubach^7^, Dan Longrois^8^, Sylvie Chollet-Martin^1^, Pierre Bruhns^2^

###### ^1^APHP, Hôpital Bichat, UF Auto-immunité et Hypersensibilités, HUPNVS, Paris, France; ^2^Institut Pasteur, Department of Immunology, Unit of Antibodies in Therapy and Pathology, Paris, France; ^3^APHP, Hôpital Bichat, Department of Epidemiology and Clinical Research, INSERM, Paris, France; ^4^Institut Pasteur, Département Biologie Structurale et Chimie, Unité de Chimie des Biomolécules, Paris, France; ^5^Hôpital Foch, Service de médecine interne, Suresnes, France; ^6^APHP, Hôpital Bichat, Service de Pneumologie A, HUPNVS, Paris, France; ^7^INSERM, ECEVE, U1123, CIC 1421, Paris, France; ^8^APHP, Hôpital Bichat, Département d’Anesthésie-Réanimation, HUPNVS, Paris, France

**Correspondence:** Luc De Chaisemartin - luc.de-chaisemartin@u-psud.fr

*Clinical and Translational Allergy* 2018, **8(Suppl 3)**:O01

**Background**

Anaphylaxis is an acute systemic hypersensitivity reaction considered to rely on IgE antibodies against an allergen and histamine release by mast cells and basophils. However, data from animal models suggest an alternative pathway dependent on IgG antibodies and involving platelet-activating factor (PAF) release by monocyte/macrophages and neutrophils. Evidence of this mechanism in human is scarce and limited to rare allergens of high molecular weight such as dextran or protamine. To determine if such a pathway exists in drug anaphylaxis, we conducted a multicentric study on patients with suspected anaphylaxis to neuromuscular blocking agents (NMBA) during general anesthesia.

**Methods**

We prospectively included 86 patients with a suspicion of NMBA-induced anaphylaxis and 86 matched controls from 10 French anesthesia departments (NASA study). Anti-NMBA IgE and IgG levels were measured by FEIA on an Immunocap 250 instrument. Expression of IgE and IgG receptors on blood cells as well as activation markers on neutrophils were determined by flow cytometry. Circulating neutrophils extracellular traps and elastase levels were measured by ELISA. PAF-acetylhydrolase (PAF-AH) activity, a plasmatic marker inversely correlated with PAF concentrations, was measured by an enzymatic method.

**Results**

Anti-NMBA IgE but also anti-NMBA IgG could be detected in patients and correlated with anaphylaxis severity. Neutrophil activation markers as well as markers of degranulation and netosis could be measured in patients early after anaphylaxis onset. PAF-AH activity was significantly lower in patients. Importantly, neutrophil activation and PAF release were associated with anaphylaxis severity and could also be observed in patients lacking evidence of classical IgE-dependent anaphylaxis. Finally, anti-NMBA IgG antibodies affinity-purified from patient serum triggered neutrophil activation ex vivo in the presence of NMBA.

**Conclusion**

This study supports the existence of a pathogenic IgG-neutrophil-PAF pathway in human NMBA-induced anaphylaxis that may contribute to anaphylaxis severity and be responsible for non-IgE mediated anaphylaxis in humans.

## Thursday 19 April 2018

### Beyond T cell in drug hypersensitivity

#### O02 Oxidative stress and sulfonylarylamines hypersensitivity reactions: new insights

##### Abdelbaset A. Elzagallaai, Michael J. Rieder

###### Western University, London, Canada

**Correspondence:** Abdelbaset A. Elzagallaai - aelzaga@uwo.ca

*Clinical and Translational Allergy* 2018, **8(Suppl 3)**:O02

**Background**

Sulfonylarylamines (SAAs), such as the antibacterial sulfamethoxazole, are a group of very important and useful medications. However, they are associated with a major adverse reaction, namely hypersensitivity reaction (HR), with a rate that ranges between 2% to 4% in the general population but can occur in nearly 50% in HIV-positive patients. The pathophysiology of sulfonylarylamine-induced HRs is not well-understood but accumulation of toxic reactive metabolites is thought to be a major factor. These RMs contribute, in part, to the formation of reactive oxygen species (ROS), which can cause cellular damage and induce cell death through apoptosis and necroptosis. ROS can also serve as ‘danger signals’ primining immune cells to mount the reaction.

**Methods**

We collected blood samples from suspected SAAs HS patients (n = 26), health volunteers (HV, n = 13) and sulfonamide-tolerant patients (ST, n = 6). We then isolated peripheral blood monocytes (PBMCs) and blood platelets and measured the induction of cell death in these cells upon in vitro challenge with different concentrations of the sulfamethoxazole (SMX) reactive metabolite, sulfamethoxazole hydroxylamine (SMX-HA). We then compared the degrees of cell death with accumulation of ROS, lipid peroxidation, level of formation of carbonyl protein, another marker of cellular oxidative stress, and cellular glutathione contents.

**Results**

When challenged with the RM in vitro, cells isolated from SSA HS patients exhibited significantly (p < 0.05) higher degrees of cell death than HV and ST groups. Also ROS accumulation, lipid peroxidation and carbonyl protein levels were found to be higher in cells from patients than the HV and ST groups after challenged with RM of the drug. In addition, there was a high degree of correlation between cell death and ROS levels.

**Conclusion**

Data from this study clearly indicate a major role of oxidative stress in the pathophysiology of SAAs hypersensitivity reactions.

## Thursday 19 April 2018

### Skin reactions in drug allergy - Poster Walk 1

#### P01 Cytokine profile in 133 patients with severe cutaneous adverse reactions

##### Sophie Lalevée, Etienne Audureau, Audrey Riou, Audrey Colin, Maxime Anquetin, Caroline Barau, Laurence Valeyrie-Allanore, Marie-Hélène Delfau-Larue, Olivier Chosidow, Pierre Wolkenstein, Saskia Oro, Sophie Hüe

###### Henri Mondor Hospital, Créteil, France

**Correspondence:** Saskia Oro - saskia.oro@aphp.fr

*Clinical and Translational Allergy* 2018, **8(Suppl 3)**:P01

**Background**

The clinical heterogenicity in Severe Cutaneous Adverse Reactions (SCARs) (Epidermal Necrolysis, DRESS and AGEP) may be explained by different immune pathways. We studied and compared four cytokines in the three main SCARs: TNFα and IL-6, two main cytokines of inflammation known to be increased in SCAR; IL1R α, antagonist receptor of IL1β involved in Th17 differentiation; and sST2, antagonist receptor of IL-33, implicated in Th2 response.

**Methods**

This monocenter study involved 133 patients (women = 76 [57%], mean age 48 ± 19 years) with SCARs, 22 [16.5%] had DRESS syndrome, 27 AGEP [20.4%], 84 [63.1%] EN including 27 Stevens–Johnson’s syndromes (SJS), 29 overlap syndromes and 28 Lyell syndromes (toxic EN, TEN). The dosage of TNFα, IL-6, IL1Rα and sST2 was performed in early patients’ sera (< day 5 after admission) using Luminex technology (R&D, Minneapolis, USA). A clustering analysis was made to identify homogenous profiles of patients with similar cytokines values. We then looked for an association between those cytokine profiles at diagnosis and the following risk of sepsis, stay in intensive care unit and mortality.

**Results**

Median levels of each cytokine are reported in Fig. 1. IL-6 levels were higher in EN than in DRESS or AGEP. TNF α levels were higher in DRESS (p < 0.05). sST2 levels were higher in AGEP or DRESS than in EN (p < 0.001).

**Fig. 1:**
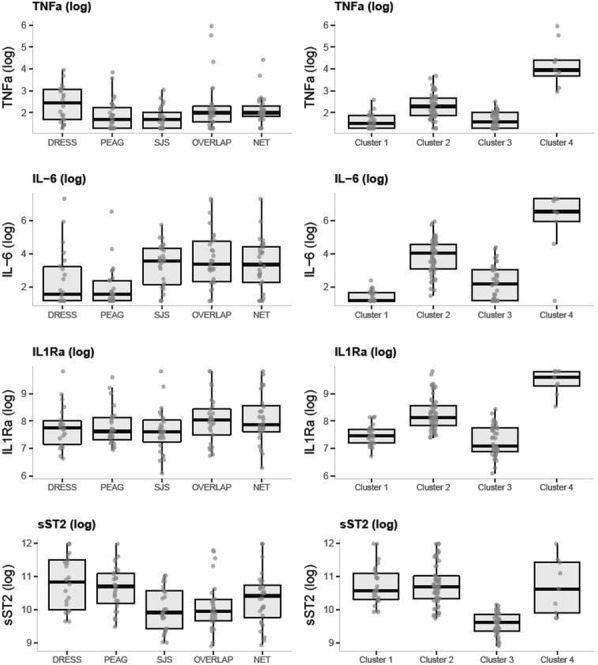
Results

Four cytokine profiles were identified by clustering. Two clusters were of particular interest. Cluster no. 1 (n = 26), characterized by a high level of sST2 with lower levels for other cytokines, mostly involved AGEP (65%) and was associated with a low rate of complications (sepsis [8%], death [0%]). Cluster no. 4 (n = 9), characterized by high levels of all cytokines, mostly involved patients with overlap or TEN (67%) and was associated with a higher rate of sepsis (56%) or death (22%). Clustering analysis did not discriminate DRESS and EN.

**Conclusion**

Our study determined four different cytokine profiles in SCARs. Interestingly, AGEP were characterized by high levels of sST2, which reflects IL-33, alarmin implied in Th2 response and tissue reparation. Cluster analysis confirmed this association between sST2 and AGEP. A high level of all 4 cytokines, mainly associated with EN with > 10% detached surface, was associated of a higher risk of complications. The dosage of IL15 will be performed to better discriminate DRESS and EN.

The prognosis role of cytokine levels at diagnosis should be evaluated with other prognostic factors such as SCORTEN.

#### P02 Significant association between HLA-B*44:03 and cold medicine related Stevens–Johnson Syndrome (SJS) and Toxic Epidermal Necrolysis (TEN) with severe ocular complications in various ethnic populations

##### Mayumi Ueta^1^, Passara Jongkhajornpong^2^, Tais Hitomi Wakamatsu^3^, Chitra Kannabiran^4^, Kaevalin Lekhanont^2^, José Álvaro Pereira Gomes^3^, Virender Sangwan^5^, Chie Sotozono^1^, Shigeru Kinoshita^1^

###### ^1^Kyoto Prefectural University of Medicine, Kyoto, Japan; ^2^Ramathibodi Hospital, Mahidol University, Bangkok, Thailand; ^3^Federal University of São Paulo, São Paulo, Brazil; ^4^Tej Kohli Cornea Institute, Hyderabad, India; ^5^L.V. Prasad Eye Institute, Hyderabad, India

**Correspondence:** Mayumi Ueta - mueta@koto.kpu-m.ac.jp

*Clinical and Translational Allergy* 2018, **8(Suppl 3)**:P02

**Background**

Stevens–Johnson syndrome (SJS) and toxic epidermal necrolysis (TEN) are acute inflammatory vesiculobullous reactions of the skin and mucosa, such as the ocular surface, oral cavity, and genitals. Severe ocular complications (SOC) appear in not all, but about half of SJS/TEN patients who diagnosed by dermatologists.

We previously reported that HLA-B*44:03 was significantly associated with CM-SJS/TEN with SOC in Japanese, Indian and Brazilian, and the association was strongly associated in Indian and Brazilian Caucasian which are in Caucasian ethnic.

**Methods**

In this study, we added Thailand population and examined the association between HLA-B*44:03 and CM-SJS/TEN with SOC using Japanese (case n = 162, control n = 265), Indian (case n = 23, control n = 50), Brazilian Caucasian (case n = 16, control n = 61), Thailand (case n = 40, control n = 60) samples.

**Results**

HLA-B*44:03 showed significant positive associations with CM-SJS/TEN with SOC in Japanese (carrier frequency; OR = 4.9, P = 0.0041, gene frequency; OR = 4.8, P = 0.0020, Pc = 0.026), Indian (carrier frequency; OR = 11.4, P = 3.3 × 10^−5^, gene frequency; OR = 12.2, P = 4.1 × 10^−8^), Brazilian Caucasian (carrier frequency; OR = 5.5, P = 0.014, gene frequency; OR = 5.4, P = 0.0061), Thailand (carrier frequency; OR = 4.9, P = 0.0041, gene frequency; OR = 4.8, P = 0.0020, Pc = 0.026) populations.

**Conclusion**

The association of HLA-B*44:03 with CM-SJS/TEN with SOC were significant not only in Caucasian ethnic such as Indian and Brazilian Caucasian, but also in Japanese and Thailand. Thus, HLA-B*44:03 might be universal marker of high risk for the onset of CM-SJS/TEN with SOC.

#### P03 The medication risk of Stevens–Johnson Syndrome and toxic epidermal necrolysis in Asians: the major drug causality and comparison to the USA FDA label

##### Chun-Bing Chen^1^, Yu-Hsin Wang^1^, Wichittra Tassaneeyakul^2^, Yoshiro Saito^3^, Siew Eng Choon^4^, Haur Yueh Lee^5^, Mimi Mee Chang^6^, Francisca D. Roa^7^, Bo Cheng^8^, Wen-Hung Chung^1^

###### ^1^Department of Dermatology, Drug Hypersensitivity Clinical and Research Center, Chang Gung Memorial Hospital, Taipei, Linkou, and Keelung, Taiwan, Taipei, Taiwan; ^2^Department of Pharmacology, Faculty of Medicine, Khon Kaen University, Khon Kaen, Thailand; ^3^Division of Medicinal Safety Science, National Institute of Health Sciences, Kanagawa, Japan; ^4^Hospital Sultanah Aminah Johor Bahru, Clinical School of Medicine and Health Sciences, Monash University, Monash, Malaysia; ^5^Department of Dermatology, Singapore General Hospital, Singapore, Singapore; ^6^Division of Dermatology, Department of Medicine and Therapeutics, Prince of Wales Hospital, the Chinese University of Hong Kong, Hong Kong, Hong Kong SAR; ^7^University of the Philippines-Philippine General Hospital, Manila, Philippines, Manila, Philippines; ^8^Department of Dermatology, the First Affiliated Hospital of Fujian Medical University, Fuzhou, Fujian, China

**Correspondence:** Chun-Bing Chen - mueta@koto.kpu-m.ac.jp

*Clinical and Translational Allergy* 2018, **8(Suppl 3)**:P03

**Background**

Stevens–Johnson syndrome (SJS) and toxic epidermal necrolysis (TEN) are life-threatening severe cutaneous adverse reactions and are mainly related to medications. Specific ethnic genetic backgrounds associated with risk of SJS/TEN especially in Asians. However, there have been no large cohort, multiple-country epidemiological studies of medication risk related to SJS/TEN in Asian populations.

**Methods**

We analyzed data regarding drug-induced SJS/TEN patients from various Asian SCAR consortium registration databases, including those of multiple medical centers in Taiwan, Thailand, Japan, Malaysia, Singapore, the Philippines, Hong Kong, and mainland China (Fujian), for the period from January 1998 to April 2017. A total 1028 SJS/TEN cases were identified by ALDEN. Furthermore, those medications labeled by USFDA as carrying a risk of SJS/TEN were also compared to the common causes of SJS/TEN in Asian countries. Moreover, we compared the Asian cohort in this study with the EuroSCAR European cohort of 379 published SJS/TEN cases (Sassolas, B. et al. Clin Pharmacol Ther 2010; 88, 60–8) in terms of drug causality.

**Results**

Oxcarbazepine, sulfasalazine, COX-II inhibitors, and strontium ranelate were identified as new potential causes (Table 1).

**Table 1:** Distributions of drugs classification related to SJS/TEN and comparison with US FDA label

Anti-epileptic drugs/antipsychotics	US FDA	n	(%)
Carbamazepine	Labeled	268	26.07
Phenytoin	Labeled	137	13.33
Lamotrigine	Labeled	104	10.12
Phenobarbital	Labeled	21	2.04
Oxcarbazepine	Labeled	18	1.75
Valproic acid	Labeled	2	0.19
Bupropion	Labeled	1	0.10
Fluoxetine	Labeled	1	0.10
Levetiracetam	Labeled	1	0.10

Antibiotics/antiviral agents	US FDA	n	(%)
Sulfamethoxazole	Labeled	81	7.88
Aminopenicillins^a^	Labeled	30	2.92
Quinolones^b^	Labeled	26	2.53
Cephalosporins^c^	Labeled	24	2.33
Clarithromycine	Labeled	9	0.88
Sulfadoxine/pyrimethamine	Labeled	6	0.58
Doxycycline	Labeled	5	0.49
Dapsone	Labeled	4	0.39
Piperacillin	Labeled	4	0.39
Rifampicin	Not labeled	4	0.39
Vancomycin	Labeled	4	0.39
Clindamycin	Labeled	2	0.19
Fluconazole	Labeled	2	0.19
Imipenem	Labeled	2	0.19
Metronidazole	Labeled	2	0.19
Nevirapine	Labeled	2	0.19
Tetracycline	Not labeled	1	0.10
Azithromycin	Labeled	1	0.10
Floxacillin	Not approved	1	0.10
Streptomycin	Not labeled	1	0.10
Sulfadiazine	Labeled	1	0.10

NSAIDs	US FDA	n	(%)
Diclofenac potassium	Labeled	9	0.88
Piroxicam	Labeled	8	0.78
Etoricoxib	Not approved	7	0.68
Celecoxib	Labeled	4	0.39
Mefenamic acid	Labeled	4	0.39
Ibuprofen	Labeled	3	0.29
Meloxicam	Labeled	2	0.19
Etodolac	Labeled	1	0.10
Metamizole	Not approved	1	0.10
Naproxen	Labeled	1	0.10

Other drugs	US FDA	n	(%)
Allopurinol	Labeled	202	19.65
Sulfasalazine	Labeled	19	1.85
Esomeprazole	Labeled	8	0.78
Strontium ranelate	Not approved	8	0.78
Acetaminophen	Labeled	1	0.10
Acetylcysteine	Not labeled	1	0.10
Carbocysteine	Not approved	1	0.10
Dimercaptopropane	Not approved	1	0.10
Erlotinib hydrochloride	Labeled	1	0.10
Hydroxychloroquine	Labeled	1	0.10
Omeprazole	Labeled	1	0.10
Pantoprazole	Labeled	1	0.10

In addition to sulfa drugs and beta-lactam antibiotics, quinolones was also a common cause. Only one acetaminophen-induced SJS was identified, while several medications (e.g., oseltamivir, terbinafine, isotretinoin, and sorafenib) labeled as carrying a risk of SJS/TEN by USFDA were not found to have caused any of the cases in the Asian countries investigated in this study. Differences between Asian countries and European countries were found in the drug causality of SJS/TEN (Fig. 1). For example, carbamazepine accounted for 26.07% of the SJS/TEN cases in Asian countries investigated in this study while only accounting for 11.39% of such cases in European countries. In contrast, phenobarbital and nevirapine pose higher risks of causing SJS/TEN in European countries than they were found to pose in the Asian countries included in this study.

**Fig. 1:**
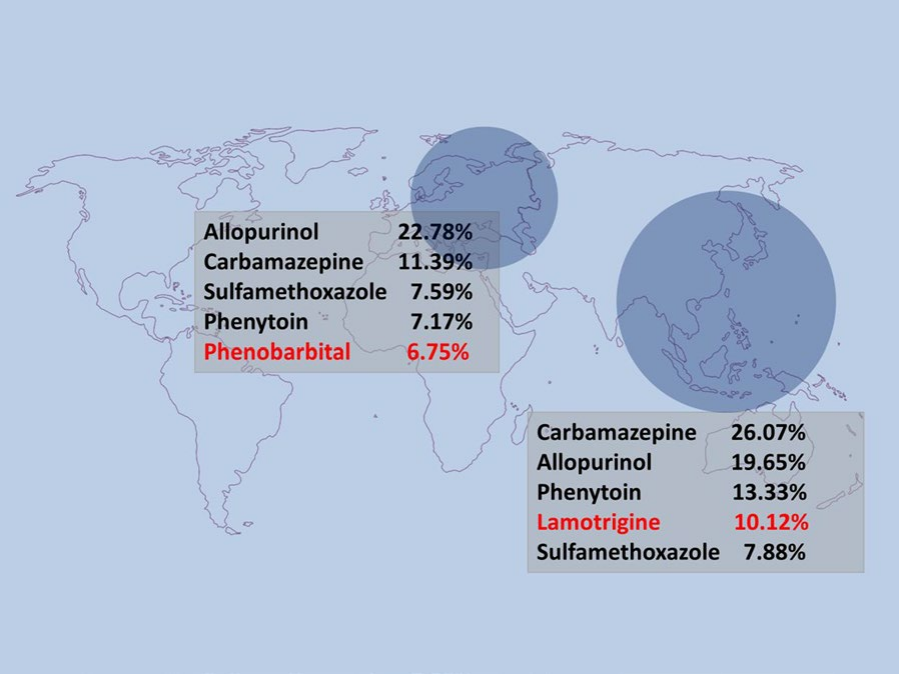
Differences between Asian countries and European countries

**Conclusion**

In summary, we provide qualitative profiles for the various medications that can potentially cause SJS/TEN in Asian populations, in addition to comparing those profiles with the corresponding profiles indicated by a European cohort study and US FDA labeling. The study results indicate a number of significant potential threats that are worthy of further investigation. The provided profiles could help to improve patient safety and increase awareness of clinicians of current medication risk related to SJS/TEN.

#### P04 Immunopathogenesis of severe T cell-mediated nevirapine hypersensitivity reactions in a South African patient cohort

##### Katherine Chanel Konvinse^1^, Jonathan Peter^2^, Katie Davis White^1^, Louise Barnett^1^, Rebecca Pavlos^3^, Juliet Esterhuizen^2^, Alec Redwood^3^, Simon Mallal^1^, Abha Chopra^3^, Rannakoe Lehloenya^2^, Elizabeth Phillips^1^

###### ^1^Vanderbilt University Medical Center, Nashville, TN, United States; ^2^University of Cape Town, Cape Town, South Africa; ^3^The Institute for Immunology & Infectious Diseases, Murdoch, Australia

**Correspondence:** Katherine Chanel Konvinse - katherine.c.konvinse@vanderbilt.edu

*Clinical and Translational Allergy* 2018, **8(Suppl 3)**:P04

**Background**

The current global use of nevirapine in first-line combination antiretroviral therapy is limited by immune-mediated adverse drug reactions (IM-ADRs) such as Stevens–Johnson Syndrome/toxic epidermal necrolysis (SJS/TEN) and drug reaction with eosinophilia and systemic symptoms (DRESS). HLA and other genetic associations such as CYP2B6*6 associations with nevirapine IM-ADRs have been variable differing across populations and clinical phenotypes. Although granulysin is the major cytotoxic peptide mediating keratinocyte death in SJS/TEN, the clonality and phenotype of T cells at the site of tissue damage for nevirapine SJS/TEN have not been defined.

**Methods**

A biobank of samples including cryopreserved PBMCs, plasma, buffy coat, saliva, blister fluid and skin was accrued from > 80 nevirapine-exposed HIV+ patients from three centers that included mother–child nevirapine-exposed pairs where only one relative developed SJS/TEN. HLA, KIR, ERAP and CYP2B6 typing was performed on all subjects. Immunohistochemistry was used to stain formalin-fixed, paraffin-embedded skin biopsies from nevirapine SJS/TEN patients with antibodies against SJS/TEN biomarker granulysin as well as general T cell (CD3, CD4, CD8), skin homing (CCR4, CLA), natural killer cell (CD56), regulatory T cell (FoxP3), and tissue resident (CD103) markers. These slides were analyzed blindly by a dermatopathologist and with Leica’s trainable and unbiased SlidePath Digital Image Hub software. Single cell TCR sequencing (sc-TCRseq) paired with whole transcriptome sc-RNAseq was performed on CD8+ T cells from the blister fluid from an acute nevirapine SJS/TEN patient.

**Results**

HLA-C*04:01 is strongly associated with nevirapine SJS/TEN. 100% of SJS/TEN cases (n = 15) carried HLA-C*04:01 compared to 22.5% of unrelated individuals tolerating nevirapine (n = 7/31) (p < 0.00001). Novel associations with HLA-B*44:03 and HLA-B*45:01, which share peptide binding specificities, were identified for nevirapine DRESS. CYP2B6 genotyping revealed that DRESS patients had slower nevirapine metabolizing phenotypes than tolerant controls. sc-TCRseq revealed a dominant TCRab CDR3 in the activated (CD137+) CD8+ T cells in the blister fluid. This clonotype was present in only a small fraction of CD137-CD8+ T and CD56+CD8+ NKT cells.

**Conclusion**

In a South African population, we identified a strong association between HLA-C*04:01 and SJS/TEN suggesting that this HLA class I allele may be necessary but not sufficient for the development of SJS/TEN. Results from our immunohistochemistry, sc-RNAseq and sc-TCR studies, that included the identification of a dominant TCR clonotype at the site of tissue damage, provide important insights into the specific immunopathogenesis of nevirapine SJS/TEN and the potential basis for why not all patients carrying an HLA risk allele might develop an IM-ADR.

#### P05 Fixed drug eruption: a monocenter clinical and histological study of 73 cases

##### Alice Viarnaud, Emilie Perron, Marina Thomas, Olivier Chosidow, Pierre Wolkenstein, Saskia Oro, Nicolas Ortonne

###### Henri Mondor Hospital, Créteil, France

**Correspondence:** Saskia Oro - saskia.oro@aphp.fr

*Clinical and Translational Allergy* 2018, **8(Suppl 3)**:P05

**Background**

Fixed drug eruption (FDE) is a rare cutaneous adverse reaction defined by more or less bullous, round purple red or brown patches, occurring typically ≤ 48 h after drug intake. Clinical features of bullous (BFDE) and non-bullous FDE (NBFDE) have never been compared. The aim of this study was to describe and compare clinical and histological features of NBFDE and BFDE.

**Methods**

This monocenter retrospective study included all patients with FDE with available skin biopsies from 2005 to 2016. FDE were classified as NBFDE or BFDE, localized (1 anatomic site) or generalized (≥ 2 sites). Clinical data were recorded from files, and follow-up was updated by phone. Skin biopsies were blindly reviewed, then correlated to the clinical presentation. Statistical analysis used Chi2, Fischer and Student tests.

**Results**

Seventy-three patients were included (89 biopsies, 44 female, median age 70). Fifteen had a NBFDE (median age 47) and 58 a BFDE (median age 77, p < 0.001, generalized n = 48), 28 (38%) reported a past history of FDE. Main culprit drugs were antibiotics (n = 20), paracetamol (n = 9) and NSAID (n = 6). Median time to onset was 2 days. Forty-eight patients (66%) were hospitalized, 25 of them had a complication (infectious n = 19), 5 required intensive care and 8 died (median age 80, all BFDE with > 10% BSA). Among 32 patients with follow-up, 4 experienced a recurrence (fatal n = 1).

Main histological findings were keratinocytic apoptosis (95%), melanophages (72%), lichenoid reaction (62.8%), junctional bullae (56.3%). T cell lymphocytes exhibited a cytotoxic CD8 profile. Inflammatory patterns were multiple in 91% of cases: eczematous (6.7%), lichenoid (48.3%) and epidermal necrolysis (EN, 66.3%). EN pattern (74% vs 27%, p = 0.008) and bullae (68% vs 7%, p = 0.006) were more frequent in BFDE. Melanophages (100% vs 66%, p = 0.02) and dermal melanosis (40% vs 4%, p = 0.0005) were more frequent in NBFDE (Fig. 1).

**Fig. 1:**
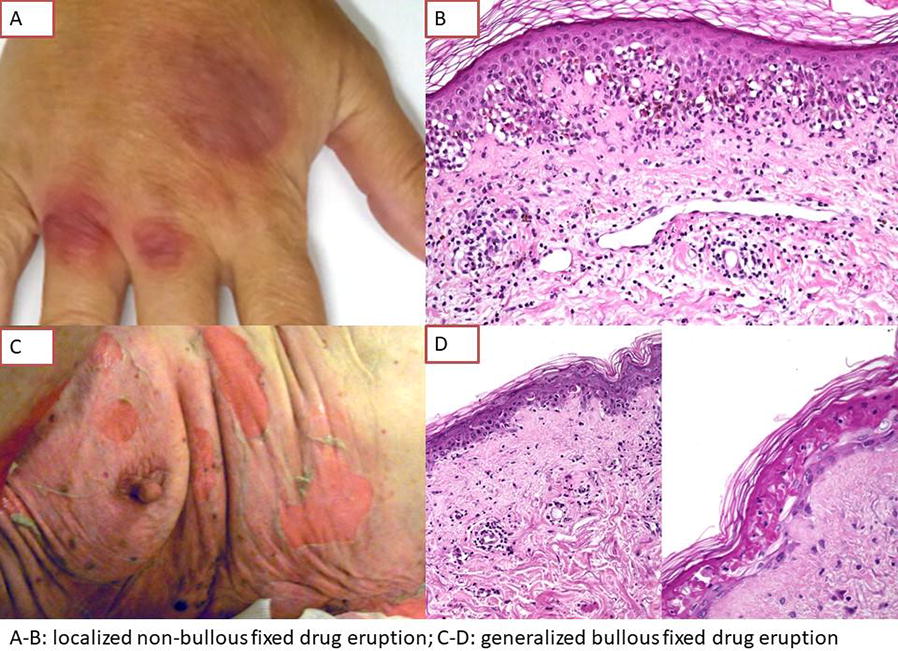
Results

**Conclusion**

Generalized BFDE occur in elderly and is associated with a high rate of morbidity. The advanced age of these patients suggests a severity spectrum between NBFDE and BFDE, but a past history of FDE often lacks. Senescence-induced dysregulation of immune response to drugs could also be hypothesized. Histologically, cytotoxic lesions mimic EN.

FDE is a spectrum from benign localized non-bullous to severe generalized bullous lesions. Histology is correlated to clinical presentation. Clinical-pathological is warranted to assess the diagnosis.

#### P06 Skin-infiltrating MDR1-expressing T cells are associated with therapeutic responsiveness to corticosteroids in severe cutaneous adverse reactions of drugs

##### Toshiharu Fujiyama^1^, Hideo Hashizume^2^, Kazuo Kurihara^1^, Yoshiki Tokura^1^

###### ^1^Department of Dermatology Hamamatsu University School of Medicine, Hamamatsu, Japan; ^2^Department of Dermatology, Shimada City Municipal Hospital, Shimada, Japan

**Correspondence:** Toshiharu Fujiyama - fujiyama@hama-med.ac.jp

*Clinical and Translational Allergy* 2018, **8(Suppl 3)**:P06

**Background**

Systemic corticosteroid administration is the mainstay of therapeutic strategy for severe cutaneous adverse reactions (SCARs) of drugs. However, some cases are resistant to the treatment and occasionally need additional or alternative treatments to abrogate the inflammatory responses. It is believed that the type of eruptions, the severity of the inflammation and the clearance of the causative drugs are the main factors to influence on the responsiveness of corticosteroids. In addition to these factors, we hypothesized that there are some variations in the corticosteroid sensitivity of skin-infiltrating T cells, containing major effector cells concerned with drug eruptions. Multidrug resistance protein 1 (MDR1, CD243; also known as P-glycoprotein 1), capable of pumping many foreign substances out of the cells, is associated with corticosteroid resistance of T cells in certain diseases.

**Methods**

In this study, we investigated skin-infiltrating T cells from nine cases of drug eruption, including four cases of Stevens–Johnson syndrome or toxic epidermal necrolysis (SJS/TEN), four cases of drug-induced hypersensitivity syndrome or drug reaction with eosinophilia and systemic symptoms (DIHS/DRESS), and one case of fixed drug eruption (FDE). Skin-infiltrating T cells were expanded from skin biopsy specimen using IL-2 and anti-CD3/CD28 mAb-coated microbeads, and MDR1 gene and protein expressions were assessed by RT-PCR and flow cytometry, respectively. The function of MDR-1 was assessed by Rhodamine 123 efflux assay.

**Results**

In our preliminary study, we confirmed that the gene expression of MDR-1 was correlated with its function. The function of MDR-1 in T cells was not affected by the cultivation and stimulation process, while it was suppressed by cyclosporine A, an inhibitor of MDR-1. As assessed by cell survival assay, MDR-1+ cells were resistant to the in vitro treatment of corticosteroid.

In all cases, both CD4+ and CD8+ T cells contained functional MDR-1+ T cells, and there was no significant difference in the frequency of MDR-1+ cells between SJS/TEN and DIHS/DRESS. However, the frequency of MDR-1+ tended to be higher in the patients who had previously received corticosteroid treatment. A case of FDE also showed a high frequency of MDR1+ T cells. MDR-1+ T cells produced IFN-gamma in a SJS case, and both IFN-gamma and IL-4 in a DIHS case.

**Conclusion**

These results suggest that MDR-1+ cells contain T cells pathogenic for drug eruptions, and a medication history of previously used corticosteroids leads to a high frequency of MDR-1+ cells, resulting in the decreased sensitivity to corticosteroid treatment.

#### P07 Specificity and sensitivity of lymphocyte transformation test in epidermal necrolysis: analysis of agreement with the Algorithm of Drug Causality for Epidermal Necrolysis (ALDEN)

##### Teresa Bellon^1^, Sara Rodríguez-Martín^2^, Victoria Lerma^3^, Elena Ramírez^4^, Carlos González-Herrada^5^, Rosario Cabañas^6^, Ana María Fiandor^6^, Olga González-Valle^7^, Francisco José De Abajo^8^

###### ^1^Drug Hypersensitivity Laboratory, Hospital La Paz Institute for Health Research-IdiPAZ, Madrid, Spain; ^2^Clinical Pharmacology Unit, Principe de Asturias University Hospital, University of Alcalá (IRYCIS), Alcalá De Henares, Madrid, Spain; ^3^Clinical Pharmacology Unit, Principe de Asturias University Hospital, University of Alcalá (IRYCIS), Alcalá De Henares, Madrid, Spain; ^4^Clinical Pharmacology Department, La Paz University Hospital, Madrid, Spain; ^5^Dermatology Department, University Hospital of Getafe, Getafe, Madrid, Spain; ^6^Allergy Department, La Paz University Hospital, Madrid, Spain; ^7^Dermatology Department, University Hospital of Getafe, Getafe, Madrid, Spain; ^8^Clinical Pharmacology Unit, Principe de Asturias University Hospital, University of Alcalá (IRYCIS), Alcalá De Henares, Madrid, Spain

**Correspondence:** Teresa Bellon - teresa.bellon@salud.madrid.org

*Clinical and Translational Allergy* 2018, **8(Suppl 3)**:P07

**Background**

Epidermal Necrolysis (EN; a common denomination for Stevens–Johnson Syndrome (SJS), toxic epidermal necrolysis (TEN) and overlap phenotypes) is the most severe cutaneous adverse drug reaction (cADR). The identification of culprit drugs is often a problem in polymedicated patients. The probability algorithm for drug causality in EN (ALDEN) establishes different categories of causality after scoring of chronology, pharmacokinetics, pre-challenge, notoriety, and alternative causes. Causality is qualified as *very probable* (ALDEN score ≥ 6), *probable* (4–5), *possible* (2–3), *unlikely* (0–1) or *very unlikely* (< 0).

The lymphocyte transformation test (LTT) has been used as a tool to evaluate drugs involved in cADR. LTT specificity and sensitivity are difficult to ascertain as the gold standard (re-challenge) is not acceptable in severe cases. Previous publications suggest that sensitivity of LTT is low in the recovery phase in EN (Kano Y et al. Allergy 2007).

**Methods**

LTT assays were performed (from 2010 to 2017) with suspected drugs after recovery in 23 validated EN cases included in the Spanish registry PIEL*enRED*. A total of 49 drugs were tested. Among them only 13 were performed with highly suspected drugs (3 allopurinol/oxypurinol, 9 aromatic anticonvulsants, 1 sulfasalazine). LTT results were considered positive if stimulation indexes (S.I.) were ≥ 2 in at least one concentration tested, except for betalactam antibiotics (S.I. ≥ 3). ALDEN was applied and suspected drugs were divided into two categories: unrelated (ALDEN score < 4) and related (ALDEN score ≥ 4). Contingency tables and Fisher exact test were used to analyze the data.

**Results**

Seventeen drugs scored as probable or very probable (ALDEN ≥ 4). Positive LTT results were obtained for 23 drugs in 21 patients. In 14 out of 23 positive LTT assays the drugs were related to the adverse reaction (ALDEN score ≥ 4). Twenty three out of the 25 drugs that tested negative were predicted to be unrelated to the adverse reaction (ALDEN score < 4). A contingency table was built (Table 1).

**Table 1:** Contingency table. Drug causality results in EN patients

	ALDEN results		Sum
	Related drug (ALDEN ≥ 4)	Unrelated drug (ALDEN < 4)	
Positive LTT	14	9	23
Negative LTT	3	23	26
Sum	17	32	49^a^

p-value = 0.001 (Fisher’s exact test)

LTT sensitivity = 82.4%; Specificity = 71.9%; PPV = 60.9%; NPV = 88.5%

k = .501: 95% CI: 0.194–0.679

^a^Data from 23 EN cases

Fisher’s exact test was used to analyze the data (p = 0.001). The Cohen’s kappa statistic (k) for agreement was 0.501. When ALDEN was considered as the gold standard, 82.4% sensitivity and 71.9% specificity were obtained for LTT assays, with a positive predictive value (PPV) = 60.9% and a negative predictive value (NPV) = 88.5%.

**Conclusion**

Although this is a small case series, to our knowledge is the largest series of LTT reported in EN cases. The results indicate that contrary to previous reports, LTT is a sensitive tool in the recovery phase of EN.

#### P08 Delayed cutaneous adverse drug reactions to macrolides, clindamycin and pristinamycin: cross-reactions and value of patch-tests

##### Maya El Khoury, Haudrey Assier, Gwendeline Gener, Cynthia Haddad, Olivier Chosidow, Pierre Wolkenstein, Saskia Oro

###### Henri Mondor Hospital, Créteil, France

**Correspondence:** Saskia Oro - saskia.oro@aphp.fr

*Clinical and Translational Allergy* 2018, **8(Suppl 3)**:P08

**Background**

Despite different biochemical structures, macrolides, lincosamides (including clindamycin) and streptogramins (including pristinamycin) are grouped in the same family, the MLS family because of their common mechanism of action. They may induce delayed cutaneous adverse drug reactions (DCADR). We aimed to investigate the possibility of cross-reactivity between MLS antibiotics and to compare the value of patch-tests (PT) in macrolides, clindamycin and pristinamycin-induced DCADR.

**Methods**

This retrospective, monocenter study was conducted between 1997 and 2016. We included all patients with any MLS drug suspected-DCADR, alone or with other culprits, and who had PT performed according ESCD guidelines after this DCADR. Between 1997 and 2012, PT involved the suspected drug(s) only. From 2012, a complete investigation of MLS, i.e. PT to macrolides, clindamycin and pristinamycin, was performed for any MLS-suspected DCADR. Patients with negative rechallenge of the culprit MLS or with positive PT to another culprit drug were excluded. Data were obtained from charts: age, gender, clinical characteristics, presence or absence of other culprit drugs, results of the PT. Prior or subsequent intake (with specifying the tolerance) of any of MLS antibiotics were recorded by phone call to reachable patients in 2016.

Cross-reactivity between drugs of the MLS family was defined by: 1/a positive PT to ≥ 2 MLS of different subclasses; and/or 2/by a clinical eruption to a MLS of a different subclass before or after the DCADR investigated by PT.

**Results**

Seventy-one patients were included (49F, mean age 55) were included. Among the 25 patients with PTs performed for the 3 MLS subclasses, 2 (8%) had cross-reactivity by PT (1 positive to pristinamycin and erythromycin and 1 to clindamycin and pristinamycin). Among the 8 patients who declared previous or subsequent intake of another MLS drug, 2 (25%) had clinical cross-reactivity (both with pristinamycin as culprit, who had maculopapular exanthema to macrolide). Type of DCADR and positivity of PT are in the Table 1. PT were significantly more often positive in pristinamycin (19/41, 46.3%) than in macrolide (2/20, 10%) or clindamycin-suspected (3/10, 30%) DCADR (p = 0.015).

**Table 1:**
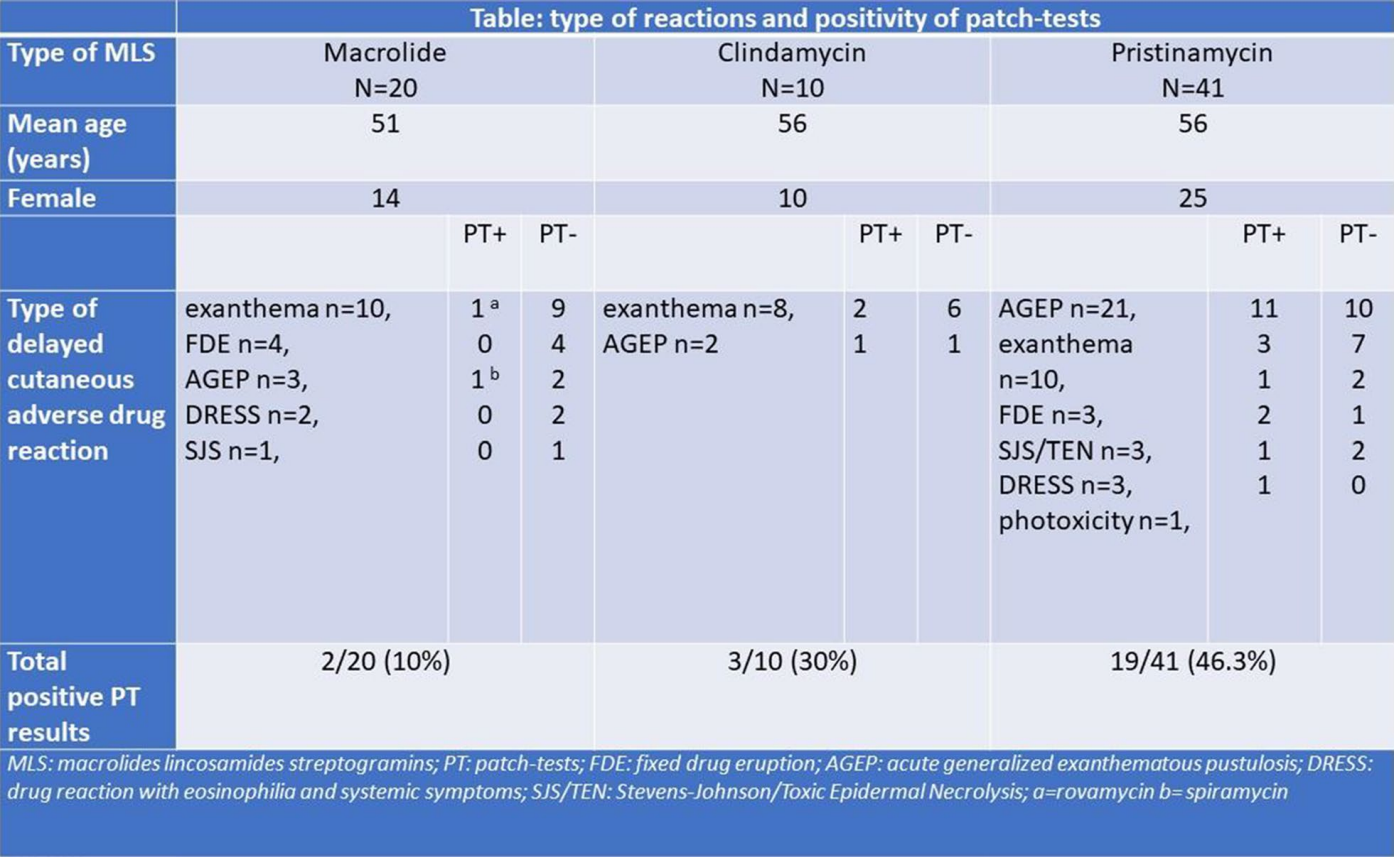
Type of reactions and possibility of patch-tests

**Conclusion**

We describe cases of cross-reactions between MLS antibiotics. Interestingly, clinical or PT cross-reactivity was already described with other drugs which have, as in MLS antibiotics, a different structure but a common mechanism of action (febuxostat/allopurinol, lamotrigine/aromatic anticonvulsivants).

Facing a MLS-induced DCADR, a complete allergologic exploration followed by a supervised oral challenge is warranted to allow safe further intakes of these commonly used antibiotics.

#### P09 ICD-9 coding – useless in identifying SJS/TEN?

##### Leonor Carneiro-Leão, Maria João Vasconcelos, Josefina Cernadas

###### Serviço de Imunoalergologia, Centro Hospitalar de São João, Porto, Portugal

**Correspondence:** Leonor Carneiro-Leão - leonorcarneiroleao@gmail.com

*Clinical and Translational Allergy* 2018, **8(Suppl 3)**:P09

**Background**

Steven–Johnson syndrome (SJS) and toxic epidermal necrolysis (TEN) are rare acute severe cutaneous adverse reactions. They are believed to be a continuum of the same disease and, independent from Erythema Multiform (EM). However, the 3 entities are frequently confused by clinicians and coding systems like ICD-9, which categorizes SJS, TEN and SJS/TEN overlap as subcategories of EM.

**Methods**

Medical records of all patients admitted to a Central University hospital from 2011 to 2015, receiving the ICD-9 codes 695.10 (EM, unspecified), 695.11 (EM minor), 695.12 (EM major), 695.13 (SJS), 695.14 (SJS-TEN overlap syndrome), 695.15 (TEN) and 695.19 (other EM) were reviewed. Data including demographics, suspected drug allergy, treatment approach and allergologic study was extracted. Medical records were reviewed by 2 physicians whom validated the cases as possible or not probable SJS/TEN.

**Results**

Of 74 feasible cases, 3 were excluded (lack of data). The final sample included 39 females (54.9%), median age of 49 (IQ range = 48). Patients were classified by ICD-9 coders as follows: 24 EM unspecified (695.10), 0 EM minor (695.11), 3 EM major (695.12), 16 SJS (695.13), 1 SJS-TEN overlap (695.14), 23 TEN (695.15) and 4 other EM (695.14).

The review of clinical data confirmed cases as possible SJS/TEN in 23 (3 EM unspecified, 1 EM major, 8 SJS, 1 SJS/TEN overlap, 10 TEN).The group of SJS/TEN possible cases included 16 females (69.6%) and the median age was 68 (IQR = 28); 7 patients died, all coded as TEN. SJS/TEN developed during hospital stay in 3 patients initially admitted for another problem. Systemic steroids were used in 13 patients, topical steroids in 5, IV IgG in 2; ciclosporin A was never used.

In 3 patients no information about suspected drug was available; in 8 patients > 1 drug was suspected. The most frequently suspected drugs were β-lactams. Only 2 patients were examined by Immunoallergology (IA) and 18 by Dermatology; 1 was referred to an IA and 2 to Dermatology follow-up after discharge.

**Conclusion**

Although SJS/TEN is rare, the potential severity of the disease makes a correct approach crucial. Despite > 1 drug was suspected in 35% of cases, an IA observation was rarely granted and drug allergy study was seldom undertaken. Many cases were misclassified by ICD-9 coders: 4 with possible SJS/TEN were classified as EM unspecified/major and 21 codified as SJS/TEN were considered not probable. These findings should alert us to the importance of competent training of coders.

**Consent to publish**

Consent to publish was obtained from the patient involved in this study.

#### P10 Liver involvement complicating SJS/TEN in an HIV endemic setting

##### Niita Haitembu, Wisdom Basera, Rannakoe Lehloenya, Jonny Grant Peter

###### University of Cape Town, Cape Town, South Africa

**Correspondence:** Jonny Grant Peter - Jonny.Peter@uct.ac.za

*Clinical and Translational Allergy* 2018, **8(Suppl 3)**:P10

**Background**

In HIV endemic settings, common culprit drugs causing SJS/TEN include nevirapine, cotrimoxazole and first-line anti-tuberculosis drugs. Hepatitis is an uncommon complication of SJS/TEN and may increase mortality. In our clinic, where ~ 80% of SJS/TEN cases occur in persons living with HIV, a significant proportion have hepatitis. The aim of this study was to determine the incidence of hepatitis and impact on outcome, as well as characterize the clinical features and patterns of liver injury.

**Methods**

We conducted a retrospective clinical record review of all patients admitted to the tertiary dermatology service at Groote Schuur Hospital, Cape Town, South Africa, over a 10-year period (2005–2015). All available clinical and laboratory variables were collected, to where possible allow Naranjo and ALDEN drug causality assessment. Classification and severity of liver injury used criteria from ICM-CADALI and the CTCAE of the National Cancer Institute for adverse events version 4.03.

**Results**

Of the 184 SJS/TEN patients admitted, 77.2% (142/184) were HIV infected, with a median (IQR) CD4 count of 185 (97–264) cells/mm^3^. SJS was the most frequent phenotype (56%, 103/184). The leading causative drugs were: Nevirapine 80/184 (43.5%), cotrimoxazole 35/184 (19.0%), and anti-TB drugs 12/184 (6.5%). 21.2% (37/184) had liver injury, with 28/37 (75.7%), 4/37 (10.8%) and 5/37 (13.5%) having a hepatocellular, cholestatic and mixed picture respectively. Of the 37 patients with liver injury, there was only one with pre-existing hepatitis B or other pre-existing chronic liver disease. CTCAE severity grades 1, 2, 3 and 4 occurred in 46.0%, 21.6%, 27.0% and 5.4% having life threatening liver injury.

**Conclusion**

Hepatitis amongst SJS/TEN in HIV endemic settings is common, occurring in more than one in five patients. The majority of hepatitis is of mild to moderate severity and death from liver failure did not occur.

## Thursday 19 April 2018

### Skin reactions in drug allergy - Poster Walk 2

#### P11 Retrospective analysis of SJS/TEN cases reveals need for diagnostic test to determine culprit drug

##### George A. Romar^1^, Ruth K. Foreman^2^, Sherrie J. Divito^1^

###### ^1^Brigham and Women’s Hospital, Boston, MA, United States; ^2^Massachusetts General Hospital, Boston, MA, United States

**Correspondence:** Sherrie J. Divito - sdivito@bwh.harvard.edu

*Clinical and Translational Allergy* 2018, **8(Suppl 3)**:P11

**Background**

Stevens–Johnson Syndrome/Toxic Epidermal Necrolysis (SJS/TEN) is a severe form of delayed-type hypersensitivity reaction with high morbidity and mortality and no treatment. Though most cases are attributed to drug (rather than infection), there is currently no diagnostic test to determine which drug is responsible. The inability to determine culprit drug negatively impacts patient care. Anecdotally, patients are incorrectly labelled allergic to multiple drugs resulting in unnecessary treatment with second or third-line agents that are more expensive, less effective and/or have higher side effect profiles. Alternatively, not labeling a patient allergic to the true culprit drug could result in dangerous re-exposure. Surprisingly, there is little published data on clinicians’ ability to identify culprit drug and its impact on care.

**Methods**

We performed a retrospective study of SJS/TEN overlap (10–30% TBSA) and TEN (> 30% TBSA) cases at Brigham and Women’s Hospital and Massachusetts General Hospital over a sixteen-year period. All cases were confirmed histologically by a dermatopathologist and clinically confirmed by a dermatologists with expertise in the field. SJS cases (< 10% TBSA) were excluded due to variability of diagnosis amongst dermatologists.

**Results**

There were 43 SJS/TEN overlap/TEN cases available for review. Of these 43, only 3 patients (6.9%) were taking one drug at the time of or immediately preceding the reaction, while 29 patients (67%) were taking ≥ 4 drugs at the time of or immediately preceding the reaction. Only 10 cases had “clear” drug etiology. Clinicians’ determination of a “clear” culprit drug was based on retrospective epidemiologic data of most common drug causes. No cases utilized a drug scoring system or drug half-life in determining culprit drug. Seventeen patients (39.5%) were labelled as allergic to 2 or more drugs (potential false-positive cases). In some cases, whole classes of drugs were listed as allergies-for example, all beta-lactams to a clear reaction to amoxicillin–clavulanate. Twenty-three patients (53%) were listed as allergic to only 1 drug, despite the fact that most of these patients had other potential culprit drugs (potential false-negative cases) and this resulted in one death. There were 17 cases with infection prior to the reaction (excluding patients with upper respiratory symptoms (n = 8) which could be initial features of SJS/TEN), yet all 17 cases were attributed directly to drug.

**Conclusion**

Development of a diagnostic test to determine culprit drug in SJS/TEN is a major unmet clinical need.

#### P12 “Look-alike” and “sound-alike” medicines: a potential cause for cutaneous adverse reactions to drugs

##### Charles Cassius^1^, Colin Davis^2^, Pierre Bravard^3^, Delphine Carre-Gislard^3^, Philippe Modiano^4^, Saskia Oro^1^, Olivier Chosidow^1^

###### ^1^Dermatology Department, Mondor Hospital, Créteil, France; ^2^School of Experimental Psychology, University of Bristol, Bristol, United Kingdom; ^3^Dermatology Department, Monod Hospital, Le Havre, France; ^4^Dermatology Department, Saint-Vincent de Paul Hospital, Lille, France

**Correspondence:** Saskia Oro - saskia.oro@aphp.fr

*Clinical and Translational Allergy* 2018, **8(Suppl 3)**:P12

**Background**

Medication error can be due to delivery problem potentially leading to the deliverance of a wrong drug. Twenty-five percent of these errors have been attributed to orthographic, i.e., “look-alike” and phonetic i.e., “sound-alike” (LASA) similarity between drug names. Cutaneous adverse drug reactions (CADR) may be severe, as toxic epidermal necrolysis (TEN) or drug reaction with eosinophilia and systemic symptoms (DRESS), and could be due to medication error. We aimed to collect cases of CADR due to medication error and to analyze the pharmacopeia to identify risky LASA drug names.

**Methods**

We collected cases of CADR due to medication error from the French authorities’ Pharmacovigilance databases between 1985 and 2016 and from the French Investigators for skin adverse reaction to drugs group of the French society of Dermatology. Doubles were excluded. We performed a systematic orthographic comparison of TEN high-risk drugs with the pharmacopeia in France.

For this comparison, we used a search string within the standardized MedDRA Queries “acute cutaneous adverse reaction” (“drug error”, “overdosage” and “accidental overdosage”) with Boolean operators “AND” and “OR”.

Comparison was made with a string-matching algorithm called “superposition matching”. It allows a match score of between 0 and 1 to be computed for any pair of letter strings, where a value of 0 is considered not risky and 1 considered highly risky.

**Results**

Eight cases were included, 4 severe CADR (3 SJS/TEN, 1 DRESS) due to erroneous substitution of LAMISIL^®^ (terbinafine) by LAMICTAL^®^ (lamotrigine) and 4 non-severe CADR due to erroneous substitution of LAMISIL^®^ (terbinafine) by LAMICTAL^®^ (lamotrigine) in 2 cases, of METEOXANE by METHOTREXATE in 1 case and of DAKTARIN^®^ by DAKIN^®^. Four hundred and forty-six risky homologies (score > 0.5) and 35 highly risky homologies were identified (Table 1) (score > 0.7). Homologies were mainly due to brand name compared to international nonproprietary names and were mainly antibiotics.

**Table 1:** Results

Drug at risk for confusion	MATCH SCORE	Inducing drug
ACIDRINE^®^	0.72	ADIAZINE
ADENYL	0.71	GARDENAL
ADEPAL^®^	0.79	ALEPSAL
ADEPAL^®^	0.71	GARDENAL
ARESTAL^®^	0.74	ALEPSAL
ARTHROCINE^®^	0.75	AZITHROMYCINE
ARTHROCINE^®^	0.77	CLARITHROMYCINE
ARTHROCINE^®^	0.82	ERYTHROCINE
AZATHIOPRINE	0.72	AZITHROMYCINE
CANOL^®^	0.82	CYCLADOL
CARDIOXANE^®^	0.71	CEFTRIAXONE
CEFALINE^®^	0.81	CEFALEXINE
CEFALINE^®^	0.7	CEFIXIME
CLARADOL^®^	0.72	CYCLADOL
DOXYLAMINE^®^	0.72	DOXYCYCLINE
ERYTHROCINE^®^	0.75	ARTHROCINE
IMNOVID^®^	0.74	INDOCID
LAMISIL^®^	0.75	LAMICTAL
LAROXYL^®^	0.78	CLAMOXYL
LECTIL^®^	0.71	TILCOTIL
LIDENE^®^	0.76	LODINE
MELODIA^®^	0.75	MELOXICAM
MINOCYCLINE^®^	0.71	MIDECAMYCINE
MOCLAMINE^®^	0.72	MINOCYCLINE
NEORAL^®^	0.71	KEFORAL
NIVAQUINE^®^	0.74	NEVIRAPINE
PEFLACINE^®^	0.71	PYOSTACINE
PHYSIOMYCINE^®^	0.71	PRISTINAMYCINE
RITALINE^®^	0.78	SERTRALINE
ROVALCYTE^®^	0.76	ROVAMYCINE
ROVAMYCINE^®^	0.72	VIBRAMYCINE
SPIRAMYCINE	0.78	PRISTINAMYCINE
SPIRAMYCINE^®^	0.75	VIBRAMYCINE
TEMODAL^®^	0.78	TEXODIL
VIBRAMYCINE^®^	0.75	SPIRAMYCINE

**Conclusion**

We confirm that some CADR may be due to drug substitution favored by LASA. Our study highlights the existence of risky homologies between drug names which could lead to CADR. The study must lead to a public policy of risk reduction (i.e. systematic use of algorithm before new drug authorization, informatics prescription, etc.), as CADR are avoidable and potentially severe adverse reactions.

#### P13 Serum thymus and activation-regulated chemokine (TARC) is a useful marker for assessing the clinical and immunological condition of DRESS/DIHS patients

##### Hideo Hideo, Yuki Nakamura-Nishimura, Fumi Miyagawa, Kazuya Miyashita, Rie Ommori, Hiroaki Azukizawa

###### ^1^Dept of Dermatol, Nara Medical University, Kashihara-City, Japan

**Correspondence:** Hideo Hideo - asadah@naramed-u.ac.jp

*Clinical and Translational Allergy* 2018, **8(Suppl 3)**:P13

**Background**

Drug reaction with eosinophilia and systemic symptoms/drug-induced hypersensitivity syndrome (DRESS/DIHS) is a severe adverse drug-induced reaction with reactivation of human herpesvirus 6 (HHV-6). We previously reported that serum thymus and activation-regulated chemokine (TARC) levels were markedly increased in patients with DRESS/DIHS and suggested TARC as a useful diagnostic marker of DRESS/DIHS in the early stage. In this study, we determined whether serum TARC levels correlate with the severity of clinical symptoms and laboratory data in patients with DRESS/DIHS.

**Methods**

We evaluated 16 patients with DRESS/DIHS for their clinical symptoms, laboratory data, copy numbers of HHV-6 and human cytomegalovirus (CMV) DNA in the peripheral blood mononuclear cells, serum cytokines and soluble interleukin-2 receptor (sIL-2R), as well as serum TARC.

**Results**

The levels of serum TARC in DRESS/DIHS were correlated with the severity of skin and mucosal lesions, fever, dysfunction of liver and kidney, and levels of HHV-6 and CMV DNA, IL-5, IL-10, and sIL-2R.

**Conclusion**

Our results suggest that TARC might be not only a diagnostic marker but also a useful marker for assessing the clinical and immunological condition of patients.

#### P14 A retrospective analysis of drug reaction with eosinophilia and systemic symptoms (DRESS) related myocarditis in Singapore general hospital

##### Rachel Wenrui Lim^1^, Chong En Linus Chan^2^, Yi Wei Yeo^1^, Karen Choo^1^, Shiu Ming Pang^1^, Haur Yueh Lee^1^

###### ^1^Singapore General Hospital, Singapore, Singapore; ^2^Duke-NUS Medical School, Singapore, Singapore

**Correspondence:** Rachel Wenrui Lim - limwrachel@gmail.com

*Clinical and Translational Allergy* 2018, **8(Suppl 3)**:P14

**Background**

DRESS (Drug reaction with eosinophilia and systemic symptoms) is a severe multi-organ adverse drug reaction. Cardiac involvement in DRESS is uncommon, with a prevalence of 4–27% of cases. Clinical symptoms are similar to that of acute coronary syndrome and it is often difficult to distinguish the two. The aim of our study is to determine the incidence of cardiac involvement in a cohort of DRESS patients, their clinical course and outcome.

**Methods**

Cases of probable and definite cases of DRESS (based on RegiSCAR scoring) who were admitted to the Singapore General Hospital from 2009 to 2017 were retrospectively analysed. Inclusion criteria for cardiac involvement in DRESS were cases who met all of the following criteria: (a) Clinical symptoms suggestive of cardiac disease, (b) raised cardiac enzymes and (c) abnormal ECG changes and/or cardiac imaging. Exclusion criteria were (a) prior history of ischemic heart disease and (b) onset of cardiac presentation later than 6 months of index date unless there are other clinical features of ongoing DRESS.

**Results**

There were a total of 100 cases of probable and definite DRESS during the study period. A total of 7 cases met the inclusion/exclusion criteria for DRESS-related cardiac involvement. There were 6 females and 1 male, with a mean age of 70 years old. 42.9% had echocardiographic changes and 1 patient underwent endomyocardial biopsy with findings consistent with myocarditis. 71.4% were treated with concurrent systemic corticosteroids while 100% received topical corticosteroids. 5 out of 7 required ICU and mortality was 42.9%. Viral reactivation occurred in 5 out of 7 cases and the most common culprit drug was allopurinol (71.4%).

**Conclusion**

DRESS-related myocarditis is uncommon however prognosis is poor with 42.9% mortality. Definitive diagnosis via endomyocardial biopsy is rarely performed. The identification of risk factors for cardiac involvement and ideal treatment strategy for such complications remain a research priority.

#### P15 Association of HLA-B allele with aromatic antiepileptic drugs induced cutaneous adverse drug reactions in Thai population

##### Napatrupron Koomdee^1^, Suthida Sririttha^1^, Chonlawat Chaichan^1^, Kanuengnit Choochuay^1^, Jettanong Klaewsongkran^2^, Ticha Rerkpattanapipat^1^, Kittika Yampayon^3^, Pornpimol Kijsanayotin^3^, Chonlaphat Sukasem^1^

###### ^1^Faculty of Medicine Ramathibodi Hospital, Mahidol University, Bangkok, Thailand; ^2^Faculty of medicine, Chulalongkorn University, Bangkok, Thailand; ^3^Faculty of Pharmaceutical Sciences, Chulalongkorn University, Bangkok, Thailand

**Correspondence:** Napatrupron Koomdee - kerk_8@hotmail.com

*Clinical and Translational Allergy* 2018, **8(Suppl 3)**:P15

**Background**

Aromatic antiepileptic drugs (AEDs) are commonly prescribed to prevent or treat the seizure. Antiepileptic therapies are associated with a high incidence of Stevens–Johnson syndrome (SJS) and toxic epidermal necrolysis (TEN). A strong association between *HLA*-B*15:02 allele and carbamazepine (CBZ)-induced SJS/TEN has been first proved in Han Chinese, which has been confirmed in other Southeast Asian countries where the allele is prevalent. Here, we extend the study of HLA-B susceptibility to AEDs-induced cADRs in the various phenotypes in Thai patients.

**Methods**

We carried out a case–control association study. One-hundred sixty-six patients with AED-induced cADRs were classified to 71 MPE, 49 DRESS, and 45 SJS/TEN, 426 AED-tolerant controls who were on the drug, respectively, for more than 6 months without the adverse reactions, and 470 normal subjects from the general Thai population. The *HLA*-*B* genotype was amplified by the polymerase chain reaction-sequence specific oligonucleotide primers.

**Results**

We found that *HLA*-B*15:02 and *HLA*-B*51:01 were present in 46 out of 166 (27.7%) and 21 out of 166 AED-induced cADRs compare to all AED tolerant control (OR: 4.282; 95% CI: 2.63–6.95; p < 0.001), (OR: 2.292; 95% CI: 1.31–4.00; p = 0.003), respectively. The association of *HLA*-B*15:02 in all AED-induced MPE (26.8%) and all AED tolerant control (8.2%), (OR: 4.30; 95% CI: 2.29–8.06; p < 0.001) and Thai populations (15.1%), (OR: 2.05; 95% CI: 1.14–3.67; p = 0.014). The *HLA*-*B* alleles which were found the significant association with AEDs-induced SJS/TEN were *HLA*-B*15:02 (OR: 10.79; 95% CI: 5.50–21.16; p < 0.001) and *HLA*-B*15:21 (OR: 10.636; 95% CI: 1.46–77.35; p = 0.042). In addition, the significant association of *HLA*-B*08:01, HLA-B*13:01 and *HLA*-B*56:02 in all AED-induced DRESS were detected (OR: 18.085; 95% CI: 1.60–203.23; p = 0.029), (OR: 2.488; 95% CI: 1.26–4.90; p = 0.007), (OR: 9.190; 95% CI: 1.80–46.88; p = 0.017), respectively.

**Conclusion**

Our study indicates that carbamazepine (CBZ) and lamotrigine (LTG) causing cADRs in *HLA*-B*15:02 carriers may act on a similar pathogenetic mechanism, although other genetic/nongenetic factors may also contribute to the pathomechanism of the disease. Moreover, we found the association of *HLA*-B*15:21 which the relationship of *HLA*-B*15:02 and *HLA*-B*15:21 is they are in the same group of HLA-B75 serotype (*HLA*-B*15:02, B*15:08, B*15:11, B*15:21). Therefore, we suggest to avoid CBZ and LTG in those who carrier *HLA*-B*15:02 and HLA-B75 serotype group and also *HLA*-B* 56:02 for PHT-induced DRESS, *HLA*-B* 51:01 for PHT-induced SJS/TEN as well. Phenobarbital (PB) is the same small sample size as LTG and OXC that is a limitation of the study.

#### P16 Single nucleotide polymorphisms (SNPs) for prediction allopurinol-induced CADR in Thai population

##### Santirhat Prommas^1^, Gaidganok Sornsamdang^1^, Ekawat Pasomsub^1^, Patompong Satapornpong^1^, Thawinee Jantararoungtong^1^, Apichaya Puangpetch^1^, Napatrupron Koomdee^1^, Jirawat Pratoomwan^1^, Kanuengnit Choochuay^1^, Therdpong Tempark^2^, Jettanong Klaewsongkram^2^, Ticha Rerkpattanapipat^1^, Chonlaphat Sukasem^1^

###### ^1^Mahidol University, Bangkok, Thailand; ^2^Chulalongkorn University, Bangkok, Thailand

**Correspondence:** Santirhat Prommas - namfar_p11@hotmail.com

*Clinical and Translational Allergy* 2018, **8(Suppl 3)**:P16

**Background**

Allopurinol, a xanthine oxidase inhibitor in purine catabolism is commonly prescribed in patients who have hyperuricemia with gouty arthritis, and can be used prophylactically to prevent chemo-therapy-induced hyperuricemia. Cutaneous Adverse Drug Reactions (CADRs) cause significant morbidity and mortality for these patients. The CADRs, involving delayed immune-mediated mechanisms present under different clinical patterns that have been very well characterized phenotypically, include urticarial, Stevens–Johnson syndrome (SJS), toxic epidermal necrolysis (TEN), drug reaction with eosinophilia and systemic symptoms (DRESS) and maculopapular exanthema (MPE). *HLA*-B*5801 is a strong genetic-marker for allopurinol-induced CADR, although the allopurinol-induced CADR can be found in patients without *HLA*-B*5801. Conversely, the patient who treated with allopurinol for long term without CADR might be a carrier of *HLA*-B*5801.

**Methods**

Patients with allopurinol-induced CADR during the period 2011–2016 were enrolled in the Thai Severe Cutaneous Adverse Reaction (THAI-SCAR) Project and patients admitted to the allergy clinic of the Faculty of Medicine, Ramathibodi Hospital, Mahidol University. Fifty seven patients were categorized into SJS/TEN (25 cases), DRESS (24 cases) and MPE (8 cases). Patients who had been taking allopurinol for more than 6 months with no evidence of cutaneous adverse drug effect were recruited as allopurinol-tolerant controls (n = 101). All CADR patients were assessed by a dermatologist and allergist. The phenotypes were classified using the criteria of the RegiSCARs. Fifteen SNPs, rs2734583 (A > G; *BAT1*), rs3099844 (C > A; *HCP5*), rs9263726 (G > A; *PSORS1C1*), rs2233945 (C > A; *PSORS1C1*), rs9263733 (C > T; *POLR2LP*), rs9263745 (G > A; *CCHCR1*), rs130077 (G > A; *CCHCR1*), rs9263785 (T > G; (G > A; *CCHCR1*), rs9263794 (A > G; *TCF19*), rs1044870 (C > T; *TCF19*), rs9263796 (C > T; *POU5F1*), rs4084090 (A > G; *HLA*-*C*), rs3131643 (G > A; *HCP5*), rs3117583 (A > G; *BAT3*), rs1150793 (A > G; *MSH5*), were assayed by Taqman real-time PCR Viia7 (ABI, Foster City CA, USA).

**Results**

A significant association was found for *HCP5* rs3099844, *PSORS1*C1 rs9263726, *POLR2LP* rs9263733 and *CCHCR1* rs9263745; odds ratio 73.2, 95% CI 24.2–266.8, p-value 1.9 × 10^−24^. The sensitivity, specificity, PPV and NPV of these combinations of SNPs were 89.5, 90.1, 83.6 and 93.8. Although analysis of SNP linkage disequilibrium (LD) compared with *HLA*-B*5801 was not absolute LD, the combination could detect variance in 89.5% (51/57). Furthermore, the combination of SNPs could be used as a genetic-marker for Allopurinol-induced CADR in Thai population.

**Conclusion**

All fifteen SNPs were associated with allopurinol-induced CADR. They can use as alternative maker to predict risk from administration of allopurinol, which depend on an objective of the analysis. But each single SNP cannot replace HLA-B*5801 for prevent risk from allopurinol administration.

#### P17 HLA-A*31:01 and Carbamazepine-induced DRESS Syndrome in Tunisian population

##### Zohra Chadli^1^, Amira Djobbi^1^, Emna Kerkeni^1^, Amel Chaabane^1^, Nadia Ben Fredj^1^, Najeh Ben Fadhl^1^, Naceur A Boughattas^1^, Karim Aouam^1^, Imen Sfar^2^

###### ^1^Pharmacology Laboratory, Faculty of Medicine, University of Monastir, University Hospital Of Monastir, Monastir, Tunisia; ^2^Immunology Laboratory, University Hospital of Charles Nicolle, Tunis, Tunisia

**Correspondence:** Zohra Chadli - zohrachadly@ymail.com

*Clinical and Translational Allergy* 2018, **8(Suppl 3)**:P17

**Background**

Drug rash with eosinophilia and systemic symptoms (DRESS) is a severe idiosyncratic adverse drug reaction with multiple-organ involvement. The most common drugs induced DRESS are antiepileptic drugs (carbamazepine, phenobarbital and phenytoin). The HLA-A*31:01 allele has been shown to be strongly correlated with carbamazepine-induced DRESS. Objective: The aim of this study was to evaluate the correlation between the HLA-A*31:01 and carbamazepine induced DRESS in Tunisian population.

**Methods**

A total of 11 patients with carbamazepine-induced DRESS syndrome and 44 healthy Tunisian subjects were included in this study. Diagnosis of DRESS was based on European *RegiSCAR* criteria. Imputability was established according to *Begaud’s* method. Informed written consent was obtained from all subjects. The genomic DNA was extracted from peripheral blood mononuclear cells using a salting-out procedure. HLA-A*31:01 typing was performed using polymerase chain reaction-restriction fragment length polymorphism (PCR–RFLP).

**Results**

In patients group, sex-ratio was 36.4: 4 men and 7 women, with a mean age of 39 years ± 22.6 (6–74 years). No significant differences between patients and controls groups were observed regarding sex-ratio and mean age. All patients had pruritic maculopapular rash involving more than 50% of their body surface area. Mucosal involvement was observed in two patients. Eosinophilia was observed in 9 patients. Liver was the most common organ affected in our series. The mean incubation period was 2 weeks. The evolution was favorable after carbamazepine withdrawal in all cases.

The HLA-A*31:01 allele was detected in 63.6% of cases (7/11) and only in 0.9% of controls subjects (4/44) (P < 10^−3^). Significant association was detected between HLA-A*31:01 and carbamazepine-induced DRESS syndrome [P < 10^−3^; odds ratio (OR): 17.5; 95% confidence interval (CI): 3.5–86.6].

**Conclusion**

Similarly to European and Japanese populations, the presence of the HLA-A*31:01 allele was strongly associated with carbamazepine-induced DRESS syndrome in a sample of Tunisian population.

#### P18 Dress and staphylococcal scalded skin syndrome: two simultaneous severe skin diseases in an immunosuppressed patient

##### Miriam Barrios Albajar^1^, Marta López San Martín^1^, Maria Antonia González De Domingo^2^, Veronica López Couso^1^, María Del Mar Reaño Martos^1^, Beatriz Torres Pérez^1^, Lucía Indira Melgar González^1^

###### ^1^Servicio de Alergología, Hospital Universitario Puerta de Hierro-Majadahonda, Madrid, Spain; ^2^Servicio de Dermatología, Hospital Universitario Puerta de Hierro-Majadahonda, Madrid, Spain

**Correspondence:** Miriam Barrios Albajar - miriam.barrios22@gmail.com

*Clinical and Translational Allergy* 2018, **8(Suppl 3)**:P18

**Background**

DRESS is a rare, severe drug induced hypersensitivity reaction that includes skin eruption, hematologic abnormalities, lymphadenopathy and internal organs involvement. Allopurinol is one of the most common offending drugs in DRESS. Complications can sometimes arise that difficult to diagnose and imply an increased risk for the patient.

**Case Report**

A 41 years-old woman with history of autoimmune disease and multi-organ involvement, in treatment with steroids and on the waiting list for heart transplant, presented to the emergency room 4 weeks after she started treatment with Allopurinol 100 mg/day, for asymptomatic hyperuricemia.

She complained of a pruritic skin rash, starting on the face, neck and chest, presenting severe edematous flushed face with perioral prominence, blepharoconjunctivitis and lips affectation, without progressing to erosions. Red raised papules spread to her trunk and extremities, progressing to desquamative erythroderma (extension > 50%) with no blisters/bullae and negative Nikolsky sign.

Initial laboratory results revealed eosinophilia (1.050/μL), renal function impairment (serum creatinine 3.7 mg/dl), hyponatremia and hyperkalemia. Exfoliative lesions on the neck were cultivated and methicillin-resistant *S. Aureus* was grown.

At that time, it was thought that this could be a DRESS case with a cutaneous staphylococcal superinfection. Allopurinol was discontinued, intravenous antibiotic treatment was started, steroids dosage was increased along with skin-mucosa cares and close monitoring.

Serological test results were negative for *Mycoplasma pneumoniae* and viral infections (HAV, HBV, HCV, HIV, HSV, parvovirus B19, EBV, CMV…).

The skin biopsy revealed an interface lesion with vacuolar degeneration, lymphocytic infiltration, and spongiosis of the epidermis. An infiltrate with eosinophils was present in the superficial dermis. All these findings were compatible with DRESS. A subcornea blister without perilesional inflammation was also present, which is compatible with Staphylococcal scalded skin syndrome (SSSS).

At least 3 weeks after the discontinuation of Allopurinol, the patient’s symptoms were resolved.

A probable DRESS was diagnosed according to Kardaun criteria (score = 4) and the diagnosis of SSSS was histopathologically and clinically (abortive form) established.

**Conclusion**

We presented a case of DRESS induced by Allopurinol, complicated by SSSS. To our knowledge, this is the first case report describing the coexistence of both entities. Although SSSS is rare in adults, it is mostly observed among patients with increased susceptibility (immunosuppressed individuals due to glucocorticoids treatment), patients with renal impairment that decreases the elimination of the toxin, and patients with lesions in the skin barriers that favor colonization by toxin-produced bacteria. All these conditions were present in our patient.

**Consent to publish**

Consent to publish was obtained from the patient involved in this study.

#### P19 HLA-B*1301-Positive two cases of salazosulfapyridine-induced drug induced hypersensitivity syndrome (DIHS)/drug reaction with eosinophilia and systemic symptoms (DRESS)

##### Masato Kakeda, Masaaki Gyobu, Takahisa Tozawa, Shouhei Iida, Hiroyuki Goto, Koji Habe, Keiichi Yamanaka

###### Department of Dermatology, Mie University Graduate School of Medicine, Tsu, Japan

**Correspondence:** Masato Kakeda - kakeda-m@clin.medic.mie-u.ac.jp

*Clinical and Translational Allergy* 2018, **8(Suppl 3)**:P19

**Background**

Drug-induced hypersensitivity syndrome (DIHS) and drug reaction with eosinophilia and systemic symptoms (DRESS) are serious cutaneous adverse drug reactions (cADRs) characterized by generalized eruption with fever, lymphadenopathy, hematological abnormalities and systemic involvement. Salazosulfapyridine (SASP; also known as sulfasalazine) is an aromatic sulfonamide and commonly prescribed in the treatment of rheumatoid arthritis (RA) and acute inflammatory bowel diseases. It is split into 5-aminosalicylic acid and sulfapyridine in the colon by the bacterial enzyme. SASP causes several ADRs such as hematological dysfunction and DIHS/DRESS. It has been shown that the association between specific HLA allele and the risk factors of cADRs induced by various drugs, such as abacavir, carbamazepine, and allopurinol. A previous publication showed that HLA-B*1301 allele is a genetic marker for dapsone-induced hypersensitivity syndrome of leprosy patients in China, and another report showed that HLA-B*1301 is associated with SASP-induced DRESS in the Han Chinese population. We report HLA-B*1301-positive two cases of SASP-induced DIHS/DRESS.

**Case report**

1st case; A 66-year old Japanese man with RA presented with fever, generalized rash, liver dysfunction, and leucocytosis 31 days after he started taking SASP. He was diagnosed as SASP-induced DIHS/DRESS. SASP was discontinued and systemic administration of prednisolone (PSL) 60 mg/day was initiated, and the systemic reaction was improved. CMV reactivation was seen during the course of treatment. LTT for SASP was positive (Stimulation index (SI) = 2.8).

2nd case; A 66-year old Japanese woman with RA presented with generalized rash with lymphoadenopathy 36 days after initiating SASP. 5 days after SASP was stopped, she presented with fever, leucocytosis, eosinophilia, and elevated liver dysfunction. A clinical diagnosis of DIHS/DRESS was made. SASP was ceased and she started 30 mg/day of prednisolone with gradual improvement of the systemic symptoms. The reactivation of HHV-6 was detected during the course. Negative LTT for SASP (SI = 1.7) but positive LTT for sulfapyridine (SI = 2.7) was detected.

HLA-B*1301 were identified in both two patients of SASP-induced DIHS/DRESS. HLA-B*1301 is present in 1.5% of Japanese population. Neither none of 4 SASP-tolerant control patients nor 15 cADR patients induced by other drugs (4 DIHS, 4 SJS/TEN, 1 AGEP, and 6 other cADRs such as MPE and EEM) was positive for HLA-B*1301.

**Conclusion**

Although the sample size were limited, these findings suggest that in the Japanese population, HLA-B*1301 may be associated with SASP-induced DIHS/DRESS same as Han Chinese and might serve as a genetic marker for SASP-induced DIHS/DRESS.

**Consent to publish**

I confirm that I have received written consent for online publication from all patients described (or their parents/guardians), where there are images, videos, or 3 more indirect identifiers to be published.

#### P20 SDRIFE: Clinical, histological, biological aspects and allergologic explorations. A 13 cases series

##### Tullia De Risi-Pugliese^1^, Aurore Hamelin^1^, Hafida Gaouar^1^, Annick Barbaud^2^, Angèle Soria^3^

###### ^1^Dermatology-Allergology department, Tenon hospital, APHP, Paris, France; ^2^Dermatology-Allergology department, Tenon hospital, APHP; Sorbonne University Paris 06, Paris, France; ^3^Dermatology-Allergology department, Tenon hospital, APHP; Sorbonne University Paris 06; Inserm, Centre d’Immunologie et des Maladies Infectieuses (Cimi-Paris), UMR 1135, Paris, France

**Correspondence:** Annick Barbaud - annick.barbaud@aphp.fr

*Clinical and Translational Allergy* 2018, **8(Suppl 3)**:P20

**Background**

The acronym SDRIFE (symmetrical drug-related intertriginous and flexural exanthema) was introduced by Hausermann et al. in 2004, to describe a flexural drug cutaneous adverse drug reaction. Its management is not yet codified.

**Methods**

We conducted a monocentric retrospective study including all patients suspected to have a SDRIFE between 2008 and 2017 according to the following criteria: (1) exposure to systemically administered drug either following the first or subsequent dose (excluding contact allergens), (2) sharply demarcated erythema of he gluteal/perianal area and/or V-shaped erythema of the inguinal/perigenital area, (3) involvement of at least one other intertriginous/flexural localization, (4) symmetry of affected area, (5) absence of systemic symptoms and signs.

**Results**

13 patients were included, 7 males and 6 females, with a median age of 65 years. They had a macular and/or papular erythema affecting meanly 4 different flexural areas (inguinal 100%, gluteal 62%, axillary 69%, mammary 46%, cervical 38%), without biological abnormalities except a moderate lymphopenia (600–1400/mm^3^, N > 1500/mm^3^) in 5/9 patients. The triggering drug was a iodinated contrast agent in 5 cases, amoxicillin in 3 cases, and another antibiotic in 3 cases. The median occurrence delay after drug exposition was of 24 h (0.5–144) when it was the first episode, and 12 h (2.5–30) for the relapses after a provocation test. Complete healing was obtained in 1–7 days for all patients. The patch tests performed in 3 cases were all negative. The prick tests and intradermal skin tests were positive at 48 h in only 1/9 case.

This is the largest case series of SDRIFE reported to date. In our study, the eruption was almost exclusively limited to the flexural areas, without signs of severity, including when relapsing after a provocation test. The skin tests were mostly negative. Iodinated contrast agents were most frequently responsible for the SDRIFE, before betalactams. This is different from previous studies, in which 50% of skin tests were positive and a betalactam was retained as the triggering drug in more than 50% of the cases. Prospective studies are required to determine the value of skin tests in SDRIFE, according to the triggering drug, and confirm the non severity of SDRIFE even after a new exposition to the offending drug.

**Conclusion**

The SDRIFE is a non-exceptional cutaneous adverse drug reaction, occurring in the 4 days, mostly in the 24 h following systemic exposition to a drug. The skin tests seem to have a low sensitivity.

## Thursday 19 April 2018

### Skin reactions in drug allergy - Poster Walk 3

#### P21 Proven pharmacological interaction concept of HLA-B*1502 and carbamazepine-induced Stevens–Johnson syndrome and toxic epidermal necrolysis: an in Silico model

##### Chonlawat Chaichan^1^, Thanyada Rungrotmongkol^2^, Apichaya Puangpetch^1^, Sirilak Kongkaew^3^, Chonnikan Hanpiboon^4^, Chonlaphat Sukasem^1^

###### ^1^Division of Pharmacogenomics and Personalized Medicine, Department of Pathology, Faculty of Medicine Ramathibodi Hospital, Mahidol University; Laboratory for Pharmacogenomics, Somdech Phra Debaratana Medical Center (SDMC), Ramathibodi Hospital, Bangkok, Thailand; ^2^Faculty of Science, Structural and Computational Biology Research Group, Department of Biochemistry, Chulalongkorn University; Faculty of Science, Program in Bioinformatics and Computational Biology, Chulalongkorn University; Faculty of science, Molecular Sensory Science Center, Chulalongkorn University, Bangkok, Thailand; ^3^Program in Biotechnology, Faculty of Science, Chulalongkorn University, Bangkok, Thailand; ^4^Structural and Computational Biology Research Group, Department of Biochemistry, Faculty of Science, Chulalongkorn University, Bangkok, Thailand

**Correspondence:** Chonlawat Chaichan - chonlawat.chaichan@gmail.com

*Clinical and Translational Allergy* 2018, **8(Suppl 3)**:P21

**Background**

Carbamazepine (CBZ) is recommended to determine HLA-B*1502 before starting CBZ in Asian ancestry to prevent Stevens–Johnson syndrome and toxic epidermal necrolysis (SJS and TEN). The mechanism of HLA-B*1502 and CBZ-induced SJS/TEN believed that CBZ directly spontaneously interact to HLA-B*1502 explained by indirect pharmacological concept (p-i concept). The p-i concept was briefly explained by laying CBZ on antigen binding groove of HLA complex with non-covalent binding meaning no need intracellular process. In update, its hypothesis is unclear to explain the mechanism. Molecular dynamics simulation is the one of the best tools for unclear phenomenon explanation how carbamazepine induced to HLA-B*1502 and endogenous nonapeptide. Our objective designed to study about carbamazepine interaction between HLA-B*1502 and endogenous nonapeptide (FLFDGSPTY).

**Methods**

The *HLA*-B*1502 modeling complex was set as 310 K body temperature and encompassed with TIP3P water. It was calculated by MM/PBSA and MM/GBSA methods. We generated five structural complexes; HLA/nonapeptide, HLA/CBZ, nonapeptide/CBZ, HLA + nonapeptide/CBZ, HLA/nonapeptide + CBZ for normal mode analysis by Amber16.

**Results**

HLA interacted to nonapeptide (HLA/nonapeptide) which natural structure simulation in the body showed the ∆G binding free energy value about − 17.98 ± 10.68, − 22.11 ± 9.77 kcal/mol in MM/PBSA and MM/GBSA, respectively. In addition, HLA + nonapeptide interacted to carbamazepine (HLA + nonapeptide/CBZ) showed lower ∆G value than HLA/nonapeptide (− 40.20 ± 9.91 kcal/mol in MM/PBSA, − 24.49 ± 11.14 kcal/mol in MM/GBSA). In

**Table 1:** The binding free energy value of five structural generated complexes calculating by MM/PBSA and MM/GBSA methods (kcal/mol **± **SD)

	Nonapeptide/CBZ	HLA/CBZ	HLA/nonapeptide	HLA + nonapeptide/CBZ	HLA/nonapeptide + CBZ
MM/PBSA	2.42 ± 2.65	1.08 ± 9.27	− 17.98 ± 10.68	− 24.49 ± 11.14	− 4.81 ± 7.25
MM/GBSA	3.62 ± 2.60	0.43 ± 9.25	− 22.11 ± 9.77	− 40.20 ± 9.91	− 15.12 ± 6.90

CBZ: carbamazepine, nonapeptide: FLFDGSPTY, HLA: *HLA*-B*1502 mutated from PDB code 1XR9

contrast, other complexes such as HLA interacted to carbamazepine (HLA/CBZ), nonapeptide interacted to carbamazepine (nonapeptide/CBZ) and HLA interacted to nonapeptide + carbamazepine (HLA/nonapeptide + CBZ) were higher ∆G binding free energy value, and it may imply that these complexes may not spontaneous occurrence in this environment (Table 1).

**Conclusion**

Carbamazepine was well done on HLA/nonapeptide complex (HLA/nonapeptide + CBZ) than any other complexes by in silico model analysis not only natural spontaneous complex (HLA/nonapeptide) but also other generated complex (HLA/nonapeptide + CBZ, HLA/nonapeptide + CBZ, HLA/CBZ, nonapeptide + CBZ). This is the first designated separated model to study and analyze on *HLA*-B*1502 and CBZ-induced SJS&TEN.

#### P22 The function of HLA-B*13:01 involved in the pathomechanism of dapsone-induced severe cutaneous adverse reactions

##### Chuang-Wei Wang

###### Department of Dermatology, Linkou, Taiwan

**Correspondence:** Chuang-Wei Wang - kiruamairo@gmail.com

*Clinical and Translational Allergy* 2018, **8(Suppl 3)**:P22

**Background**

Dapsone-induced hypersensitivity reactions may cause severe cutaneous adverse reactions (SCAR), such as drug reaction with eosinophilia and systemic symptoms (DRESS). It has been reported that *HLA*-B*13:01 is strongly associated with dapsone-induced hypersensitivity reactions among leprosy patients. However, the phenotype specificity and detailed immune mechanism of HLA-B*13:01 remains unclear. We investigated the genetic predisposition, HLA-B*13:01 function, and cytotoxic T cells (CTLs) involved in pathogenesis of dapsone-SCAR.

**Methods**

We enrolled patients with DRESS and maculopapular eruption (MPE) with chronic inflammatory dermatoses, from Taiwan and Malaysia.

**Results**

Our results revealed that *HLA*-B*13:01 allele was present in 85.7% (6/7) of patients with dapsone-DRESS (OR = 49.64; 95% CI = 5.89–418.13; *pc *= 2.92 × 10^−4^), but in only 10.8% (73/677) of general population controls in Taiwan. Granulysin level, the SCAR specific cytotoxic protein released from CTLs, was increased in both DRESS patients’ plasma (36.14 ± 9.02 ng/ml, *p *< 0.05) and in vitro lymphocyte activation test (71.4%, 5 in 7 cases) compared to healthy controls (Fig. 1). Furthermore, dapsone-specific CTLs were significantly activated when co-cultured with HLA-B*13:01-expressing antigen presenting cells in the presence of dapsone (3.9-fold increase, compared to cells with no HLA-B*13:01 expression; *p *< 0.01).

**Fig. 1:**
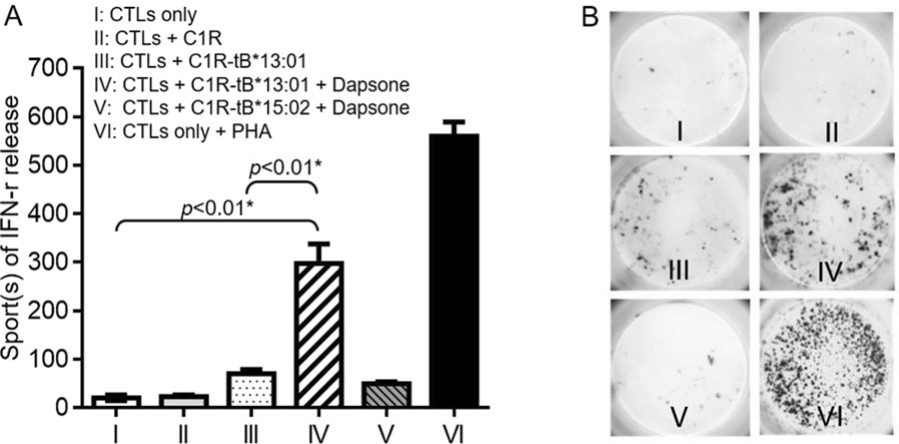
Results

**Conclusion**

This study demonstrates HLA-B*13:01 is strongly associated with dapsone-DRESS and provides a functional role of HLA-restricted immune mechanism induced by dapsone.

#### P23 Should a long delay always be considered as a DRESS criterion?

##### Angèle Soria^1^, Claire Bernier^2^, Gwenaelle Veyrac^3^, Annick Barbaud^4^, Brigitte Milpied^5^

###### ^1^Dermatology and Allergy Department Tenon Hospital APHP and CIMI-Paris INSERM 1135, Paris, France; ^2^Dermatology and Allergy Department Nantes CHU, Nantes, France; ^3^Pharmacology Department Nantes CHU, Nantes, France; ^4^Dermatology-Allergology department, Tenon hospital, APHP; Sorbonne University Paris 06, Paris, France; ^5^Dermatology and Allergy Department Tenon Hospital APHP, Bordeaux, France

**Correspondence:** Annick Barbaud - annick.barbaud@aphp.fr

*Clinical and Translational Allergy* 2018, **8(Suppl 3)**:P23

**Background**

DRESS (Drug Reaction with Eosinophilia and Systemic Symptoms) potentially, associates a widespread skin involvement, hyperthermia, polyadenopathies and at least one visceral involvement, biological abnormalities may exist (eosinophilia, hyperbasophilic lymphocytes). A DRESS diagnosis validation score was established by the RegiSCAR group (Kardaun et al. 2007). It is conventionally accepted that the delay in occurrence of DRESS is between 2 and 8 weeks after the introduction of the culprit drug. This parameter is often used as a diagnostic criterion, although not included in the RegiSCAR score (Kardaun et al. 2007, 2013). Some people reject the diagnosis of DRESS when the time of onset after drug intake is less than 21 days.

**Methods**

All patients with a 1st occurrence of DRESS hospitalized/explored in 3 French dermatology departments, for which a drug was highly suspected (positive patch tests and/or single and/or highly attributable drug) were analyzed retrospectively. The delays of occurrence of DRESS (index day) after the introduction of the culprit drug were classified in short time group ≤ 21 days or long-time group > 21 days.

**Results**

75 patients with DRESS were included; 48 in short-time group and 27 in long-time group. In the short time group, antibiotics (n = 23; of which 18 betalactams) and iodinated contrast media (n = 7) (with often very short delay) were predominant. Carbamazepine (n = 8), lamotrigine (n = 6), allopurinol (n = 2) and salazopyrin (n = 2) were found in the long-time group (Table 1).

**Table 1:** Results

Short-time group (≤ 21 days) n = 48	Long-time group (> 21 days) n = 27
Amoxicillin: n = 10	Carbamazepine: n = 8
Iodinated contrast media : n = 7	Salazopyrin: n = 2
Lamotrigine: n = 4	Lamotrigine: n = 6
Ceftriaxone: n = 3	Allopurinol: n = 2
Olanzapine: n = 2	Rifampicine: n = 1
Terbinafine: n = 2	Adiazine: n = 1
Isoniazide: n = 2	Biperidene: n = 1
Atovaquone: n = 2	Vancomycin: n = 1
Bactrim: n = 1	Bactrim: n = 1
Tazocillin: n = 1	Tazocillin: n = 1
Fluindione: n = 1	Fluindione: n = 1
Cefuroxime: n = 1	Raltegravir : n = 1
Cefpodoxime: n = 1	Fluconazole: n = 1
Ceftazidime: n = 1	
Meronem: n = 1	
Dalacine: n = 1	
Rovamycine: n = 1	
Atazanavir : n = 1	
Etravirine: n = 1	
Emtricitabine/tenofovir: n = 1	
Abacavir: n = 1	
Valaciclovir: n = 1	
Miconazole: n = 1	
Anafranil: n = 1	

**Conclusion**

It appears to be a link between the delay of onset and the class of drugs involved. This could be related to different pathomechanisms; “Short-time group” DRESS would be related to a challenge in previously sensitized patients and long-time group to active sensitizations during the 1st treatment. Complementary, prospective studies are needed; however, it seems important not to reject the diagnosis of DRESS when the delay of onset is short if the other criteria are present.

Kardaun, S. H., et al. 2013. *Br J Dermatol*

Kardaun, S. H., et al. 2007. *Br J Dermatol*

#### P24 The German Registry of Stevens–Johnson syndrome/toxic epidermal necrolysis (SJS/TEN): results of 10 years (2003–2012)

##### Maren Paulmann^1^, David Nägele^1^, Peggy Sekula^2^, Maja Mockenhaupt^1^

###### ^1^Dokumentationszentrum schwerer Hautreaktionen, Department of Dermatology, Medical Center and Medical Faculty, University of Freiburg, Freiburg, Germany; ^2^Institute of Genetic Epidemiology, Department for Biometry, Epidemiology and Medical Bioinformatics, Medical Center and Medical Faculty, University of Freiburg, Freiburg, Germany

**Correspondence:** Maren Paulmann - maren.paulmann@uniklinik-freiburg.de

*Clinical and Translational Allergy* 2018, **8(Suppl 3)**:P24

**Background**

Severe mucocutaneous reactions like SJS/TEN are rare, but life-threatening. These reactions are often induced by drug intake. The severity is determined by the amount of skin detachment of body surface area (SJS < 10%, SJS/TEN-overlap 10–30%, TEN > 30%). Since its foundation in 1990 the German Registry (dZh) actively collects patients with SJS/TEN to monitor changes in epidemiology and pharmacovigilance.

**Methods**

Patients, who were registered between 2003 and 2012 with a validated diagnosis of SJS/TEN, were included for analysis of epidemiological data and incidence rates based on population data (provided by Federal Institute of Statistics).

**Results**

From 2003 to 2012 the dZh registered 760 patients with a validated diagnosis of SJS/TEN. 400 cases (53%) were classified as SJS, 252 cases (33%) as SJS/TEN-overlap and 108 cases (14%) as TEN. 229 patients (30%) developed the reaction in hospital, whereas 531 cases (70%) were community acquired. The median age was 63.5 years (1–98) with similar distribution for SJS (65 years (1–97)) and SJS/TEN-overlap (64.5 years (2–98)), but patients with TEN were substantially younger (51 years (2–89)). Women are more often affected (61%). In total, 213 patients died with increasing mortality rate related to severity (SJS: 14%, SJS/TEN-overlap: 43%, TEN: 48%).

The estimated incidence was 0.93 per million inhabitants per year (95% confidence interval (CI) 0.86–0.99). The incidence decreases with the severity of disease: SJS 0.49 (95% CI 0.44–0.54), SJS/TEN-overlap 0.31 (96% CI 0.27–0.35), TEN 0.13 (95% CI 0.11–0.16). The incidence is higher for women 1.11 (95% 1.01–1.22) compared to men 0.74 (95% CI 0.66–0.83) and increases for SJS and SJS/TEN-overlap with increasing age, but remains similar for TEN over all age groups.

About 50% of the cases can be explained by a drug with a high risk for SJS/TEN, e.g. allopurinol, sulfamethoxazole and lamotrigine, and approximately 20% of the cases by drugs with a moderate risk.

**Conclusion**

Unknown changes in drug risks for SJS/TEN can be a major problem in public health. Therefore, an extensive ascertainment of cases is necessary. The stable overall incidence indicates that the coverage of the German Registry is very high and no larger proportion of cases is missed. These data collected over a period of 10 years allow the conclusion that for some high risk drugs nothing has changed in drug risks for SJS/TEN despite previous warnings. Nevertheless, due to the introduction of new drugs into the market a continuous surveillance and ongoing case registration remains important—not only in Germany.

#### P25 Drug reaction with eosinophilia and systemic symptoms due to spironolactone: case report

##### Rosa-Anita Fernandes, Frederico Regateiro, Emília Faria, Margarida Gonçalo, Ana Todo-Bom

###### Centro Hospitalar e Universitário de Coimbra, Coimbra, Portugal

**Correspondence:** Rosa-Anita Fernandes - rosa.anita.fernandes@gmail.com

*Clinical and Translational Allergy* 2018, **8(Suppl 3)**:P25

**Background**

Spironolactone, an aldosterone antagonist with antiandrogenic activities, is often used in combination with other medicines to treat hypertension and heart failure. Hypersensitivity reactions to spironolactone are very rare and often mild.

**Case report**

79-year-old male with a history of tophaceous gout, hypertension and heart failure who developed a generalized pruritic exanthema after 10 days of treatment with amlodipine, spironolactone and allopurinol. He was immediately advised to stop these drugs but skin eruption did not resolve and, during the following 6 days, pruritus worsened and he developed facial oedema and erythema, sore throat and fever (39 °C), leucocytosis (20.8 × 10^9^/L), hypereosinophilia (40.9%, 8500 eosinophils/mm^3^), ALT 84U/L, AST 69U/L, ALK 261U/L and GGT 204U/L, therefore fulfilling the RegiSCAR criteria of probable DRESS. He was admitted to a medical ward, all remaining medications were halted, oral prednisone 1 mg/kg/day was started. Symptoms and laboratory alterations improved gradually and the rash resolved within 3 days.

On discharge, life-long eviction of allopurinol, amlodipine and spironolactone was recommended.

Seven years later, he was referred to our Drug Allergy consultation for the investigation of drug hypersensitivity and for possible desensitization to spironolactone needed for exuberant tophaceous gout. Taking into consideration that allopurinol is one of the most frequent causes of DRESS and only two published case reports associated DRESS with spironolactone, we performed a diagnostic drug provocation test (DPT) with oral spironolactone to exclude hypersensitivity to this medication. The patient tolerated 10% (9 g) of the dose without symptoms but on the second day, within 6 h of the second dose (28.5 mg) (cumulative dose of 37.5 mg), the patient developed generalized skin erythema and pruritus, no fever, and hypereosinophilia (30%, 4 × 10^9^/L) with no other laboratory alterations. A bolus of 125 mg methylprednisolone was administered, with complete resolution of symptoms after 12 h. Six weeks after DPT, we performed patch tests with spironolactone and allopurinol prepared in saline and petrolatum in 3 concentrations each (1%, 10%, 20%). A strong positive reaction to spironolactone was observed at 48 h in all tested concentrations (0.25 mg/mL, 2.5 mg/mL, 5 mg/mL) but no reaction to allopurinol at any concentration.

**Conclusion**

Spironolactone has rarely been associated with DRESS or indeed other delayed HS reactions. In this patient, the positive non-immediate DPT with drug rash and hypereosinophilia, and the positive patch tests support the diagnosis of DRESS to spironolactone. The positivity of the patch tests opens the way for the development of standardized methodologies to study delayed allergic reactions.

**Consent to publish**

I confirm that I have received written consent for online publication from all patients described (or their parents/guardians), where there are images, videos, or 3 more indirect identifiers to be published.

#### P26 Bullous pemphigoid associated with dipeptidyl-peptidase IV inhibitors intake: myth or reality?

##### Florence Tetart^1^, Marthe Plaquevent^2^, Laurence Fardet^3^, Jacques Benichou^2^, Pascal Joly^1^

###### ^1^Dermatology Department, Rouen, France; ^2^Rouen University Hospital, Rouen, France; ^3^Dermatology Department, Créteil, France

**Correspondence:** Florence Tetart - florence.tetart@chu-rouen.fr

*Clinical and Translational Allergy* 2018, **8(Suppl 3)**:P26

**Background**

Dipeptidyl-peptidase IV inhibitors (DPPIV-I) are oral anti-diabetic drugs used in the treatment of adult type II diabetes mellitus. Bullous pemphigoid (BP) is an auto-immune blistering skin disease which mainly occurs in the elderly. It is characterized by the production of anti BPAG1 and anti-BPAG2 antibodies leading to the formation of sub epidermal blisters. A link between DPPIV-I and BP has been recently suggested by a pharmacovigilance study and some case reports.

**Methods**

The main objective of this multicentre study (collaborative work between “FISARD” and “Bulles” workgroups of the French Society of Dermatology) was to compare the frequency of DPPIV-I intake in a population of BP patients referred between 2012 and 2015, relative to the age-adjusted general population using data from the national health insurance data base during the same period. Secondary objective was to describe the clinical characteristics and course of DPPIV-I-associated BP (delay of BP control, rate of relapse of BP after DPPIV-I withdrawal).

**Results**

Out of 1787 BP patients, 108 [58 males; 50 females] (6%) of mean age 78 years were treated by DPPIV-I, relative to 3.8% in the aged-adjusted general population (indirect standardization) (p < 0.001). The proportions of types of DPPIV-I intake in the BP and aged-adjusted general populations were 2.4% versus 2.5%, for sitagliptine, 3.3% versus 0.8% for vildagliptine (p < 0.001) and 0.1% versus 0.3% for saxaglitine, respectively. Median delay between gliptin introduction and the occurrence of BP was 15 months [interquartile range (IQR): 6–27 months]. Gliptins were stopped in 48 cases (44%), corresponding to an early withdrawal (delay shorter than 1 month) in 29 cases (27%), or after a longer delay (> 1 month) in 19 cases (18%). Median delay of BP control was 15 days when DPPIV-I were stopped versus 14 days when they were continued (p = 0.32). However, this delay was shorter, 8 days in patients with an early DPPIV-I withdrawal than in those with a late withdrawal 41 days (p < 0.05). The relapse rate was not different whatever DPPIV-I were stopped or not (42% versus 37%, p = 0.81). Rechallenge was not performed.

**Conclusion**

DPPIV-I are more frequently prescribed in BP than in the age-related general population. Early DPPIV-I withdrawal might shorten the delay to BP control.

#### P28 DRESS are not virus-related diseases in the majority of cases

##### Benoit Ben Said^1^, Jean Francois Nicolas^2^, Denis Jullien^1^

###### ^1^SCARS National Reference Center, Dermatology, GH Lyon Centre, Lyon, France; ^2^Allergology and Clinical Immunology Department, GH Lyon Sud, Lyon, France

**Correspondence:** Benoit Ben Said - bensaidprof@gmail.com

*Clinical and Translational Allergy* 2018, **8(Suppl 3)**:P28

**Background**

DRESS is one of the most severe SCARS associated with a mortality of 5–10%. During the last decade the role herpes virus reactivation (HHV6-7-cmv-ebv) in the development of DRESS was discussed. In this work we have evaluated in common practice the frequency of viral reactivation during DRESS syndrome detected by whole blood PCR.

**Methods**

Between 01/2012 and 01/2015 all patients seen for DRESS confirmed by KARDAUN score > 5 were evaluated for reactivation of the HERPES group viruses (HHV6, EBV, CMV) by blood PCR evaluated at diagnosis (D0) and repeatedly later (D7–D42) and in the resolution phase (> 6 months) when possible.

**Results**

During the study 85 DRESS patients were included. Among them 12% had a positive viral PCR at diagnosis (D0). Of these 85 cases, 55 cases were evaluated for these viruses several times. Of these 55 cases, 16% had remote reactivation (D7–D42) bringing the total viruses reactivation to 28% in acute phase. These late reactivations were dominated by CMV (80%) and HHV6 (20%). Of these 85 cases 65 were evaluated for these viruses in the resolution phase with a reactivation rate of 20% and blood levels remains identical to the initial level in all cases. In the resolution phase, the skin tests with the attributable drugs (score > I3) (Patch tests) found a positivity of patch tests in 60% of cases.

Discussion: In contrast of previous, we find very rare viral reactivations during DRESS.

These data do not mean that the virus can not play a role in DRESS but suggests that virus are not the initial event or the cause of the disease. Viruses such as CMV seems to play a prognosis role in DRESS during evolution as has already been observed in dysimmune diseases such as GVH. Among the 65 cases evaluated, the skin tests were found to be positive in 60% of cases, confirming the role of drug delayed hypersensitivity during DRESS.

**Conclusion**

The role of virus in the pathophysiology of DRESS is discussed. Our study confirms that viral reactivations detected, according to our method of detection and the virus searched, are positive in a minority of DRESS and that these viruses are not in our study, the original mechanism of the disease.

#### P29 Epidermal necrolysis-associated mortality in a predominantly HIV-infected population exclusively managed with supportive care. A series of 184 consecutive cases at a single centre

##### Rannakoe Joseph Lehloenya, Niita Hiutembu, Wisdom Basera, Jonny Peter

###### University of Cape Town, Cape Town, South Africa

**Correspondence:** Jonny Peter - jonny.peter@uct.ac.za

*Clinical and Translational Allergy* 2018, **8(Suppl 3)**:P29

**Background**

Epidermal necrolysis (EN)-associated mortality rate in a population determines the sample size required to show benefit, deleterious effects or the number needed to treat to save one life for any intervention. In rare diseases like EN, a small variation in mortality can have a major impact on the study outcome. There has recently been a concerted global effort to identify interventions that improve mortality in EN. However, there are limited mortality data when care of EN is exclusively supportive. This baseline data is needed to assess benefits of any intervention.

Human immunodeficiency virus (HIV) infection is associated with higher incidences of EN and HIV-infected persons are likely to form a significant portion of the global cohort that assesses an intervention. Thus, good quality baseline mortality data in HIV-infected persons with EN is necessary.

We suspected lower than expected mortality in our predominantly HIV-infected cohort exclusively managed with supportive care in a South African tertiary hospital.

**Methods**

To confirm this, we retrospectively reviewed all cases of epidermal necrolysis managed by the dermatology service at a tertiary hospital in Cape Town, South Africa between January 2004 to December 2014. Our management of epidermal necrolysis throughout the study period was exclusively supportive. Patient management included oral fluid resuscitation, enteral nutritional support, daily baths with antiseptic solution and sterile non-adherent dressings. Intravenous lines were only used when intravenous antibiotics or urgent resuscitation were necessary and indwelling catheters were generally avoided. No prophylactic antibiotics, systemic steroids, intravenous immunoglobulin, cyclosporine or other specific therapeutic medications were administered to any of the patients. Anticoagulation was only administered to patients who were completely bed-bound for more than 7 days.

**Results**

184 cases of EN were seen during the study period. 41% were > 40 years old; 70% were females; 78% were HIV-infected with a median (IQR) CD4 count of 185 cells/mm^3^ (97–264); 12% were pregnant; 17% had renal impairment; 7% had urea ≥ 10 mmol/l; none had a malignancy; 56% has Stevens Johnson syndrome (SJS); 27% had toxic epidermal necrolysis (TEN); 16% had SJS/TEN overlap; 6(3.3%) died in hospital before discharge.

Using available parameters to calculate SCORTEN (age, recent malignancy, heart rate, serum urea, serum glucose and affected surface area) our expected mortality was 6.9% vs. 3.3% actual mortality.

**Conclusion**

Mortality in this predominantly HIV-infected population was significantly lower than that predicted by SCORTEN. These data inform design of global studies assessing interventions in EN.

#### P30 Stevens–Johnson syndrome and toxic epidermal necrolysis associated with NSAIDs intake: A case series

##### Anastasiia Allenova, Anfisa Lepekhova, Olga Olisova, Natalya Teplyuk, Natalia Pereverzina, Pavel Kolkhir

###### I.M. Sechenov First Moscow State Medical University, Division of Immune-mediated Skin Diseases, Department of Dermatology and Venereology, Moscow, Russia

**Correspondence:** Natalia Pereverzina - natalia.pereverzina@gmail.com

*Clinical and Translational Allergy* 2018, **8(Suppl 3)**:P30

**Background**

Stevens–Johnson syndrome/toxic epidermal necrolysis (SJS/TEN) is a life-threatening, immunologically mediated, and usually drug-induced disease. It is characterized by an epidermal blistering rash with necrosis, desquamation and mucosal surface involvement. Anti-infective, anti-epileptic, chemotherapy and non-steroidal anti-inflammatory (NSAIDs) drugs are the most common causative drugs.

**Methods**

We report a case series of six adult patients with NSAIDs-associated SJS/TEN. The diagnosis SJS/TEN was confirmed by histopathology and direct immunofluorescence. All patients were treated in the burn centers in accordance with clinical guidelines. SCORTEN score was used to assess severity and predict mortality.

**Results**

Patients with NSAIDs-associated SJS/TEN comprised 46% (n = 6) of 13 patients with SJS/TEN who attended our Department over a 9-year period. Five out of six patients were females aged 36 or older (mean: 61 years). All patients had disseminated blisters and erosions of the skin and mucous membranes. The lesions developed 5–28 days after administration of NSAIDs. We observed electrolyte imbalance, bronchial obstruction, kidney injury and intestinal failure in six, two, two and three patients, respectively. Two patients (33%, SCORTEN 3–4 points) died due to severe reactions caused by piroxicam or ketoprofen. Naproxen, indomethacin and celecoxib caused milder reaction (SCORTEN 2 points).

**Conclusion**

SJS/TEN causes high mortality. Although the risk of SJS/TEN in patients receiving NSAIDs is considered to be extremely low, practitioners and patients need to be aware of the initial clinical signs of such severe drug reactions. The management of the disease is based on immediate withdrawal of potentially causative drug and prompt referral to an appropriate medical center for specific supportive treatment.

## Thursday 19 April 2018

### Skin reactions in drug allergy - Poster Walk 4

#### P31 Bullous Pemphigoid-Like during treatment with rivaroxaban: a case report

##### Cristiana Sofia Ferreira, Maria Arminda Guilherme, Natividade Rocha, Ana Cristina Oliveira, Jose Almeida Ribeiro, Antonia Furtado

###### CHVNG/E, Gaia, Portugal

**Correspondence:** Cristiana Sofia Ferreira - cristianascferreira@gmail.com

*Clinical and Translational Allergy* 2018, **8(Suppl 3)**:P31

**Background**

Rivaroxaban is a new oral anticoagulant drug, acting as an direct factor Xa inhibitor and is used in venous thromboembolism (VTE) prophylaxis. This drug is increasingly prescribed because is an oral drug and no coagulation monitoring is needed. Rare cases of hipersensibility reactions has been described.

**Case report**

We report the case of a 76-year-old female patient with a history of paroxismal atrial fibrillation and depression, who developed a rash characterized by urticarial plaque on her back, upper limbs, and anterior surfaces of her thighs and also sero-haemorrhagic tense blisters on both legs, 15 days after starting treatment with sertraline, rivaroxaban and furosemide. Given the known association between furosemide and bullous pemphigoid, it was discontinued and started topical corticosteroid application. Laboratory study showed WBC of 7220 with 14.4% eosinophils, platelets 197,000/μl, CEA, Ca 15.3, Ca 19.9, and Ca 125 results in normal ranges. The search for anti-skin, anti-basement, epidermal membrane and anti-keratinocyte autoantibodies was negative. Histological examination disclosed intraepidermal blistering with eosinophils and apoptotic keratinocytes inside, with significant spongiosis in the neighboring epidermis. Discrete infiltrates of eosinophils were diffusely represented among spongiotic areas and throughout the upper part of the dermis in a perivascular and interstitial infiltrate pattern. Immunofluorescence was negative for IgA, IgG, IgM, C3, and C1. Two weeks after the use of furosemide had been discontinued, the cutaneous condition worsened; bullous lesions appeared on the forearms. At that time, rivaroxaban was discontinued and replaced with warfarin; furosemide was reintroduced. A rapid improvement in the lesions was noted within a week, and no relapse occurred during the 12-month follow-up. Histology was consistent with bullous pemphigoid. Negative immunofluorescence studies, as in this case, occur in 4% of the patients.

**Conclusion**

The diagnosis of bullous pemphigoid-like skin eruption to rivaroxaban was established. To the best of the authors’ knowledge, this is the first case reported in the literature.

**Consent to publish**

Consent to publish was obtained from the patient involved in this study.

#### P32 Drug reaction with eosinophilia and systemic symptoms syndrome induced by three drugs: a case report

##### Cristiana Ferreira, Arminda Guilherme, Miguel Vieira, Patricia Barreira, Ines Lopes

###### CHVNG/E, Gaia, Portugal

**Correspondence:** Cristiana Ferreira - cristianascferreira@gmail.com

*Clinical and Translational Allergy* 2018, **8(Suppl 3)**:P32

**Background**

Drug reaction with eosinophilia and systemic symptoms (DRESS) syndrome is a severe potentially life-threatening drug hypersensitivity reaction, most frequently associated with antiepileptic drugs, usually characterized by skin rash, fever, lymphadenopathy, eosinophilia, and visceral organ involvement, typically presenting within 8 weeks of initiation of therapy. We present a case of DRESS induced by the consecutive use of valproic acid, levofloxacin e levetiracetam.

**Case report**

We report a case of DRESS by the consecutive use of three drugs in which the diagnosis was difficult to establish because any drug intake could induce this severe reaction. The allergy work-out should include all involved drugs.

**Fig. 1:**
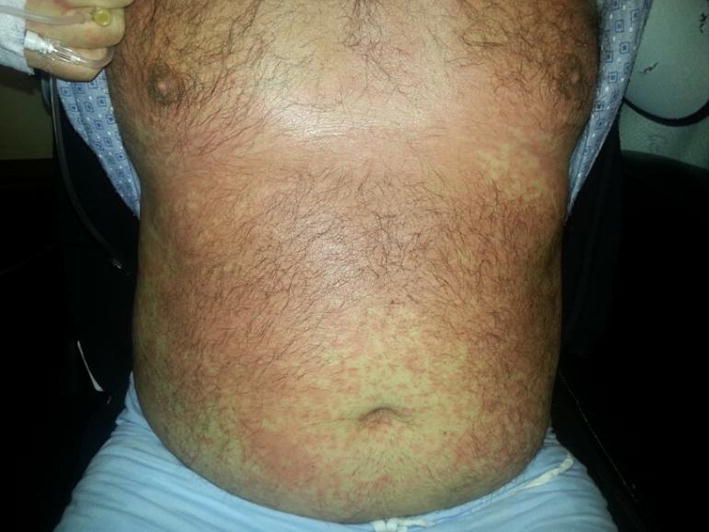
Case report

LTT has been useful to identify the etiological agent (Fig. 1).

**Conclusion**

A 44-year-old male presented to our emergency department with acute pulmonary edema and during hospital stay developed a generalized tonic–clonic seizure and respiratory infection. Twenty and fifteen days after starting treatment with valproic acid and levofloxacin respectively, he presented a generalized maculopapular rash with erythema. After withdrawal of both drugs his rash improved. In view of the history of seizure, patient was switched to levetiracetam and 2 weeks later, he presented a progressively worsening of the generalized erythematous maculopapular rash (trunk, abdomen, upper and lower extremities) and fever. Laboratory findings revealed eosinophilia (1020/µl), and elevated serum transaminases: AST 162 U/l, ALT 196 U/l. Based on the European Registry of Severe Cutaneous Adverse Reactions (RegiSCAR) criteria, a probable diagnosis of DRESS was made. At that time, levetiracetam was switched to clonazepam and zonisamide and oral corticoid treatment was established. After discharge the patient was seizure free, transaminitis has resolved and the rash has subsided. He was evaluated on Allergy Unit, lymphocyte transformation test (LTT) was positive to valproic acid and levetiracetam and doubtful to levofloxacin.

**Consent to publish**

Consent to publish was obtained from the patient involved in this study.

#### P33 Netherton syndrome plus amoxicillin SCAR: how to crack the case

##### Carneiro-Leão Leonor, José Luís Plácido, Josefina Cernadas

###### ^1^Serviço de Imunoalergologia, Centro Hospitalar de São João, Porto, Portugal

**Correspondence:** Carneiro-Leão Leonor - leonorcarneiroleao@gmail.com

*Clinical and Translational Allergy* 2018, **8(Suppl 3)**:P33

**Background**

Netherton syndrome is a rare inherited autosomal recessive disorder that characteristically presents with ichthyosiform erythroderma, “bamboo hair” (trichorrhexis invaginata) and atopic diathesis. Skin symptoms can be very significant and are commonly treated with topical steroids.

**Case report**

A 27-year-old female patient with Netherton syndrome was referred to our Drug Allergy Unit for suspected amoxicillin allergy. She reported a history of a new vesicular skin rash, with generalized confluent vesicles and desquamation, targetoid lesions, pruritus, skin pain and malaise, starting in the 5th day of amoxicillin treatment for urinary tract infection. Her dermatologist prescribed oral steroids (prednisolone, 1 mg per kg for 2 weeks), with rash resolution with skin desquamation over 2 weeks.

Although the patient presented photographs of this reactions, the interpretation of skin lesions was impaired due to Netherton syndrome related skin changes. However, the history was strongly suggestive of a severe cutaneous adverse drug reaction (SCADR) to amoxicillin.

Due to chronic atrophy related to chronic topical steroid use, intradermal skin tests with delayed readings or patch tests were unfeasible. In these circumstances, we decided to perform a lymphocyte transformation test (LTT), not only to confirm amoxicillin as the culprit drug but also to assess the safety of alternative beta-lactam antibiotics. LTT was positive to amoxicillin (stimulation index [SI] = 6.9) and negative to cefixime (SI < 2).

**Conclusion**

Netherton syndrome is a rare skin disease, with major skin changes. In this case, not only the skin symptoms related to the suspected SCADR episode were difficult to evaluate but also the in vivo allergologic diagnostic work-up was impaired. This case illustrates the importance of in vitro tests, particularly the LTT in very specific and clear cases, which was determinant to confirm amoxicillin allergy and to help establish a 3rd generation cephalosporin as a most likely safe alternative to this patient.

**Consent to publish**

Consent to publish was obtained from the patient involved in this study.

#### P34 Acute exanthemas: clinical, virologic, histological, cytokine and metagenomic analysis of a prospective series of 98 patients

##### Olivia Deschamps, Nicolas Ortonne, Sophie Hüe, Christophe Rodriguez, Carine Deschodt, Gaëlle Hirsch, Audrey Colin, Marie-Hélène Delfau-Larue, Olivier Chosidow, Pierre Wolkenstein, Saskia Oro

###### Henri Mondor Hospital, Créteil, France

**Correspondence:** Saskia Oro - saskia.oro@aphp.fr

*Clinical and Translational Allergy* 2018, **8(Suppl 3)**:P34

**Background**

Acute exanthemas are frequent but poorly studied. We conducted a prospective study to describe their clinical, virologic, and histological aspects and to investigate their cytokine and metagenomic profile.

**Methods**

We included all acute exanthemas consulting in our emergency department from Feb. to May 2014. Clinical data were recorded in a standardized chart. Virologic investigation included HIV, HBV, HCV, EBV, and parvovirus B19 serologies; PCR EBV, CMV, and HHV6. A skin biopsy was performed. A cytokine analysis of a panel of 16 cytokines with Luminex kit R and D (Minneapolis, USA) was performed in available blood and skin samples. A shotgun metagenomic analysis was performed in samples selected in cytokine analysis.

**Results**

98 patients were included. According clinical aspect, drug anamnesis and virologic investigations, we defined 4 groups (n = 88) with maculopapular exanthemas (MPE) [“viral” (n = 18), drug-induced (n = 33), drug and virus (n = 5), and “idiopathic” MPE (n = 32)], and 1 with aspect of pityriasis rosea (n = 10). In total, a virus was identified in 29 cases (HHV6 74%).

Among the 92 biopsies, 24 showed a pure dermal infiltrate, 53 an epidermal infiltrate (eczematous and/or lichenoid pattern), and 5 another aspect. A pure dermal infiltrate was more frequent in viral MPE than drug-induced and idiopathic MPE (57% vs 33% and 25%, p = 0.13). Eosinophil rate was not discriminant (virus 41%, drug-induced 48%, idiopathic 37%, p = 0.67).

Cytokine analysis was performed in 23 MPE cases [viral (n = 8), drug-induced (n = 7) and idiopathic (n = 8) MPE]. Medians of cytokine concentrations in the skin are shown in the Table 1. As expected, IFNγ and IL1Rα rates in the skin were higher in viral MPE. Interestingly, idiopathic MPE displayed higher IL10, granzyme A and IL33 values. Blood cytokine concentrations were very low.

**Table 1:**
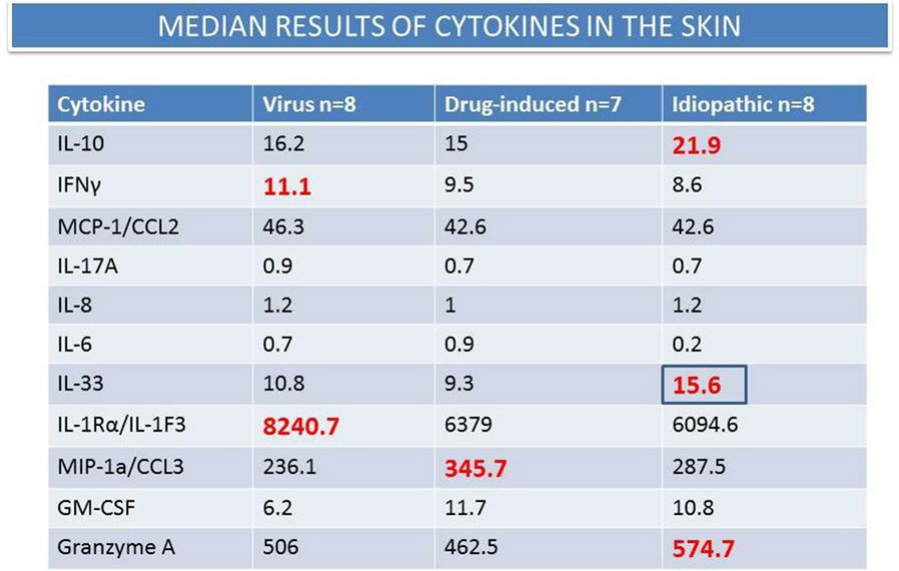
Results

Metagenomic analysis was performed in 10 skin biopsies. In the viral group (n = 4), the virus identified by routine techniques was not found. We showed Enterovirus A in 2 cases (1 from viral and 1 from drug MPE) and *P. aeruginosa* in 1 case (idiopathic MPE).

**Conclusion**

Routine investigations found HHV6 as the most frequent virus. Histology did not discriminate viral and drug-induced MPE. In idiopathic MPE (1/3 of cases), cytokines analysis in the skin found higher rates of IL-33 and metagenomic analyses did not identified any pathogen in 3/4 cases suggesting environmental factors as trigger. However, clinical examination, drug anamnesis and minimal viral investigation (including HIV serology) remain to date the key points of the management of exanthemas in routine practice.

#### P35 Drug reaction with eosinophilia and systemic symptoms (DRESS) in Singapore: clinical features, treatment and outcomes of 100 patients managed in an academic medical centre

##### Chong En Linus Chan^1^, Jui Lin Karen Choo^2^, Yi Wei Yeo^2^, Shiu Ming Pang^2^, Haur Yueh Lee^2^

###### ^1^Duke-NUS Medical School, Singapore, Singapore; ^2^Department of Dermatology, Singapore General Hospital, Singapore, Singapore

**Correspondence:** Chong En Linus Chan - linus.chan@u.duke.nus.edu

*Clinical and Translational Allergy* 2018, **8(Suppl 3)**:P35

**Background**

Drug reaction with eosinophilia and systemic symptoms (DRESS), also termed drug-induced hypersensitivity syndrome (DIHS), is a life-threatening and rare severe cutaneous adverse drug reaction. Its clinical course is variable, ranging from benign and self-resolving, to severe and life-threatening with multi-organ failures and even death. This study aimed to evaluate the clinical characteristics, course, response to therapy, and outcomes of DRESS.

**Methods**

Patients admitted to the Department of Dermatology, Singapore General Hospital, from January 2009 to December 2017 were retrospectively reviewed. Cases which fulfilled the probable or definite case criteria based on the European Registry of Severe Cutaneous Adverse Reactions (RegiSCAR) were included for analysis. Clinical features, demographic data, treatment choices and outcomes were retrieved from medical records and analysed.

**Results**

Of the 100 patients, 41 were men (41%) and 59 were women (59%) with an age range of 17–86 years (mean, 57 ± 17.62 years). The most commonly implicated drugs were allopurinol (40%), sulfonamides (23%), and anti-epileptic drugs (9%). Facial edema (90%), confluent erythema (73%), and maculopapular exanthema (72%) were the most common dermatologic features. Associated systemic features included eosinophilia (88%), atypical lymphocytes (87%), hepatic involvement (73%), and renal involvement (42%). For management, 42 patients (42%) and 54 patients (54%) were treated with topical and systemic steroids respectively. 16% (n = 16) of patients required ICU management, and the in-hospital mortality was 15% (n = 15). Amongst survivors, 26% (n = 26) had flares requiring clinic visits or hospitalisations.

**Conclusion**

Our study validates the findings that the majority of DRESS is related to a small number of medications. Liver, renal and blood involvement were the most common associated findings. There appears to be a higher mortality rate in our cohort compared to prevailing studies and this may be in part explained by the referral bias in an academic centre.

#### P36 Drug Rash with eosinophilia and systemic symptoms (DRESS): a case series

##### Zohra Chadli, Haifa Ben Romdhane, Amel Chaabane, Najeh Ben Fadhl, Nadia Ben Fredj, Naceur A. Boughattas, Karim Aouam

###### Pharmacology Laboratory, Faculty of Medicine, University of Monastir, University Hospital of Monastir, Tunisia, Monastir, Tunisia

**Correspondence:** Zohra Chadli - zohrachadly@ymail.com

*Clinical and Translational Allergy* 2018, **8(Suppl 3)**:P36

**Background**

Drug rash with eosinophilia and systemic symptoms (DRESS) is a severe idiosyncratic adverse drug reaction with multiple-organ involvement. Common clinical manifestations and laboratory findings include fever, cutaneous eruption, facial oedema, lymphadenopathy, eosinophilia, atypical circulating lymphocytes, and liver injury. Other systemic manifestations include pneumonitis, pancreatitis, renal failure, and neurological symptoms. The most common drugs induced DRESS are antiepileptic drugs (AED) (carbamazepine, phenobarbital and phenytoin), allopurinol and antibiotics.

Objective: The aim of this study was to evaluate the epidemiological, clinical and chronological Drug Rash with Eosinophilia and Systemic Symptoms (DRESS) characteristics and to identify the implicated drugs.

**Methods**

We carried out a retrospective study including all cases of DRESS notified to the Pharmacovigilance Unit of Monastir during 13 years (2004–2016). Diagnosis of DRESS was based on European *RegiSCAR* criteria. Imputability was established according to *Begaud’s* method. Skin tests were performed according *ENDA* recommendations.

**Results**

Seventy four cases of DRESS were included in our study: 34 men and 40 women, with a mean age of 49 years ± 20.6 (6–74 years). Antiepileptics drugs (23 cases: 19 carbamazepine, 3 phenobarbital, and 1 lamorigine) were the most frequent responsible drugs in our study followed by antibiotics (20: 12 betalactams, 5 glycopeptides, 1 cotrimoxazole, 1 ethambutol and 1 ciprofloxacin), allopurinol (18), salazopyrin (7) and antihypertensive drugs (3), terbinafine, celecoxib and meglumine antimoniate (1 case each). All patients had pruritic maculopapular rash involving more than 50% of their body surface area. Mucosal involvement was observed in 17 cases (23%). Lymphadenopathy was more frequent with salazopyrin and antiepileptics drugs (55% each). Eosinophilia was observed in 77% of cases. Atypical lymphocytosis was observed only in 9 cases. Liver was the most common organ affected (74.5%) in our series. Renal failure was observed in all cases induced by allopurinol. Pulmonary involvement was observed in 5 cases (3 with salazopyrin and 2 with antiepileptics drugs). The mean incubation period was two weeks. The evolution was favorable after drug withdrawal in 95.7% of cases. Two patients with DRESS induced by allopurinol died because of multiple organ failure. Skin tests (patch or intradermal tests) were done in 50 cases. 58% of skin tests were positive (mainly with antiepileptics drugs and antibiotics). Skin tests with salazopyrin and allopurinol were all negative.

**Conclusion**

Throughout this study, we point out the variability of DRESS clinical characteristics according to the incriminated drug in one hand and the usefulness of skin tests with salazopyrin and allopurinol in the other hand.

#### P37 Comparison among three methods of interferon-gamma enzyme-linked immunospot assay for identifying hypersensitivity-inducing drug Culprits

##### Hiroaki Azukizawa^1^, Asami Kawase^1^, Kenichi Kato^2^, Hideo Asada^1^

###### ^1^Department of Dermatology, Nara Medical University, Kashihara, Japan; ^2^Department of Dermatology, Kinki Central Hospital, Itami, Japan

**Correspondence:** Hiroaki Azukizawa - azukizaw@naramed-u.ac.jp

*Clinical and Translational Allergy* 2018, **8(Suppl 3)**:P37

**Background**

Drug-induced Interferon-gamma (IFN-g) enzyme-linked immunoSpot (ELISpot) is an useful in vitro test for identifying culprit drugs in cutaneous adverse drug reactions (cADR) cases. Recent report suggested that the measurement of oxypurinol-inducing IFN-g-releasing cells yields a high diagnostic value in distinguishing between allopurinol-allergic and control subjects, when anti-programmed death ligand 1(PD-L1) antibodies were added (anti PD-L1 IFN-g ELISpot). More recently, we reported that drug-induced IFN-g ELISpot using Peripheral blood mononuclear cells (PBMCs), which were stimulated with anti-CD3/CD28 antibody-coated microbeads and IL-2 for 7 days before exposure to the culprit drugs (modified IFN-g ELISpot), was more sensitive than the conventional IFN-g ELISpot. The aim of this study was to clarify the utility of conventional IFN-g ELISpot, anti PD-L1 IFN-g ELISpot, and modified IFN-g ELISpot.

**Methods**

Seventeen patients with cADR, caused by clinically convincing culprit drugs, were enrolled in this study. In some cases, the blood samples were obtained at two or three different time points. PBMCs from total 21 samples were analyzed using conventional IFN-g ELISpot, anti PD-L1 IFN-g ELISpot, and modified IFN-g ELISpot.

**Results**

Among the culprit drugs tested in each patient, both conventional IFN-g ELISpot and modified IFN-g ELISpot were positive in six samples, while anti PD-L1 IFN-g ELISpot were positive in four samples.

**Conclusion**

Since enrolled cases were limited, more samples are needed for clarifying the enhancement of drug induced IFN-g production by anti PD-L1 antibody. Combination of modified IFN-g ELISpot and conventional IFN-g ELISpot may increase the sensitivity for detecting IFN-g production by drug culprits.

#### P38 Drug-induced hypersensitivity syndrome caused by hepatitis C treatment with ribavirin and HSV reactivation

##### Simona Kasinskaite, Brigita Sitkauskiene

###### Lithuanian University of Health Siences, Kaunas, Lithuania

**Correspondence:** Simona Kasinskaite - simonaityte@gmail.com

*Clinical and Translational Allergy* 2018, **8(Suppl 3)**:P38

**Background**

Drug induced hypersensitivity syndrome (DIHS) also called drug rash with eosinophilia and systemic symptoms (DRESS) is a severe reaction usually characterized by fever, rash, and multiorgan failure. The pathogenesis of this syndrome is not well understood and it is hypothesized that a complex of interaction between genetic factors and a possible virus-drug interaction may have a role. Usually, DIHS/DRESS is associated with the reactivation of herpes virus family.

**Case report**

A 41 year-old man attended our department due to suspicion of medical allergy. Previously he was treated in the Department of Dermatovenerology for the severe erythematous rash spreaded all over the body, fever and painful lymph nodes. Symptoms appeared 3 days after the initiation of hepatitis C treatment with drug combination of ribavirin, dasabuvirin and ombitasvir/paritaprevir/ritonavir. The patient also had painful, grouped small blisters on the upper lip and skin around the nose (cold sores), and previously diagnosed psoriasis. Laboratory evaluation showed leucocytosis with eosinophilia, liver enzyme elevation, proteinuria, increase of CRP (22 mg/l) and high total IgE (1602 kU/ml). Treatment with local and systemic glucocorticosteroids, antihistamines, emollients and acyclovir was effective, and after improvement the patient was referred to an allergist. Additional laboratory testing was performed (FBC—with no changes, anti-HSV IgM—negative, anti-HSV IgG—1422 U/ml), as well as skin patch test for ribavirin, dasabuvirin and ombitasvir/paritaprevir/ritonavir, which revealed a positive reaction to ribavirin.

**Conclusion**

We describe a clinical case of drug hypersensitivity syndrome, which appears to be induced by the complex of factors—treatment with ribavirin for hepatitis C and reactivation of HSV.

**Consent to publish**

I confirm that I have received written consent for online publication from all patients described (or their parents/guardians), where there are images, videos, or 3 more indirect identifiers to be published.

#### P39 Severe cutaneous adverse reactions (SCARs) in Colombia: experience in three centers

##### Carlos D. Serrano^1^, Ricardo Cardona^2^, Dolly V. Rojas^3^, Luis F. Ramírez^1^, Nelson Angulo^4^, Diana L. Silva^3^, Edgardo Jares^5^

###### ^1^Fundación Valle del Lili, Cali, Colombia; ^2^Universidad de Antioquia, Medellín, Colombia; ^3^Universidad Icesi, Cali, Colombia; ^4^Clínica Sebastian de Belalcazar, Cali, Colombia; ^5^Fundación LIBRA, Buenos Aires, Argentina

**Correspondence:** Carlos D. Serrano - cdserranoreyes@gmail.com

*Clinical and Translational Allergy* 2018, **8(Suppl 3)**:P39

**Background**

Severe cutaneous delayed drug reactions (toxic epidermal necrolysis—TEN—, Stevens–Johnson syndrome—SJS—, acute generalized exanthematous pustulosis—AGEP—and drug reaction with eosinophilia and systemic symptoms/drug-induced hypersensitivity syndrome—DRESS/DiHS—) among others, are a rare but potentially fatal complications of drug treatment. Although its epidemiology has been described in different countries, it is unknown in Colombia. Our aim was to describe the epidemiological characteristics of severe cutaneous reactions to drugs in three high complexity centers in Colombia.

**Methods**

An online questionnaire was designed to report new and old cases (since 2013). It was a modified and adapted version of ENDA questionnaire for drug allergy interesting group. Sociodemographic data, type of reaction (TEN, SJS, DRESS-DiHS, AGEP), culprit drug (s), treatment, complications, mortality and sequelae, were included. Three centers from Colombia (two in Cali and one in Medellín) were included. An Excel database was created, in which cases were recorded and analyzed.

**Results**

Thirty one cases were reported. 20 (64.5%) were women. The median age was 45 years. 15 (48%) had DRESS/DiHS, 6 (19%) TEN, 3 (9.7%) SJS, 3 (9.7%) AGEP, 3 (9.7%) other not classified SCARs, and 1 (3%) overlapping TEN/SJS. The main culprit drugs were aromatic anticonvulsants in 14 cases (45%), beta lactam antibiotics in 6 (19%) and non-beta lactam antibiotics in 4 (13%). In 100% of the patients the suspect drug was withdrawn. Twenty one patients (84%) received systemic corticosteroids. Complications occurred in 13 cases (42%) and death in one patient (3%). Seven patients (22%) had some type of sequel.

**Conclusion**

In this preliminary study of SCARs in Colombia, DRESS/DiHS was the most frequently reported clinical entity and anticonvulsants were the main triggers. Most of patients received systemic corticosteroids. Complications were frequent, but mortality was low.

#### P40 Antituberculosis drug-associated DRESS: a case series

##### Marion Allouchery^1^, Sophie Logerot^2^, Benoit Ben Said^3^

###### ^1^Department of Clinical Pharmacology, Centre Hospitalier Universitaire, Poitiers, France; ^2^Department of Clinical Regional Pharmacovigilance Center, Centre Hospitalier Universitaire, Grenoble, France; ^3^SCARS national reference center, Lyon, France

**Correspondence:** Benoit Ben Said - bensaidprof@gmail.com

*Clinical and Translational Allergy* 2018, **8(Suppl 3)**:P40

**Background**

Although antituberculosis drug-associated drug reaction with eosinophilia and systemic symptoms (DRESS) is rarely reported, its diagnosis should not be dismissed. Its management implies an early withdrawal of suspected drugs.

**Methods**

The objective of this study was to describe the characteristics of antituberculosis drug-associated DRESS and to identify the most likely involved drugs. We searched for potential cases of DRESS with rifampicin, isoniazid, pyrazinamide, and ethambutol reported from January 1, 2005, to July 30, 2015, in the French pharmacovigilance database. A literature review was also performed.

**Results**

Sixty-seven cases of antituberculosis drug-associated DRESS were analyzed (40 women and 27 men, median age of 61 years). Liver and kidneys were the most frequently involved organs. Two patients died from DRESS. Skin tests were performed in 11 patients and were positive in 8 cases. Discrepancies between epicutaneous tests and reintroduction of the culprit drugs were observed for 2 patients with a premature reintroduction of antituberculosis drugs in 1 case. Antituberculosis drugs were the only suspects in 20 cases. As for the literature data, rifampicin was the most suspected drug because of its larger indications, but in case of tuberculosis infections, isoniazid was the most suspected drug.

**Conclusion**

We described the largest case series of first-line antituberculosis drug-associated DRESS in the literature. All antituberculosis drugs pose a risk of DRESS. An early withdrawal of the culprit drugs is essential. A drug allergy evaluation must be performed to optimize the second-line treatment of tuberculosis infection.

## Thursday 19 April 2018

### Skin reactions in drug allergy - Poster Walk 5

#### P42 Glibenclamide-induced photodistributed lichenoid eruption: a first reported case

##### Zohra Chadli, Amel Chaabane, Haifa Ben Romdhane, Najeh Ben Fadhl, Nadia Ben Fredj, Naceur A Boughattas, Karim Aouam

###### Pharmacology Laboratory, Faculty of Medicine, University of Monastir, University Hospital of Monastir, Monastir, Tunisia

**Correspondence:** Zohra Chadli - zohrachadly@ymail.com

*Clinical and Translational Allergy* 2018, **8(Suppl 3)**:P42

**Background**

Glibenclamide, also known as glyburide, is a sulfonylurea hypoglycemic drug used for the treatment of type 2 diabetes. Its common side effects include hypoglycaemia, gastrointestinal disorders (nausea, vomiting, abdominal pain…), cholestasis, muscle spasms and joint pain. Though uncommon, some cutaneous adverse effects might be involved. The most frequently reported ones are itching, urticaria, and maculopapular eruption. More serious reactions, such as Stevens–Johnson syndrome, toxic epidermal necrolysis and vasculitis, have also been documented. Lichenoid eruption has been very rarely reported with this drug. However, the association of glibenclamide with a photodistributed lichenoid eruption has not been previously reported. We describe herein a case of a glibenclamide-induced photodistributed lichenoid eruption.

**Case report**

A 75-year-old woman, without a personal history of atopy or other allergic diseases, presented with a skin eruption. According to her medical history, the eruption had started on the face approximately 2 months before and then spread to the neck and hands a few weeks later. The patient had already received a local symptomatic treatment without any improvement. The eruption had gradually darkened especially with exposure to the sun. The patient has been taking glibenclamide 15 mg/day and insulin for diabetes, and ursodesoxycholic acid 600 mg/day for primary biliary cirrhosis for 3 and 4 years now, respectively. The physical examination revealed circumscribed oval and round hyperpigmented macules and papules on the face, neck and dorsa of the hands. Their diameters varied from 1 to 8 cm. The pigmentation was reticulated and ranged from slate-gray to brown. No lesions were found on the mucous membranes. This clinical picture suggested a lichenoid eruption. A biopsy of the skin lesions was performed and the histopathological examination substantiated the diagnosis and showed a hyperorthokeratosis and acanthosis of the epidermis, a basal cell attack and a perivascular lymphocytic infiltrate in the superficial dermis. The role of glibenclamide was suspected and the drug was replaced by acarbose. The patient continued to take insulin and ursodesoxycholic acid. A gradual resolution of the lesions was observed over the following 2 months without any symptomatic medication.

**Conclusion**

We describe the first case of glibenclamide-associated photodistributed lichenoid eruption. Physicians should take note of this unusual glibenclamide-induced photosensitive eruption, even a long time after starting the medication. This drug should be withdrawn once lichenoid skin lesions appear.

**Consent to publish**

Consent to publish was obtained from the patient involved in this study.

#### P43 Fixed drug eruption - a case report

##### Joana Pita^1^, Raquel Gomes^2^, Rui Silva^2^, Carlos Loureiro^1^, Ana Todo Bom^1^

###### ^1^Coimbra Universitary Hospital Centre, Coimbra, Portugal; ^2^Trás-os-Montes e Alto Douro Hospital Centre, Vila Real, Portugal

**Correspondence:** Joana Pita - joana.s.pita@gmail.com

*Clinical and Translational Allergy* 2018, **8(Suppl 3)**:P43

**Background**

Fixed drug eruption (FDE) is an adverse drug reaction with skin and mucosal manifestations. It is characterized by one or multiple skin lesions that begin 1 or 2 weeks after exposure to a culprit drug. These lesions appear more commonly on the oral mucosa, lips and genitals.

When re-exposed, the patient may have reactivation of old lesions as well as new lesions in different locations, fading after several days, maintaining a grey or brown pigmentation.

Non-steroid anti-inflammatory drugs, antibiotics and anticonvulsants are the most implicated drugs in FDE. Cyclooxygenase 2 (COX-2) inhibitors have been rarely reported to cause FDE.

**Case report**

The authors present a case of a 69 years old female patient sent to our consultation due to a FDE. She was medicated with amoxicillin and clavulanic acid 7 days before a dental extraction. Immediately after dental extraction she was medicated with etoricoxib. One hour after etoricoxib intake, she noticed one bullous skin lesion on her back, with progressive worsening and further lesions on her back, abdomen and legs. She was treated with intravenous medication, oral and topical corticosteroids. Resolution took place 1 week later, although she maintained multiple residual lesions for months. No organ affection was observed in the first medical approach and on re-evaluation.

We performed cutaneous tests with amoxicillin, ampicillin and benzylpenicillin that were negative on immediate and 72 h readings, and specific IgE to antibiotics, also negative.

Based on other case reports we assumed that amoxicillin was the culprit drug. So, we performed a drug provocation test with oral etoricoxib. Approximately 30–40 min after intake the patient initiated general pruritus and burning sensation in the old lesions as well as other body parts. Old lesions became erythematous and larger and new lesions appeared in the back and perineal regions. She was medicated with clemastine, methylprednisolone and topical betamethasone. Surprisingly we confirmed that the culprit drug was etoricoxib.

A few months later, a drug provocation test with amoxicillin and clavulanic acid was performed, with negative result.

**Conclusion**

In this patient we had two possible culprit drugs. Clinical history and the known high frequency of fixed drug eruptions associated to antibiotics made us assume that amoxicillin and clavulanic acid was the culprit drug. Beyond that fact, when performing a drug provocation test in a patient with a fixed drug eruption, we don’t expect an immediate reaction. This is an important case report which enlightens the involvement of COX-2 inhibitors in fixed drug eruptions.

**Consent to publish**

Consent to publish was obtained from the patient involved in this study.

#### P44 Pantoprazole as the probable cause of a Stevens Johnson syndrome

##### Beatriz Rojas^1^, Vanesa Barba^1^, Ana Herrero^2^, Mar Garcés^3^, Lucía Ferrer^3^

###### ^1^San Jorge Hospital, Huesca, Spain; ^2^Nuestra Señora de Gracia Hospital, Zaragoza, Spain; ^3^Clínico hospital, Zaragoza, Spain

**Correspondence:** Beatriz Rojas - rojashijazo@yahoo.es

*Clinical and Translational Allergy* 2018, **8(Suppl 3)**:P44

**Background**

Stevens Johnson syndrome is a type of severe skin reaction. The most common cause is certain medications such as antiepileptic drugs, allopurinol, sulfonamide antibiotics and nSAIDs among others. Other causes can include infections such as *Mycoplasma pneumoniae* and cytomegalovirus or the cause may remain unknown.

**Case report**

The aim of this study was try to know the drug that caused a Stevens Johnson syndrome in our patient.

A 39 years old man with psoriatic arthritis in treatment with etanercept and neotigason started with fever and flu-like symptoms. He took an azithromycin tablet and 2 days later he started with a red rash that spreads and forms blisters. The affected skin eventually peeled off. His oral mucous was also affected but not other mucous. He was taking naproxen and pantoprazole 1 month ago because of his psoriasic arthritis.

A skin biopsy was performed and also a blood sample was taken.

We performed patch test with the standard series of contactants, with nSAIDs series, with proton bomb inhibitors omeprazole, pantoprazole, lansoprazole and with azithromycin.

The patient was challenged orally with aspirin.

We didn’t performed oral challenge test with Naproxen because risks did not lead the benefits of the test.

The skin biopsy demonstrated subepidermal bullae and perivascular areas infiltrated with lymphocytes, eosinophilic and mast cells.

Blood count revealed a normal white blood cell count.

Patch test were positive for colophony, methylisothiazolinone and pantoprazole.

The oral challenge test with aspirin was negative.

**Conclusion**

In the present case, Pantoprazole seems to be the cause of the Stevens Johnson Syndrome, but we were not able to confirm it because we did not perform an oral challenge test with Naproxen. Proton bomb inhibitors are not a common cause of this Syndrome. Perhaps these days with its frequent use proton bomb inhibitors become to be a common cause of Stevens Johnson Syndrome.

**Consent to publish**

Consent to publish was obtained from the patient involved in this study.

#### P45 Clinical features and drug characteristics of fixed drug eruption: a case series of 39 patients

##### Najah Ben Fadhel, Helmi Ammar, Zohra Chadly, Haifa Ben Romdhane, Nadia Ben Fredj, Naceur A. Boughattas, Karim Aouam, Amel Chaabane

###### Laboratory of Pharmacology, Faculty of Medicine of Monastir, University Hospital, Monastir University, Monastir, Tunisia

**Correspondence:** Najah Ben Fadhel - benfadhelnaj@hotmail.com

*Clinical and Translational Allergy* 2018, **8(Suppl 3)**:P45

**Background**

Fixed drug eruption (FDE) is a pattern of a drug-induced skin reaction. It is characterized by skin erythematous plaques that recur at the same site each time the drug is administered. Several drugs have been associated to such cutaneous reaction, including, anticonvulsant agents, sulfonamides, tetracyclines, paracetamol and non steroidal anti-inflammatory drugs (NSAID).

We describe herein an original case-series of FDE where the culprit was identified using patch test and/or a positive rechallenge with the suspected drug.

**Methods**

We included all cases of FDE reported in our department of pharmacovigilance of Monastir. The drug imputability of FDE was established according to Begaud and *al* method. Patch tests were performed in the involved and normal skin following the ENDA recommendations. Rechallenge test was performed for patients with negative skin tests.

**Results**

We reviewed 39 medical records of patients with suspected FDE during the period from 2004 to 2017. The culprit drug was identified in 35 patients. There were 14 males and 21 females. The mean age was 44 years. The number of episodes varies between one and five times and 23 patients have had at least two episodes. Multiple eruptions was diagnosed in 32 patients and bullous eruptions were observed in 12 cases. The arms were most commonly involved (51%) (n = 20), followed by legs, face and trunk. FDE lesions were seen in one case in genital areas.

NSAIDs were the most common causative agents, accounting for 41% of cases (n = 16). The other causative drugs included nine (23%) antibiotics and seven (17.9%) analgesics. The most common offending drugs were mefenamic acid in eight (20.5%) cases followed by paracetamol in six (15.4%) cases, and piroxicam in seven (17.9%) cases.

The determination of the culprit drug was made possible by a positive patch test in 24 out of 39 patients. For the remaining cases, accidental rechallenge or oral provocation helped to establish the culprit drug.

**Conclusion**

We demonstrated in our study that NSAIDs are the most causative drugs of FDE and pointed out the usefulness of drug patch tests in identifying the culprit drug. Raising awareness of this side effect and its elicitor drugs will increase the likelihood of prompt diagnosis and resolution after the drug withdrawal.

#### P47 Systemic allergic contact dermatitis associated with topical diltiazem and/or cinchocaine

##### Ana Luísa Moura^1^, Luis Santiago^2^, Coutinho Ines^2^, Margarida Gonçalo^2^

###### ^1^Department of Immunoallergology, Coimbra University Hospital, Coimbra, Portugal; ^2^Department of Dermatology, Coimbra University Hospital, Coimbra, Portugal

**Correspondence:** Ana Luísa Moura - al-moura@hotmail.com

*Clinical and Translational Allergy* 2018, **8(Suppl 3)**:P47

**Background**

Diltiazem is a calcium channel blocker that has been used topically for the treatment of anal fissures or hemorrhoids. Few cases of allergic contact dermatitis, and a single case of systemic contact dermatitis to diltiazem have been report.

**Case report**

A 61-year-old non-atopic female applied several topical pharmacologic agents for the treatment of a chronic anal fissure—Hemofissural^®^ (titanium dioxide, zinc oxide and tetracaine), Faktu^®^ (cinchocaine), Nupercainal^®^ (cinchocaine) and Anotrit^®^ (diltiazem chloride, polyoxyethylene sorbitan monopalmitate, caprylyl/capryl glucoside, cetylstearyl alcohol, benzoic acid and paraffin). One day after using the 2 last ones she reported perianal pruritus, 3 days later erythema and oozing of the perianal area and 5 days later she was observed also with a symmetric generalized erythematous eruption with vesicles particularly on the large body folds (buttocks and intergluteal cleft, pubic area, groins and upper inner thighs, inter and sub-mammary folds and axillae). She stopped the medications and applied topical corticosteroid in the affected areas with complete recovery within 10 days.

The patient had never used topical or systemic diltiazem neither any other calcium channel blocker and she never suffered local or systemic symptoms from local anesthesia in dental treatments.

We performed patch tests with the Portuguese baseline series and the series of topical anesthetics and topical drugs, diltiazem 10% pet (Chemotechnique diagnostics^®^, Sweden) and patient’s medications as is. Patch tests were applied on the upper back for 48 h and readings performed at day 3 showed positive reactions to diltiazem 10% pet (+++), caine mix III (benzocaine, cinchocaine, tetracaine) 10% pet (+++), cinchocaine 5% pet (+++), mepivacaine 1% pet (++), Anotrit^®^ (+++) and Nupercainal^®^ (+++). All other topical anesthetics were negative as well as ingredients from Anotrit^®^ cream. Further patch tests with several calcium channel blockers revealed a positive reaction only for diltiazem (+++).

She was advised to avoid contact with all products containing these substances, including oral ingestion, and she is completely free from symptoms for more than 4 months.

**Conclusion**

Systemic absorption of diltiazem and/or cinchocaine through the anal and rectal mucosae can justify the generalized systemic contact dermatitis, seldom reported. Diltiazem used systemically can also cause of delayed drug eruptions often with positive patch tests. This example of diltiazem further strengthens the relation between allergic contact dermatitis and T-cell mediated drug reactions, where systemic contact dermatitis can be considered one type of such drug eruptions.

**Consent to publish**

I confirm that I have received written consent for online publication from all patients described (or their parents/guardians), where there are images, videos, or 3 more indirect identifiers to be published.

#### P48 Codeine-induced acute generalized exanthematous pustulosis without IL36RN mutations

##### Zohra Chadlia, Amel Chaabane, Emna Kerkeni, Amira Djobbi, Nadia Ben Fredj, Naceur A. Boughattas, Karim Aouam

###### Pharmacology Laboratory, Faculty of Medicine, University of Monastir. University Hospital of Monastir, Monastir, Tunisia

**Correspondence:** Zohra Chadlia - zohrachadly@ymail.com

*Clinical and Translational Allergy* 2018, **8(Suppl 3)**:P48

**Background**

Acute generalized exanthematous pustulosis (AGEP) is a rare and severe cutaneous adverse reaction. Recent studies have suggested an association between mutations in interleukin-36 receptor antagonist gene (IL-36RN) and the onset of pustular generalized such as generalized pustular psoriasis (GPP) and AGEP. In literature, only one case of AGEP induced by dihydrocodeine phosphate in a patient with psoriasis vulgaris and a heterozygous IL36RN mutation has been reported. Objective: To report a rare case of codeine-induced AGEP in a patient with a history of psoriasis without IL36RN mutations.

**Case report**

A woman, with a history of psoriasis, had received Voltarene Retard^®^ (diclofenac sodium), Co-Algesic^®^ (paracetamol-codeine) and Lanzopral^®^ (lansoprazole) for knee pain. Two weeks later, she developed fever and pruritic skin eruption. Physical examination showed a diffuse erythema marked in the intertriginous folds dotted with nonfollicular pustules. Histopathological examination was in accordance with AGEP. Patch tests performed with Voltarene^®^, Co-Algesic^®^ and Lanzopral^®^ were all negative. After obtaining informed consent from patient, a sequential oral provocation tests were separately performed. Two hours after administration of Co-Algesic^®^, the patient developed an identical skin rash. After informed consent, genomic DNA was extracted from venous blood sample. The four coding exons of IL36RN gene including its intron–exon boundaries were amplified by polymerase chain reaction (PCR) and Sanger sequencing was performed. No IL36RN mutations were found.

**Conclusion**

We have reported an unusual case of AGEP caused by codeine in a patient with a history of psoriasis and confirmed by an oral provocation test. In our case, we have shown that IL36RN gene mutation is not a constant condition in drug-induced AGEP. Clinicians should be aware of this side effect of codeine especially in patients with a history of psoriasis. More studies are needed to clarify the association between drug-induced AGEP and IL36RN gene mutations.

**Consent to publish**

Consent to publish was obtained from the patient involved in this study.

#### P50 Sensitization to antibiotics in patients with drug reaction with eosinophilia and systemic symptoms (DRESS) to alopurinol or anticonvulsants

##### Luis Santiago, André Pinho, Margarida Gonçalo

###### CHUC, Coimbra, Portugal

**Correspondence:** Luis Santiago - luisgsantiago2@gmail.com

*Clinical and Translational Allergy* 2018, **8(Suppl 3)**:P50

**Background**

Drug reaction with eosinophilia and systemic symptoms (DRESS) is a severe cutaneous adverse drug reaction (CADR) with an estimated an annual incidence of 0.9/100,000, where drug-specific immune response is a key factor, with antiepileptic agents and allopurinol as the most frequently reported culprits.

Nevertheless, the pathogenesis of DRESS is complex and not fully understood: genetic deficiency of detoxifying enzymes; association with HLA haplotypes, like HLA-B*57-01 for abacavir and HLA-B*58-01 for allopurinol; virus–drug interactions (HHV-6 reactivation, eventually induced by drugs); drug-induced immunosuppression followed by an inflammatory immune recovery syndrome; and drug-specific T-cells have been isolated from the blood and skin in DRESS induced by lamotrigine and carbamazepine, and positive in vitro lymphocyte proliferation assays and positive patch tests (in 72.2% of the cases induced by carbamazepine although not for alopurinol) suggest a T-cell-mediated reaction.

**Methods**

We reviewed patch test data of patients referred to our dermatology clinic between 2010 and 2017 for the study of a well-characterized DRESS.

**Results**

We describe eight patients (6 males and 2 females; mean age 45.9 years) with DRESS (5 to carbamazepine, 1 to phenytoin and 2 to alopurinol) who developed positive patch tests to different antibiotics tested at 10% pet (6 with amoxicillin, 5 with ampicillin, 2 with cefoxitin and 1 with vancomycin, cefotaxime and benzylpenicillin) that were administered concomitantly during the alopurinol or anticonvulsivant-induced DRESS. Patch tests were positive to the anticonvulsants and negative to alopurinol, but the clinical history and the presence of HLA-B*58-01 favours imputability of this drug.

Four cases were re-patch test and three patients remained positive to the antibiotics and carbamazepine 3–28 years thereafter, although in one case phenytoin positivity was lost.

**Conclusion**

Antibiotics are frequently administered during DRESS, due to the initial prodrome, which mimics an acute bacterial infection. Although cross-reactions between anticonvulsants are common, chemical/molecular structure differences between the antibiotics and alopurinol/anticonvulsants seem to rule out this hypothesis.

It has been suggest that during DRESS, exposure to non-related drugs, such as antibiotics, amitriptyline and acetaminophen, could induce a neosensitization, with a new DRESS episode occurring during re-challenge with the second drug, even in the absence of the initial culprit. The suggested mechanism is a massive nonspecific activation of the immune system, after the initial immunosuppression stage, which enables antigen presentation leading to the development of specific T cells and neosensitization.

Reproducibility of positive patch test reactions to antibiotics, even after several years, seems to support the strength of this T cell reaction.

#### P51 SDRIFE due to azithromycin: case report

##### Sofia Martins Farinha, Barbara Kong Cardoso, Elza Maria Tomaz, Filipe Fernando Inácio

###### Centro Hospitalar de Setúbal, Setúbal, Portugal

**Correspondence:** Sofia Martins Farinha - sofiamf_@hotmail.com

*Clinical and Translational Allergy* 2018, **8(Suppl 3)**:P51

**Background**

Symmetrical drug related intertriginous and flexural exanthema (SDRIFE), formerly named baboon syndrome, is an uncommon cutaneous reaction that occurs after the systemic administration of drug-related allergens. It is characterized by five clinical criteria: occurrence after exposure to systemic drugs; sharply-demarcated erythema of the buttocks and/or V-shaped erythema of the thighs; involvement of at least one other flexural fold; symmetry; absence of systemic symptoms. A type IV delayed hypersensitivity immune response is thought to be involved, thus patch tests are used to identify the causative drug. However they are negative in one-third to one-half of the patients. In most cases beta-lactam antibiotics are the trigger, but other drugs may be involved. On the other hand, allergic reactions to macrolide antibiotics appear to be relatively uncommon (0.4–3% of treatments), making them be seen as “safe” regarding to allergy. Often a drug reaction is empirically attributed to a drug and a confirmation study is not requested.

**Case report**

A 83-year-old woman was referred to our Department with a history of symmetrically pruritic erythema located in inner face of thighs, groin, armpits, buttocks and abdomen 8 h after the first intake of azithromycin for a respiratory infection. The lesions evolved with a little peeling and slowly improved with oral corticoid, topical corticosteroid and skin hydration leaving lesions of hyperpigmentation.

The patient remembered having a similar reaction some years ago, which was attributed to an antibiotic prescribed for pneumonia. Consulting patient files the episode was identified, 8 years before. Amoxicillin–clavulanate plus azithromycin were prescribed at that time and amoxicillin was taken as the culprit drug.

Patch tests with amoxicillin–clavulanate and azithromycin (readings at 48 and 96 h) were negative. LTT was negative for amoxicillin–clavulanate, but and positive for azithromycin.

Final diagnosis was SDRIFE due to azithromycin.

**Conclusion**

To our knowledge this is the first report of a SDRIFE related to azithromycin. Lymphocyte transformation test (LTT) played a key role in the diagnosis, considering the negativity of patch tests. Assuming culprit drugs based on frequency criteria must be avoided and an allergic work-up must be performed.

**Consent to publish**

Consent to publish was obtained from the patient involved in this study.

#### P52 Skin patch testing with offending drugs in patients with drug reactions with eosinophilia and systemic symptoms (DRESS)

##### Sri Awalia Febriana^1^, Graciella Regina^2^, Fajar Waskita^3^, Niken Indrastuti^3^

###### ^1^Departement of Dermatology and Venereology, Faculty of Medicine, Universitas Gadjah Mada, Yogyakarta, Indonesia; ^2^Dept. of Dermatology and Venereology Faculty of Medicine Universitas Katolik Atmajaya, Jakarta, Indonesia; ^3^Dept. of Dermatology and Venereology Faculty of Medicine Universitas Gadjah Mada, Yogyakarta, Indonesia

**Correspondence:** Sri Awalia Febriana - awalia_febriana@ugm.ac.id

*Clinical and Translational Allergy* 2018, **8(Suppl 3)**:P52

**Background**

Adverse cutaneous drug reactions (ACDR) are drug-induced response of the skin in regards of its structure or function, appendages or mucous membranes. On a worldwide scale, ACDR often occur in 2–3% of all hospitalized patients, with approximately 1 in 1,000 people experiencing a severe form of cutaneous drug reaction (Roujeau & Stern, 1994). Drug reaction with eosinophilia and systemic symptoms (DRESS) syndrome is a potentially life-threatening adverse drug-induced reaction, with an estimated mortality of 10%. (Husain, 2013)

Drug provocation tests (DPTs) are often needed when evaluating patients with suspected drug hypersensitivity reactions. In spite of the advantages that DPT holds over all the other test procedures, it cannot be disputed that DPTs represent a potential risk to the patient, and contraindicated for patients with DRESS.

Drug skin pacth tests can reproduce delayed hypersensitivity to drugs and entail a moderate reexposure of patients to offending drugs (Barbaud, 2014) and considered as a good choice for evaluating the patients with severe drug reaction including DRESS.

**Case report**

A 30 years-old female came to the dermatology clinics Sardjito hospital for having the skin patch test to know the causative drugs. Three months before, she was hospitalised in Sardjito hospital with the diagnosis of DRESS. She was suspected cephadroxil and mefenamic acid as a causative drugs. Those drugs was administered after she underwent the breast tumors resection. Three weeks after consumed those drugs, she had a skin rash all over the body and fever.

Based on the physical examination she had a lympnode enlargement, lymphocytosis with atypical lymphocyte, trombocytopenia and hepatic problems Patients was diagnosed with probable DRESS and RegiScar score 5.

Patch test was done in the dermatology polyclinic 3 months later with cephadroxil and mefenamic acid, ranitidin and paracetamol diluted in vaselin album and aquadest 30%. The result showed (++) reactions to cephadroxil in both preparations. There was negative results in other preparations after 48, 72 and 96 h reading.

**Conclusion**

In this case report, we reported a 30-years-old female with the history of DRESS possibly due to cephadroxil and mefenamic acid. Patch test with the offending drugs showed strong positive reactions in patch test with cephadroxil 30% in vaseline album and aquadest.

**Consent to publish**

I confirm that I have received written consent for online publication from all patients described (or their parents/guardians), where there are images, videos, or 3 more indirect identifiers to be published.

## Thursday 19 April 2018

### Diagnosis and pathophysiology - Poster Walk 6

#### P53 Characterization of the HLA-B*57:01 immunopeptidome in the presence of flucloxacillin

##### James C. Waddington^1^, Xiaoli Meng^1^, Patricia T. Illing^2^, Arun Tailor^1^, Rosalind Jenkins^1^, Anthony W. Purcell^2^, Dean J. Naisbitt^1^, B. Kevin Park^1^

###### ^1^CDSS, Department of Clinical and Molecular Pharmacology, The University of Liverpool, Liverpool, United Kingdom; ^2^Infection and Immunity Program, Monash Biomedicine Discovery Institute and Department of Biochemistry and Molecular Biology, Monash University, Clayton, Australia

**Correspondence:** James C. Waddington - hljwaddi@liv.ac.uk

*Clinical and Translational Allergy* 2018, **8(Suppl 3)**:P53

**Background**

The β-lactam antibiotic flucloxacillin can cause idiosyncratic drug-induced liver injury (iDILI), yet despite intensive research the mechanisms are unknown. Genetic association between flucloxacillin iDILI and *HLA*-B*57:01, and flucloxacillin-specific CD8+ T-cells in patients with iDILI, suggest immune-mediated mechanisms are involved. Covalent binding of flucloxacillin to proteins, followed by antigen processing and presentation of drug-derived antigenic determinants, may drive the adverse event. Currently, little is known about the proteins that are haptenated by flucloxacillin, and whether the modification of intracellular proteins leads to the presentation of functional antigenic drug-modified peptides. This study aimed to (1) identify intracellular binding and flucloxacillin-modified naturally-processed peptides that might act as T-cell antigens; (2) investigate the immunogenicity of flucloxacillin-modified HLA-B*57:01 peptides using flucloxacillin-specific T-cells from iDILI patients in functional T-cell assays.

**Methods**

C1R-B*57:01, B-lymphoblast cells transfected with *HLA*-B*57:01, were incubated with flucloxacillin for 48 h. A flucloxacillin-specific antibody was used as a precise tool for pinpointing intracellular haptenated proteins that were subsequently identified by state-of-the-art LC–MS. This approach was also used to study the uptake, distribution and protein binding of flucloxacillin in immortalised and primary cells from liver and the immune system. MHC molecules were isolated from C1R-B*57:01 cells, and naturally-processed antigenic peptides were eluted, fractionated using RP-HPLC and characterised by LC–MS/MS analysis.

**Results**

Flucloxacillin modified proteins were detected, and their potential contribution to the development of iDILI will be explored. Of the thousands of naturally processed peptides eluted from flucloxacillin-treated C1R-B*57:01 cells, a total of 12 flucloxacillin-modified peptides were detected; however, only a few peptides were fully annotated to confirm that flucloxacillin was covalently bound to specific amino acid residues. Moreover, direct modification of HLA-B*57:01 was also observed. Flucloxacillin-modified peptides were successfully synthesized and purified, to be used in functional T-cell assays.

**Conclusion**

Formation of covalently modified drug-protein adducts is thought to be the molecular initiating event in many immunological drug reactions. However, our data are the first to fully characterize the nature of the drug-derived antigens presented to the immune system in the context of a HLA molecule. We have shown that flucloxacillin-modified peptides, derived from either intracellular processing of flucloxacillin-modified proteins or direct modification of HLA-B*57:01 peptides, can be presented to the immune system. Direct modification of HLA-B*57:01 was also detected, which may have potential to alter the peptide repertoire presented by HLA B*57:01.

#### P54 Cyto-LTT: a sensitive tool for the diagnosis of delayed-type drug hypersensitivity

##### Theresa Fachruddin^1^, Antonia Bünter^2^, Anna Gschwend^1^, Oliver Hausmann^2^, Florian Pichler^2^, Christoph Schlapbach^1^, Werner J. Pichler^2^

###### ^1^University Hospital Inselspital, Bern, Switzerland; ^2^Adverse Drug Reactions – Analysis & Consulting (ADR-AC) GmbH, Bern, Switzerland

**Correspondence:** Theresa Fachruddin - theresa.fachruddin@adr-ac.ch

*Clinical and Translational Allergy* 2018, **8(Suppl 3)**:P54

**Background**

The diagnosis of delayed-type drug hypersensitivity (DH) is based on patient history, skin testing, challenge tests and in vitro tests. The lymphocyte transformation test (LTT) is the most commonly used in vitro tool for this purpose bearing a high specificity. However, limited sensitivity and the use of radioactivity restrict its use and demand for alternatives. In our work, we could demonstrate an enhanced sensitivity by quantification of multiple mediators in supernatants of well-defined drug allergic patients.

**Methods**

So far, 22 well-defined drug allergic patients (DRESS, MPE, SJS/TEN, AGEP) are included and > 70 patients from routine diagnosis. PBMCs were isolated and cultured for 7 days with and without the culprit drug, which in the majority of cases included Amoxicillin (n = 19). Subsequently, supernatants were taken and levels of IL-5, IL-13, IFNg, Granzyme B and Granulysin were quantified with either ELISA or by bead assays using flow cytometry (“Cytokine-LTT”, short ***cyto*-*LTT***).

**Results**

We could observe that different cytokines are released: IL-5 and IL-13 bear a very high specificity (85–95%), comparable to the LTT. Sensitivity was improved from 30 to 60% for the LTT to > 75% for IL-5. Granzyme B is sensitive for rather cytotoxic reactions (e.g. SJS/TEN), but is limited by its specificity (60–70%). We could demonstrate that some cytokine release occurs in the absence of strong proliferation, which explains the better sensitivity of ***cyto*-*LTT*** compared to conventional LTT.

**Conclusion**

The ***cyto*-*LTT*** is a simple approach which improves the sensitivity of in vitro assays by analyzing several mediators at once, and still retains excellent specificity. In addition, by carefully choosing phenotype-specific cytokines the ***cyto*-*LTT*** helps in defining the clinical picture of DH. The main disadvantage is the rather high cost of reagents and equipment.

#### P55 Nitroso sulfamethoxazole forms multiple haptenic determinants with cysteine, lysine and tyrosine

##### Arun Tailor^1^, James Waddington^1^, Jane Hamlet^1^, Laila Kafu^1^, John Farrell^1^, Gordon Dear^2^, Paul Whitaker^3^, Dean J. Naisbitt^1^, B. Kevin Park^1^, Xiaoli Meng^1^

###### ^1^Department of Molecular & Clinical Pharmacology, MRC Centre for Drug Safety Science, University of Liverpool, Liverpool, United Kingdom; ^2^GlaxoSmithKline, Park Road, Hertfordshire, Ware, United Kingdom; ^3^Regional Adult Cystic Fibrosis Unit, St. James’s Hospital, Leeds, United Kingdom

**Correspondence:** Arun Tailor - a.tailor@liverpool.ac.uk

*Clinical and Translational Allergy* 2018, **8(Suppl 3)**:P55

**Background**

Severe delayed-type hypersensitivity reactions occur in certain patients upon treatment with sulfamethoxazole (SMX). Metabolism of SMX by CYP2C9 can form the chemically reactive metabolite sulfamethoxazole hydroxylamine which undergoes spontaneous auto-oxidation to the highly reactive product nitroso-sulfamethoxazole (SMX-NO). The ability of SMX-NO to activate T-cells from hypersensitive patients has been studied in detail. T-cells are activated through binding of the metabolite to proteins or through the direct modification of MHC-bound peptides. In vitro, SMX-NO has been shown to modify cysteine residues in glutathione, designer peptides and proteins; however, the presence of these adducts have not yet been identified in patient serum. Aim: To characterize the SMX-NO modification of HSA in vitro *and* identify SMX-NO-HSA adducts in patient serum.

**Methods**

SMX-NO was incubated with HSA at a range of molar ratios and free drug was removed. HSA was depleted from patient serum and from cell culture media using affinity chromatography. All samples were analysed using a QTRAP 5500 mass spectrometer for MRM or QTOF 5600 for discovery and accurate mass analysis. The established in vitro priming assay was used to prime naïve T-cells isolated from healthy volunteers to SMX-NO to characterise adducts in cell culture media.

**Results**

In addition to the known cysteine adducts, multiple haptenic structures were found on lysine and tyrosine residues when SMX-NO was incubated with HSA in vitro. On lysine residues two haptenic structures were identified including an arylazoalkane adduct and a Schiff base adduct. On tyrosine residues a direct displacement of the nitroso functional group by the hydroxyl side chain was observed. Interestingly, these adducts are labile to heat and susceptible to hydrolysis as shown by the presence of allysine. SMX modified HSA adducts on cysteine and lysine were also detected in patients taking SMX therapy.

**Conclusion**

Multiple haptenic adducts were successfully identified in vitro and in patient serum. The presence of these adducts could provide a reasoning for the immunogenicity of SMX therapy and the strong responses to SMX-NO observed in T-cell culture assays. Also, the degradation of these adducts to allysine could indicate signal 2 required for T-cell activation. Mass spectrometric identification of modified proteins could have a role in the diagnosis of drug hypersensitivity.

#### P56 Characterization of healthy donor-derived T-cell responses specific to telaprevir diastereomers

##### Khetam Alhilali, Zaid Al-Attar, Andrew Gibson, B. Kevin Park, Dean J. Naisbitt

###### ^1^Dept. Molecular & Clinical Pharmacology, MRC Centre for Drug Safety Science, University of Liverpool, Liverpool, United Kingdom

**Correspondence:** Khetam Alhilali - adriana.ariza@ibima.eu

*Clinical and Translational Allergy* 2018, **8(Suppl 3)**:P56

**Background**

Hepatitis C virus (HCV) is a potentially life-threatening viral infection. Most infected patients develop chronic HCV infection ultimately resulting in severe liver disease including hepatocellular carcinoma, liver fibrosis and cirrhosis. Treatment with teleprevir, an NS3.4.A protease inhibitor for use against HCV genotype 1, increases the frequency of patients that achieve viral control from 39% on standard dual therapy, to 70%. However, the triple regimen is associated with severe skin reactions including the DRESS and SJS. These manifestations, along with the delayed onset and slow resolution after drug discontinuation are indicative of a T-cell mediated hypersensitivity reaction. Due to competition from newer and safer therapies, telaprevir was withdrawn from use; however, it remains an interesting model compound to study mechanisms of drug hypersensitivity reactions. Aims: To study whether telaprevir and/or its diastereomer activates T-cells from healthy donors.

**Methods**

Telaprevir in its S-configurated therapeutic form or the alternative R-diastereomer were either cultured directly with healthy donor-derived PBMC, or with naïve T-cells and autologous matured dendritic cells in an in vitro T-cell priming co-culture system. After priming, cultures were restimulated with a second batch of dendritic cells and analysed for drug-antigen specific proliferation by [^3^H]-thymidine incorporation. Cultures were then serially diluted to perform T-cell cloning. From two healthy donors, we identified T-cell clones responsive to telaprevir and the R-diastereomer.

**Results**

T-cell clones proliferated and secreted IFN-γ, IL-13, and Granzyme B in response to culture with telaprevir and the diastereomer at the same concentrations, but did not respond to the M11 metabolite, which had been identified as a potential immunogen in animal studies. Furthermore, the response in granzyme B-secreting cytotoxic CD8^+^ T-cell clones was MHC I-restricted and dependant on the presence of soluble drug. Flow cytometric analysis showed that responsive T-cell clones most highly expressed CCR4 (skin homing) and CXCR3 (migration to peripheral tissue) and expressed one of three distinct TCR Vβs; TCR Vβ 2, 5.1, or 22.

**Conclusion**

In this study, we identify the propensity of telaprevir to generate skin-homing cytotoxic T-cells that may induce the cutaneous reactions observed in patients. These responses were induced using cultures from drug-naïve healthy donors and so define the utility of in vitro human platforms to explore the requirements for naïve T-cell priming.

#### P57 Lymphocyte activation test and cross-reactivity of proton pump inhibitors-induced SCAR

##### Wen-Hung Chung^1^, Chun-Bing Chen^2^, Ying-Wen Wang^1^

###### ^1^Department of Dermatology, Drug Hypersensitivity Clinical and Research Center, Chang Gung Memorial Hospital, Linkou, Taiwan; ^2^Department of Dermatology, Drug Hypersensitivity Clinical and Research Center, Chang Gung Memorial Hospital, Keelung, Taiwan

**Correspondence:** Ying-Wen Wang - hunter76811@gmail.com

*Clinical and Translational Allergy* 2018, **8(Suppl 3)**:P57

**Background**

Proton pump inhibitors (PPI) has been known to induce type I hypersensitivity reactions. However, severe delayed type hypersensitivity reactions (DHR) induced by PPI, such as Stevens–Johnson syndrome (SJS), toxic epidermal necrolysis (TEN), or drug rash with eosinophilia and systemic symptoms (DRESS), are rarely reported. We conducted a study of a large series of PPI-related DHR, followed up their tolerability to alternative anti-ulcer agents, and use the lymphocyte activation test to investigated the T cell reactivity to PPI in PPI-related DHR patients.

**Methods**

We retrospectively analyzed patients with PPI-related DHR from multiple medical centers in Taiwan. We analyzed the causative PPI, clinical manifestations, organ involvement, treatment, and complications. We also followed up the potential risk of cross-hypersensitivity or tolerability to other PPI after their hypersensitivity episodes. Drug lymphocyte activation test (LAT) was conducted by measuring granulysin and interferon-γ to confirm the causalities.

**Results**

There were 69 cases of PPI-related DHR, including SJS/TEN (n = 27) and DRESS (n = 10). The LAT by measuring granulysin showed a sensitivity of 59.3% and specificity of 96.4%. Esomeprazole was the most commonly involved in PPI-related DHR (51%).

**Table 1:**
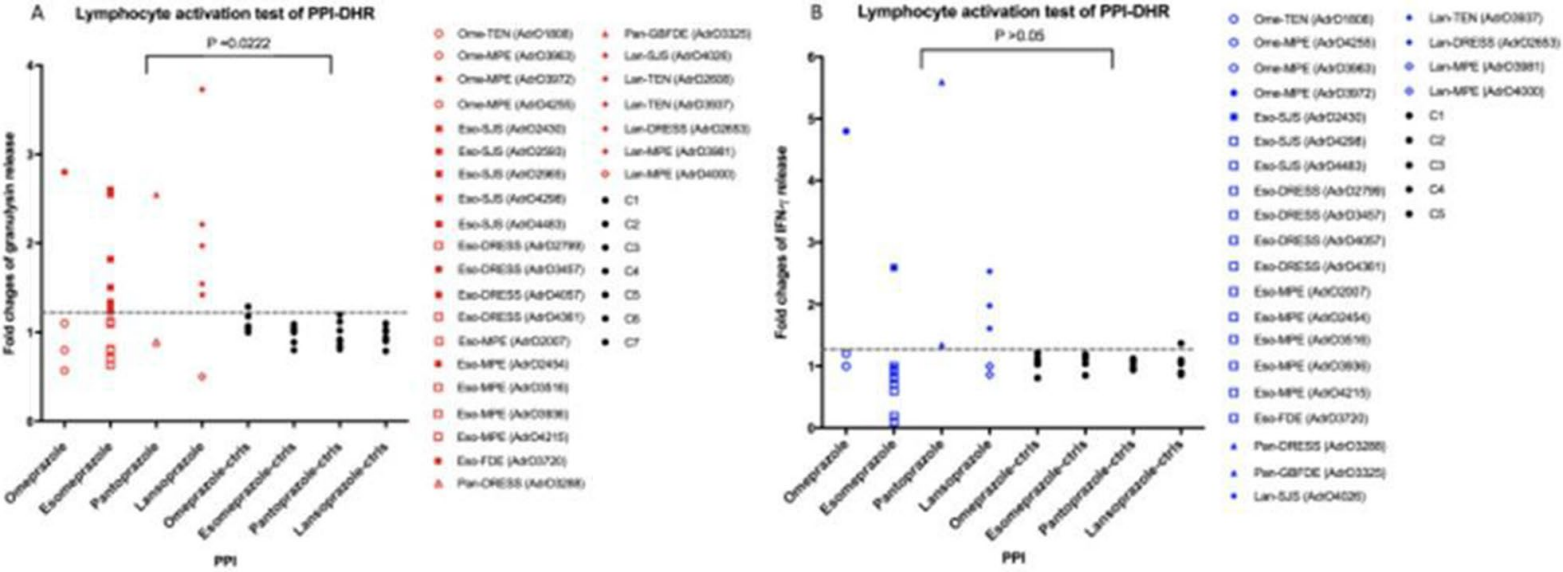
Allergological investigation by lymphocyte activation text of PPI-related DHR

Thirteen patients allergic to one kind of PPI could tolerate other structurally different PPI without cross-hypersensitivity reactions, whereas three patients developed cross-hypersensitivity reactions to alternative structurally similar PPI. The cross-reactivity to structurally similar PPI was also observed in LAT assay (Table 1).

**Conclusion**

PPIs have the potential to induce life-threatening DHR. In patients when PPI is necessary for treatment, switching to structurally different alternatives should be considered.

#### P58 Utility of basophils activation tests in drug allergy clinical practice

##### Ravishankar Sargur^1^, Alla Nakonechna^2^, Kirsty Swallow^1^, William Egner^2^

###### ^1^Immunology Protein Reference Unit, Sheffield, United Kingdom; ^2^Clinical Immunology & Allergy Unit, Sheffield Teaching Hospitals NHS Foundation Trust, Sheffield, United Kingdom

**Correspondence:** Ravishankar Sargur - ravishankar.sargur@sth.nhs.uk

*Clinical and Translational Allergy* 2018, **8(Suppl 3)**:P58

**Background**

Basophil activation tests (BATs) have been shown to be a useful research tool in the assessment of allergy, with many reported cases of how they can be used to assess responses to anaesthetics, antibiotics and venoms. We validated the assay for for routine clinical use in patients attending Sheffield Allergy clinic by performing the test on patients presenting with likely anaesthetic, antibiotic drug allergy.

**Methods**

The assay detects CD63 expression after allergen stimulation to assess basophil response. The results of all the basophil activation tests performed since the assay was introduced in 2008 were compared to the clinical outcome (where available). Clinical outcomes were obtained via patient notes, letters and at clinical multidisciplinary team (MDT) meetings.

**Results**

In total 355 tests were performed on 150 patients. 12 patients were from external hospitals and clinical outcome is unknown therefore the data has not been included in the statistics.

**Table 1:** Results

Allergen	Number of tests	Sensitivity (%)	Specificity (%)	PPV (%)	NPV (%)
Ceftazidine	7	*	100	*	85.7
Suxamethonium	11	42.9	100	100	50
Propofol	7	*	100	*	85.7
Atracurium	13	14.3	100	100	50
Rocuronium	9	*	100	*	66.7
Ibuprofen	7	*	80	0	66.7
Amoxicillin	25	10	100	100	62.5
Paracetamol	8	*	100	*	62.5
Metronidazole	7	*	100	*	100
Bee	7	50	100	100	83.3
Latex	6	100	100	100	100
Wasp	9	75	100	100	33.3
Chlorhexidine	13	50	100	100	70
Flucloxicillin	10	20	100	100	55.6
Gentamycin	8	*	100	*	87.5
Pancuronium	6	*	100	*	66.7
Augmentin	7	*	100	*	14.3
Teicoplanin	8	33.3	100	100	33.3

3 patients were non-responders to the test (Table 1). Therefore a total of 327 results were evaluable for 103 different allergens (29 removed which were tested on non-responders and external requests).

21 had inconclusive results or an unclear clinical outcome at the time of the audit e.g. Awaiting challenge test.

35 were true positive results; 182 were true negative results; 2 were false positive results.

87 were false negative results.

Overall: sensitivity = 28.7%, specificity = 98.9%, PPV = 94.6% and NPV = 67.7%.

**Conclusion**

From our experience it is clear that the outcomes of BATs have varying degrees of correlation with SIgE, SPT/IDT and the advice given to the patient.

BATs provide additional information in difficult cases but cannot be used in isolation.

They share similar problems of false negativity and false positivity with sIgE, and skin testing.

Interpretation requires considerable allergy experience but they can aid in decision making when conventional allergy tests give conflicting results.

Additional information can be gained in difficult cases with regards to the significance of positive SPT/IDT in the absence of good clinical history.

BATs are a complementary diagnostic tool in the study of immediate hypersensitivity reactions and may make it possible to avoid challenges in some cases.

#### P59 Chlorhexidine allergy: in vitro data predict a good tolerability to alexidine

##### Antonia Buenter^1^, Nicole Mueller^1^, Suran Fernando^2^, Didier Ebo^3^, Martin Glatz^4^, David Spoerl^5^, Arthur Helbling^6^, Oliver Hausmann^7^, Nisha Gupta^8^, Werner J. Pichler^1^

###### ^1^ADR-AC GmbH, Bern, Switzerland; ^2^Department of Clinical Immunology and Allergy, Sydney, Australia; ^3^Royal North Shore Hospital, Antwerp, Belgium; ^4^Antwerp University Hospital, Zurich, Switzerland; ^5^University of Antwerp, Geneva, Switzerland; ^6^Allergiestation, Bern, Switzerland; ^7^University of Zurich, Bern, Switzerland; ^8^Department of Clinical Immunology and Allergy, Reading, United States

**Correspondence:** Antonia Buenter - antonia.buenter@adr-ac.ch

*Clinical and Translational Allergy* 2018, **8(Suppl 3)**:P59

**Background**

Chlorhexidine (CHX) is a widely utilized disinfectant for skin and mucosal surfaces as well as medical devices. CHX is a biguanide compound with two chlorophenyl endings linked by a hexamethylene chain. CHX can cause rarely IgE-mediated anaphylaxis. Alexidine (ALX), another biguanide with similar hexamethylene center but 2-ethyl-hexyl-end groups instead of the aromatic endings, has similar bactericidal properties and represents a potential substitute for CHX. The allergic potential of ALX is unknown and was the focus of this study.

**Methods**

23 sera from Switzerland with allergic reactions to CHX, 9 sera with elevated CHX-specific IgE from Australia, and 5 sera from Belgium were included. All patients (n = 36) had a clinically well documented CHX reactivity with anaphylaxis or urticaria upon parenteral and/or mucocutaneous exposure, with positive skin test (ST) and/or positive CHX-ImmunoCAP test; the latter being an IgE test for chloroguanide (CG). For determining ALX cross-reactivity/reactivity, we performed direct and indirect basophil activation tests (BAT; with CD63 and CD203c as activation markers) with CHX and ALX (n = 26). ALX reactivity was also evaluated in those sera with IgE to CHX/CG of > 0.7 kU/ml by IgE inhibition tests (n = 28). To detect isolated biguanide reactivity, we also investigated the reactivity to polyhexanide by the ImmuoCAP FEIA method (ThermoFisher Scientific, Uppsala, Sweden) in 28 sera. Five patients agreed for re-testing via skin tests with ALX and CHX.

**Results**

15/26 patients showed a positive BAT with CHX and 3/21 with ALX, but only if basophils were pretreated with IL-3. An Inhibition of IgE-binding to the CHX-ImmunoCAP > 60% was achieved in 26/30 sera by CG, 13/30 sera by CHX, and 2/30 sera by ALX. IgE reactivity to polyhexanide as surrogate marker for biguanide reactivity was detected in only 4/30 sera. In 5 patients ST were repeated to CHX and compared to ALX: Only 1/5 was also positive to ALX, confirming the ALX reactivity in BAT of this patient (also sensitive to many other contact allergens).

**Conclusion**

The IgE response to CHX appears to be polyclonal: While CG seems to be the main epitope, an additional reactivity with other antigenic components residing in other biguanides was also evident but appears to be rare with less affinity (< 20%).

#### P60 How do we need to interpret analytical results of specific IgE of the ImmunoCAP ß-lactams tests in relation to elevated total IgE?

##### Douwe De Boer^1^, Manou Kessels^1^, Gwen Keulen^1^, Lotte Strijbos^1^, Paul Menheere^1^, Chris Nieuwhof^2^, Judith Bons^1^

###### ^1^Central Diagnostic Laboratory, MUMC+, Maastricht, the Netherlands; ^2^Internal Medicine, MUMC+, Maastricht, the Netherlands

**Correspondence:** Douwe De Boer - douwe.de.boer@mumc.nl

*Clinical and Translational Allergy* 2018, **8(Suppl 3)**:P60

**Background**

Non-specific binding of IgE in in vitro IgE allergy tests contribute to false-positive results. For ImmunoCAP it is stated in the Directions-for-Use that very low levels of allergen specific IgE (IgE_spec_) for all allergens should be evaluated with caution when total IgE (IgE_tot_) > 1000 kU/L. For β-lactams this warning limit is when total IgE (IgE_tot_) > 500 kU/L. Such a notice implies to measure IgE_tot_ for an interpretation of analytical results of at least ImmunoCAP β-lactams IgE_spec_. Goal of this study is to clarify how the interpretation of analytical results of ImmunoCAP β-lactams IgE_spec_ should be done.

**Methods**

Relationship between IgE_tot_ and IgE_spec_ was investigated for ImmunoCAP (Thermo Fisher) IgE_spec_ penicilloyl G and V as well as for amoxilloyl, ampicilloyl and cefaclor. All tests were performed according the manufacturers’ instructions. A variety of serum samples with a wide range of IgE_tot_ concentrations was selected from patients without any known history of β-lactam hypersensitivity. An iterative polynomial regression procedure, which excluded outliers when those after fitting were outside the 95% confidence interval, was applied to check for the nature of a relationship.

**Results**

For ImmunoCAP penicilloyl G (r^2^ = 0.9109; n = 43) and V (r^2^ = 0.8716; n = 46), amoxilloyl (r^2^ = 0.9378; n = 32) and ampicilloyl (r^2^ = 0.9077; n = 32) strong second order polynomial relations were observed, while for cefaclor (r^2^ = 0.9078; n = 31) a strong first order polynomial relation was observed. For IgE_tot_ > 500 kU/L most of the IgE_spec_ values were > 0.10 kU/L and with increasing IgE_tot_ the number of IgE_spec_ values > 0.35 kU/L increased exponentially up to 2.2 kU/L.

**Conclusion**

ImmunoCAP is based on high-capacity binding cellulose and at relatively high concentrations of IgE_tot_, the frequency of possible false-positive results for β-lactams IgE_spec_. is high. The warning limit of 500 kU/L of IgE_tot_ for ImmunoCAP β-lactams IgE_spec_ is valid and above the limit false-positive results are very likely. Consequently, for the respective tests we need to measure always IgE_tot_ for any IgE_spec_ result > 0.35 kU/L.

#### P61 A case of severe anaphylaxis to octenidine: in vitro cross-reactivity to antiseptics containing hexamethylene residues

##### Nicole Müller-Wirth^1^, Werner J. Pichler^1^, Oliver Hausmann^2^

###### ^1^Adverse Drug Reactions- Analysis and Consulting (ADR-AC) GmbH, Bern, Switzerland; ^2^Löwenpraxis & ADR-AC GmbH, Lucerne & Bern, Switzerland

**Correspondence:** Nicole Müller-Wirth - nicole.wirth@adr-ac.ch

*Clinical and Translational Allergy* 2018, **8(Suppl 3)**:P61

**Background**

Anaphylaxis to antiseptics is a rare complication in chronic wound care. Most of the cases are due to a chlorhexidine allergy with single cases reacting to polyhexanide. So far, no anaphylactic reaction to the antiseptic octenidine has been reported.

**Case report**

We present a case of a 87-year old woman suffering from repeated severe anaphylaxis requiring hospitalization after wound care with lidocain as well as an octenidine containing wound gel. During the acute phase, tryptase levels rose to max. 54.7 mcg/l (n < 11.4) with an elevated baseline tryptase of 16.2 mcg/l in the resting phase, on the basis of a mild renal impairment (creatinine clearance of 40–50 ml/min/1.73 m^2^). Skin prick test and basophil activation test (BAT) were positive to octenidine. Indirect BAT using donor basophils re-sensitized with the patient’s serum proved the IgE dependency of the reaction. Additional testing with structurally related compounds showed positivity to chlorhexidine, alexidine and polyhexanide as well. The broad cross-reactivity as well as the negative result for metformin (containing biguanide motives but no hexamethylene structure) points towards a hexamethlyene specificity of the involved IgE.

**Conclusion**

To our knowledge, it is the first report of an anaphylactic reaction to octenidine, based on a reactivity to the hexamethylene motif explaining the broad cross-reactivity to topical antiseptics. Our case report shows that not only the classical biguanide structure of chlorhexidine can be a sensitizer. Octenidine as well as polyhexanide might be emerging allergens and structurally unrelated topical antiseptics are important as safe alternatives.

**Consent to publish**

Consent to publish was obtained from the patients involved in this study.

#### P62 Homemade in-vitro IgE and IgG4 tests specific for carboplatin hypersensitivity reactions

##### Douwe De Boer^1^, Marco Maenen^1^, Paul Menheere^1^, Chris Nieuwhof^2^, Judith Bons^1^

###### ^1^Central Diagostic Laboratory, MUMC+, Maastricht, the Netherlands; ^2^Internal Medicine, MUMC+, Maastricht, the Netherlands

**Correspondence:** Douwe De Boer - douwe.de.boer@mumc.nl

*Clinical and Translational Allergy* 2018, **8(Suppl 3)**:P62

**Background**

Hypersensitivity reactions (HSRs) to platinum-based chemotherapies are an on-going and common problem, limiting optimal therapy when HSRs are present. Risk-stratification protocols using in vivo skin tests and desensitization protocols may enable patients with such HSRs to still receive first-line chemotherapy treatment relatively safely. However, allergy skin testing is invasive and has limited clinical specificity and sensitivity. In order to develop a novel risk-stratification protocol, we designed a homemade in vitro specific IgE (sIgE) and IgG4 (sIgG4) test for carboplatin HSRs using serum samples. We validated the sIgE test using 3 different groups of patients and evaluated during chemotherapy the biomarkers in sera of two patients with carboplatin-induced HSRs.

**Methods**

Carboplatin was conjugated to biotinylated human albumin (HSA) or transferrin (HST). Both haptens were bound separately to streptavidin ImmunoCAP sponges and used to measure sIgE and sIgG4 according the ImmunoCAP principle on the Phadia 250 platform. Serum samples of patients were divided into three groups: (A) exposed with clinical symptoms [n = 2]; (B) exposed without clinical symptoms [n = 9]; (C) negative controls [n = 25]. In each group total IgE and sIgE were measured, while in the exposed groups, sIgG4 were determined additionally.

**Results**

Based on sIgE measurements in group C, the analytical cut-off setting for both haptens was set at 0.11 kU/L for sIgE against carboplatin. No sIgE against both the haptens could be detected in group B above the cut-off. Nevertheless, in group C sIgE could be detected up to 2 kU/L using HSA if the total IgE was above 1000 kU/L. Serum samples around HSRs of the patients in group A demonstrated measurable IgEs against at least one of the haptens (patient #1: 6.9 kU/L using HSA and 4.4 kU/L using HST; patient #2: < 0.11 kU/L using HSA and 0.15 kU/L using HST). The presence of sIgG4 could not be proven in group B except for one patient. Patient #2 of group A demonstrated prior to HSRs presence of relatively elevated sIgG4 at 0.74 mg/L using HSA (patient #1: mean 0.15 mg/L and patient #2: mean 0.55 mg/L).

**Conclusion**

The homemade in vitro test was successfully developed using carboplatin-HSA and carboplatin-HST. sIgE and sIgG4 could be determined in patients who were exposed to carboplatin and suffered from HSRs. In general sIgE and sIgG4 could not be detected in patients who were exposed and did not suffer HSRs. The approach looks promising and should be evaluated in groups with larger sample sizes.

## Thursday 19 April 2018

### Diagnosis and pathophysiology - Poster Walk 7

#### P63 Basophil activation test in ondansetron allergy

##### Bárbara Kong Cardoso, Sofia Martins Farinha, Elza Tomaz, Filipe Inácio, Sara Correia

###### Centro Hospitalar de Setúbal, Setúbal, Portugal

**Correspondence:** Bárbara Kong Cardoso - barbarakc@gmail.com

*Clinical and Translational Allergy* 2018, **8(Suppl 3)**:P63

**Background**

Ondansetron Hydrochloride is a selective serotonin (5-HT3) receptor antagonist used as an antiemetic drug, being generally well tolerated. Hypersensitivity reactions to ondansetron have been reported in either perioperative or emetogenic chemotherapy settings. The wider use of the drug for example in Emergency Pediatric Department, may increase the frequency of reactions. Skin tests are positive in most reported allergic patients and the maximal non-irritating concentration was defined. However for patients with contraindicated or negative skin tests other diagnostic methods would be useful. The Basophil Activation Test (BAT) has been studied for the past decade and showed to be useful particularly in the study of allergy to some antibiotics, neuromuscular blockers and pyrazolones. Our aim was to evaluate BAT as a tool to study ondansetron allergy.

**Methods**

This study involved 4 patients referred to our clinic for suspected ondansetron induced immediate allergic reaction, in a 2 years period.

Skin prick tests (SPT) with undiluted concentration and intradermal test (IDT) with Ondansetron at 2, 0.2 and 0.02 mg/m were performed. BAT was also executed in every case.

**Results**

Data concerning patient characterization and allergy workup results are shown in Table 1. Two patients were from pediatric ages. BAT was positive in two cases, one of them a child with negative skin tests.

**Table 1:** Results

Patient no.	Age	Gender	Reaction	SPT	BAT
1	6	M	Urticaria	Neg	Pos
2	13	F	Anaphylaxis	Pos (IDT 0.02 mg/ml)	Neg
3	37	M	Anaphylaxis	Pos (IDT 0.2 mg/mL)	Pos
4	50	F	Urticaria	Pos(IDT 0.02 mg/ml)	Neg

*M* male, *F* female, *Pos* positive, *Neg* negative, *SPT* skin prick tests, *IDT* intradermal test

**Conclusion**

Our results highlight the value of the BAT in the ondansetron allergy workup. BAT may be a useful tool to support the final diagnosis particularly in patients with life-threatening reactions.

#### P65 Covalent Anchoring of b-lactam determinants on digital surfaces for developing in vitro tests for the diagnosis of allergy

##### Sergi Morais, Mª José Juarez, Edurne Peña-Mendizabal, Salvador Mas-García, Luis Tortajada-Genaro, Ángel Maquieira

###### Instituto Interuniversitario de Investigación de Reconocimiento Molecular y Desarrollo Tecnológico, (IDM), Universitat Politècnica de València, Camino de Vera s/n, 46022, Valencia, Spain

**Correspondence:** Luis Tortajada-Genaro - luitorge@upv.es

*Clinical and Translational Allergy* 2018, **8(Suppl 3)**:P65

**Background**

The in vitro detection of allergy to β-lactam antibiotics (BLCs) requires analytical methodologies to determine accurately the concentration of specific IgEs with high sensitivity. Currently, the antigenic determinants are anchored to mono or polydisperse polymers or proteins that are embedded in hydrogels with which the test is performed. The tests are carried out in expensive bulky autoanalyzers that analyze only 5 BLCs, one by one, and display low sensitivity and false negative and positive results. This is probably because the tests use determinants that are not specifically recognized by the IgEs. Also, the differences between IgEs of different patients make very difficult to have a constant response. Moreover, validation of the tests is a challenging task to perform. For all these reasons, the development of reliable, portable and cost-effective in vitro test is a highly demanded solution for primary and secondary care. The development of i*n vitro* methods require multidisciplinary approaches that take into account the chemical structure of the determinant, the way how the determinants are anchored, IgE exposition and the patient immune response.

**Methods**

A mass consumer electronic DVD system, offering advantages to serve as basis of health devices for diagnosis of allergy, is presented. The system incorporates a disc drive as a portable optical detector, and digital versatile compact discs on which a set of determinants in microarray format are covalently immobilized. The anchoring is carried out through different reactive sites of the BLCs, following the carbodiimide and carbohydrazide chemistry. The performances of the assay are evaluated, using artificial human sera (ARTHUS) as control.

**Results**

The multiplex covalent anchoring of the β-lactam derivatives directly onto the analytical surface of a compact disc is key to delineate the selectivity and sensitivity of the assays by increasing and facilitating the repertoire and exposition of IgE epitopes.

**Conclusion**

The centrifugal system on disc is a striking and promising multiplex alternative easily transferable into clinical practice for the rapid diagnosis of allergy to all BLCs families in a selective and sensitive manner because of its cost efficiency, scalability to different scenarios, minimal sample volume and reagent consumption.

Acknowledgments: This work has been funded by the H2020 program (Project COBIOPHAD, Grant Agreement No. 688448), being an initiative of the Photonics Public Private Partnership (www.photonics21.org).

#### P66 Massive expansion of drug-specific, clonotypic and polycytotoxic CD8+ T cells in toxic epidermal necrolysis

##### Axel Patrice Villani^1^, Aurore Rozières^1^, Benoit Bensaid^1^, Klara Eriksson^2^, Mathilde Tardieu^1^, Floriane Albert^1^, Virginie Mutez^1^, Tugba Baysal^1^, Denis Jullien^1^, Catherine Giannoli^3^, Marc Pallardy^4^, Janet Maryanski^5^, Jean-François Nicolas^1^, Osami Kanagawa^1^, Daniel Yerly^2^, Marc Vocanson^1^

###### ^1^INSERM U1111-CIRI, Lyon, France; ^2^Drug Allergy Research Laboratory, Inselspital Bern, Bern, Switzerland; ^3^HLA/HPA laboratory - EFS Lyon-Gerland, Lyon, France; ^4^INSERM UMR 996, Châtenay-Mallabry, France; ^5^Sophia Antipolis University, Nice, France

**Correspondence:** Axel Patrice Villani - axel.villani@gmail.com

*Clinical and Translational Allergy* 2018, **8(Suppl 3)**:P66

**Background**

Toxic epidermal necrolysis (TEN) is a life-threatening and blistering adverse drug reaction, characterized by massive epidermal necrosis. Diverse studies have reported that the onset of TEN correlates with skin infiltration by cytotoxic lymphocytes (T [CTL], NK cells) and inflammatory monocytes.

**Methods**

To further characterize the phenotype of skin-infiltrating lymphocytes at the acute phase of TEN, we conducted a prospective study on the blood and blister fluids from 16 TEN patients, using flow and mass cytometry, as well as next generation TCR sequencing. Samples collected in patients suffering from the frequent and benign maculopapular exanthema (MPE) were also used as controls (MPE, n = 15).

**Results**

Our results confirm that conventional CD8+ T cells (CD45+TCRab+CD8b+), and at a lesser extend of CD4+ T cells, were the main leucocyte subsets found in TEN blisters. Consequently, the CD4/CD8 ratio was inversed in blisters (mean: 0.8) compared to blood (mean: 2). However, we failed to repeatedly detect NK (CD45+ TCRab-CD56+), NKT cells (CD45+ TCRab^int^TCRVa24+) or MAIT cells (CD45+ TCRVa7.2 + CD8b-CD8a+) in TEN blisters. Strikingly, deep sequencing of the T cell receptor revealed massive expansion of certain CTL clones both in the skin and the blood of TEN patients, conversely to MPE patients, and which were confirmed at TCR-Vb usage level by flow cytometry. Over-represented blister clones were mainly effector memory TCRab+CD8b+CD45RA-CCR7-CD27+ T cells. They expressed elevated levels of markers of inflammatory activation and displayed a poly-cytotoxic phenotype since they co-expressed Granulysin, Granzyme B, Granzyme A, Perforin and TWEAK, as demonstrated by unsupervised mass cytometry analysis. By comparison, the TCR repertoire bias and poly-cytotoxic phenotype were far less marked in the cells extracted from the inflamed skin of MPE patients. By transfecting α and β chains of the expanded clonotypes into Jurkat and SKW3 immortalized cells, we were able to confirm that they were drug-specific. Finally, we analyzed the TCR repertoire diversity and showed that clonality was significantly more elevated in the skin of TEN patients compared to the skin of MPE patients (p = 0.02).

**Conclusion**

Our results highlight a massive expansion of clonotypic and poly-cytotoxic CD8+ T cells in the blisters of TEN patients, which could explain the severity of this life-threatening disease. Deep phenotype analysis and next generation TCR sequencing unveils new potential treatment targets and severity biomarkers.

#### P67 Analysis of peripheral blood mononuclear cell subtypes in drug hypersensitivity reactions

##### Eszter Jakobicz^1^, Zsuzsanna Palotás^1^, István Balázs Németh^1^, Katinka Ónodi-Nagy^1^, Lajos Kemény^2^, Zsuzsanna Bata-Csörgo^2^

###### ^1^University of Szeged, Department of Dermatology and Allergology, Szeged, Hungary; ^2^University of Szeged, Department of Dermatology and Allergology; MTA-SZTE Dermatological Research Group, Szeged, Hungary

**Correspondence:** Eszter Jakobicz - eszter.jakobicz@gmail.com

*Clinical and Translational Allergy* 2018, **8(Suppl 3)**:P67

**Background**

Drug hypersensitivity reactions can present with various skin manifestations. The role of the skin infiltrating immune cells in the pathogenesis is well characterized, but literature data is conflicting about their distribution in the peripheral blood. In our present study we looked for patterns in the distribution of different subtypes of peripheral blood mononuclear cells (PBMCs) in different manifestations of drug hypersensitivity reactions.

**Methods**

Forty-five patients with the following acute drug hypersensitivity symptoms were enrolled in the study: urticaria (9), drug-induced vasculitis (3), maculopapular exanthema (17), erythema multiforme (4), Stevens–Johnson syndrome (3), DRESS (3), fixed drug exanthema (3), and EBV-associated drug rash (3). Using flow cytometry we analyzed the distribution of different subtypes of freshly separated PBMCs, we compared the patients’ results to healthy controls. For labeling the different subsets of cells the following markers were used: CD2, CD3, CD4, CD8, CD19 and CD56.

**Results**

The analyses of PBMC subtypes in patients with acute drug hypersensitivity symptoms showed a significant decrease in the proportion of the CD3^+^CD4^+^ T lymphocytes in Stevens–Johnson syndrome, and a significant decrease in the proportion of CD3^+^CD8^+^ T lymphocytes in maculopapular exanthema, fixed drug exanthema, and DRESS syndrome, the latter accompanied by a decrease in the proportion of CD19^+^ B lymphocytes. In EBV-associated drug rash, we found a lower proportion of CD19^+^ B lymphocytes and CD3^+^CD4^+^ T lymphocytes; and a higher proportion of CD3^+^CD8^+^ T lymphocytes; while a lower proportion of CD56^+^ NK cells and a higher proportion of CD3^+^CD4^+^ T lymphocytes could be detected in erythema multiforme. In the two antibody-mediated drug rashes—urticaria and drug-induced vasculitis—none of the PBMC subtypes’ proportion differed from the healthy controls.

**Conclusion**

The decrease in different PBMC subtypes’ proportion in the circulation during the acute drug hypersensitivity reaction could be the result of immune cell migration to the skin. With increased patient number, this type of flow cytometric analysis of peripheral blood may prove to be of clinical use.

#### P68 Discovery of five novel amino acid variants within HLA-DRB1 associated with Dapsone hypersensitivity syndrome in addition to HLA-B*13:01

##### Yonghu Sun, Zhenhua Yue, Hong Liu, Furen Zhang

###### ^1^Shandong Provincial Institute of Dermatology and Venereology, Jinan, China

**Correspondence:** Yonghu Sun - suohandong@126.com

*Clinical and Translational Allergy* 2018, **8(Suppl 3)**:P68

**Background**

Dapsone hypersensitivity syndrome is a rare yet severe adverse drug reaction caused by dapsone, a principal drug in the multidrug therapy for leprosy. *HLA*-B*13:01 has been identified to be a strong risk factor of dapsone hypersensitivity syndrome, however its low positive predictive value indicated that additional genetic variants may involve in the disease development.

**Methods**

To answer the questions about the low positve predictive value, we performed a high coverage next-generation sequencing based *HLA* typing analysis in 103 dapsone-hypersensitive and 857 dapsone-tolerant *HLA*-B*13:01 positive leprosy patients in Chinese population.

**Results**

We firstly identified none of 11 subtypes of *HLA*-B*13:01 at 6-digit resolution were present in either DHS cases or DDS-tolerant controls except HLA-B*13:01:01. Secondly, five amino acid variants in high linkage disequilibrium of HLA-DRB1 were significantly associated with dapsone hypersensitivity syndrome (positions 133, 142, − 17, 11, and 13). DRB1*16:02 and DRB1*15:01 tagged by these risk-conferring amino acid residues were associated at a nominal significance level.

**Conclusion**

This study identified five amino acid variants within HLA-DRB1 that are in high linkage disequilibrium and significantly associated with dapsone hypersensitivity syndrome in addition to HLA-B*1301 in a Chinese population and greatly improve the knowledge of DHS.

#### P69 NMBA-specific memory T cell quantification by CD154 expression in anaphylaxis diagnosis

##### Stéphane Baillard^1^, Fatma Sahli^2^, Sylvain Petit^2^, NIcolas Gigant^2^, Aurélie Gouel-Chéron^3^, Benoît Noël^1^, Sandy Peltier^4^, Catherine Neukirch^5^, Rami Bechara^1^, Pierre Bruhns^6^, Friederike Jönsson^6^, Dan Longrois^3^, Florence Tubach^7^, Marc Pallardy^1^, Delphine Joseph^2^, Sylvie Chollet-Martin^4^, Luc De Chaisemartin^4^

###### ^1^INSERM UMR 996, Univ Paris-Sud, Université Paris-Saclay, Châtenay-Malabry, France; ^2^UMR CNRS 8076, Univ Paris-Sud, Université Paris-Saclay, Châtenay-Malabry, France; ^3^APHP, Hôpital Bichat, Département d’Anesthésie-Réanimation, HUPNVS, Paris, France; ^4^APHP, Hôpital Bichat, UF Auto-immunité et Hypersensibilités, HUPNVS, Paris, France; ^5^APHP, Hôpital Bichat, Service de Pneumologie A, HUPNVS, Paris, France; ^6^Institut Pasteur, Department of Immunology, Unit of Antibodies in Therapy and Pathology, Paris, France; ^7^INSERM, ECEVE, U1123, CIC 1421, Paris, France

**Correspondence:** Luc De Chaisemartin - luc.de-chaisemartin@aphp.fr

*Clinical and Translational Allergy* 2018, **8(Suppl 3)**:P69

**Background**

Perioperative anaphylaxis is an acute systemic hypersensitivity reaction occurring during general anesthesia and frequently triggered by neuromuscular blocking agents (NMBA). It is considered to rely on specific IgE antibodies against an allergen and histamine release by mast cells and basophils. However, data from animal models and clinical study results (NASA study) suggest an alternative pathway dependent on specific IgG antibodies and involving platelet-activating factor. Both IgE and IgG pathways imply the existence of NMBA-specific T cells that are necessary for isotype switch and antibody production. Drug-specific T cells have been well documented mostly in antibiotic allergy, but definitive evidence of NMBA-specific memory T cells as well as data on their clinical relevance is lacking.

**Methods**

26 patients with a suspicion of NMBA-induced anaphylaxis from 10 French anesthesia departments were prospectively included and their PBMC isolated during an allergology visit (NASA study). 20 healthy blood donors were used as controls. As the original antigen driving sensitization is not known, we constructed and characterized by MALDI-TOF bioconjugates of NMBA (suxamethonium) with human serum albumin. PBMC were cultured for 48 h with these bioconjugates and a cytokine cocktail to differentiate and activate antigen-presenting cells. After 48 h, T cells activation was quantified by CD154 expression (flow cytometry) and cytokine production (INF-g, IL-5, IL-13) was measured by Fluorospot.

**Results**

CD154+ T cells could be detected at higher levels in patients as compared to controls (p < 0.0001). ROC curve analysis of CD154 levels showed an area at 0.98 with a sensitivity of 96% and specificity of 98% at optimal cut-off. Cytokine production analysis showed mainly INFg-producing T cells. There were no link between memory T cells levels and anaphylaxis severity or injected NMBA. Interestingly, memory T cells could be detected in 8/9 patients with negative NMBA skin tests, of which 4 had a severe reaction.

**Conclusion**

We characterize for the first time INF-g producing NMBA-specific T cells and show they could represent an interesting additional diagnostic tool in perioperative anaphylaxis particularly when usual tests are negative.

#### P70 The potential and limitations of molecular docking to elucidate the association of HLA alleles with adverse drug reactions

##### Kerry Anne Ramsbottom^1^, Dan Carr^2^, Andrew R Jones^1^, Daniel J Rigden^1^

###### ^1^Institute of Integrative Biology, University of Liverpool, Liverpool, United Kingdom; ^2^Institute of Translational Medicine, University of Liverpool, Liverpool, United Kingdom

**Correspondence:** Kerry Anne Ramsbottom - K.A.Ramsbottom@liverpool.ac.uk

*Clinical and Translational Allergy* 2018, **8(Suppl 3)**:P70

**Background**

Adverse drug reactions (ADRs) have been linked with HLA in numerous different studies whereby individuals carrying particular alleles of HLA genes are at higher risk of developing ADRs to particular drugs. The role of HLA in these adverse reactions have been hypothesised in three main ways; the Hapten model, the Pharmacological Interaction model and the Altered Peptide Repertoire model. The current most strongly associated ADR is that of Abacavir with HLA-B*57:01. This drug–HLA interaction has been shown to follow the Altered Peptide Repertoire model. There is therefore growing interest in searching for evidence supporting this model for other ADRs using bioinformatics techniques, including molecular docking.

**Methods**

In silico docking was used to assess the utility and reliability of well-known docking programs when addressing challenging HLA–drug interactions. The crystal structure of Abacavir bound in complex with B*57:01 was used as a benchmark. Four docking programs; SwissDock, Rosetta Online Server that Includes Everyone (ROSIE), AutoDock Vina and AutoDockFR, were used to investigate if each software could accurately dock the Abacavir back into the risk allele crystal structure and if they were able to distinguish between the HLA-associated and non-associated HLA alleles. The programs that were best able to predict the binding position of Abacavir were then used to recreate the docking of Carbamazepine with B*15:02 seen in previous studies.

**Results**

SwissDock and AutoDockFR were seen to be able to most accurately predict the binding positions of Abacavir with B*57:01. The other programs used, excluding ROSIE, were able to identify the correct sub-pocket of binding and distinguish between the risk and control alleles. SwissDock and AutoDockFR were also able to recreate previously published predictions for Carbamazepine with B*15:02 and could correctly distinguish between risk and control alleles.

**Conclusion**

The purpose of this exercise was to compare multiple docking programs to assess their performance with these challenging HLA-ADR cases. It was found that the binding mode could not always be predicted but the programs used were mostly able to identify the correct sub-pocket and distinguish between risk and control alleles. Furthering our understanding of the potentials and limitations of docking small molecules to HLA is important to aid understanding of the underlying mechanisms. Identifying how the binding varies between risk and control alleles may enable us to make predictions of potential ADRs and identify polymorphisms contributing to direct binding.

#### P71 Development and optimization of assays to predict the intrinsic immunogenicity of drugs

##### Joel Watkinson, Lee Faulkner, Andrew Gibson, B. Kevin Park, Dean J. Naisbitt

###### Department of Molecular & Clinical Pharmacology, MRC Centre for Drug Safety Science, University of Liverpool, Liverpool, United Kingdom

**Correspondence:** Andrew Gibson - a.gibson@liv.ac.uk

*Clinical and Translational Allergy* 2018, **8(Suppl 3)**:P71

**Background**

For T cell mediated delayed-type drug hypersensitivity reactions, assays such as the lymphocyte transformation test are utilised for diagnostic purposes, but an approach that predicts potential immunogenicity of drugs during development would be preferable. Both the patient and Pharma can be aided by the successful prediction of immunogenic drugs at an early stage. The main challenge is to develop and optimize simplified assays that account for and incorporate the multi-factorial nature of drug hypersensitivity, including: HLA associations, immune checkpoint signaling and different forms of drug antigen. Aim: To develop and optimise assays that (1) assess the number of individuals and (2) the number of T cells that respond to a given test compound. To do this, we have developed 2 assays; T-MWA (multiple well assay) and T-MDA (multiple donor assay), utilizing PBMC from an HLA-typed cell bank containing PBMC from > 1000 healthy donors.

**Methods**

*In vitro* naïve T cell priming was performed in a single 96-well plate, where healthy volunteer naïve T cells and autologous DCs were cultured for two weeks with model compounds nitroso sulfamethoxazole (SMX-NO), nitroso Dapsone (DDS-NO), Bandrowski’s Base and piperacillin (T-MDA; 6 wells per donor, T-MWA; 48 wells). Co-cultures were then re-challenged and assessed for antigen specific T cell proliferation and cytokine secretion; using thymidine incorporation, CFSE labelling and flow cytometry, ELISpot and IFN-y ELISA respectively. Responses were determined according to their stimulation index as low (1.5–1.99), moderate (2–3.99) and high (4+) to determine the strength of response.

**Results**

The T-MDA detected SMX-NO specific T cell response in 5/5 donors in a single plate assay. Responses to other compounds were detected in a lower number of donors. The T-MWA provided a more detailed picture of the immunogenicity of the test compounds. Antigen-specific T cell responses to SMX-NO and BB were detected in all donors with 55–70% and 60–100% of individual wells responding, respectively. Piperacillin and DDS-NO activated T-cells from all donors, but responses were detected in fewer drug-treated wells (1–20%). ELISA experiments for cytokine secretion solidified findings; showing similar levels of response as the proliferation assays.

**Conclusion**

We have successfully transformed complex laboratory-based T cell priming assays into simplified methods that may in the future be used to screen the intrinsic immunogenicity of drugs and chemicals. The methods use a traffic light system to identify compounds with a low, moderate or high risk of causing immune reactions when administered widely to patients.

#### P72 Characterization of pathways of dapsone- and nitroso dapsone-specific CD4+ and CD8+ T-cell activation using PBMC from HLA-B*13:01+ hypersensitive patients

##### Qing Zhao^1^, Khetam Alhilali^2^, Abdulaziz Alzahrani^2^, Mubarak Almutairi^2^, Hong Liu^1^, Yonghu Sun^1^, Xiaoli Meng^2^, Andrew Gibson^2^, Monday O. Ogese^3^, B. Kevin Park^2^, David Ostrov^4^, Furen Zhang^1^, Dean J. Naisbitt^2^

###### ^1^Department of Dermatology, Shandong Provincial Hospital for Skin Disease, Shandong University, Jinan, Shandong, China. Shandong Provincial Institute of Dermatology and Venereology, Shandong Academy of Medical Sciences, Jinan, Shandong, China; ^2^Department of Molecular and Clinical Pharmacology, MRC Centre for Drug Safety Science, University of Liverpool, Liverpool, United Kingdom; ^3^Pathology Sciences, Drug Safety and Metabolism, IMED Biotech Unit, AstraZeneca, Cambridge Science Park, Milton Road, Cambridge, United Kingdom; ^4^University of Florida, Gainesville, Florida, USA

**Correspondence:** Monday O Ogese - m.o.ogese@liverpool.ac.uk

*Clinical and Translational Allergy* 2018, **8(Suppl 3)**:P72

**Background**

Approximately 0.5–3.6% of individuals exposed to dapsone (DDS) develop a hypersensitivity syndrome. Recently, a genetic association study identified HLA-B*13:01 as a risk factor of DDS hypersensitivity. Furthermore, we have shown that DDS and its protein reactive metabolite nitroso-dapsone (DDS-NO) activate CD4+ and CD8+ T-cells from healthy volunteers and hypersensitive patients. Despite this, the molecular interaction between DDS and/or DDS-NO and the HLA-B*13:01 peptide complex and whether this is involved in the selective activation of CD8+ T-cells has not been studied.

The objective of this study was to investigate pathways of DDS/DDS-NO-specific T-cell activation and the role of HLA-B*13:01 in the activation of CD8+ T-cells.

**Methods**

DDS- and DDS-NO-specific CD4+ and CD8+ T-cell clones were generated from three hypersensitive patients. Flow cytometry and antibody blocking were used to determine CD phenotype and MHC restriction. Antigen presenting cell (APC) fixation assay and APC pulsing were performed to determine the mechanisms of antigen presentation. Glutathione was added to block DDS-NO protein binding. DDS-NO protein adducts were characterized by mass spectrometry. To determine the role of HLA-B*13:01 allele in the activation of CD8+ T-cells, clones were cultured with or without APC to exclude self-presentation. Next, clones were cultured with autologous or allogeneic APC from 14 donors with varying homologies to HLA-B*13:01 allele and T-cell proliferation evaluated.

**Results**

Activation of DDS- and DDS-NO-specific CD4+ and CD8+ clones (proliferation and cytokine release) was dependent on APC. DDS-NO CD4+ and CD8+ clones responded to soluble drug and APC pulsed with DDS-NO for 0.5, 2, and 4 h. T-cell activation was blocked with glutathione, which prevented the binding of DDS-NO to protein. In contrast, DDS-specific CD4+ and CD8+ clones were not activated with DDS pulsed APC and glutathione did not inhibit DDS-specific responses. Both DDS and DDS-NO activated clones in the presence of glutheraldehyde-fixed APC, suggesting that parent drug and metabolite bind directly to APC in the in vitro assay to activate T-cells. Certain CD8+ clones were only activated with DDS or DDS-NO in the presence of autologous APC, while others were activated with APCs expressing an array of different HLA-B alleles. However, some clones were stimulated to proliferate selectively with DDS or DDS-NO and APCs expressing HLA-B*13:01 and closely related alleles.

**Conclusion**

These data show that DDS and DDS-NO activate T-cells through reversible and irreversible binding interactions with APCS, respectively. Furthermore, several CD8+ clones are activated with the drug (metabolite) bound selectively to APC expressing HLA-B*13:01.

## Thursday 19 April 2018

### Diagnosis and pathophysiology - Poster Walk 8

#### P73 Docking model of dapsone, phenobarbital, and phenytoin bound to HLA-B*13:01 explains the risk of drug-induced hypersensitivity syndrome

##### Hideaki Watanabe^1^, Yasuya Tashiro^1^, Hanako Nakamura^1^, Haruka Ando^1^, Ran Ono^1^, Hirohiko Sueki^1^, Yoshio Kusakabe^2^

###### ^1^Department of Dermatology, Showa University School of Medicine, Tokyo, Japan; ^2^Laboratory of Chemistry, Faculty of Pharma Sciences, Teikyo University, Tokyo, Japan

**Correspondence:** Hideaki Watanabe - hwatanabe@med.showa-u.ac.jp

*Clinical and Translational Allergy* 2018, **8(Suppl 3)**:P73

**Background**

Recently, human leukocyte antigen (HLA)-B*13:01 was identified as a marker of susceptibility to drug-induced hypersensitivity syndrome (DIHS) caused by dapsone, phenobarbital, and phenytoin. To investigate why HLA-B*13:01 is responsible for DIHS from a structural point of view.

**Methods**

First, we used homology modeling to derive the three-dimensional structures of HLA-B*13:01 (associated with DIHS) and HLA-B*13:02 (not so associated despite strong sequence identity [99%] with HLA-B*13:01). Next, we used molecular docking, molecular dynamic simulations, and the molecular mechanics Poisson–Boltzman surface area method, to investigate the interactions of dapsone, phenobarbital, and phenytoin with HLA-B*13:01 and 13:02.

**Results**

We found a crucial structural difference between HLA-B*13:01 and 13:02 in the F-pocket of the antigen-binding site. As Trp95 in the α-domain of HLA-B*13:02 is replaced with the less bulky Ile95 in HLA-B*13:01, we found an additional well-defined sub-pocket within the antigen-binding site of HLA-B*13:01. Docking poses of dapsone, phenobarbital and phenytoin against the antigen-binding site of HLA-B*13:01 used this unique sub-pocket, indicating its suitability for binding these three drugs. However, HLA-B*13:02 does not seem to possess a binding pocket suitable for binding drugs. Moreover, these three drugs have a common chemical structure, which might be easy to combine with the sub-pocket of HLA-B*13:01.

**Conclusion**

Our computational results suggest that dapsone, phenobarbital, and phenytoin would fit within the structure of the antigen-recognition site of HLA-B*13:01. Moreover, these drugs have a common chemical structure, and this structure is presumed to be a responsible for easy combine with sub-pockets of HLA-B*13:01.

#### P74 Tissue influx of neutrophils and monocytes is delayed during development of trovafloxacin-induced tumor necrosis factor-dependent liver injury in mice

##### Giulio Giustarini^1^, Richard Weaver^2^, Marianne Bol Schoenmakers^1^, Joost Smit^1^, Raymond Pieters^1^

###### ^1^IRAS Utrecht University, Utrecht, the Netherlands; ^2^Servier Group, Paris, France

**Correspondence:** Raymond Pieters - r.h.h.pieters@uu.nl

*Clinical and Translational Allergy* 2018, **8(Suppl 3)**:P74

**Background**

Idiosyncratic drug-induced liver injury (iDILI) has a poorly understood pathogenesis. However, iDILI is often associated with inflammatory stress signals in human patients as well as animal models. Tumor necrosis factor (TNF) and neutrophils play a key role in onset of trovafloxacin (TVX)-induced iDILI, but the exact role of neutrophils and other leukocytes remains to be defined.We therefore set out to study the kinetics of immunological changes during the development of TVX-induced iDILI in the established murine model of acute liver injury induced by administration of TVX and TNF.

**Methods**

C57BL6 mice were fasted and exposed to TVX, levolfoxacin of saline at t = − 3 h, and at t = 0 h TNF was injected. Next, kinetics (at t = 0, 2 and 4 h after TNF injection) of changes in liver, spleen and blood were evaluated for presence of innate immune cells (flowcytometry), cytokines (ELISA, PCR) and pathology.

**Results**

Initially, TNF stimulated the appearance of leukocytes, in particular neutrophils, into the liver of TVX-treated mice, but even more so in control mice treated with the non-DILI inducing analogue levofloxacin (LVX) or saline as vehicle (Veh). This difference was apparent at 2 h after TNF administration, but at 4 h, the relative neutrophil amounts were reduced again in Veh- and LVX-treated mice whereas the amounts in TVX-treated mice remained at the same increased level as at 2 h. The influx of monocytes/macrophages, which was unaffected in Veh- and LVX-treated mice was markedly reduced or even absent in TVX-treated mice. Unlike controls, mice receiving TVX+ TNF display severe hepatotoxicity with clear pathology and apoptosis, coagulated hepatic vessels and increased alanine aminotransferase levels and interleukin 6/10 ratios.

**Conclusion**

Findings indicate that TVX delays the acute influx of neutrophils and monocytes/macrophages. Considering their known anti-inflammatory functions, the disruption of influx of these innate immune cells may hamper the resolution of initial cytotoxic effects of TVX and thus contribute to liver injury development.

#### P75 Genetic variants in COX-1 and COX-2 encoding genes in nonsteroidal anti-inflammatory drug-induced urticaria/angioedema

##### Jurado-Escobar Raquel^1^, Inmaculada Doña^2^, James Richard Perkins^1^, Gador Bogas^2^, Natalia Pérez-Sánchez^2^, Joan Bartra^3^, José Augusto Agúndez^4^, María Isidoro-García^5^, Cristobalina Mayorga^1^, María José Torres^2^, José Antonio Cornejo-García^1^

###### ^1^Research Laboratory, IBIMA, Regional University Hospital of Malaga, UMA, Malaga, Spain; ^2^Allergy Unit, IBIMA, Regional University Hospital of Malaga, UMA, Malaga, Spain; ^3^Unitat d´Allergia, Servei de Pneumologia, Hospital Clinic, Universitat de Barcelona, Barcelona, Spain; ^4^Department of Pharmacology, University of Extremadura, Cáceres, Spain; ^5^Department of Clinical Biochemistry, Pharmacogenetics Unit, University Hospital of Salamanca, Salamanca, Spain

**Correspondence:** José Antonio Cornejo-García - josea.cornejo@gmail.com

*Clinical and Translational Allergy* 2018, **8(Suppl 3)**:P75

**Background**

Nonsteroidal anti-inflammatory drugs (NSAIDs), among the most highly consumed medicines worldwide, are the main triggers of drug hypersensitivity reactions (DHRs). At least five different phenotypes caused by DHRs to NSAIDs are recognised by EAACI, including NSAIDs-exacerbated respiratory disease (NERD), in patients with underlying rhinitis and/or asthma with or without nasal polyposis, and NSAIDs-induced acute urticaria/angioedema (NIUA) in otherwise healthy individuals. According to the cyclooxygenase-1 (COX-1) hypothesis, COX-1 inhibition shunts the arachidonic acid metabolism towards the synthesis of cysteinil-leukotrienes, which in turn elicit a hypersensitivity reaction in susceptible individuals. This susceptibility is thought to be influenced by genetic factors, which have mainly been analysed in NERD patients, in spite of NIUA being the most frequent clinical entity induced by DHRs. The aim of this study was to evaluate the overall genetic variability in the COX-1 encoding gene *PTGS1* (prostaglandin-endoperoxide synthase 1) and its inducible isozyme, the COX-2 encoding gene, *PTGS2* (prostaglandin-endoperoxide synthase 2) in NIUA patients.

**Methods**

A total of 269 NIUA patients and 300 healthy controls with no significant age and sex differences were included. We selected a total of 12 tagging single nucleotide polymorphisms (tSNPs) in *PTGS1* and 9 in prostaglandin-endoperoxide synthase 2 *PTGS2*, using European populations data available from the 1000 Genomes Project. Genotyping was perfomed using the iPlex Sequenom MassArray technology.

**Results**

Two tSNPs in *PTGS1* (rs10306194 and rs1330344) were found to be statistically associated with NIUA after Bonferroni multiple testing correction (corrected p-values of 0.014 and 0.019, respectively). In addition, two other variants in *PTGS1* (rs3119773 and rs76942325) and one in *PTGS2* (rs689467) were marginally associated with NIUA.

**Conclusion**

Our results suggest a role for UTR COX-1 genetic variants in NIUA, the most frequent entity induced by DHRs, possibly by affecting gene expression Further studies are required to replicate these associations, to evaluate the potential participation of COX-1 variability in other entities induced by DHR to NSAIDs and to shed light on the molecular basis underlying these associations.

#### P76 Amoxicillin-peptide adducts activate T-cells from DILI patients in a HLA DRB1*15:01-DQB1*06:02 risk haplotype restricted manner

##### Arun Tailor^1^, John Farrell^1^, James Waddington^1^, Gordon Dear^2^, B. Kevin Park^1^, Xiaoli Meng^1^, Dean J. Naisbitt^1^

###### ^1^Department of Molecular & Clinical Pharmacology, MRC Centre for Drug Safety Science, University of Liverpool, Liverpool, United Kingdom; ^2^GlaxoSmithKline, Park Road, Hertfordshire, Ware, United Kingdom

**Correspondence:** Arun Tailor - a.gibson@liv.ac.uk

*Clinical and Translational Allergy* 2018, **8(Suppl 3)**:P76

**Background**

Exposure to the antibiotic amoxicillin (AX) is associated with the development of several adverse drug reactions including liver and skin injury. AX-specific T-cells have been detected in patients presenting with both liver and skin reactions, suggestive of an immune disease aetiology. These reactions do not occur in every drug administered patient therefore their origin may be attributed to the individual biology of a patient. This theory has gained credence with the discovery of a number genetic associations including the HLA DRB1*15:01-DQB1*06:02 haplotype. β-lactam antibiotics form drug-protein adducts by conjugating with lysine residues that are postulated to activate T-cells, following protein processing and the liberation of peptide epitopes. Several AX-modified proteins have been found including AX conjugated with human serum albumin. However, as yet, a role for such adducts in the activation of T-cells has not been described. This study aimed to investigate the role of AX-peptide adducts in the activation of a drug-specific T-cell response.

**Methods**

Lysine containing peptides carrying anchors for the HLA DRB1*15:01-DQB1*06:02 haplotype were designed using the SYFPEITHI database. Positional derivatives containing the same anchors with lysine in different TCR contact sites were also generated. Peptides were incubated with AX at a 1:10 molar ratio for 24 h and the AX-modified peptide was purified using reverse phase chromatography.

**Results**

Successful peptide modification was confirmed using the TripleTOF 5600 mass spectrometer (AB Sciex). AX-Peptides were incubated with PBMCs from hypersensitive patients carrying the risk haplotype for 2 weeks to generate antigen-responsive T-cell lines. Subsequently, T-cells were cloned by serial dilution and repetitive mitogen stimulation. Well growing clones were tested with AX, AX-modified peptides and unmodified peptides. Clones were expanded and characterized in terms of cellular phenotype and cytokine release. AX-peptide specific clones proliferated and secreted cytokines such as IFN-g in a dose dependent manner with high specificity to the antigen showing no cross reactivity with free drug, with unmodified peptide or with positional derivatives at different TCR contact sites. Clones were also shown to respond in a class II restricted and HLA DRB1*15:01-DQB1*06:02 restricted manner.

**Conclusion**

Here we present a strategy to study T-cell responses to drug-modified peptides in individuals expressing specific HLA alleles associated with immunological drug reactions. Further work on designer drug-modified peptides could help (1) elucidate the principal antigenic signals in drug-induced skin and liver injury and (2) develop reagents to improve diagnosis.

#### P77 Abacavir altered self-peptides can be recognised by CD8+ T-cells, leading to the induction of a hypersensitivity reaction

##### Paul James Thomson^1^, Patricia T. Illing^2^, John Farrell^1^, Mohammad Alhaidari^1^, Nicole A. Mifsud^2^, B. Kevin Park^1^, Dean J. Naisbitt^1^

###### ^1^MRC Centre for Drug Safety Science, Dept Molecular & Clinical Pharmacology, University of Liverpool, Liverpool, UK; ^2^Infection and Immunity Program, Monash Biomedicine Discovery Institute and Dept of Biochemistry and Molecular Biology, Monash University, Clayton 3800, Victoria, Melbourne, Australia

**Correspondence:** Paul James Thomson - p.j.thomson@liverpool.ac.uk

*Clinical and Translational Allergy* 2018, **8(Suppl 3)**:P77

**Background**

Abacavir is a reverse transcriptase inhibitor associated with hypersensitivity reactions in individuals expressing HLA-B*57:01. It has been shown that up to 25% of the peptides bound to HLA-B*57:01 in the presence of abacavir are novel self-peptides not observed in the absence of the drug. Abacavir alters the shape and chemistry of the antigen binding cleft of HLA-B*57:01, leading to the presentation of these novel self-peptides. This shift in peptide presentation is hypothesised to trigger a CD8^+^ T-cell response in hypersensitive patients; however, the peptide sequences that activate T-cells have not been fully defined.

The aim of this study was to investigate whether abacavir induced self-peptides activate or participate in the activation of abacavir-responsive CD8^+^ T-cell clones.

**Methods**

Characterization of the HLA-B*57:01 peptide repertoire in the presence and absence of abacavir identified a series of self-peptides present in abacavir treated cells but not observed in untreated. A selection of abacavir induced self-peptides were used to generate CD8^+^ T-cell clones in the presence and absence of abacavir. The T-cell activity of the clones was measured by means of proliferation and IFN-Ƴ secretion via ^3^H thymidine incorporation and Eli-spot assays, respectively. The effect of the abacavir-induced self-peptides on T-cell activation was further dissected by examining the dose-responsiveness and processing requirements of peptide-pulsed antigen presenting cells.

**Results**

HLA-B*57:01 restricted CD8^+^ T-cell clones generated to self-peptides in the presence of abacavir proliferated and secreted IFN-Ƴ when rechallenged with the peptides and abacavir. Dose titration experiments revealed an enhanced response to the presence of self-peptides of a small number of clones at low abacavir concentrations. Similarly, certain clones produced responses to a subset of self-peptides in the absence of abacavir. Antigen presenting cell pulsing and glutaraldehyde fixation assays also revealed a stronger T-cell response to abacavir and a small number of self-peptides, when compared with abacavir alone. The Vβ receptor usage of these clones was unique to other abacavir-responsive clones.

**Conclusion**

These studies demonstrate that abacavir-induced HLA-B*57:01 binding self-peptides are involved in the activation of certain abacavir-responsive CD8^+^ T-cells.

#### P78 Investigation of the immunological basis of tolvaptan-induced liver injury

##### Andrew Gibson^1^, Khetam Alhilali^1^, Sharin E. Roth^2^, B. Kevin Park^1^, Dean J. Naisbitt^1^

###### ^1^Department of Molecular & Clinical Pharmacology, MRC Centre for Drug Safety Science, University of Liverpool, Liverpool, United Kingdom; ^2^Otsuka Pharmaceutical Development & Commercialization, Inc, Rockville, Maryland, United States

**Correspondence:** Andrew Gibson - a.gibson@liv.ac.uk

*Clinical and Translational Allergy* 2018, **8(Suppl 3)**:P78

**Background**

Tolvaptan, a nonpeptide arginine vasopressin V2-receptor antagonist, is associated with idiosyncratic liver injury. The delayed nature and prompt recurrence upon rechallenge indicate that injury results from an adaptive immune attack on the liver. Thus, a hypothesis for tolvaptan-induced liver injury is that the drug or a metabolic derivative(s) forms antigenic determinants with MHC molecules to activate T-cells.

**Methods**

Peripheral blood mononuclear cells were isolated from 9 tolvaptan-exposed patients with signs of hepatic injury and lymphocyte transformation tests were performed. To identify antigen-specific T-cells, peripheral blood mononuclear cells were cultured with antigen for 2 weeks prior to serial dilution and mitogen-driven expansion to generate T-cell clones. Antigen-specific T-cell proliferative responses were measured by thymidine incorporation, while secretion of IFN-γ, granzyme B, IL-13, and IL-22 were analysed by ELISpot. Individual T-cell clones were defined as CD4^+^ or CD8^+^ by flow cytometry. Further T-cell cloning was performed after magnetic bead purification of CD8^+^ T-cells from the initial bulk cultures in order to enhance the potential for identification of CD8^+^ antigen-specific T-cell clones.

**Results**

Lymphocyte transformation tests to all patients were negative for tolvaptan, and to two metabolites, DM-4103 and DM-4107. For 5/9 patients, a handful of T-cell clones surpassing the 1.5 stimulation index threshold were identified, the majority of which responded to tolvaptan-metabolites rather than tolvaptan itself. The best responding clone derived from bulk cultures with DM-4107, proliferated and secreted IFN-γ and granzyme B, alongside IL-13 in response to DM-4107. Cross-reactivity with tolvaptan or DM-4103 was not detected. All clones identified were CD4^+^. DM-4107 was selected as the most promising antigen to repeat the cloning experiments using purified CD8^+^ T-cells. Bulk cultures were subject to CD8^+^ magnetic bead separation prior to the second round of cloning. A handful of CD8^+^ DM-4107-responsive clones were found to secrete low levels of IFN-γ and IL-13 after exposure to DM-4107. Unlike CD4^+^ clones, weak cross reactivity was observed with tolvaptan and DM-4103.

**Conclusion**

We report the identification of CD4^+^ and CD8^+^ clones specific for the tolvaptan-derived metabolite DM-4107 in patients with DILI. Only a handful of clones were identified indicating that they likely represent a low frequency population in peripheral blood. In future experiments, an in vitro T-cell priming model will be used with peripheral blood mononuclear cells from drug-naïve donors to gain further insight into the aetiology of tolvaptan-induced liver injury.

#### P79 Characterization of dapsone- and nitroso-dapsone-specific T cells from hypersensitive patients expressing HLA-B*13:01

##### Khetam Alhilali^1^, Qing Zhao^2^, Abdulaziz Alzahrani^1^, Mubarak Almutairi^1^, Hong Liu^2^, Yonghu Sun^2^, Xiaoli Meng^1^, Andrew Gibson^1^, Monday O. Ogese^3^, B. Kevin Park^1^, David Ostrov^4^, Furen Zhang^2^, Dean J. Naisbitt^1^

###### ^1^Department of Molecular and Clinical Pharmacology, MRC Centre for Drug Safety Science, University of Liverpool, Liverpool, United Kingdom; ^2^Department of Dermatology, Shandong Provincial Hospital for Skin Disease, Shandong University, Jinan, Shandong 250022, China. Shandong Provincial Institute of Dermatology and Venereology, Shandong Academy of Medical Sciences, Jinan, Shandong, China; ^3^Pathology Sciences, Drug Safety and Metabolism, IMED Biotech Unit, AstraZeneca, Cambridge Science Park, Milton Road, Cambridge, United Kingdom; ^4^University of Florida, Gainesville, Florida, USA

**Correspondence:** Monday O Ogese - m.o.ogese@liverpool.ac.uk

*Clinical and Translational Allergy* 2018, **8(Suppl 3)**:P79

**Background**

Dapsone Hypersensitivity Syndrome is a severe adverse drug reaction characterized by development of fever, rash, jaundice, and lymphadenopathy after 5 or 6 weeks of dapsone (DDS) therapy. Approximately 0.5–3.6% of people suffer from the condition. Although the clinical presentation is well established, the role of drug-specific T cells has not been defined.

The objective of this study was to generate DDS- and nitroso DDS (DDS-NO; the main reactive metabolite of DDS)-specific T cells from hypersensitive patients and characterize the clones in terms of phenotype and function.

**Methods**

PBMC from 6 patients were cultured with DDS, DDS-NO, clofazimine and rifampicin and proliferative responses and IFN-g release were measured. DDS- and DDS-NO-specific T cell clones were generated from 3 patients using serial dilution followed by mitogen-driven expansion. The phenotype and function of drug-specific T cells were then determined. Drug-specific proliferative responses, cytokine secretion, cross reactivity with DDS-analogues/sulfonamides and MHC restricted drug-antigen presentation were evaluated.

**Results**

A total of 2728 clones were tested for drug specificity. 395 clones were activated with DDS, while DDS-NO activated 399 clones. 80% and 78% of the DDS and DDS-NO clones were CD4^+^, respectively. DDS- and DDS-NO-specific clones responded to graded concentrations of antigen with significant cross reactivity. Furthermore, DDS-NO-specific T cells displayed cross reactivity with DDS hydroxylamine. DDS and DDS-NO specific CD4^+^ T cells were MHC class II restricted, while CD8^+^ T cells responded to the drugs in an MHC class I-restricted manner. All clones secreted IFN-γ, IL-5, IL-13, IL-22, perforin, granzyme B, and Fas L in response to drug stimulation. IL-17A secretion was not detected. DDS and DDS-NO CD4^+^ T cells were CCR4_high_, CCR10_low_ and migrated in response to CCL17 and CCL27. Furthermore, DDS-specific CD8^+^ T cells expressing CCR4 and CCR9 migrated toward CCL17 and CCL25. T cells expressed a range of Vβ receptors.

**Conclusion**

These data show that DDS- and DDS-NO-specific CD4^+^ and CD8^+^ T cells circulate in hypersensitive patients; highlighting a critical role for antigen-specific T cells in the pathogenesis of DDS hypersensitivity.

#### P80 Histamine-release test in angioedema patients without urticaria – a retrospective cohort study of 404 patients

##### Georg Authried^1^, Sumangali Prasad^2^, Eva Rye Rasmussen^3^, Anette Bygum^4^

###### ^1^Department of Dermatology and Allergy Centre, Odense, Denmark; ^2^Odense University Hospital, Odense, Denmark; ^3^Department of Dermatology and Allergy Centre, Copenhagen, Denmark; ^4^Odense University Hospital, Copenhagen, Denmark

**Correspondence:** Eva Rye Rasmussen - eva.h.rye.rasmussen@gmail.com

*Clinical and Translational Allergy* 2018, **8(Suppl 3)**:P80

**Background**

A subset of patients with angioedema (AE) and urticaria has histamine releasing autoantibodies. The histamine release test (HR-test) has been used as a tool in chronic urticaria to define the autoimmune subgroup and may possibly guide the clinician to a more effective therapy like omalizumab and cyclosporine. The prevalence of positive histamine releasing autoantibodies in monosymptomatic AE is sparsely described in the literature. The purpose of the study was to report the prevalence of positive histamine releasing autoantibodies in a cohort of patients with recurrent AE without urticaria and compare it with previously published data.

**Methods**

We performed a retrospective cohort study of 612 AE patients seen at the Department of Dermatology, Odense University Hospital, between 1995 and 2013. Patients with AE with or without urticaria were compared using Fisher’s exact test. Difference of proportion test and odds ratio calculations were employed when comparing treatment efficacy. p-values ≤ 0.05 were considered statistically significant. 95% confidence intervals (CI) were reported when appropriate.

**Results**

HR-test results were available in 404 patients and showed that 17.3% had positive HR-tests in the subgroup of patients with AE and urticaria and 4.3% positive HR-tests in the subgroup of patients with AE only. There was a statistically significant difference between the two groups (*p *= 0.00002). In patients with a negative HR-test, treatment efficacy of antihistamines were significantly higher among AE patients with concomitant urticaria compared to patients with mono-symptomatic AE (p = 0.006, 95% CI 3.7726–23.5795). T

**Table 1:** Patients with angioedema (AE) ± urticaria tested with a HR-test

Total number of patients	404 with HR-test
Males	171
Females	233
M:F-ratio	0.73
Age, mean, median, [range], years	50.16, 51.39, [range 2.1–85.1 years]
Ethnicity	
Caucasian	393
Middle eastern	3
Black race	1
Asian	5
Other	1
Current tobacco use, n	
Yes	70
No	188
Unknown	146
Positive family history of AE, n	31
Number of HR-tests, *total*	404
Positive	39 (9.7%)
Negative	360 (89.1%)
Unknown result	5 (0.5%)
Comorbidities:	
Diabetes mellitus	32
Hypertension	115
Ischemic heart disease	24
Heart failure	7
Atopic dermatitis	21
Allergic rhinitis	56
Asthma	42
Other respiratory disease	4
Follow-up time, mean; [range], weeks	66.4; [0–675.1 weeks]
Reported effect of antihistamines, n	270
Reported effect of corticosteroids, n	165
Hospitalized due AE, n (%)	138 (34.2%)
ER visits due to AE, n (%)	144 (35.6%)

n = number of patients

he odds ratio was 2.0 for having a positive effect of antihistamine, if the patient had concomitant urticaria (Table 1).

When monosymptomatic AE patients with positive HR-test were compared with AE patients with concomitant urticaria and positive HR-test, the odds ratio was 2.2 for having a positive effect of antihistamine treatment.

**Conclusion**

Patients with AE and concomitant urticaria more often have a positive HR-test and might gain more treatment benefit from anti-allergic medication than patients with monosymptomatic AE.

#### P81 HLA-DR*15:02 allele increases the risk of low osmolar contrast media induced anaphylaxis

##### Dong Yoon Kang^1^, Hye Ryeon Son^1^, Sujeong Kim^2^, Seung Eun Lee^3^, Da Woon Sim^4^, Min-Gyu Kang^5^, Jae Woo Jung^6^, Sae-Hoon Kim^7^, Yoon-Seok Chang^7^, Whal Lee^8^, Hye Ryun Kang^9^

###### ^1^Drug Safety Monitoring Center, Seoul National University Hospital, Seoul, South Korea; ^2^Department of Internal Medicine, Kyungpook National University School of Medicine, Daegu, South Korea; ^3^Department of Internal Medicine, Pusan National University Yangsan Hospital, Yangsan, South Korea; ^4^Department of Internal Medicine, Chonnam National University Medical School, Gwangju, South Korea; ^5^Department of Internal Medicine, Chung-Buk National University Hospital, Chungju, South Korea; ^6^Department of Internal Medicine, Chung-Ang University Hospital, Seoul, South Korea; ^7^Department of Internal Medicine, Seoul National University, Bundang Hospital, Seongnam, South Korea; ^8^Department of Radiology, Seoul National University College of Medicine, Seoul, South Korea; ^9^Department of Internal Medicine, Seoul National University College of Medicine, Seoul, South Korea

**Correspondence:** Dong Yoon Kang - kdy@snu.ac.kr

*Clinical and Translational Allergy* 2018, **8(Suppl 3)**:P81

**Background**

As the use of iodinated contrast media (ICM) increases, the adverse reactions are also increasing. Among them, anaphylaxis is getting more attention according to its fatal consequences. However, there is no effective method for predicting anaphylaxis in patients other than a previous history. To develop the strategy of risk prediction based on genetic susceptibility, we analyzed the association between severe anaphylaxis to ICM and human leukocyte antigen (HLA) genotypes.

**Methods**

We searched patients who ever experienced anaphylaxis with administration of low osmolar ICM in the Korean registry of ICM adverse reaction. After obtaining consent to participate, blood samples were collected from for genotyping of HLA A, B, C, DR. Their genotypes were compared with those of Korean general population.

**Results**

A total of 23 patients suffering anaphylaxis to ICM were recruited. Their genotypes of HLA-A, B, C were not different from those of Korean general population. However, while DRB1*15:02 allele was positive in 6.6% of the general population in Korea, 30.4% in the anaphylaxis group had DRB1*15:02 allele. The risk for the development of ICM-induced anaphylaxis was significantly increased with this allele (OR 6.2 [95% CI 2.38–16.14], p = 0.00002).

**Conclusion**

A significant association of ICM-induced anaphylaxis with the HLA-DRB1*15:02 allele was observed in a Korean population. The results suggest that HLA-DRB1*15:02 allele may contribute to the development of ICM-induced anaphylaxis.

#### P82 Basophil activation tests can distinguish between IgE dependent and independent drug-induced anaphylaxis

##### Tatsuo Horiuchi^1^, Tomonori Takazawa^2^, Shigeru Saito^1^

###### ^1^Department of Anesthesiology, Gunma University Graduate School of Medicine, Maebashi, Japan; ^2^Intensive Care Unit, Gunma University Hospital, Maebashi, Japan

**Correspondence:** Tomonori Takazawa - takazawt@gunma-u.ac.jp

*Clinical and Translational Allergy* 2018, **8(Suppl 3)**:P82

**Background**

Drug-induced anaphylaxis is caused by various triggers, including binding of antigen specific Immunoglobulin E (IgE) to mast cells and basophils, and their direct activation. It is possible to investigate whether drug-specific IgE antibodies are involved in the onset of anaphylaxis by measuring their serum levels. However, this can only be assessed for certain drugs. Recently, the basophil activation test (BAT) has been established as a tool to detect the causative agent of anaphylaxis. In this study, we aimed to investigate whether drug-induced anaphylaxis by different drugs were IgE dependent or independent by using the BAT.

**Methods**

We recruited five patients with perioperative anaphylaxis, for which the culprit drugs were either rocuronium, protamine, cefazolin or cefotiam. All patients provided us with written informed consent for study participation. Skin tests with all drugs administered during the perioperative period were followed by BATs, with CD203c as the activated basophil marker. BATs were performed using serial dilutions of the culprit agents with and without wortmannin, an inhibitor of phosphoinositide 3-kinase. The percentage inhibition of basophil activation by wortmannin at drug concentrations at which basophils were most activated in each patient was calculated. Moreover, the area under the curve (AUC) in BATs with and without wortmannin was calculated. We used two different positive controls, anti-IgE antibodies and formyl-methionyl-leucyl-phenylalanine (fMLP), which is known to activate basophils through a different pathway from IgE.

**Results**

Skin tests showed positive reactions with culprit drugs in all patients. The culprit drugs in all patients resulted in basophil activation. The average value of activated basophils at drug concentrations at which basophils were most activated was 46 ± 35% (n = 5). The average value of the AUC was 346350 ± 287012. Wortmannin suppressed the number of activated basophils and AUC (Paired t-test, P = 0.02 and 0.03, respectively). Furthermore, the percentage inhibition of basophil activation by wortmannin was greater when anti IgE antibodies was used than when fMLP was used (92 ± 3% vs 32 ± 11%, t-test, P < 0.001).

**Conclusion**

Activation of basophils by culprit agents was inhibited by wortmannin in all patients tested in this study. We confirmed that wortmannin inhibited anti-IgE antibody-induced activation of basophils. These results suggest that rocuronium-, protamine-, cefazolin-, and cefotiam-induced anaphylaxis are likely caused by IgE antibody-dependent pathways. The BAT with wortmannin could distinguish IgE dependent and independent drug-induced anaphylaxis.

#### P83 An improved, non-radioactive lymphocyte proliferation test (LPT) for the diagnosis of delayed drug allergy

##### Marcello Albanesi^1^, Vincenzo Aresta^1^, Maria Pia Rossi^1^, Lucia Giliberti^1^, Mariangela Di Giacomo^1^, Andrea Nico^1^, Attilio Di Girolamo^1^, Danilo Di Bona^1^, Maria Filomena Caiaffa^2^, Luigi Macchia^1^

###### ^1^University of Bari-Aldo Moro, Bari, Italy; ^2^University of Foggia, Foggia, Italy

**Correspondence:** Marcello Albanesi - albanesimf@gmail.com

*Clinical and Translational Allergy* 2018, **8(Suppl 3)**:P83

**Background**

Drug hypersensitivity reactions (DHR) affect about 7% of the population. Nearly 15% of them have an underlying adaptive immunological mechanism, ranging from immediate to delayed hypersensitivity. DHR can be classified into type I to IV, according to Gell and Coombs. Type IV DHR have typically a delayed onset after the last drug intake, with diverse clinical manifestations ranging from Steven-Johnson syndrome to maculo-papular eruption. It has been demonstrated that T cell play a pivotal role in the development of the delayed DHR. In clinical practice, in vitro approaches for this kind of reactions are particularly scarce.

We developed an improved, non-radioactive lymphocyte proliferation test for the diagnosis of delayed DHR.

**Methods**

*Patients:* we performed LPT in 60 patients that were referred to our Clinic for a suspected delayed DHRs to different class of drugs including, antibiotics, NSAIDs and chemotherapeutic agents.

*Lymphocyte collection:* peripheral mononuclear cells were obtained from the patient’s buffycoats by dextran sedimentation followed by centrifugation on Lymphoprep and hypotonic lysis of contaminating erythrocytes.

*Proliferation assay:* Upon culture on glass Petri dishes for 4 days, in order to remove monocytes, the lymphocytes were incubated for 5 days with 3 different 10-fold concentrations of the offending drugs. Of note, the concentrations tested were chosen taking into account the volume of distribution of the drug. Upon incubation for 2 h with bromodeoxyuridine, lymphocyte proliferation was assessed by using an anti-bromodeoxyuridine monoclonal antibody. The test was deemed positive when the proliferation rate of any of the three concentrations tested (compared to the control) equals or exceeds 2, also called Stimulation Index (SI).

**Results**

*Positive cases:* the LPT was positive (SI > 2) in 20/60 patients analyzed. In these patients we were able to confirm the diagnosis of delayed DHR. Thus, we either suggested the avoidance of the offending drug, or, if the drug was considered indispensable, we applied a desensitization protocol to administered the drug.

*Negative cases:* the LPT was negative (SI < 2) in 40/60 patients. In these cases the previously suspected delayed DHR was ruled out. Thus, we performed drug-tolerance test, enabling the reintroduction in therapy of the suspected drug.

**Conclusion**

The presented improved and non-radioactive LPT is a useful diagnostic tool that could be routinely used in the diagnosis of delayed DHR.

## Thursday 19 April 2018

### Diagnosis and pathophysiology - Poster Walk 9

#### P84 Humanized in Vitro IgE mediated desensitization: microscopy phenotype of desensitized mast cells and variability of mediators’ release during the different steps of the desensitization

##### Leticia De Las Vecillas Sanchez^1^, Leila A. Alenazy^2^, Mariana C. Castells^3^

###### ^1^Department of Allergy, Marqués de Valdecilla University Hospital-IDIVAL, Santander, Spain; ^2^Department of Medicine, College of Medicine, King Saud University, Riyadh, Saudi Arabia; ^3^Division of Rheumatology, Immunology and Allergy, Department of Medicine, Brigham and Women’s Hospital, Harvard Medical School, Boston, MA, United States

**Correspondence:** Leticia De Las Vecillas Sanchez - leticia.delasveci@gmail.com

*Clinical and Translational Allergy* 2018, **8(Suppl 3)**:P84

**Background**

Drug desensitization (DS) was developed to reintroduce first line therapies in a safe and effective fashion in allergic patients. Mast cells (MCs) are key effectors and are believed to be the primary target cell in DS. In vitro model of rapid MCs IgE mediated DS has shown the inhibition of the MC activation hallmarks, reaching at the end of the protocol the antigen target dose. This study presents a humanized in vitro model of DS showing the microscopy phenotype difference between activated-desensitized MCs and the variable hyporesponsiveness of MC along the DS protocol.

**Methods**

Bone marrow-derived mast cells (BMMCs) from transgenic Balb/c mice expressing the human high affinity IgE receptor alpha chain (hFeRI) were sensitized with chimeric IgE anti-NP. A dose–response curve to NP-BSA were done to assess the target antigen dose. Desensitization protocol were done in an eleven sequentially delivery doses, starting with a 1/1000 of the target dose. Antigen dose was doubled each ten minutes (optimal interval time to inhibit MCs degranulation), reaching the target dose at the end of the protocol. Light microscopy was used to see toluidine blue dye mast cell granules during activation (single target dose delivery) and desensitization. Beta-hexosaminidase release during single doses antigen delivery were compared with the same antigen doses added sequentially following the eleven steps of the DS protocol.

**Results**

A dose–response curve to NP-BSA antigen showed 10 ng of NP-BSA as the optimal triggering dose (target dose). Light microscopy shows “empty” mediators MCs after challenge, in contrast to the presence of cytoplasmic mediator granules in desensitized MCs. The beta-hexosaminidase percentage release were significantly lower in desensitized MCs compared to activated MCs. Challenge with single DS doses induced a sequential increase of beta-hexosaminidase release after the sixth step of the protocol, creating a dose response curve. Sequentially added doses also induced an hyporesponsiveness as shown by lack of degranulation.

**Conclusion**

Humanized MCs/IgE-mediated desensitization model allows to evaluate the inhibition of MC mediators release during DS. Microscopy images prove the presence of MCs mediator granules inside the cells after received the target antigen dose by DS in contrast to activated MCs which lose all the cytoplasmic granules. This fact confirms that desensitization blocks completely MCs degranulation. Comparing the beta-hexosaminidase release during the different DS protocol steps, we confirm the importance of the first sub-optimal DS steps to get a total MC hyporesposiveness at the end of the procedure.

#### P85 Active pharmaceutical ingredient concentration in false-negative drug patch tests

##### Maja Jošt^1^, Tanja Prodanovic^2^, Mitja Košnik^1^, Mihaela Zidarn^1^, Mojca Kerec Kos^2^

###### ^1^University Clinic Golnik, Golnik, Slovenia; ^2^Faculty of Pharmacy, Ljubljana, Slovenia

**Correspondence:** Maja Jošt - maja.jost@klinika-golnik.si

*Clinical and Translational Allergy* 2018, **8(Suppl 3)**:P85

**Background**

Drug patch tests (DPT) for diagnostics of delayed cutaneous adverse drug reactions should be highly specific and sensitive, but at the same time non-irritant. The non-irritant concentrations of some active pharmaceutical ingredients (APIs) were established. However, the data on the lowest concentration of APIs that still produces positive reaction is scarce.

Our goal was to assess the content of the active ingredient in the DPT in connection to the results of the DPT and oral provocation test (OPT) in the routine clinical practice.

**Methods**

A retrospective single-centre study was conducted in adult patients referred to allergy department of University Clinic Golnik between January 2011 and May 2015. Patients with negative DPT and subsequent OPT with the same drug were selected. Two allergologists independently assessed the type and probability of allergic reaction described in medical history. The patients with doubtful delayed cutaneous adverse drug reaction or insufficient data were excluded. The patients’ documentation, the results of DPT and OPT, as well as the content of the API in DPT, were reviewed. The subsequent assessment of the API content in the preparations for DPT made by 30% dilution of the commercialised drugs in petrolatum was made from documentation However, only of the hospital pharmacy.

**Results**

In 108 patients 299 DPT were performed with 87 different APIs. 38 patients with 47 negative DPT and further OPT were selected for further analysis. However, only patients with 33 negative DPT suited all inclusion criteria. Seven out of 33 performed OPT (21%) were positive, indicating false-negative DPT. They were performed with 7 different APIs.

The average content of API in the DPT prepared by 30% dilution of the commercialised drugs in petrolatum was 19.73%, ranging from 0.12% (dexamethasone) to 28.05% (metformin). In the group of false-negative tests the average content of AI in DPT was 19.60% (range from 9.03 to 25.00%).

**Conclusion**

The analysis shows a high number of different APIs considering the number of all performed DPT. It confirms as it was stated in similar studies, that the actual content of API in the DPT made by 30% dilution of the commercialised drugs in petrolatum differs significantly depending on API and drug formulation. High percentage of false-negative DPT, though in the small sample, urges better standardization of tests in clinical practise, especially the evaluation of the actual API content for the uncommon drugs.

#### P86 Passive Basophil sensitization assay for the diagnosis of immediate type drug hypersensitivity reactions

##### Nicole Müller-Wirth^1^, Oliver Hausmann^2^, Antonia Bünter^1^, Werner J. Pichler^1^

###### ^1^Adverse Drug Reactions- Analysis and Consulting (ADR-AC) GmbH, Bern, Switzerland; ^2^Löwenpraxis & ADR-AC GmbH, Lucerne & Bern, Switzerland

**Correspondence:** Nicole Müller-Wirth - nicole.wirth@adr-ac.ch

*Clinical and Translational Allergy* 2018, **8(Suppl 3)**:P86

**Background**

Besides skin testing, basophil activation test with patient basophils (direct BAT) is used in the diagnosis of immediate type drug hypersensitivity reactions. However, the need of fresh, functionally active basophils and the occurrence of non-responder basophils in 10–15% of patients limit the use of direct BAT. Here we present a serum-based test, “indirect BAT” using well-characterized healthy donor basophils sensitized with patients’ sera in vitro.

**Methods**

PBMCs of well-characterized basophil donors were isolated and donor basophils were stripped of their IgE by lactic acid pre-treatment and resensitized with patients’ sera in vitro. IL-3 primed basophils were then stimulated with serial drug dilutions and controls. Basophil resensitization (surface IgE expression) and basophil activation (CCR3+ CD63+ cell population) was measured by flow cytometry.

**Results**

Using sera of patients sensitized to chlorhexidine (divalent drug) revealed that 11/12 patients with a positive direct BAT were also positive in indirect BAT, if the chlorhexidine specific IgE was > 1.0 kU/l (n = 12). Further drugs, disinfectants and additives (divalent and monovalent) tested positive in the indirect BAT were penicillin G, amoxicillin, cefaclor, cefuroxime, ceftriaxone, ceftazidime, cefotaxime, carboxymethylcellulose, ibuprofen, octenidine and proguanil.

**Conclusion**

We were successful in improving and standardizing the conditions of indirect BAT using sera of drug-allergic patients on non-allergic donor basophils. It is a promising tool for drugs, where no serologic testing is available and/or drug provocation or skin testing is too risky. In addition, indirect BAT increases reproducibility using different sera on the same basophil donor (batch analysis) and provides a way to overcome the problem of IgE non-responders in BAT. Limitations of the indirect BAT are (a) a certain concentration of drug specific IgE is needed for basophil activation (preliminary cut-off > 1.0 kU/l) and (b) a better understanding of drug-protein interactions and conditions for IgE crosslinking by drugs is needed to further optimize preanalytic test conditions.

#### P87 Single cell approaches to define Allopurinol-SJS/TEN Pathogenesis

##### Katie D. White^1^, Mark A. Pilkinton^1^, Wyatt J. Mcdonnell^1^, Katherine C. Konvinse^1^, Alec Redwood^2^, Abha Chopra^2^, Louise Barnett^1^, Rama Gangula^1^, Ren-You Pan^3^, Wen-Hung Chung^4^, Shuen-Iu Hung^3^, Simon A. Mallal^1,2^, Elizabeth J. Phillips^1,2^

###### ^1^Vanderbilt University Medical Center, Nashville, TN, United States; ^2^The Institute for Immunology & Infectious Diseases, Murdoch University, Perth, Australia; ^3^National Yang-Ming University, Taipei, Taiwan; ^4^Chang Gung Memorial Hospital, Taipei, Taiwan

**Correspondence:** Katie D. White - katie.d.white@vanderbilt.edu

*Clinical and Translational Allergy* 2018, **8(Suppl 3)**:P88

**Background**

Stevens–Johnson syndrome/toxic epidermal necrolysis (SJS/TEN) pathogenesis is mediated by CD8+ T cells, NK cells, and NKT cells. In Southeast Asians, allopurinol-associated SJS/TEN is associated with carriage of HLA-B*58:01 with a negative predictive value of 100%. Outside of Southeast Asia HLA-B*58:01 explains only 60% of the risk for allopurinol SJS/TEN and to-date there have been no studies defining other HLA class I risk alleles. Furthermore, the positive predictive value of the HLA-B*58:01 association is ≤ 8% suggesting that factors other than HLA genotype confer risk.

**Methods**

A biobank of paired peripheral blood, blister fluid and skin was accrued from an ethnically diverse group of patients with allopurinol SJS/TEN. High resolution HLA ABC DR DQ DP typing was performed on the Illumina Miseq platform. Single cell technologies including T-cell receptor αβ sequencing, multi-parameter flow cytometry, full transcriptome RNA sequencing, and mass cytometry were used to define the clonality and molecular signatures of T cells from paired PBMC, blister fluid, and skin obtained from patients with allopurinol-SJS/TEN.

**Results**

Dominant CD8+ T-cell clonotypes of effector memory phenotype (CCR7-) with variable expression of skin homing markers (CD69, CLA) were identified in HLA-B*58:01-associated allopurinol-SJS/TEN blister fluid and were present at much lower frequency in oxypurinol-stimulated recovery phase peripheral blood. Single-cell RNA sequencing of activated CD8+ T cells from blister fluid (CD137+) demonstrated enrichment of extracellular exosome and T-cell activation (CD38, TIGIT, TNFRSF1B, and KLRC1) transcripts compared to non-activated (CD137-) CD8+ T cells. Further, activated CD8+ T cells expressed significantly higher levels of granulysin mRNA (p = 4.6 × 10^−9^), the cytolytic protein thought to mediate keratinocyte cell death in SJS/TEN. Analysis of a second case of allopurinol-SJS/TEN in an African American woman that was secondary to inadvertent drug re-challenge revealed a novel HLA association and identified a dominant CD8+ T-cell clonotype that was shared among blister fluid and anatomically-paired epidermal resident (CD103+) T-cell populations but seen at lower frequency in T cells isolated from unaffected skin.

**Conclusion**

Single cell approaches that define CD8+ T-cell clonality and transcriptional signatures of allopurinol-SJS/TEN have the potential to identify candidate pathogenic effector cells and will be tested for drug reactivity in vitro. These methods will help drive the development of targeted therapeutics and screening approaches.

#### P88 Study of protein targets for haptenation by biotinylated clavulanic acid: usefulness in studies on allergy towards betalactams

##### Ángela Martín-Serrano^1^, Nekane Barbero^2^, Juan Manuel González-Morena^3^, Francisco José Sánchez-Gómez^3^, Tahía Fernández^4^, Alba Rodríguez-Nogales^4^, Ezequiel Pérez-Inestrosa^2^, Dolores Pérez-Sala^3^, Cristobalina Mayorga^4^, Adriana Ariza^4^, María José Torres^1^, María Isabel Montañez^1^

###### ^1^IBIMA-UMA; BIONAND, Málaga, Spain; ^2^UMA; BIONAND, Málaga, Spain; ^3^CIB-CSIC, Madrid, Spain; ^4^IBIMA-UMA, Málaga, Spain

**Correspondence:** Adriana Ariza - a.arizaveguillas@gmail.com

*Clinical and Translational Allergy* 2018, **8(Suppl 3)**:P88

**Background**

Clavulanic acid (CLV) is a betalactam (BL) which inhibits betalactamases activity and is frequently administered combined with amoxicillin (AX). Both BLs can be independently involved in allergic reactions. Indeed, selective immediate allergic reactions to CLV have recently been reported in 30% of patients allergic to the AX-CLV combination. Although protein haptenation with BLs is considered necessary to activate the immune system, currently there are no straight-forward detection tools, such as a monoclonal antibody against CLV, for the study of protein haptenation with CLV.

The objective was to study haptenation by CLV and develop tools to identify CLV target proteins. To achieve this aim, CLV was derivatized with biotin, then incubated with either human serum albumin (HSA) or sera and, finally, the resulting protein adducts were analyzed by different techniques.

**Methods**

Biotinylated CLV (CLV-B) was synthesized by means of introducing an oxygenated linker between CLV and a biotin moiety. HSA was incubated with increasing concentrations of CLV or CLV-B and the resulting conjugates were purified by filtration and characterized by MALDI-TOF MS. Sera were incubated with CLV-B and proteins separated by 2D electrophoresis followed by HRP-streptavidin detection. Competition experiments between CLV and CLV-B for HSA haptenation were performed by HSA incubation with CLV-B, after a preincubation with increasing concentrations of CLV. Aliquots of the incubation were subjected to SDS-PAGE and transferred to a PVDF membrane. The incorporation of CLV-B, assessed by detection with HRP-streptavidin.

**Results**

MALDI-TOF analysis of conjugates showed that HSA was modified by both CLV and CLV-B (mass increment of 279.8 and 543.0 respectively, compared with HSA control). The modification was proportional to the amount of CLV or CLV-B used during incubation. Results were in agreement with the grade of biotinylation in HSA-CLV-B, determined by electrophoresis using biotinylated BSA as calibrator. 2D electrophoresis of sera incubated with CLV-B showed spots corresponding to HSA, transferrin and heavy and light chains of immunoglobulins as candidate targets for CLV-B haptenation. As result of competition experiments, HSA pre-incubation with an excess of CLV moderately reduced the incorporation of CLV-B.

**Conclusion**

Our results show that CLV-B could be a valuable tool for the identification of CLV targets with high sensitivity. The elucidation and comparison of CLV and CLV-B reactivity during conjugation deserve further study to finally understand the activation of the immune system by CLV.

#### P89 Single cell analysis of cutaneously recruited drug-specific T cells: support for the altered peptide model of abacavir hypersensitivity

##### Alec James Redwood^1^, Francois Rwandamuriye^1^, Abha Chopra^1^, Shay Leary^1^, Ramesh Ram^1^, Katherine Konvinse^2^, Katie White^2^, Rebecca Pavlos^1^, Simon Mallal^2^, Elizabeth Jane Phillips^2^

###### ^1^Institute for Immunology and Infectious Diseases, Murdoch University, Perth, Australia; ^2^Vanderbilt University Medical Center, Nashville, TN, United States

**Correspondence:** Alec James Redwood - a.redwood@iiid.com.au

*Clinical and Translational Allergy* 2018, **8(Suppl 3)**:P89

**Background**

Abacavir specifically binds within the F pocket of HLA-B*57:01 altering the peptide binding cleft and repertoire of presented self-peptides resulting in T-cell recognition of altered self and clinical hypersensitivity (ABC HSR). In keeping with this model, T-cell responses to ABC in the peripheral blood are known to be polyclonal, reflecting the vastly different immunopeptidome presented by HLA-B*57:01 in the context of abacavir.

Specific T-cell responses in the skin of ABC HSR have not been previously studied. We sought to define the characteristics of abacavir specific T cells that are recruited to the skin at the site of a positive ABC patch test several years following the acute reaction.

**Methods**

A patient with a prior history of ABC HSR and positive ABC patch test in 2003 was re-patch tested and biopsied at 96 h following patch application. We isolated T cells from 1% and 10% patch test positive skin and unaffected control skin. CD3+ T cells were single cell sorted and subjected to primer based single cell TCR-seq (SC-TCR-seq) and RNA-seq (SC-RNA-seq). Cloned T-cell lines from patch test positive and control skin were tested for ABC specificity by interferon gamma ELISpot and subjected to bulk TCR sequencing.

**Results**

Both CD4 + and CD8+ T cells were recruited to ABC patch test positive skin. SC-TCR-seq defined a highly polyclonal CD3+ T cell population and SC-RNA-seq defined their gene expression profiles. T-cell lines cloned from patch test positive skin, unlike those cloned from unaffected control skin, expressed a polyclonal TCR repertoire that responded to ABC (10 µg/ml).

**Conclusion**

For first time we have shown single-cell definition of T cells at the site of a cutaneous response to ABC. Furthermore, these ABC reactive T cells can be cloned from patch test positive skin and are distinctly polyclonal. This supports the altered peptide repertoire model of T-cell activation and provides a road map for studying drug-specific responses at a tissue level.

#### P90 Reduction of the drug-specific T-cell response during desensitisation in patients with non-immediate piperacillin hypersensitivity reactions

##### Paul Whitaker^1^, Andrew Gibson^2^, John Farrell^2^, B. Kevin Park^2^, Daniel Peckham^1^, Dean J. Naisbitt^2^

###### ^1^Regional Adult Cystic Fibrosis Unit, St James’s Hospital, Leeds, United Kingdom; ^2^Department of Molecular and Clinical Pharmacology, MRC Centre for Drug Safety Science, University of Liverpool, Liverpool, United Kingdom

**Correspondence:** Andrew Gibson - a.gibson@liv.ac.uk

*Clinical and Translational Allergy* 2018, **8(Suppl 3)**:P90

**Background**

ß-lactam hypersensitivity reactions in patients with cystic fibrosis represent an important clinical problem with 20% in the Leeds unit having a single adverse event and 30% two or more adverse events. The vast majority of patients present with non-immediate rashes, arthralgia and fever. Patient management is especially complicated in the multi-allergic patient group where alternative treatment strategies have often been exhausted. In these patients, desensitisation represents a well-tolerated method of reintroducing the drug by gradually increasing a suboptimal dose until a therapeutic dose is achieved. Patient success with tazocin (piperacillin, tazobactam) desensitisation is approximately 55%. Despite this, little is known about the immunological basis of a successful non-immediate desensitisation. Aim: To conduct lymphocyte transformation tests on hypersensitive patient PBMC before, during and after tazocin desensitisation to assess whether a graded increase in drug exposure modulates the drug-specific T-cell response.

**Methods**

Nine piperacillin hypersensitive patients (eight patients maculopapular rash and one patient with arthralgia/drug fever) were reintroduced to tazocin through a seven step rapid desensitisation protocol. Blood was taken prior to desensitisation and on up to 5 occasions after treatment commenced at 1, 4, 24, 168, 336 h. PBMC were isolated and cultured with piperacillin (0.1–4 mM) for 6 days. [^3^H] thymidine was added for the final 16 h of the culture period and incorporated radioactivity was measured by scintillation counting.

**Results**

PBMC from all nine hypersensitive patients were stimulated to proliferate in a concentration-dependent manner with piperacillin prior to commencement of the desensitisation protocol. Seven of the patients safely tolerated the tazocin desensitisation and a 14 day treatment course (4 g piperacillin by rapid infusion, 3 × a day). The strength of the piperacillin-specific PBMC proliferative response weakened in all patients 1 and 24 h after desensitisation was started, and remained negative for the duration of the treatment. Two patients failed desensitisation at day 5 (arthralgia/fever) and day 10 (maculopapular rash).

**Conclusion**

These data show that the drug-specific T-cell response is inhibited in b-lactam hypersensitive patients with non-immediate symptoms undergoing an established desensitisation protocol. In on-going experiments we are using viable PBMC and plasma collected prospectively from the patients to characterize levels of drug protein adducts and changes in regulatory receptors/molecules that might explain the results described herein.

#### P91 Importance of specific IgE/total IgE ratio in disambiguating amoxicillin allergy diagnosis in a real-life setting

##### Alessandro Sinisi^1^, Marcello Albanesi^1^, Flavia Maria Frisenda^1^, Danilo Di Bona^1^, Maria Filomena Caiaffa^2^, Luigi Macchia^1^

###### ^1^School and Chair of Allergology and Clinical Immunology, Department of Emergency and Organ Transplantation, Bari, Italy; ^2^School and Chair of Allergology and Clinical Immunology, Department of Medical and Surgical Sciences, Foggia, Italy

**Correspondence:** Alessandro Sinisi - alessandro.sinisi90@gmail.com

*Clinical and Translational Allergy* 2018, **8(Suppl 3)**:P91

**Background**

Allergic reactions to b-lactams are the most frequent cause of Adverse Drug Reactions (ADRs) mediated by specific immunological mechanisms. Amoxicillin, widely used for the treatment of bacterial infections, belongs to this drug class. In clinical practice, detection of b-lactam-specific IgE levels (CAP System) offers a complementary approach to diagnose allergy to antibiotics. Its sensitivity is rather low, although specificity is high. The positive predictive value of this test might also be influenced by the total amount of serum IgE. It was recently proposed that a positive predictive value of 95% is achieved if the ratio between drug-specific IgE and total IgE is ≥ 0.002, whereas this value diminishes significantly if the ratio is < 0.002.

**Methods**

To clarify the clinical diagnosis of suspected ADR to amoxicillin, we used the 0.002 amoxicillin-specific IgE/total IgE ratio in 5 cases of particularly ambiguous diagnosis, based on clinical evidence only.

We observed 5 patients with suspected, although ambiguous, immediate ADR to amoxicillin. At first, we evaluated the circulating amoxicillin-specific IgE levels (CAP System). In all patients the levels were slightly above the current threshold limit (i.e. 0.1 kU/l). We also evaluated the total IgE levels, which were all above the usually accepted threshold limit of 100 kU/l. Thereby, we calculated the amoxicillin-specific IgE/total IgE ratio. Notably, this ratio was below 0.002 in all the patients, resulting in a positive predictive value for amoxicillin-specific IgE < 95%. That suggested that the 5 patients were not sensitized to amoxicillin. To validate this hypothesis, we performed an oral drug provocation test with amoxicillin. Moreover, the drug was administered for 3 consecutive days, following the provocation test day.

**Results**

All the patients tolerated the drug provocation procedure. No adverse reactions were observed, thus excluding the possible amoxicillin hypersensitivity that could have been suspected formerly, based on crude RAST results.

**Conclusion**

The ratio between amoxicillin-specific IgE and total IgE (threshold 0.002) is a useful parameter to rule out false positives. We propose that total IgE levels should be always assessed when amoxicillin-specific IgE are evaluated.

#### P92 Generation and characterization of amoxicillin- and clavulanic acid-specific t-cell clones derived from blood of amoxicillin-clavulanic acid hypersensitivity patients

##### Adriana Ariza^1^, María Isabel Montañez^2^, Monday O Ogese^3^, Arun Tailor^4^, Tahia D Fernández^1^, María José Torres^5^, Dean J Naisbitt^4^

###### ^1^Research Laboratory, IBIMA – Regional University Hospital of Malaga – University of Malaga, Málaga, Spain; ^2^Research Laboratory, IBIMA – Regional University Hospital of Malaga – University of Malaga; BIONAND, Málaga, Spain; ^3^Pathology Sciences, Drug Safety and Metabolism, IMED Biotech Unit, AstraZeneca, Cambridge, United Kingdom; ^4^Dept. Molecular & Clinical Pharmacology, MRC Centre for Drug Safety Science, University of Liverpool, Liverpool, United Kingdom; ^5^Allergy Unit, IBIMA – Regional University Hospital of Malaga – University of Malaga, Málaga, Spain

**Correspondence:** Adriana Ariza - adriana.ariza@ibima.eu

*Clinical and Translational Allergy* 2018, **8(Suppl 3)**:P92

**Background**

Betalactam antibiotics are the most frequently prescribed antibiotics to treat infections. However, they are the most common cause of drug hypersensitivity reactions mediated by a specific immunological mechanism. Amoxicillin (AX) is the most often elicitor, which was originally prescribed alone, and is now often prescribed alongside clavulanic acid (Clav). The diagnosis is complex, based on skin testing and drug provocation test, methods not risk-free. *In vitro* testing can be used, however their sensitivity is low, probably due to the use of chemical structures that are not optimally recognized by the immune system. The aim of this study was to generate and characterize AX- and Clav-specific T-cell clones from blood of hypersensitive patients to be used as tool to study the immunological recognition of new chemical structures derived from AX and Clav to be included in in vitro diagnostic tests.

**Methods**

Peripheral blood mononuclear cells (PBMC) were isolated from AX-Clav hypersensitive patients. Drug-specific T-cell clones were generated from PBMC by serial dilution and repetitive mitogen stimulation. Antigen specificity was assessed by measurement of proliferation and cytokine release using [^3^H]-thymidine release and IFN-γ and IL-13 ELISpot, respectively.

**Results**

110 AX-specific and 96 Clav-specific T-cell clones were generated from 7 patients. Activation and proliferation of AX- and Clav-specific T-cell clones was dose-dependent, no cross-reactivity between AX and Clav was observed and they presented mainly with a CD4^+^ phenotype. AX- and Clav-specific T-cell clones required the presence of drug and antigen presenting cells to proliferate. Drugs were presented to CD4^+^ T-cell clones by MHC class II and to CD8^+^ T-cells by MHC class I. Finally, the highest level of cytokine secreted following drug treatment was IFN-γ, followed by IL-13, IL-5 and IL-10. Most clones expressed high levels of CD69, CCR4, CCR10 and CXCR3.

**Conclusion**

AX- and Clav-specific T-cell clones can be generated from AX-Clav hypersensitivity patients, with no cross-reactivity between AX and Clav. They are activated by AX or Clav only in the presence of antigen presenting cells, supporting the hapten hypothesis for the recognition and presentation of betalactam antibiotics. The specific T-cell clones generated are an immunologically characterized tool that can be used for the analysis of new structures derived from AX and Clav to be included in in vitro diagnostic tests.

#### P93 HLA-A*11:01 and A*24:02 are risk factors for Malay patients with urticaria/angioedema induced by non-steroidal anti-inflammatory drugs

##### Mohammed Faizal Bakhtiar^1, 2^, Chun Lai Too^3^, Min Moon Tang^4^, Lay Kim Tan^3^, Salsabil Sulaiman^3^, Nurul Aain Ahmad Fauzi^3^, Anastasia Ria Nagum^1^, Giriyappanavar Chandrashekar Rayappa^2^

###### ^1^Allergy Unit, Allergy & Immunology Research Center, Institute for Medical Research, Kuala Lumpur, Malaysia; ^2^Faculty of Medicine, Universiti Kuala Lumpur Royal College of Medicine Perak, Ipoh, Perak, Malaysia; ^3^Immunogenetic Unit, Allergy & Immunology Research Center, Institute for Medical Research, Kuala Lumpur, Malaysia; ^4^Department of Dermatology, Kuala Lumpur Hospital, Kuala Lumpur, Malaysia

**Correspondence:** Mohammed Faizal Bakhtiar - drfuzzy73@gmail.com

*Clinical and Translational Allergy* 2018, **8(Suppl 3)**:P93

**Background**

Non-steroidal anti-inflammatory drugs (NSAIDs) hypersensitivity is one of the leading cause of adverse reactions in susceptible patients, with NSAIDs induced urticaria/angioedema (NIUA) being the commonest phenotype. HLA genes associations with NSAIDs hypersensitivity have been observed across different populations. This study aimed to determine the HLA genetic risk factors in Malay patients with NIUA as compared to the ethnically matched NSAIDs-tolerant controls.

**Methods**

Thirty-five Malay patients with at least two chemically different NSAIDs hypersensitivity of the NIUA phenotype and 32 Malay NSAIDs-tolerant controls (NTC) were included. All participants were genotyped for HLA-A, -B, -DRB1, -DQA1 and -DQB1 using polymerase chain reaction sequence-specific oligonucleotide probe hybridization method.

**Results**

We observed increased frequencies of HLA-A*11:01 and HLA-A*24:02 in NIUA patients induced by NSAIDs when compared to the NSAIDs tolerant controls (HLA-A*11:01, NIUA versus NTC, 34.1% versus 9.4%, OR = 5.0, 95% CI 1.76–14.23; HLA-A*24:02, NIUA versus NTC, 22.7% versus 6.3%, OR = 4.40, 95% CI = 1.29–15.15). Intriguingly, stratification analysis by atopy subsets revealed that HLA-A*11:01 and DRB1*12:02 were significantly associated with NIUA with atopy (A*11:01, OR = 4.83, 95% CI 1.72–13.58; DRB1*12:02, OR = 2.33, 95% CI 1.03–5.27, respectively). On the other hand, HLA-A*24:02 and DRB1*14:04 were significantly associated with NIUA without atopy (A*24:02, OR = 4.83, 1.72–13.58; DRB1*14:04, OR = 5.98, 95% CI = 1.30–27.59). In this study, we also observed that HLA-A*11:01 was significantly associated with the clinical manifestation towards angioedema (angioedema only, OR = 3.78, 95% CI = 1.21–11.83, p < 0.05; urticaria and angioedema, OR = 3.22, 95% CI = 1.04–9.97, p < 0.05).

**Conclusion**

The current data suggest that HLA-A*11:01 and HLA-A*24:02 as risk factors for developing NIUA in the Malay population. Whilst HLA-A*11:01 increased the risk of NIUA with atopy subset and with clinical manifestation of angioedema, HLA-A*24:02 increased the risk of NIUA subset without atopy.

#### P94 Modifications of the cyclopropyl moiety of abacavir provides an insight into the relationship between the drug interaction within the HLA-B*57:01 antigen binding cleft and T-cell activation

##### Paul James Thomson^1^, Patricia T. Illing^2^, John Farrell^1^, Mohammad Alhaidari^1^, Neil Berry^1^, Paul M. O’Neill^1^, Anthony W. Purcell^2^, B. Kevin Park^1^, Dean J. Naisbitt^1^

###### ^1^MRC Centre for Drug Safety Science, Dept of Molecular and Clinical Pharmacology, University of Liverpool, Liverpool, United Kingdom; ^2^Infection and Immunity Program, Monash Biomedicine Discovery Institute and Dept of Biochemistry and Molecular Biology, Monash University, Clayton 3800, Victoria, Melbourne, Australia

**Correspondence:** Paul James Thomson - p.j.thomson@liverpool.ac.uk

*Clinical and Translational Allergy* 2018, **8(Suppl 3)**:P94

**Background**

The reverse transcriptase inhibitor abacavir is associated with severe hypersensitivity reactions seen exclusively in individuals positive for the HLA-B*57:01 risk allele. Abacavir interacts within the F-pocket of HLA-B*57:01, altering the shape and chemistry of the antigen binding cleft, leading to an altered array of self-peptides displayed on the cell surface. Presentation of this new array of peptides to T-cells is hypothesized to trigger a CD8^+^ T-cell response in hypersensitive patients.

The aim of this study was to investigate the relationship between the interaction of abacavir derivatives with HLA-B*57:01 and CD8^+^ mediated T-cell responses using novel compounds as molecular probes.

**Methods**

Eighteen abacavir analogues were synthesised with modifications to the cyclopropyl moiety, namely derivatives of the azetidine group. Abacavir responsive T-cell clones were generated from four healthy donors, positive for HLA-B*57:01. The cytokine secretion of these clones was measured using an IFN-Ƴ Eli-spot assay. In silico docking studies were used to assess the potential binding poses of the abacavir analogues within HLA-B*57:01. The effect of the abacavir analogues on the repertoire of self-peptides eluted from antigen presenting cells expressing HLA-B*57:01 was measured by mass spectrometry.

**Results**

Major histocompatibility complex class I restricted CD8^+^ T-cell clones proliferated and secreted IFN-Ƴ in response to abacavir. Several abacavir analogues were devoid of T-cell activation, whilst others demonstrated a strong CD8^+^ response. Molecular docking studies demonstrated a relationship between the binding orientations of the analogues within HLA-B*57:01 and the ability to induce a T-cell response. Detailed analysis of abacavir clones with two HLA-B*57:01 binding derivatives revealed that T-cell activation was enantiomer specific. Impact on HLA-B*57:01 peptide presentation was investigated for several compounds, identifying a relationship between the observed T-cell responses and presentation of peptides with small C-terminal anchors. CD8^+^ T-cells were cloned from lines generated by culturing PBMC with 4 abacavir derivatives to confirm absence of T-cell reactivity.

**Conclusion**

These studies demonstrate that modification of the cyclopropyl moiety of abacavir offers an insight into the interaction of parent drug with HLA-B*57:01. This approach may provide a platform towards the design of safer therapeutics known to interact selectively with HLA molecules.

## Friday 20 April 2018

### Desensitisation or treating through the reaction?

#### O03 Pathways to improved antibiotic allergy practice - the validation of a beta-lactam antibiotic allergy assessment tool to aid accurate phenotyping and management

##### Misha Devchand^1^, Karen Urbancic^1^, Sharmila Khumra^1^, Steven Walker^1^, Abby Douglas^2^, Olivia Smibert^2^, Ar Kar Aung^3^, Elizabeth J. Phillips^4^, Jason A. Trubiano^1^

###### ^1^Austin Health, Melbourne, Australia; ^2^Peter MaCallum Cancer Centre, Melbourne, Australia; ^3^Alred Health, Melbourne, Australia; ^4^Vanderbilt University Medical Centre, Nashville, TN, United States

**Correspondence:** Jason A Trubiano - jason.trubiano@austin.org.au

*Clinical and Translational Allergy* 2018, **8(Suppl 3)**:O03

**Background**

Patient-reported antibiotic allergies (so-called antibiotic allergy labels [AALs]) are frequently encountered in hospitalized patients, however the correct assessment of these AALs are often poor. We validated an antibiotic allergy assessment tool (AAAT) to aid non-allergist in phenotyping and management of patients with reported beta-lactam AALs.

**Methods**

An antibiotic allergy assessment tool (AAAT) was developed at Austin Health (Melbourne, Australia). The AAAT utilizes patient-reported symptoms and signs associated with an index beta-lactam allergy label (Fig. 1), to assign an ADR phenotype and management recommendation. The phenotypic outcomes of the AAAT were classified as; (i) severe immediate (IgE-mediated), (ii) non-severe immediate (IgE-mediated), (iii) severe delayed (T-cell-mediated), (iv) non-severe delayed (T-cell-mediated), (v) potential immune mediated (e.g. acute interstitial nephritis) or (vi) unlikely to be significant/non-immune mediated (e.g. gastrointestinal upset, unknown history). Each phenotype was allocated a corresponding management recommendation of either; (i) direct de-labelling with removal from electronic health record ii) direct oral rechallenge (iii) inpatient (point-of-care) antibiotic allergy skin testing, or (iv) outpatient antibiotic allergy assessment potentially including skin testing and oral challenge. The tool was initiatelly reviewed for content and usability, utilizing hypothetical case scenarios and the final validation involved a range of medical participants utilizing the AAAT, in real-patient case studies (from the Austin Health Antibiotic Allergy Database).

**Fig. 1:**
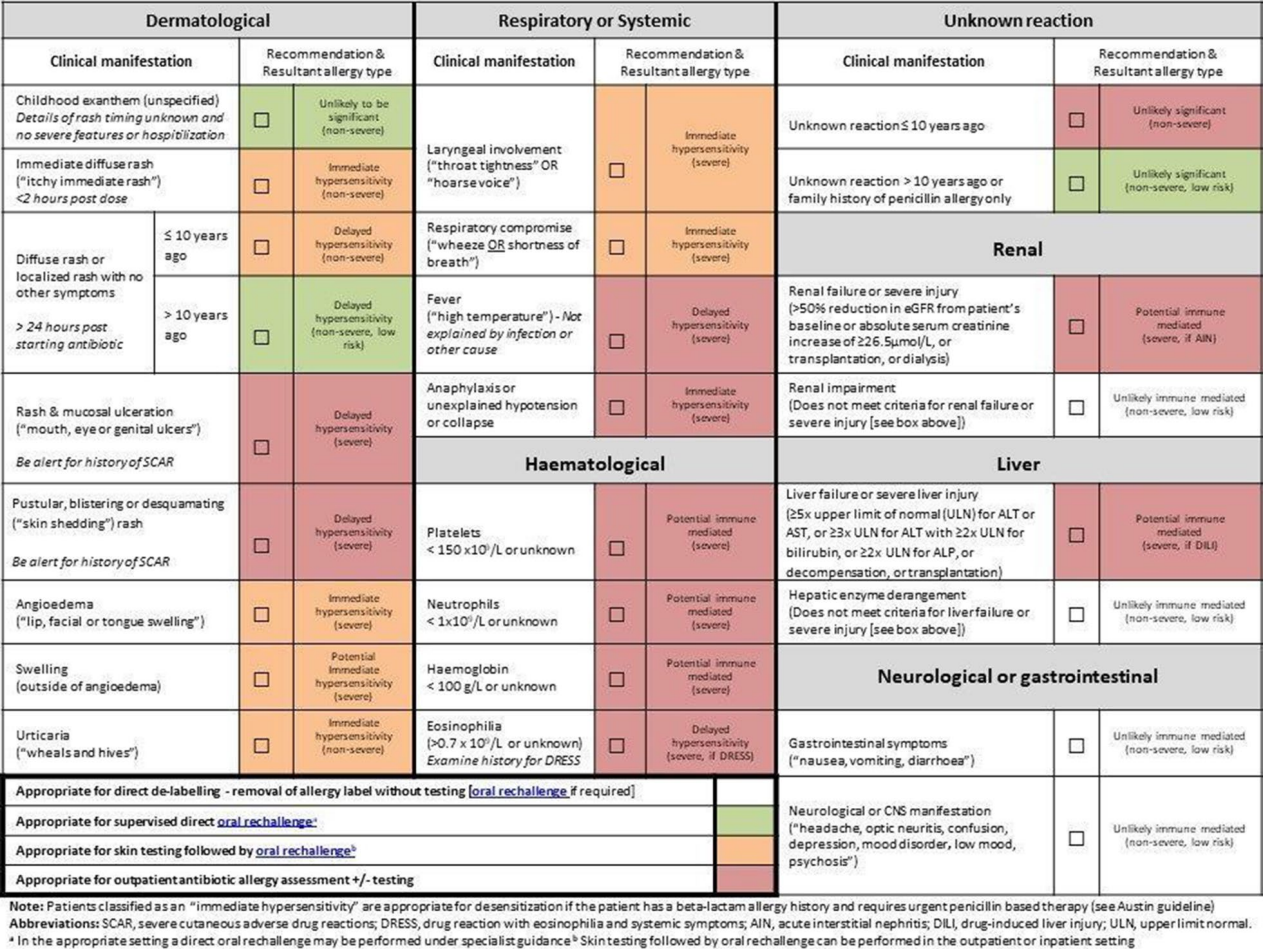
Beta-lactam antibiotic allergy assessment tool

**Results**

Eight antibiotic allergy case studies (immune-mediated [5] and non-immune-mediated [3]) were employed by forty participants to validate the tool; 19 junior doctors (48%), 11 pharmacists (28%), 8 (20%) infectious diseases doctors [consultants and fellows] and 2 (5%) specialist nurses. The AAAT demonstrated an overall sensitivity of 91.5% (95% CI 87.9–94.3) for assigning the correct phenotype and 85.9% (95% CI 81.5–89.5) for the appropriate management. The highest sensitivity and specificity was for the identification of severe immediate hypersensitivities, 97.5% (95% CI 91.3–99.7) and 99% (95% CI 96.7–99.9) respectively, and lowest for severe delayed hypersensitivities, 77.5% (95% CI 61.6–89.2) and 99.5% (97.5–99.9) respectively. The AAAT demonstrated a sensitivity and specificity of 97.5% (95% CI 86.8–99.9) and 96.0% (95% CI 90.2–98.9) respectively, for the identification of non-immune-mediated phenotypes.

**Conclusion**

The beta-lactam AAAT proved a useful tool for a range of healthcare workers in the identification of antibiotic allergy phenotypes, and designating appropriate management. Lower sensitivity for severe delayed hypersensitivity identifies this as a safety concern and a target for education of healthcare personnel. Antimicrobial stewardship programs and a range of hospital specialists could use such tools, in the future, to aid inpatient antibiotic allergy “de-labelling”.

## Friday 20 April 2018

### Genetics of drug hypersensitivity

#### O04 HLA-B*13:01 to prevent dapsone hypersensitivity syndrome

##### Hong Liu^1^, Yonghu Sun^1^, Furen Zhang^2^

###### ^1^Shandong Provincial Institute of Dermatology and Venereology, Jinan, China; ^2^Correspondence Author, Shandong Provincial Institute of Dermatology and Venereology, Jinan, China

**Correspondence:** Hong Liu - suohandong@126.com

*Clinical and Translational Allergy* 2018, **8(Suppl 3)**:O04

**Background**

Dapsone hypersensitivity syndrome (DHS), as a life-threatening condition, is the most serious adverse reaction associated with dapsone intake and one of the major causes of death in leprosy patients. *HLA*-B*13:01 allele has been identified as the genetic determinant of DHS in Chinese population. This study aimed to determine the clinical efficacy of prospective *HLA*-B*13:01 screening to reduce the incidence of DHS.

**Methods**

*HLA*-B*13:01 genotyping was performed using quantitative polymerase chain reaction (qPCR) method. *HLA*-B*13:01 carriers were suggested to eliminate dapsone from multi-drug therapy (MDT) and *HLA*-B*13:01 non-carriers took MDT. All the subjects were followed up for two months to monitor the adverse events. Immunological tests were done to distinguish DHS from leprosy reactions and reactions to other drugs. Historic incidence of DHS was used as controls to evaluate the clinical efficacy of prospective *HLA*-B*13:01 screening.

**Results**

We recruited a total of 1,272 newly diagnosed leprosy patients for *HLA*-B*13:01 genotyping from 21 provinces throughout China in the period of February, 2015 to November, 2017. 216 (17.0%) subjects were found to carry *HLA*-B*13:01 allele and treated by excluding dapsone from MDT. 1,056 (83.0%) subjects were *HLA*-B*13:01 negative and were treated with standard MDT. None of the subjects in the *HLA*-B*13:01 negative group received a diagnosis of DHS. In contrast, based upon the historic incidence, 11–33 patients of DHS would be expected. No significant correlation was found between other adverse events and *HLA*-B*13:01 status.

**Conclusion**

The prospective screening of *HLA*-B*13:01 allele before commencing leprosy treatment and elimination of dapsone from MDT for *HLA*-B*13:01 carrier could significantly reduce the incidence of DHS in Chinese population.

## Friday 20 April 2018

### Clinical cases - Poster Walk 10

#### P96 Inpatient penicillin allergy evaluation safely increases utilization of beta lactams

##### Keith Sacco, Benjamin Cochran, Mehmet Tatari, Catalina Sanchez Alvarez, Tiba Al Sagheer, Beric Berlioz, Kristina Colmenares, Emily Gilbert, Lindsay Gardner, Cory Gooch, Anna Maria Mechtler, Kevin Epps, Alexei Gonzalez Estrada, Mark Parkulo

###### Mayo Clinic, Jacksonville, Florida, United States

**Correspondence:** Benjamin Cochran - cochran.benjamin@mayo.edu

*Clinical and Translational Allergy* 2018, **8(Suppl 3)**:P96

**Background**

A history of penicillin allergy is associated with increased risk of nosocomial infection (MRSA, VRE and *C. difficile*) and increased length of hospitalization. Over 95% of patients reporting penicillin allergy can tolerate beta lactams without adverse events. Our aim was to increase uptake of beta lactam antibiotics by 15% in patients reporting a penicillin allergy without increasing adverse drug reactions and length of hospitalization.

**Methods**

We prospectively collected and analyzed demographic data of patients with a penicillin allergy admitted to the internal medicine residency service at Mayo Clinic Florida between August 1 and August 30, 2017. We implemented an educational initiative including use of a validated antibiotic selection algorithm to aid physicians in prescribing beta lactams based on history of drug hypersensitivity.

**Results**

247 patients were admitted between August 1 and August 30, 2017 of which 42 (17.0%) reported a history of penicillin allergy. 42.9% (18) of patients received inpatient antibiotics. 19.5% received a cephalosporin while 4.76% received penicillins. No inpatient penicillin skin testing was performed. In the 21-day post-intervention re-measurement phase, 23 patients with penicillin allergy were admitted of which 56.5% received inpatient antibiotics. Post-intervention drug allergy history documentation increased by 43.1%, p < 0.001. Cephalosporin use increased by 55.5%, p < 0.001 and penicillins by 20.24%, p = 0.03. Mean length of stay was unchanged (3.62 days [CI 2.4–4.8 days] versus 3.12 days [CI 2.2–4.1 days], p = 0.24). No adverse drug reactions were reported.

**Conclusion**

A significant proportion of hospitalized patients with penicillin allergy receive unnecessary broad-spectrum antibiotics partly due to poor penicillin allergy evaluation. This quality improvement initiative focused on educating primary providers to take a thorough drug allergy history is safe and effective at increasing uptake of beta lactams without increasing healthcare utilization.

#### P97 Flucloxacillin hypersensitivity: patient outcomes in a multicenter, retrospective study

##### Lucinda Kennard^1^, Leonard Siew^2^, Rita Mirakian^1^, Krzysztof Rutkowski^2^, Phillip Li^3^, Alla Nakonechna^4^, Annette Wagner^1^

###### ^1^Cambridge University Hospitals NHS Foundation Trust, Cambridge, United Kingdom; ^2^Guy’s and St.Thomas’ NHS Foundation Trust, London, United Kingdom; ^3^Queen Mary Hospital University of Hong Kong, Hong Kong, Hong Kong SAR; ^4^Liverpool Hope University, Liverpool, United Kingdom

**Correspondence:** Annette Wagner - annette.wagner@talk21.com

*Clinical and Translational Allergy* 2018, **8(Suppl 3)**:P97

**Background**

Immediate and delayed drug hypersensitivity reactions (DHR) to beta lactams can be directed to both the shared beta lactam ring and to specific side chains. DHR to flucloxacillin are less common than those to aminopenicillins.

We present the results of a 5 year retrospective audit of patients with a history of suspected hypersensitivity reaction to flucloxacillin, referred to two allergy centers in the UK.

**Methods**

Data analyzed included patient demographics, index reaction details, outcomes of skin prick (SPT), intradermal (IDT) testing with penicillin determinants (PPL and MD, Diater), benzylpenicillin, amoxicillin and flucloxacillin and oral drug provocation tests with flucloxacillin.

**Results**

83 patients (56 female, 27 male) were investigated. 22 patients (26.5%) were diagnosed with flucloxacillin allergy. One patient had a positive SPT to Flucloxacillin, 13 patients had a positive IDT to flucloxacillin, in 2 of those patients the IDT was delayed positive. 2 of 11 patients with a positive immediate IDT were also positive to PPL and MD, one of the patients developed a systemic reaction to skin testing.

One of two patients with a positive delayed IDT was also positive to benzylpenicillin and amoxicillin.

In 8 patients with negative skin tests allergy was confirmed by oral drug provocation with flucloxacillin, using a 1–3 step protocol. Of those patients 5 developed an immediate reaction (one required adrenaline); 3 reacted within 24–72 h of a course of flucloxacillin, given as a continuation of oral drug provocation.

2 patients subsequently tolerated amoxicillin.

**Conclusion**

Flucloxacillin allergy is more common in women than in men. IgE mediated hypersensitivity is more common (77%) than T-cell mediated hypersensitivity. The majority of skin test positive patients (81%) reacted to flucloxacillin only, confirming the importance of side chain recognition. The negative predictive value of skin tests in this cohort was 88%. Allergy persisted for more than 5 years in 3 patients.

Oral drug provocation testing in skin test negative patients, using a shortened protocol, was safe in the specialist setting.

#### P98 Beta-Lactam allergy in children: are skin testing and serum specific IgE reliable predictors?

##### Annabelle Arnold^1^, Laure Braconnier^1^, Aine Sommerfield^2^, Michelle Trevenen^3^, Yogesh Jeelall^4^, Christine Blundell^5^, David Sommerfield^6^, Lliana Slevin^2^, Kevin Murray^7^, Britta Von Ungern-Sternberg^6^, Kristina Rueter^1^, Michaela Lucas^1^

###### ^1^Department of Clinical Immunology, Princess Margaret Hospital, Perth, Australia; ^2^Telethon Kids Institute, Perth, Australia; ^3^Centre for Applied Statistics, University of Western Australia, Perth, Australia; ^4^UWA Medical School, University of Western Australia, Perth, Australia; ^5^Immunology Department, PathWest Laboratory Medicine, Perth, Australia; ^6^Department of Anaesthesia and Pain Management, Princess Margaret Hospital, Perth, Australia; ^7^School of Population and Global Health, University of Western Australia, Perth, Australia

**Correspondence:** Michaela Lucas - michaela.lucas@health.wa.gov.au

*Clinical and Translational Allergy* 2018, **8(Suppl 3)**:P98

**Background**

Beta-lactam allergy can currently be diagnosed based on positive skin testing, serum-specific IgE and provocation challenge. However, in the context of distant reactions, large paediatric cohort studies assessing the sensitivity and specificity of skin testing and serum-specific IgE are few. The role of extended antibiotic challenges remains unclear.

**Methods**

320 children (52.3% male) with a distant history (> 6 months) of beta-lactam allergy were assessed. Children with a clear history of anaphylaxis (e.g. directly observed and documented by medical staff) were excluded. Previously-reported reactions included macular/maculopapular rashes (75.5%), urticaria (30.7%), gastrointestinal symptoms (15.8%), angioedema (15.2%), anaphylaxis (15.8%) and non-specific symptoms (5.3%). Data collection included results of skin-prick testing (SPT), intradermal testing (IDT; immediate and delayed), graded oral provocation challenge (OPC) (1/10 and full dose), 5-day oral antibiotic extended course (EC), serum specific IgE (penicillin G and V, ampicillin, amoxicillin). All children received an OPC regardless of reaction history, or preceding skin and serum test results. Children were challenged with the culprit beta-lactam antibiotic; if unknown, amoxicillin was used.

**Results**

320 children aged 1–17.7 years (mean 8.16) underwent OPC; the three most common antibiotics tested were amoxicillin (63.7%), penicillin (21.1%) and cephalexin (7.8%). Six children reacted immediately to the OPC (2.2%) with mild rash/urticaria, three after the full dose. One of these children had a positive IDT. One child with a positive skin-prick test passed OPC. Five additional children had a positive IDT (1.9%) and passed the OPC. 14/320 had a positive serum-specific IgE for at least one beta-lactam, all of these children passed the OPC.

Of the 311 children who went home on a 5-day EC, 20 (6.4%) reacted during the course between days 1–6. None had a positive delayed IDT, 1 had a positive immediate IDT, 19 had negative skin testing and specific IgE. The most common reactions to the EC were rashes (62.1%), urticaria (24.1%), and angioedema (10.3%), no child required adrenaline. 280 children passed the OPC and EC, 14 had inconclusive outcomes (4.5%).

**Conclusion**

In our study, skin testing and serum beta-lactam specific IgEs were poor predictors of positive reactions to oral provocation testing. For the 320 children challenged, all reactions to OPC or EC were mild with no anaphylaxis. This provides further data that oral provocation testing in children with distant reactions is safe and that skin testing and testing for specific IgE were poor predictors of true allergy.

#### P99 Penicillin allergy de-labelling in the elective surgical population - PADLES study

##### Louise Savic^1^, Vikas Kaura^1^, Lucy Gurr^2^, John Toolan^3^, Philip Hopkins^3^, Jonathon Sandoe^3^, Sinisa Savic^3^

###### ^1^Leeds Teaching Hospitals NHS Trust, Leeds, United Kingdom; ^2^University of Leeds, Leeds, United Kingdom; ^3^Leeds Teaching Hospital NHS Trust, Leeds, United Kingdom

**Correspondence:** Louise Savic - louise.savic@nhs.net

*Clinical and Translational Allergy* 2018, **8(Suppl 3)**:P99

**Background**

Tackling spurious penicillin allergy labels is a key target for improving antimicrobial stewardship. 10–20% of the population carry the label, but 95–98% are incorrect when tested. The label results in use of broad spectrum alternatives, and is associated with higher rates of *Clostridium difficile*, VRE and MRSA, increased hospital stay and critical care admission.

In elective surgical patients, the potential consequences of the allergy label carry significant health cost implications. Work in our Trust demonstrated that 17% of elective patients have the label, with half of these requiring a penicillin for surgical prophylaxis.

Current testing guidelines are laborious and expensive, requiring skin tests prior to oral challenge. An increasing body of evidence demonstrates that patients can be risk stratified on the basis of history, and ‘low risk’ patients can safely omit skin testing, proceeding to direct oral challenge.

We amied to identify and de-labelling ‘low risk’ penicillin allergic patients pre-operatively, thereby reducing use of alternative antimicrobial prophylaxis.

**Methods**

All patients attending for pre-operative assessment who self-reported penicillin allergy, completed a screening questionnaire. Using a proforma, pre-assessment nurses identified ‘low risk’ patients (defined as symptoms not suggestive of immediate and/or immunologically mediated adverse reaction), and invited them to be tested. At a dedicated de-labelling clinic, they received a graded oral amoxicillin challenge (or the index penicillin if known), performed by a specialist nurse with medical support available.

Patients completed a 3-day amoxicillin course, and were then contacted by phone. De-labelling was confirmed in writing, with GP and hospital records updated accordingly.

Antibiotics subsequently used during surgery were recorded, and GPs contacted at 3–6 months to check the recorded allergy status.

**Results**

Of 160 patients screened, 45 were eligible for inclusion, 115 ineligible. To date, 37 patients have been de-labelled, and (where known), received appropriate penicillin prophylaxis uneventfully. 2 patients had minor side effects and were not de-labelled. No patient had a reaction on initial challenge.

In the ineligible group, 68/115 were classified as ‘high risk’; 21% of these only had an itchy rash. Interest in testing is high; 75% of all patients wanted to be tested, largely irrespective of presenting symptoms (80% low risk and 70% high risk patients requested testing). Around 30% of patients with life-threatening symptoms also requested testing.

**Conclusion**

Elective surgical patients at low risk of penicillin allergy can be identified and de-labelled pre-operatively, as part of their existing surgical care pathway. Patients appear keen to be tested, providing this pathway is simple and quick.

#### P100 Penicillin allergy evaluation

##### Miriam Sobrino García, Francisco Javier Muñoz Bellido, María Teresa Gracia Bara, Esther Moreno Rodilla, María Elena Laffond Yges, Eva María Macías Iglesias, Milagros Lázaro Iglesias, Sonia De Arriba Méndez, María Del Valle Campanón Toro, Ignacio Jesús Dávila González

###### Hospital Universitario de Salamanca, Salamanca, Spain

**Correspondence:** Miriam Sobrino García - miriam_sobrino@hotmail.com

*Clinical and Translational Allergy* 2018, **8(Suppl 3)**:P100

**Background**

Unverified penicillin allergy leads to adverse downstream clinical and economic sequelae. Penicillin allergy evaluation can be used to identify true, IgE-mediated allergy.

Beta-lactam antibiotics are the drugs that most frequently produce adverse reactions mediated by a specific immunological mechanism.

Within these, penicillins are the group most involved and best studied. Currently, amoxicillin, probably due to its high consumption, has displaced benzylpenicillin in terms of the number of reactions.

**Methods**

We have evaluated 90 patients in our Hospital with reported penicillin allergy for 7 months, doing penicillin allergy histories, penicillin skin testing and drug challenges under medical observation to distinguish true allergy.

**Results**

We have obtained a positive result in 17 patients out of 90 with reported penicillin allergy (18.88%). Less than a quarter of patients have personal history of atopy (22.22%) and nearly a third of patients have family history of atopy (30%). They had a personal history of drug allergy 7.78% of patients. Most of the patients had taken one (40.24%) or two doses (15.85%) previously allergic reaction. The reaction was immediate on the 31% and not immediate in 49% of patients. The clinic was predominantly cutaneous (75.86%), anaphylaxis presenting nearly the fifth part of patients (18.39%). Most of patients did not go to the emergency room (57.30%) or were once (33.71%), requiring treatment with corticoids (47.78%), antihistamines (52.22%), adrenaline (5.56%) and other treatments (10%). The number of visits to nursing in the majority of cases was two (40%) or one (24.44%). The 53.33% previously tolerated the drug and the 30% tolerated later drugs from the same group.

**Conclusion**

In our study, skin prick test was positive in the 4.49% (one patient), intradermal test in the 13.48% (eleven patients) and patch tests in the 1.12% (one patient). Drug challenge was positive in the 4.44% (four patients). We have obtained a positive result in the 18.88%, data similar to others described in the literature. One of them shows us that only 19% of the suspected cases of reported betallactams allergy were confirmed, emphasizing the importance to refer patients to an Allergy Centre for investigation.

#### P101 Implementing allergy tests in inpatients with history of allergy to betalactam antibiotics

##### Lluis Marques, Silvia Lara, Eva Alcoceba

###### University Hospitals Santa Maria - Arnau de Vilanova. IRBLeida, Lleida, Spain

**Correspondence:** Lluis Marques - situmarques@gmail.com

*Clinical and Translational Allergy* 2018, **8(Suppl 3)**:P101

**Background**

Inpatients who have unconfirmed history of allergy to betalactam antibiotics and who need antibiotics pose a challenge. Avoiding betalactamic antibiotics suppose therapeutic limitations, the use of second line drugs, a prolonged hospital stay, an increase of risk of adverse effects and more expenditure. A majority of the patients who report allergy to betalactam antibiotics tolerate this drugs.

Objectives: To confirm or to rule out betalactam allergy with allergy tests in inpatients of two university hospitals with an unconfirmed history of betalactam allergy an who need antibiotic treatment.

**Methods**

Skin tests with betalactam antibiotics, following ENDA guidelines, were done on demand of the physician responsible of the inpatient. If the skin tests were negative and the decision was to change the antibiotic, a provocation test was done with a 10% of the dose and after 30 min with 100% of the dose. The protocol was approved by the infectious diseases committee.

**Results**

In 4 years, allergy tests to betalactam antibiotics were done in 63 inpatients. 36 (57%) were women and the median age was 79. 60.3% of patients were using second line antibiotics and in 28.5% some betalactam antibiotics were prescribed before the study. Skin tests were negative in 95.2% of patients, not valid in 1 case and positive in 3.1% (2 patients). One was positive with bencilpeniciloate and had a history of a mild immediate reaction; the other was positive with piperacillin and a history of a late cutaneous reaction. Provocation tests were done in 78.3% of the 60 patients where it could be performed: all were negative, and antibiotic treatment was changed in all of them. In 6 patients it was decided to continue with the previous antibiotic, 1 patient denied consent, in 1 antibiotic was not needed, and in 5 there were no data available. Provocations were done with amoxicillin (40.4%), cephalosporins (25.5%), carbapenems (27.6%) and other betalactams (6.4%). In 27.6% of patients with negative provocation tests, the alarm of allergy to betalactam antibiotics was not erased from the clinical history.

**Conclusion**

A majority of inpatients with history of allergy to betalactam antibiotics are not allergic, but a minority is really allergic. The allergy study permits the reduction of use of second line antibiotic. The number of patients studied is low: is necessary to improve the knowledge of the possibility of doing allergy tests in inpatients and increase the visibility of allergologists in the hospital. The wrong labelling of allergies is common.

#### P102 Booster effect in penicillin allergy - a case report

##### Lenka Sedlackova, Miroslav Prucha

###### Hospital Na Homolce, Prague, Czech Republic

**Correspondence:** Lenka Sedlackova - lenka.sedlackova@homolka.cz

*Clinical and Translational Allergy* 2018, **8(Suppl 3)**:P102

**Background**

Sensitivity of penicillin allergy tests decreases with time elapsed between reaction and evaluation. Therefore some recommendations include a repetition of allergy work-up in penicillin anaphylaxis evaluated years after index reaction. A case report of a 41-year old patient documents the risks related to the delayed allergy examination.

**Case report**

The patient experienced her first reaction within 3–4 h after the first tablet of penicillin at the age of 16: a rash, pruritus and a subjective feeling of tongue swelling. Treatment at the emergency department had a prompt effect. Two months later the same reaction to penicillin appeared again, only the symptoms subsided after H1-antihistamine at home. Allergy work-up started 25 years later. Specific IgE to penicilloyl V was 0.28 kUA/l, ampicilloyl 0.16 kUA/l, penicilloyl G and amoxicilloyl < 0.1 kUA/l. BAT was negative for PPL, MD, benzylpenicillin G, ampicillin, amoxicillin. Skin prick and intradermal tests were negative for PPL, MD, benzylpenicillin G, ampicillin and amoxicillin + clavulanic acid in immediate and later readings. The oral provocation test with phenoxymethylpenicillin was performed according to a protocol for non-immediate reactions at weekly intervals. Doses 5, 25 and 100 mg were well tolerated. Ten minutes after 750 mg anaphylaxis grade 2 occurred. Specific IgE measured four weeks later increased to 9.4 kUA/l (penicilloyl V), 3.31 kUA/l (penicilloyl G), 6.66 kUA/l (ampicilloyl) and 0.11 kUA/l (amoxicilloyl). BAT remained negative.

Delayed allergy work-up is negatively affected by inaccurate history and insufficient sensitivity of tests. In this case the skin tests performed 25 years after the last reaction failed completely and specific IgE fell into a grey zone which was regarded as non-specific. The interval 3–4 h between drug and reaction together with mildness of both anamnestic reactions contributed to misclassification as a non-immediate reaction. Penicillin provocation at weekly intervals between individual doses then led to booster response with anaphylaxis after the fourth dose. Immediate type of hypersensitivity was confirmed by the rise of specific IgE.

**Conclusion**

Immediate type of hypersensitivity to beta-lactam should be considered in an overlapping interval 1–6 h between drug administration and reaction as well as in borderline results of specific IgE 0.1–0.35 kUA/l. Drug provocation ought to be performed according to a protocol for immediate hypersensitivity in such patients and investigation should be repeated in patients with a history of remote immediate reaction when the first evaluation is negative.

**Consent to publish**

Consent to publish was obtained from the patient involved in this study.

#### P103 Antibiotic allergy labels in children are associated with adverse clinical outcomes

##### Michaela Lucas^1^, Annabelle Arnold^1^, Aine Sommerfield^2^, Michelle Trevenen^3^, Laure Braconnier^1^, Alina Schilling^1^, Fuad Abass^1^, Lliana Slevin^2^, Brittany Knezevic^4^, Christopher Blyth^5^, Kevin Murray^6^, Britta Von Ungern-Sternberg^7^, Kristina Rueter^1^

###### ^1^Department of Clinical Immunology, Princess Margaret Hospital, Perth, Australia; ^2^Telethon Kids Institute, Perth, Australia; ^3^Centre for Applied Statistics, University of Western Australia, Perth, Australia; ^4^School of Pathology and Laboratory Medicine, University of Western Australia, Perth, Australia; ^5^Pathwest Laboratory Medicine, Perth, Australia; ^6^School of Population and Global Health, University of Western Australia, Perth, Australia; ^7^Department of Anaesthesia and Pain Management, Princess Margaret Hospital, Perth, Australia

**Correspondence:** Michaela Lucas - michaela.lucas@health.wa.gov.au

*Clinical and Translational Allergy* 2018, **8(Suppl 3)**:P103

**Background**

Self-reported antibiotic allergies are common among hospitalised adults and children. There is clear data demonstrating that adults carrying an antibiotic allergy label have poorer clinical outcomes; however, there is a paucity of studies investigating the impact of an antibiotic allergy label in childhood. The objective of this study was to examine if parentally reported antibiotic allergy labelling in children significantly impacts on their clinical care.

**Methods**

A retrospective study was conducted in a major paediatric tertiary hospital in order to capture all inpatient admissions in April 2014 and April 2015. Data were collected by chart review and included documented antibiotic allergy labels, antibiotic prescriptions during the stay, infections during hospital stay, admitting specialty, hospital length of stay, and hospital readmissions.

**Results**

Of the 1675 paediatric patients surveyed, 58.1% were male and 44.7% were prescribed antibiotics. The age ranged between 0 and 18.7 years with 44.1% of patients being under 5 years old. The admitting specialties were classified as 52.1% general surgical, 26.6% general medical, 6.1% oncology and 15.1% “other medical specialties”. Antibiotic allergy labels were recorded in 5.3% of patients; the majority were beta-lactam labels (85%), mostly to unspecified penicillins. There was an increasing incidence of antibiotic allergy label with age, which was statistically significant (P < 0.001); no gender effect was seen. Patients with antibiotic allergy labels received more macrolide (P = 0.045), quinolones (P = 0.01), lincosamide antibiotics (P < 0.001) as well as more metronidazole (P = 0.009) than patients without an antibiotic allergy label. After adjusting for patient age, sex and admitting specialty, children with any antibiotic or beta-lactam allergy label, had longer hospital lengths of stay (OR 1.74, 95% CI 1.13–2.67, P = 0.01).

**Conclusion**

This is the first study demonstrating the negative impact of antibiotic allergy labels on clinical outcomes in children, as evidenced by significant alternate antibiotic use and longer hospital lengths of stay. De-labelling strategies implemented in childhood may be a means to reduce the use of alternative antibiotics and the associated increase in bacterial resistance. Moreover, early de-labelling may be beneficial from a health economic point of view, by reducing the prevalence and negative impact of allergy labels among children, and the future adult population,

## Friday 20 April 2018

### Clinical cases - Poster Walk 11

#### P104 Suspected ceftriaxone hypersensitivity in children is difficult to confirm

##### Tina Vesel Tajnšek^1^, Anja Koren Jeverica^1^, Mira šilar^2^, Jasmina Livk^3^, Alojz Ihan^3^, Peter Korošec^2^, Tadej Avcin^1^

###### ^1^University Children’s Hospital, University Medical Center, Ljubljana, Slovenia; ^2^University Clinic of Respiratory and Allergic Diseases, Golnik, Slovenia; ^3^Medical faculty, University of Ljubljana, Ljubljana, Slovenia

**Correspondence:** Tina Vesel Tajnšek - tina.vesel@kclj.si

*Clinical and Translational Allergy* 2018, **8(Suppl 3)**:P104

**Background**

Diagnosing procedures when suspected hypersensitivity to betalactame antibiotics (BL) are clearly defined. Ceftriaxon is among cephalosporins most frequently associated with suspected hypersensitivity in children in Slovenia. The aim of our study was to describe clinical pictures and diagnosing procedures when hypersensitivity to ceftriaxon is suspected.

**Methods**

The demographic and clinical data of 65 children referred to tertiary allergologic department during years 2011–2015 because of suspected ceftriaxone hypersensitivity were retrospectively studied. 36 children during treatment of Lyme disease presented with suspected hypersensitivity to ceftriaxone (three children with anaphylaxis, six with immediate urticaria, nine with delayed urticarial/angioedema, 18 with complex delayed reactions) and 29 children had confirmed hypersensitivity to other BL. Diagnosing procedures made were skin testing (ST)—including with ceftriaxone of concentration 2 mg/ml, determining specific IgE to routinely available BL, basophil activation testing (BAT) to ceftriaxone and drug provocation testing (DPT) to ceftriaxone. DPT to ceftriaxone lasted one to three days. Two different performances of BAT were used—one according to Buhlmann (in 35 children) and other adopted in University Clinic of Respiratory and Allergic Diseases Golnik (in 30 children) both performing analyses of basophil CD63 surface expression in association with stimulation index (SI).

**Results**

Hypersensitivity to ceftriaxone was confirmed in seven children (in six children with ST and in one child with DPT). In two children with anaphylaxis after ceftriaxone hypersensitivity could not be confirmed even by repeated DPT with ceftriaxone. Two children had anaphylaxis during ST with ceftriaxone and were treated with intramuscular adrenaline. DPT with ceftriaxone was not be recommended in half of children (18) with suspected ceftriaxone hypersensitivity because of history of delayed complex reactions. BAT did not significantly differ by either technique allergic vs tolerant (p > 0.05). Four children had SI to ceftriaxone > 2, including one child with clinical history of anaphylaxis with ceftriaxone and one who suffered anaphylaxis during performing ST with ceftriaxone.

**Conclusion**

In suspected hypersensitivity to ceftriaxone in children limitations of available diagnosing methods were observed: 1. cases of anaphylaxis could not be confirmed even by repeated DPT, 2. during ST anaphylaxis were caused, 3. ST to ceftriaxone of concentration 2 mg/ml, DPT of duration 1–3 days and BAT were not very effective in diagnosing suspected hypersensitivity to ceftriaxone, 4. DPT was not allowed in complex delayed reactions. Further solutions including higher concentrations ceftriaxone for ST and prolonging DPT with ceftriaxone (despite inconvenience) should be investigated in future.

#### P105 Suspected and proved hypersensitivity to sulfonamides among data of Vilnius University Hospital Santaros Klinikos

##### Andrius Kontrimas^1^, Rasa Gauronskaite^2^, Violeta Kvedariene^3^

###### ^1^Vilnius University, Faculty of Medicine, Vilnius, Lithuania; ^2^Clinic of Infectious, Chest diseases, Dermatology and Allergology, Institute of Clinical Medicine, Vilnius, Lithuania; ^3^Vilnius University, Faculty of Medicine; Clinic of Infectious, Chest diseases, Dermatology and Allergology, Institute of Clinical Medicine; Institute of Biomedical Sciences, Department of Pathology, Faculty of Medicine, Vilnius University, Vilnius, Lithuania

**Correspondence:** Andrius Kontrimas - andriuskontri@gmail.com

*Clinical and Translational Allergy* 2018, **8(Suppl 3)**:P105

**Background**

Sulfonamides are present in many different chemical structure drugs carrying the SO_2_–NH_2_ group. The term “sulfa allergy” is therefore misleading. The aim of the study was to investigate allergy reactions of sulfonamide drugs according to data of Vilnius university hospital Santaros klinikos.

**Methods**

1825 ENDA questionnaires from 2000 to 2016 were analysed. Average age of patients was 48.65 (SD ± 14.97) years. Most of patients were females N = 1546 (84.7%). ENDA questionnaires were filled in Pulmonology and Allergology center of Vilnius University Hospital Santaros Klinikos.

**Results**

22 (1.21%) patients had clinical histories (CH) similar to drug allergy to sulfonamides: 20 (1.29%) females and 2 (0.71%) males. 17 (77.27%) patients had reactions related to antibacterial sulfonamides (sulfamethoxazole et trimethoprim), 5 (22.73%)—to other sulfonamides drugs (2—sulfasalazine, 1—indapamide, sulfadiazine, furosemide). Most frequent CH was maculopapular exanthema in 16 cases. Average age of patients was 54.09 (SD ± 13.72) years. The difference between age and sex of all patients with suspected drug hypersensitivity and patients with CH to sulfonamides, was not statistically significant. Only 4 patients were further investigated for sulphanilamide hypersensitivity with culprit drug. Two patients’ results obtained by patch test were negative (sulfamethoxazole et trimethoprim). The other two patients’ results were positive: one by patch test (sulfamethoxazole et trimethoprim), and one by OPT (sulfasalazine).

**Conclusion**

Adverse reactions to sulfonamides were rare. Females were affected more often than males. The most common clinical reaction was maculopapular exanthema. Significant difference of age and sex in sulfonamides group compared to other drugs reactions was not reported.

#### P107 Improving the diagnosis of immediate hypersensitivity to clavulanic acid using synthetic antigenic determinants for basophil activation

##### Tahia D. Fernandez^1^, Maria I. Montañez^1^, Nekane Barbero^2^, Angela Martin-Serrano^1^, Gador Bogas^1^, Adriana Ariza^1^, Ruben Fernandez-Santamaria^1^, Alba Rodriguez-Nogales^1^, Cristobalina Mayorga^1^, Ezequiel Perez-Inestrosa^2^, Maria J. Torres^1^

###### ^1^IBIMA-Regional University Hospital of Malaga-UMA, Malaga, Spain; ^2^Andalusian Center for Nanomedicine and Biotechnology - BIONAND, Malaga, Spain

**Correspondence:** Tahia D Fernandez - tahia.fernandez@ibima.eu

*Clinical and Translational Allergy* 2018, **8(Suppl 3)**:P107

**Background**

Immediate allergic reactions to amoxicillin (AX) and clavulanic acid (CLV) in combination have increased in parallel with an increase in its consumption. Around 30% of all patients reporting an allergic reaction after AX-CLV administration are selective reactors to CLV. Diagnostic work-up includes skin test, with not optimal sensitivity, and drug provocation test, a risky procedure especially in patients suffering severe reactions. Thus, in vitro test must be the rational alternative. However, basophil activation test (BAT) is the only available in vitro assay for diagnosing patients with immediate hypersensitivity to CLV, although its sensitivity is still not optimal. This lack of sensitivity could be due to the deficiencies in the knowledge about CLV degradation pathways and the formation of antigenic determinants. The aim of this study was to identify the antigenic determinant of CLV responsible of the allergic reaction to improve BAT sensitivity for the diagnosis of immediate hypersensitivity to this drug. This was performed designing different synthetic CLV determinants that correspond to the hypothetic fragments of the CLV molecule that would be bound to a protein carrier.

**Methods**

Two potentially antigenic determinants of CLV were hypothesized (AD-I and AD-II) (Fig. 1A) and 3 synthetic molecules of each one were designed: Clav1, Clav2 and Clav3 (from AD-I), presenting aldehyde functionality and Clav4, Clav5 and Clav6 (from AD-II), presenting amine functionality (Fig. 1B). These determinants were tested using BAT in 30 patients with selective reactions to CLV and 20 non-atopic tolerant subjects. The co-expression of CCR3 and CD203c were used to select basophils and the expression of CD63 to determine activation.

**Fig. 1:**
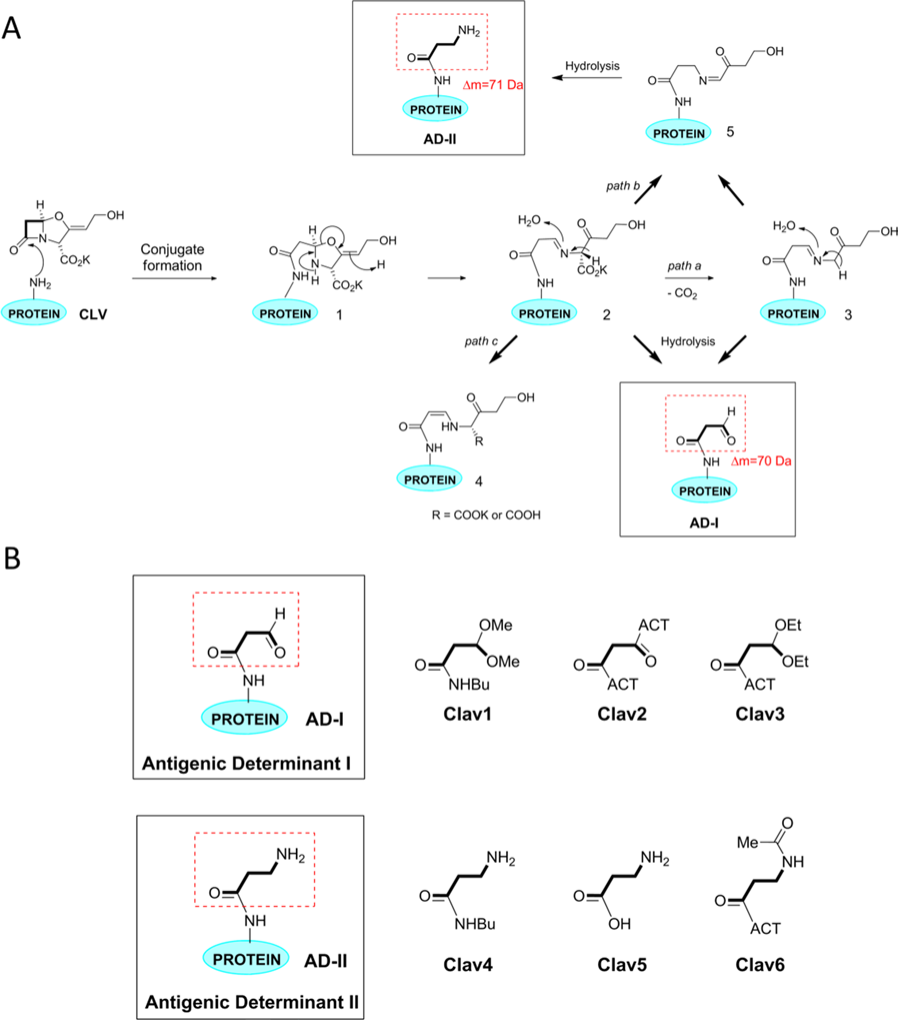
Methods

**Results**

Only CLV and two of the synthetic determinants showed positive BAT results in patients (40% for CLV, 56.7% for Clav2 and 26.7% for Clav3). Combination of CLV and Clav2 results increased sensitivity to 66.6%. Specificity was 88% for the three molecules. Wortmannin treatment, a potent inhibitor of the IgE signaling pathway, significantly reduced the CD63 expression compared to BAT performed without inhibition (p < 0.001 for CLV; p < 0.001 for Clav2; p = 0.007 for Clav3), proving that the observed activation was IgE mediated.

**Conclusion**

AD-I could be one of the determinants involved in CLV immediate allergic reactions. The inclusion of Clav2, a synthetic molecule derived from these determinants, can significantly improve the current BAT sensitivity using the parent drug. These findings may be important for the development of new in vitro tests for diagnosing CLV immediate allergic reactions.

#### P108 Should benzyl-penicilloyl continue to be recognized as the major PCN determinant? Retrospective analysis of positive penicillin skin test determinants

##### Miguel Park

###### Mayo Clinic, Rochester, MN, United States

**Correspondence:** Miguel Park - park.miguel@mayo.edu

*Clinical and Translational Allergy* 2018, **8(Suppl 3)**:P108

**Background**

Penicillin (PCN) skin testing (PST) with the major determinant (benzyl-penicilloyl) and minor determinant mix (penicillin G, benzyl-penicilloate, and benzyl-penilloate) is the primary diagnostic tool in the evaluation of IgE-mediated penicillin allergy. In prior reviews of positive PST, European studies have noted that benzyl-penicilloyl is specifically positive with decreasing frequency. Concurrently, amoxicillin and minor determinants have demonstrated increasingly positive rates in positive PST. In contrast, these findings have not been noted in the North American PST experience. Hence, we conducted this study to determine if benzyl-penicilloyl remains the major determinant in a large U.S. medical center.

**Methods**

Retrospective chart review was conducted for patients with a history of PCN allergy undergoing PCN allergy evaluation from July 1, 2004 to January 22, 2008. Extracted data included rates of positive results to specific major and minor determinants by skin prick (SP) and intradermal (ID) testing. A wheal and flare 3x3 mm greater than the negative control was defined as a positive PST. Positive rates were also determined if a wheal and flare of 5x5 mm or greater is used as a definition of a positive PST. The IRB approved the study, and all subjects signed a written informed consent.

**Results**

6185 patients with a history of PCN allergy underwent PST, and 1.6% (100) had positive PST. Among the positive PST group, the mean age (SD) was 50 ± 22 years and 77% were females. 72% (72 of 100) were positive to only one PCN determinant [26% (19 of 72) benzyl-penicilloyl; 31% (22) PCN G; 22% (16) penicilloate; 21% (15) amoxicillin]. If a positive PST was defined as a 5 × 5 mm wheal or greater, then 58 (1%) of 6185 would have positive PST. Forty-three (74%) of 58 were positive to only one PCN determinant [26% (11 of 43) benzyl-penicilloyl; 48% (16) PCN G; 14% (6) penicilloate; 23% (10) amoxicillin].

Among all the positive PCN determinants used for PST, 106 of 126 (84%) were ID skin test positive compared to 20 (16%) positive SP. Twenty-three (18%) of 126 were positive to benzyl-penicilloyl, 33% (41) PCN G, 26% (33) penicilloate, 23% (29) amoxicillin.

**Conclusion**

Benzyl-penicilloyl may no longer be the major determinant in PST. Penicilloate and amoxicillin are important components of PST. ID PST is more likely to be positive compared to SP PST in patients with a history PCN allergy.

#### P109 Incidence of immediate hypersensitivity reactions to cefazolin in our hospital

##### Jose Julio Martinez^1^, Javier Dionicio Elera^2^, Cosmin Boteanu^2^, Rosario González-Mendiola^2^, Gian Marco Chiarella Privette^3^, Ana Santos^3^, Joaquin Archilla^4^, Isabel Olazabal Olarreaga^5^, Aranzazu Jimenez Blanco^2^

###### ^1^Allergy Unit, Allergy-Anesthesia Unit, Cruz Roja Hospital, Faculty of Medicine, Alfonso X el Sabio University, Madrid, Spain; ^2^Allergy Unit, Cruz Roja Hospital. Faculty of Medicine, Alfonso X el Sabio University, Madrid, Spain; ^3^Allergy Unit, Cruz Roja Hospital, Madrid, Spain; ^4^Allergy-Anesthesia Unit, Cruz Roja Hospital, Madrid, Spain; ^5^Faculty of Medicine, Alfonso X el Sabio University, Madrid, Spain

**Correspondence:** Jose Julio Martinez - josejuliolaguna@gmail.com

*Clinical and Translational Allergy* 2018, **8(Suppl 3)**:P109

**Background**

Betalactam (BL) antibiotics are the most frequently drugs involved in IgE-mediated allergic reactions. Sensitization to cephalosporins are less frequent but are increasing due wide use for preoperative antibiotic prophylaxis. Cefazolin, a first-generation cephalosporin, is commonly used for preoperative prophylaxis in Spain. Subjects with cephalosporin allergy may present positive responses to common penicillin determinants or respond selectively to cephalosporin determinants with a good tolerance of penicillins.

Our first objective was to confirm the incidence of sensitization to cefazolin in our hospital. A second purpose was investigate the cross-reactivity between cefazolin and other betalactam antibiotics.

**Methods**

We evaluated patients with a compatible history of immediate hypersensitivity reaction to cefazolin (in the Cruz Roja Hospital, Madrid) from January 2009 to January 2017.

After obtaining informed consent, we performed:

Skin prick test (SPT) and intradermal test (IDT) using cefazolin from 2 mg/ml up to 20 mg/mL, classic penicillin determinants, amoxicillin, ampicillin and clavulanic acid determinants according ENDA protocol.

Total IgE and specific IgE with penicillin determinants (penicilloyl G, amoxicilloy, penicilloyl V and ampicilloyl). Drug provocation Test (DPT) with cefazolin was performed if cefazolin skin tests were negative.

In patients exhibiting a positive skin or challenge tests with cefazolin, we performed a graded drug challenges with benzylpenicillin and amoxicillin to study cross-reactivity.

**Results**

Forty-nine patients were evaluated, all coming from the perioperative area of our hospital.

We confirmed an immediate hypersensitivity type reaction to cefazolin in 8 patients A total of 4 (50%) patients were diagnosed by skin testing and the rest by DPT.

Skin tests, specific IgE with penicillin and aminopenicillin reagents were negative.

The average of total IgE was 86.1 KU/l, standard deviation (SD) 28.15 KU/l.

Challenge tests with benzylpenicillin and amoxicillin were negative.

From the total, 6 patients were women (75%) and 2 were men (25%). The patients age was between 19 and 75 years old, with an average age of 48.12, SD 12.15 years. The mean time in months from the reaction to the start of the study was 12.15 months, SD 24.43 months.

A total of 5 patients (62.5%) reported urticaria-angioedema and 3 (37.5%) anaphylaxis.

**Conclusion**

We diagnosed 8 patients of immediate hypersensitivity to cefazolin. This represents a 16.3% (8/49) of a total patient studied.

All patients tolerated selectively to cephalosporin determinants with a good tolerance of benzylpenicillin and amoxicillin.

All reactions where cefazolin was involved, occurred in perioperative area.

#### P110 Cefazolin allergy – different sensitization profiles

##### Sofia Martins Farinha, Barbara Kong Cardoso, Elza Maria Tomaz, Filipe Fernando Inácio

###### Centro Hospitalar de Setúbal, Setúbal, Portugal

**Correspondence:** Sofia Martins Farinha - sofiamf_@hotmail.com

*Clinical and Translational Allergy* 2018, **8(Suppl 3)**:P110

**Background**

Cefazolin is a first-generation cephalosporin used for the treatment of a number of bacterial infections and in prevention of group B *Streptococcus* disease in delivery and surgery. How safe is to administer it to patients allergic to penicillin? There is not a consensual answer. The rate of cross-reaction between penicillins and cephalosporins remains to be determined. Cross-reactions seem to be more frequent with 1st generation cephalosporins, but appear to be related to the similarity between R1 side chains. Cefazolin R1 side chain is different from the other cephalosporin and at least one study showed a similar number of reactions to cefazolin in patients allergic to penicillin, comparing to non-allergic. Nevertheless the role of beta-lactam ring metabolites in cross-reactions cannot be entirely excluded. In face of the uncertainty the practice of avoiding cephalosporin administrations to penicillin-allergic patients persists in most of the cases, with impact on the efficacy and/or costs.

**Case report**

Three cases of perioperative anaphylaxis due to cefazolin were referred to our department. Two of them were women, 64 and 17 year-old respectively and the other a 16 year-old male. Skin prick and intradermal tests to cefazolin, major and minor penicillin determinants (PPL and MDM), penicillin, ampicillin, amoxicillin and cefuroxim were performed. Oral challenges were performed when skin tests were negative.

The 3 patients had intradermal positive tests to cefazolin 2 mg/mL.

Two of them had negative skin test to penicillins and cefuroxime and negative oral provocation tests to amoxicillin and cefuroxime.

The 17 year-old woman had a positive intradermal test to MDM and a positive intradermal test to cefuroxime.

**Conclusion**

Patients allergic to cefazolin exhibit different profiles of sensitization to penicillins and other cephalosporins. Positivity to MDM and cefuroxime in one patient support the hypothesis of allergy to cefazolin due to sensitization to beta-lactam ring metabolites in some cases.

Patients allergic to a beta-lactam antibiotic should be tested for cefazolin to decide about its use, especially in perioperative protocols.

**Consent to publish**

Consent to publish was obtained from the patient involved in this study.

#### P111 Penicillin allergy evaluation in the intensive care unit: an opportunity for antimicrobial stewardship

##### Benjamin Cochran, Keith Sacco, Arveen Bhasin, Claudia Libertin

###### Mayo Clinic, Jacksonville, Florida, United States

**Correspondence:** Benjamin Cochran - cochran.benjamin@mayo.edu

*Clinical and Translational Allergy* 2018, **8(Suppl 3)**:P111

**Background**

Reported penicillin (PCN) allergy rarely reflects intolerance. Failure to address PCN allergies results in broader-spectrum drug use. Little data exist on reported PCN allergic patients in intensive care units (ICU) and resulting antimicrobial use. We aim to investigate the current practice of our hospitalists/intensivists initiating antimicrobials in patients with a history of PCN in ICUs of a medium-sized tertiary care hospital in United States.

**Methods**

Using our institution’s electronic medical record, we retrospectively reviewed 50 randomly selected patients admitted to the intensive care unit with reported penicillin allergy from 1/1/2015 to 12/31/2016. Patients were not excluded based on the lack of a suspected infection.

**Results**

The median age was 66.5 years (SD = 15.85); 58% (CI = 43.8–72.17) were female; mean length of stay was 5.16 days (CI = 5.94–9.97); and the median Charlson-Comorbidity Index was 4 (CI = 4.02–6.3). PCN allergy histories were documented as follows: 34% (CI = 22.43–47.85) were reported as rash/hives, 2% anaphylaxis, 6% blistering reaction, 6% itching/swelling, 8% other non-hypersentivity reactions and 44% (CI = 31.16–57.69) as “unknown.” Only 4% (CI = 1.10–13.46) had a documented PCN allergy evaluation by a board certified allergist within 5 years prior to admission, 2% (CI = 0.35–10.5) had an inpatient penicillin allergy evaluation, and 4% (CI = 1.10–13.46) were evaluated by an allergist after discharge. 70% received parenteral antibiotics, of which 30% were only perioperative. The drugs distributed were vancomycin (56% of pts), cephalosporin (14%), aminoglycosides (10%), aztreonam (6%), carbapenem (4%), and pipercillin/tazobactam (8%) [Table 1]. No adverse reactions were reported. Of the patients with a reported PCN allergy who received a beta-lactam, only 1 had a history of rash, while the others were “unknown”.

**Table 1:**
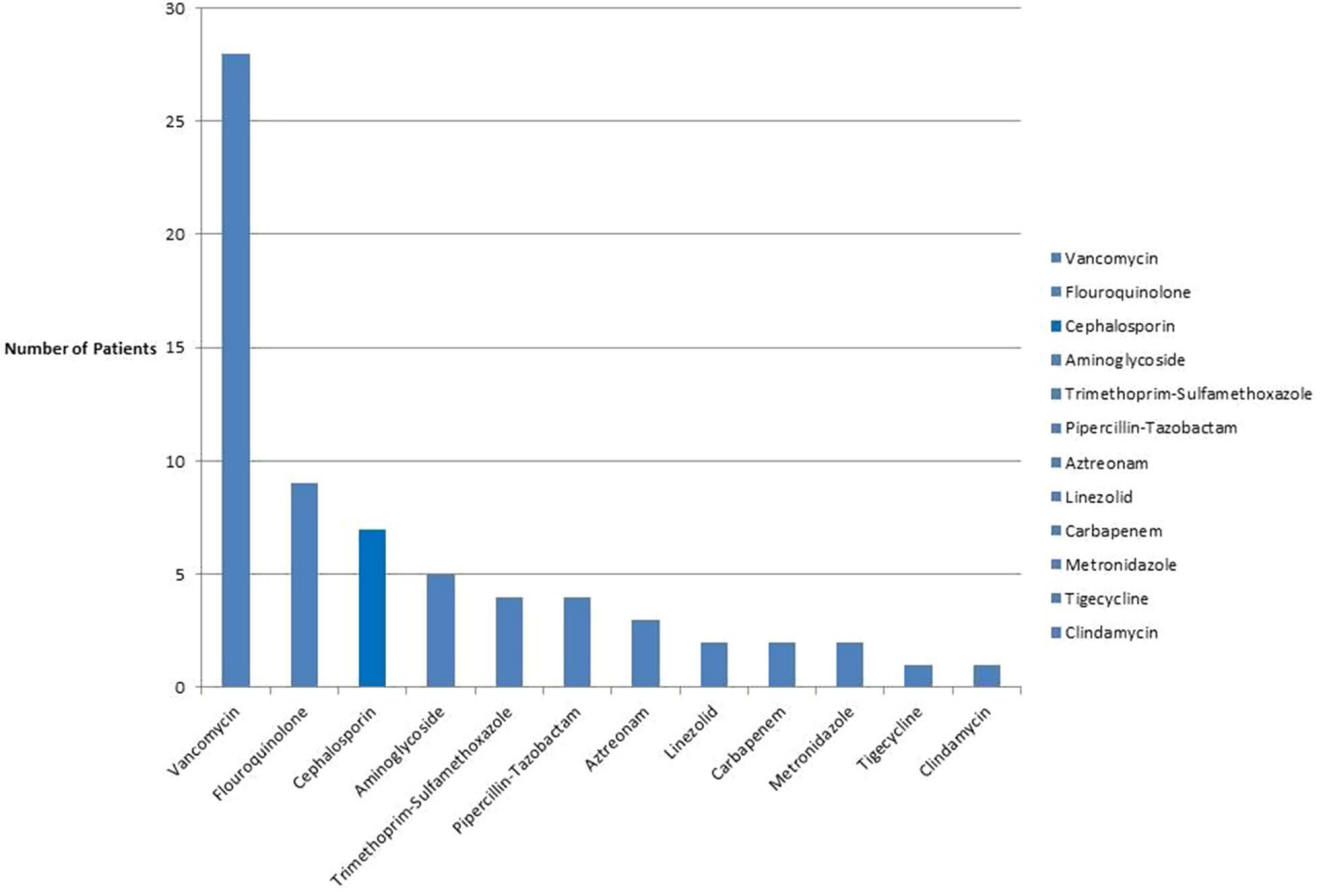
Antimicrobial usage in the intensive care unit in patients with reported penicillin allergy

**Conclusion**

This study reveals that lack of correct PCN reporting dramatically affects antimicrobial selection in the ICU. Opportunities to improve antibacterial stewardship in this setting include education of staff on the types of PCN reactions and how to document appropriately, evaluation of perioperative antibiotic protocols, and development of criteria for allergist consultation in the ICU.

## Friday 20 April 2018

### Clinical cases - Poster Walk 12

#### P113 Incidence of immediate hypersensitivity reactions to cephalosporins in betalactam allergic patients

##### Jose Julio Laguna^1^, Cosmin Boteanu^1^, Rosario González-Mendiola^1^, Agne Ramonaite^2^, Aranzazu Jiménez Blanco^1^, Gian Marco Chiarella Privette^3^, Raquel Fuentes Irigoyen^4^, Irene Carrasco Garcia^3^, Javier Dionicio Elera^1^

###### ^1^Allergy Unit, Cruz Roja Hospital, Faculty of Medicine, Alfonso X el Sabio University, Madrid, Spain; ^2^Republic Klaipeda Hospital (Clinical Fellow EAACI 2017, Allergy Unit, Cruz Roja Hospital, Madrid), Klaipeda, Lithuania; ^3^Allergy Unit, Cruz Roja Hospital, Madrid, Spain; ^4^Pharmacy Department, Cruz Roja Hospital, Madrid, Spain

**Correspondence:** Jose Julio Laguna - josejuliolaguna@gmail.com

*Clinical and Translational Allergy* 2018, **8(Suppl 3)**:P113

**Background**

Betalactam (BL) antibiotics are the drugs most frequently involved in IgE-mediated allergic reactions. Allergy to cephalosporins has mainly been evaluated in the context of patients with confirmed penicillin allergy.

The aim of the study was to assess the incidence of immediate sensitization to cephalosporins in our hospital.

**Methods**

We retrospectively analyzed the medical records of all patients who attended to the Allergy Unit of our hospital, with suspected allergic reaction to BL antibiotics between 1999 and 2017.

After obtaining the patient´s written informed consent, all patients underwent allergy work-up according to the ENDA protocols for immediate reactions:

Skin Tests: prick test (SPT) and intradermal test (IDT) with:

Classical penicillins: penicilloyl polylysine (PPL), minor determinants mixture (MDM: benzylpenicillin and sodium benzylpenicilloate) and benzylpenicillin (penicillin G).

Semi-synthetic penicillins (amoxicillin 20 mg/ml, amoxicillin/clavulanic acid 20 mg/ml and ampicillin 20 mg/ml) and other suspected beta-lactams.

Total IgE and specific IgE: were performed with penicillin determinants (penicilloyl G, amoxicilloy, penicilloyl V and ampicilloyl)

Challenge test: A single-blind challenge test was performed when skin and in vitro test were negative.

**Results**

From a total of 5073 patients initially evaluated, 592 were finally diagnosed of immediate type reactions to BL, 42 of them were allergic to cephalosporins.

A total of 73.8% (31/42) were diagnosed by skin testing and 26.2% (11/42) by challenge test. Twenty-eight patients were women (66.7%) and 14 were men (33.3%) The patients age was between 14 and 84 years old, with an average age of 47.5 and standard deviation (SD) of 15.95 years. The mean time in months from the reaction to the start of the study was 27.7 months and SD of 50.65 months.

A total of 22 patients (52.4%) reported urticaria-angioedema and 20 (47.6%) anaphylaxis.

**Conclusion**

The incidence of immediate sensitization to betalactams antibiotics in our center was 11.7%. Only 7% (42/592) patients with confirmed immediate hypersensitivity reactions to betalactams were allergic to cephalosporins.

From all the patients with suspected reaction after the administration of beta-lactam antibiotics, only 0.83% (42/5073) was confirmed to be allergic to cephalosporins.

#### P115 Test diagnose treat, a pilot study to delabel patients with suspected penicillin allergy

##### Anne Sloan, Niall Conlon, Vyanka Redenbaugh, Patricia Mccrea, Ann Barron, McKeever Aoife, Hanaa Awad

###### St James Hospital, Dublin, Ireland

**Correspondence:** Anne Sloan - asloan@stjames.i.e.

*Clinical and Translational Allergy* 2018, **8(Suppl 3)**:P115

**Background**

Medication allergy and allergy to antimicrobial agents has been long recognized internationally as a significant public health issue. Typically between 10 and 15% of patients self report allergy to medication, most frequently to penicillin. However the true rate of medication allergy is around 1%. The misdiagnosis of medication allergy changes prescription strategies with alternative higher cost drugs being utilized unnecessarily. A recent prevalence survey of 574 in-patients in a large Dublin teaching hospital identified a prevalence rate of 12%. The highest prevalence was amongst the infectious disease and haematology patients.

The project wishes to establish a nurse led “drug allergy and delabelling “service, this will be the first formal project of its nature in an Irish setting.

**Methods**

Utilise NICE and EAACI guidelines to establish and implement a local protocol for the investigation and management of antimicrobial allergy.

The pilot phase will be restricted to infectious disease patients and consults only. Other patients will be dealt with through standard referral pathways. The Drug Allergy Nurse (DAN) will identify 10 patients per month to enroll in the study. This is the pilot phase of the project. Patients with antimicrobial allergy will be identified by the infectious disease team and the drug allergy nurse (DAN) will be informed. A record of the target medication and the regime to be chosen if allergy delabelling is not possible will be made to a priori to allow a cost comparison to be made.

**Results**

A total of 18 patients(n = 18) were recruited in the pilot phase 9 male and 9 female, 2 patients required no testing as they had tolerated penicillin following their reported allergy. 15 patients were tested as per protocol, one patient failed to respond to histamine due to concurrent medications. Of the 18, one patient skin tested positive. Of SPT negative patients n = 14 successfully tolerated a dose of preferred beta lactam as per Microbiology and Infectious disease identifying a cost saving and adherence to good principles of antimicrobial stewardship.

**Conclusion**

The pilot phase has showed proof of principle upon which the roll out of the study will continue. Further efforts will be made to increase recruitment numbers. Demonstrating cost savings and adherance to best practice guidelines as well as positive feedback regarding the testing process and outcomes will allow for the completion of further phases of the project.

#### P116 Value of skin tests in betalactam allergy in patients older than 80 years

##### Teodorikez Wilfox Jimenez Rodriguez^1^, Victor Soriano Gomis^2^, Begoña Cueva Oliver^3^, Jose Manuel Ramos Rincon^4^, María Ruano Zaragoza^5^, Javier Fernandez Sanchez^6^

###### ^1^Allergy Section, Alicante University General Hospital, PhD Program in Public Health, Alicante, Spain; ^2^Medical and Surgical Sciences, Miguel Hernandez University, Alicante, Spain; ^3^Allergy Section. Alicante University General Hospital. Departament of Clinical Medicine, Miguel Hernandez University, Alicante, Spain; ^4^Allergy Section, Alicante University General Hospital, Alicante, Spain; ^5^Service of Internal Medicine, Alicante, Spain; ^6^Alicante University General Hospital. Department of Clinical Medicine, Miguel Hernandez University, Alicante, Spain

**Correspondence:** Teodorikez Wilfox Jimenez Rodriguez - teodorikez@gmail.com

*Clinical and Translational Allergy* 2018, **8(Suppl 3)**:P116

**Background**

The β-lactam allergy is the most reported drug allergy with a prevalence rate of 5–10%. Of those patients who reported being allergic, 95% have a negative allergic study and are able to tolerate penicillins.

As few studies consider patients older than 80 years, the aim of our study was to determine the actual prevalence of allergy to beta-lactams in patients older than 80 years admitted in two units of the Alicante University General Hospital, Spain; and also the value of skin testing in this group of age.

**Methods**

Cross-sectional study, conducted between November 2015 and May 2017. Skin tests (ST) were performed at BPO, MDM (Diater, Spain); penicillin G, amoxicillin, ampicillin, amoxicillin/clavulanic acid, cefuroxime, cefazolin, meropenem and the culprit drug when it was different from those previously mentioned. Specific IgE (sIgE) to penicillin, amoxicillin, ampicillin and cefaclor were carried out by CAP Thermofisher. Controlled challenge was done after negative allergic study. The protocol was approved by the Institutional Research Board.

**Results**

2100 patients older than 80 years were admitted in the units considered for the study. 171 (8.1%) patients reported being allergic to betalactams; 126 (73.7%) accepted to participate in the study and 94 (74.6%) completed the study.

ST were performed on 89 (94.7%) patients; 2 (4.5%) had positive results in the immediate lecture: 1 to amoxicillin and 1 to both, ampicillin and amoxicillin; no delayed responses were observed.

sIgE were performed in 81 (86.2%) patients; 8 (9.9%) were positive to at least one of them, and only in one of the two patients with positive skin tests.

92 (97.9%) patients were challenged with the culprit drug or cefuroxime in a patient with history of allergy to ceftriaxone. 2 patients (with a history of delayed reactions) reacted to the challenge, presenting delayed reactions (maculopapular rash) within 24 h of starting the procedure, 1 to amoxicillin and 1 to cefuroxime, respectively.

The found prevalence of allergy to betalactam antibiotics was 6.6% (IC95% = 1.72–11.56%). Beta-lactam allergy was ruled out in 97.8% of the patients reporting penicillin allergy. The sensitivity, specificity and also the negative predictive value (NPV) in immediate lecture was 100%, whereas, the delayed lecture it was not sensitive to detect allergic patients, the specificity was 100% and the NPV 89.5%.

**Conclusion**

Skin testing was useful for diagnosing IgE-mediated hypersensitivity reactions but not delayed reactions in patients older than 80 years.

#### P117 Involvement of the adaptive immune system in metamizole-induced agranulocytosis

##### Dolores N. Dina^1^, Klara K. Eriksson^2^, Deborah Rudin^3^, Manuel Haschke^1^, Daniel Yerly^2^

###### ^1^Clinic for Rheumatology and Clinical Immunology/Allergology, Bern, Switzerland; ^2^University Hospital of Bern, Bern, Switzerland; ^3^University of Bern, Basel, Switzerland

**Correspondence:** Dolores N Dina - dolores.dina@allergy.unibe.ch

*Clinical and Translational Allergy* 2018, **8(Suppl 3)**:P117

**Background**

Metamizole is a pro-drug with analgesic and antipyretic proprieties. Commonly prescribed in western countries because of its favorable efficacy/toxicity balance, metamizole can cause in rare cases agranulocytosis. The mechanism leading to the disease has not been yet elucidated. Few case reports showing that patients re-exposed to metamizole develop more rapidly and more severely symptoms suggest a possible involvement of the adaptive immune system in metamizole-induced-agranulocytosis (MIA). Objective: In this study, we investigated T cell reactivity upon stimulation with metamizole. We aimed to evaluate the ability of T cells to elicit an immune mediated-toxicity response that could be involved in agranulocytosis pathogenesis.

**Methods**

Patients with history of MIA, metamizole-tolerant donors and unexposed donors were recruited to build a clinical cohort. Peripheral blood mononuclear cell (PBMCs) were incubated with metamizole and derivatives. T cell activation and proliferation were analyzed by flow cytometry using the early activation marker CD69 and the proliferation dye CFSE, respectively. The ability of T cells to secrete pro-inflammatory cytokines (e.g. IFN-g, TNF-a) following metamizole exposure was assessed, as well as the T cell degranulation (CD107a). Finally, we analyzed the direct toxicity induced by metamizole on MIA-patients and controls leucocytes.

**Results**

After 3 days of culture, expression of CD69 was up-regulated in T cells from few MIA-patients and tolerant individuals. Statistical significance was not achieved. Despite IL-2 supplementation and a second restimulation with metamizole, activated cells could not be expanded. Indeed, we did not observe a secretion of pro-inflammatory cytokines or an up-regulation of CD107a upon drug re-exposure. While an immune response against metamizole could not be specifically demonstrated, a direct toxicity upon drug stimulation was observed in all patients and controls. Our results revealed an increased cell death in cultures simultaneously supplemented with IL-2 and exposed to metamizole. In contrast to resting T cells, activated T cells were very sensitive to metamizole toxicity.

**Conclusion**

So far, a T cell response against metamizole could be shown in a minority of patients and tolerant individuals. However the confirmation of this immune response was actually hampered by the high toxicity of metamizole on activated T cells. The reason why cell death of activated T cells was promoted by metamizole is under further investigations.

#### P118 Frequency of cross reactivity to beta lactam antibiotics in Ukrainian children with suspected immediate hypersensitivity reaction to cephalosporins

##### Tetiana Umanets, Vladyslava Barzylovych, Yuriy Antipkin, Volodymyr Lapshyn

###### Institute of Pediatrics, Obstetrics and Gynecology, Kyiv, Ukraine

**Correspondence:** Vladyslava Barzylovych - v.barzylovich@gmail.com

*Clinical and Translational Allergy* 2018, **8(Suppl 3)**:P118

**Background**

Beta-lactam antibiotics (BLA) widely used and often cause allergic reactions in Ukrainian children. The frequency of choice optimal antibiotic therapy in children makes the necessity of cross reactivity investigation between beta-lactam antibiotics very important.

The aim of this study was to evaluate the frequency of crossreactivity to beta-lactam antibiotics in children with suspected immediate hypersensitivity reaction to *cephalosporins* by skin prick test (SPT) and intradermal test (IDT).

**Methods**

71 children (aged from 4 to 16) with suspected immediate hypersensitivity reaction to *cephalosporins*.

SPT and IDT were performed with DAP PPL, DAP MDM, DAP Amoxicillin (20 mg/ml) and DAP Clavulanic (20 mg/ml), and Cefuroxime (2 mg/ml), Ceftriaxone (2 mg/ml), Cefotaxime (2 mg/ml), Ceftazidim (2 mg/ml), Meropenem (1 mg/mL).

**Results**

23.9% children had negative all skin tests. 25.4% had positive skin test with PPL and 9.8% to MDM (1 child had positive PPL + MDM). Sensitization to amoxilin was detected in 18.3% children, to clavulanic acid 4.2%. 54 (76%) children were sensitized to at least one cephalosporin antibiotic—from them 12 children were sensitized to 1 cephalosporin; 34 children to 2 and 8 children to 3 cephalosporins. There were 10.7% children with positive skin tests to meropenem (did not use this drug in the anamnesis).

**Conclusion**

There was cross-reactivity between amoxicillin and chefalosporins in 18.3% of children, and within cephalosporins with identical chains in 59.1% of children with suspected immediate hypersensitivity reaction to cephalosporins.

Considering 10.7% of children with cross reactivity to meropenem, it is rational to add meropenem to the basic testing set for children with suspected allergies to cephalosporins.

#### P119 Beta-lactams neosensitization during phenytoin-induced DRESS syndrome

##### María Andreína Marques-Mejías^1^, Teresa Bellón^2^, Marta Sánchez-Jareño^1^, Ana Fiandor^3^, Elena Ramirez^4^, Rosario Cabañas^3^

###### ^1^Allergy Department, Hospital General Universitario La Paz, Madrid, Spain; ^2^Hospital La Paz Institute for Health Research (IdiPAZ), Madrid, Spain; ^3^Allergy Department, Hospital General Universitario La Paz. Hospital La Paz Institute for Health Research (IdiPAZ), Madrid, Spain; ^4^Farmacology Department. Hospital General Universitario La Paz, Madrid, Spain

**Correspondence:** María Andreína Marques-Mejías - mandreina.marques@gmail.com

*Clinical and Translational Allergy* 2018, **8(Suppl 3)**:P119

**Background**

Previous studies have reported neosensitizations to drugs administered within the acute stage of DRESS syndrome.

**Case report**

A 55-year-old man started treatment with phenytoin after a stroke. Thirty-five days later, he developed generalized exanthema, facial edema, fever, elevated liver enzymes and leukocytosis with eosinophilia. The symptoms lasted more than a month, gradually decreasing in treatment with corticosteroid and after having stopped treatment with phenytoin. Two weeks after initiating treatment with phenythoin and until 4 days after its suspension, the patient received treatment with cefepime that was followed by meropenem for 1 month. During admission the patient also received enoxaparine and metamizole treatment. The patient was diagnosed with definite DRESS syndrome according to RegiSCAR scale criteria. Causality was established with the Algorithm of the Spanish Pharmacovigilance System; Possible (+5) for phenytoin and Conditional (+2) for metamizole and enoxaparine.

A Lymphocyte Transformation Test (LTT) was performed 1.5 months after corticosteroids had been stopped, with positive results with phenytoin (Stimulation Index > 20 in 3 different concentrations) and negative results for metamizol and enoxaparine.

Four months after discharge, he received cefazoline due to a craniotomy. Within 48 h he developed a mild morbilliform exanthema, Tª 37.2 °C without any laboratory tests alterations. A higher dose of corticosteroids was administered with subsequent resolution of the lesions in 3 days. Betalactams and metamizol were suspended at discharge.

One year after the first reaction, skin tests with penicilloyl polylysine (PPL) and minor determinant (MD) were positive in intradermal immediate reading; and negative with penicillin G and amoxicillin. Intradermal tests with cephalosporins (cefepime, cefazoline, and ceftriaxone) were positive within 24 h and positivity persisted for 1 week.

A new LTT was then performed, resulting highly positive for cefazoline, cefepime, and phenytoin and positive for penicillin and meropenem. The patient was diagnosed with definite DRESS syndrome induced by phenytoin and neosensitization to beta-lactams.

**Conclusion**

There have been few cases reported with similar characteristics. In these patients, cross-reactivity was excluded due to the lack of structural similarities between the administered drugs. It could be related instead with a massive non-specific immune activation that translated in increased expression of co-stimulation molecules and pro-inflammatory cytokines. This would lead to a more efficient antigenic presentation and lower immune tolerance to drugs.

We suspect that the sensitization to Cephalosporins took place during treatment with one of them, cefepime, during the acute stage of DRESS syndrome.

Empirical treatments should be avoided during DRESS syndrome due to the high risk of developing neosensitization.

**Consent to publish**

Consent to publish was obtained from the patient involved in this study.

#### P120 Penicillin allergy: a challenging case

##### Tshegofatso Mabelane

###### University of Cape Town, Cape Town, South Africa

**Correspondence:** Tshegofatso Mabelane - tmabelane@hotmail.com

*Clinical and Translational Allergy* 2018, **8(Suppl 3)**:P120

**Background**

There is evidence that there is a decline in the number of positive skin tests in patients with penicillin allergy. We present a patient with negative skin tests who was diagnosed with penicillin allergy confirmed on drug provocation test. References: Macy E, Schatz M, Lin C, Poon KY. The falling rate of positive penicillin skin tests from 1995 to 2007. *Perm J*. 2009; 13:12–8.

**Case report**

54 years old lady presented to Allergy clinic at Groote Schuur Hospital in 2017 with a history of an immediate reaction to Ampicillin which she received for a chest infection as an inpatient the previous year. She experienced a severe reaction of chest tightness and hypotension within 5 min of drug administration. She was treated with adrenalin and antihistamine.

She had a medical history of asthma and allergic rhinitis which were controlled on Seroflo 25/250 bd and Fluticasone nasal spray bd. She had a drug allergy history of NSAIDS hypersensitivity.

She had no family history of penicillin allergy.

Physical examination was normal, with FEV1 of 87%, BP 135/82

Skin prick test: Penicilloylpolylysin (PPL) 0 mm; *Minor determinant mix (MDM) 0 mm; Negative control (normal saline) 0 mm; NAOH control 0 mm; Amoxycillin 0 mm; Histamine 3 mm.

Intradermal test: Penicilloylpolylysin 0 mm; Minor determinant mix 0 mm; Negative control 0 mm.

Ampicillin skin test was deferred due to some reactivity on blood test and recent severe reaction.

*Minor determinant mix composed of sodium benzylpenicillin, benzylpenicilloic acid and sodium benzylpenicilloate, (manufactured in Spain).

CAST ELISA: Pen G 67 pg/mL (50PG/mL); PPL: 0 pg/mL (110 pg/mL); MDM 0 pg/mL (100 pg/mL); Ampicillin 57 pg/mL (70 pg/mL); Amoxicillin 0 pg/mL (100 pg/mL).

The plan was to conduct a drug provocation test to amoxicillin based on negative skin and blood test and a history of tolerating amoxicillin (last used in 2016).

She was challenged to Amoxicillin 500 mg and tolerated1/10^th^ (50 mg) of the dose, however had a severe reaction of chest tightness, wheeze and dizziness within 10 min of receiving the 9/10th (450 mg) dose. Her symptoms resolved on Adrenalin and bronchodilators.

A diagnosis of penicillin allergy was confirmed.

Management: She was given a medic alert indicating her allergy to penicillin and cross reactive cephalosporins. For future use of safe antibiotics, an oral drug challenge to cefuroxime was conducted and was negative.

**Conclusion**

This case has shown the importance of drug provocation test in diagnosing penicillin allergy in patients with negative skin and lab tests. Drug challenges should be performed in specialised units as there is a risk of anaphylaxis.

**Consent to publish**

Consent to publish was obtained from the patient involved in this study.

#### P121 Allergic to beta-lactam patients vs non-allergic: comparison of risk factors

##### Blanca Andrés López, Jaume Martí Garrido, Ramon Lleonart Bellfill, Gemma Vilà Nadal, Mercè Corominas Sanchez

###### Hospital Universitari de Bellvitge, Hospitalet De Llobregat, Barcelona, Spain

**Correspondence:** Jaume Martí Garrido - jaume.marti.garrido@gmail.com

*Clinical and Translational Allergy* 2018, **8(Suppl 3)**:P121

**Background**

Reactions to beta-lactam antibiotics are a major problem in daily clinical practice and a frequent reason for consultation in Allergy Department.

In this study we aimed to determine the differences between patients in whom allergy to beta-lactam antibiotics has been confirmed and those that have been ruled out, and differences between all beta-lactam allergic patients and amoxicillin allergic ones. Also we wanted to find risk factors of being allergic to beta-lactam antibiotic.

**Methods**

360 patients underwent allergologic evaluation (including skin, blood and challenge tests). The 82 diagnosed as allergic patients were compared to the non-allergic ones. We assessed age, sex, symptoms, medical attention, study delay, comorbidities, atopic and other medical background. Bivariate analysis was performed using the *X*^2^ test with the bilateral Fisher test. Risk factors’ data were analyzed by logistic regression (*stepwise* method).

**Results**

Allergic patients had fewer delay to reach the allergologic study (31.1 months compared to 162.6 months for non-allergic patients), more anaphylactic reactions (54.3% vs 0%), more visits to Emergency Department (50% vs 11.7%), more episodes being hospitalized (12.8% vs 3.4%) compatible anamnesis for allergy (72.3% vs 3.4%). Other data (age, sex, comorbidities, allergy to other drugs, and atopic background) showed no significant differences.

Allergic to beta-lactam antibiotic patients were found to be attended more frequently to the Emergency Department than those only allergy to amoxicillin (83.3% vs 52%).

Attending to the analyzed risk factors in the bivariate analysis, we found that suffering anaphylaxis (OR 2269.5), having skin symptoms (OR 10.37) and being attended in the Emergency Department (OR 7.58) were found to be statistically significant (p < 0.001).

**Conclusion**

True allergic patients were found to be 22.8%. Suffering anaphylaxis, having skin symptoms and being attended in the emergency department were found to be risk factors of being allergic to a beta-lactam antibiotic in our study.

## Friday 20 April 2018

### Clinical cases - Poster Walk 13

#### P122 Multicenter retrospective study on NSAIDs hypersensitivity in children

##### Francesca Mori^1^, Marina Atanaskovic-Markovic^2^, Natalia Blanca Lopez^3^, Eva Rebelo Gomes^4^, Francesco Gaeta^5^, Lucrezia Sarti^1^, Marcel M Bergmann^6^, Vladimir Tmusic^2^, Rocco Valluzzi^7^, Jean Christoph Caubet^6^

###### ^1^Allergy Unit, Department of Pediatrics, Anna Meyer Children’s University Hospital, Florence, Italy; ^2^University Children’s Hospital, Medical Faculty University of Belgrade, Belgrade, Serbia; ^3^Allergy Unit, Infanta Leonor University Hospital, Madrid, Spain; ^4^Immunoallergology Department, Centro Hospitalar do Porto EPE, Porto, Portugal; ^5^Allergy Unit, Presidio Columbus, Rome, Italy; ^6^Geneva University Hospitals, pediatric allergy unit, Geneva, Switzerland; ^7^Department of Pediatrics, Division of Allergy, Pediatric Hospital Bambino Gesù, Rome, Italy

**Correspondence:** Francesca Mori - f.mori@meyer.it

*Clinical and Translational Allergy* 2018, **8(Suppl 3)**:P122

**Background**

Non-Steroidal Anti-Inflammatory drugs (NSAIDs) are commonly used in children, and generally considered to be safe. However, hypersensitivity (HS) reactions can occur. Some studies have shown that the prevalence of NSAIDs HS is comparable or even greater than the one reported for betalactam antibiotics HS. However, the exact prevalence remains not well known, as most of the studies are based on clinical history and a complete allergy workup to confirm or exclude a real allergy is lacking. Aim: To identify the real proportion of NSAIDs HS among children with history of reaction, investigated in 6 different centers [Italy (Florence, Rome); Belgrade; Madrid; Porto; Geneva). To describe the NSAIDs most commonly involved in HS reactions.

**Methods**

We retrospectively collected children with history of NSAID reactions who underwent a complete allergy work up in 6 different pediatric Centers.

The drug provocation test (DPT) was performed with up-dosing intakes of the culprit drug (1/100-1/10-2/10-7/10 of the total dose calculated basing on the patient’s weight), every 30–90 min for the first day in a hospital setting.

In all involved centers, the observation period time was of 2–3 h after the last dose. In case of severe reactions suggestive clinical history an anternative NSAID was tested.

**Results**

A total of 693 children (mean age 8.2; range 2 months-18 years) with history of NSAIDs reactions were enrolled. A total of 816 reactions were reported.613 out of 816 (75.12%) were skin reactions (i.e. isolated urticaria or urticaria plus angioedema). In patients with single reactions (in total 574 episodes), the drug most frequently involved was ibuprofen (66.2%).

A total of 661 DPT were performed (526 with the culprit drug and 135 with an alternative drug). One hundred and fifty six out of 661 (23.6%) DPT resulted positive.

**Conclusion**

This study includes a large sample of children from Europe. The diagnosis of NSAIDs HS was confirmed in 19.6% of children by performing a DPT with the culprit drug. The most frequently involved NSAID was ibuprofen.

#### P123 Patient versus allergy specialist interpretation of an allergy workup for suspected iodinated contrast media hypersensitivity

##### Rik Schrijvers^1^, Christine Breynaert^1^, Jean-Luc Bourrain^2^, Pascal Demoly^2^, Anca Mirela Chiriac^2^

###### ^1^Laboratory of clinical immunology, Department of microbiology and clinical immunology, KU Leuven, Leuven, Belgium; ^2^Exploration des Allergies, Département de Pneumologie et Addictologie, Hôpital Arnaud de Villeneuve, University Hospital of Montpellier, Montpellier, France

**Correspondence:** Rik Schrijvers - rik.schrijvers@uzleuven.be

*Clinical and Translational Allergy* 2018, **8(Suppl 3)**:P123

**Background**

Drug allergy workup aspires to validate or invalidate assumed allergies, identify potential cross-reacting drugs and provide safe alternatives. However, the results obtained and information given by the allergist is not always perceived as such by the patient. Therefore, the label of *‘allergy’* often persists for patients despite a negative workup.

**Methods**

In the management of iodinated contrast media (ICM) hypersensitivity reactions (HR), skin testing is used to identify safe skin-test-negative ICMs for potential re-exposure. A recent survey in 597 patients with ICM HR who underwent skin testing during a 13.5-year period at a single centre, evaluated for re-exposure to negatively skin tested ICMs using a standardized questionnaire. At the end of the questionnaire, patients were asked whether they considered themselves as *‘allergic’* to ICM or not. Only physically contacted patients were included (n = 387) and patients with positive skin tests (n = 57) or reactions upon re-exposure despite negative skin tests (n = 13), or incomplete data (n = 18) were excluded, as they could be perceived as *‘allergic’*.

**Results**

In 299 patients with all negative skin tests, 121 (40.4%) were re-exposed, all uneventfully, and 178 (59.5%) were not re-exposed. Patients that were re-exposed (with tolerance) reported *‘not to be allergic’* in 92/121 (76.0%) of cases, *‘allergic’* in 11/121 (9.1%), and *‘uncertain’* in 18/121 (14.9%). Those that were not re-exposed reported *‘not to be allergic’* in 57/178 (32.0%) of cases, *‘allergic’* in 43/178 (24.2%), and *‘uncertain’* in 78/178 (43.8%). The proportion of patients reporting *‘not to be allergic’* was higher in the re-exposed versus not re-exposed group (76.0% versus 32.0%, p < 0.0001 Chi square test), possibly reflecting a change in perception after tolerated re-exposure or a priori a lower threshold for re-exposure in the former group. However, still 54/299 (18.1%) questioned patients were convinced to be *‘allergic’* despite a negative allergy workup and 96/299 (32.1%) remained *‘uncertain’*.

**Conclusion**

Although skin testing can identify safe alternative(s) for ICM re-exposure and potentially discriminate between allergic and non-allergic ICM HRs, the allergist and patient interpretation often remain to differ. It is unclear whether more solid information on the negative predictive value of skin testing in ICM HR at the time of the allergy workup(s) in this study would have reduced the number of patients continuing to perceive themselves as ‘allergic’ or ‘uncertain’. However, our study indicates the need for better dissemination of information of the allergy workup towards patients and health care workers.

#### P124 Characteristics of perioperative anaphylaxis in children

##### Ekaterina Khaleva^1^, Lene Heise Garvey^2^, Amber Franz^3^, Nicola Jay^4^, Henry Tee Bahnson^5^, Gideon Lack^6^, George du Toit^6^

###### ^1^Saint-Petersburg State Paediatric Medical University, Saint-Petersburg, Russia; ^2^Allergy Clinic, Danish Anaesthesia Allergy Centre, Copenhagen University Hospital, Gentofte, Denmark; ^3^Seattle Children’s Hospital, Seattle, WA, United States; ^4^Sheffield Children’s NHS Foundation Trust, Sheffield, United Kingdom; ^5^Benaroya Research Institute, Seattle, WA, United States; ^5^King’s College of London, London, United Kingdom

**Correspondence:** Ekaterina Khaleva - doctor.khaleva@gmail.com

*Clinical and Translational Allergy* 2018, **8(Suppl 3)**:P124

**Background**

Serious allergic events occurring in the peri-operative period are rare, but can rapidly prove life-threatening if not recognised and managed promptly. Available data on perioperative anaphylaxis is largely limited to the adult population; very few studies and case reports have investigated children. The objective of this pilot study was to describe the characteristics of anaphylaxis in the perioperative setting in children.

**Methods**

We performed a retrospective audit of notes from paediatric patients with perioperative anaphylaxis from centres in the United Kingdom and United States and recorded time-dependent symptoms and signs from anaesthetic charts and the results of diagnostic workup.

**Results**

A total of 42 children with perioperative anaphylaxis were included. Mean age was 9 years; 24 (57%) girls, and 18 (43%) boys. Neuromuscular blocking drugs (NMBA) were the most common cause of perioperative anaphylaxis and atracurium was responsible for 18 (43%) of all cases.Based on a modified Ring and Messmer Grading Scale: mild reactions were seen in 3 (7.1%) children; moderate-severe (grade 2–3) in 34 (81%) and 5 (11.9%) children experienced cardiac arrest (grade 4) and required cardiopulmonary resuscitation. The lowest recorded systolic blood pressure (BP) during anaphylaxis was 30 mm Hg (mean 48 SD 24.3) and diastolic BP 10 mm Hg (mean 35 SD 17.6). The mean time of documented hypotension from induction of anaesthesia was 9.4 SD37 min and after administration of the causative drug was 5.7 SD 22 min. Median time from onset of reported hypotension to treatment with epinephrine was 7.6 (IQR, 1–15) minutes. Out of the 42 cases, 23 (54.8%) were accompanied by tachycardia, 26 (61.9%) by respiratory manifestations and 27 (64%) by skin symptoms, mostly manifested as urticaria and flushing. Surgery was abandoned in 38% of cases and the patient was admitted to an intensive care unit in 59.5%.

**Conclusion**

Perioperative anaphylaxis in children is frequently accompanied by severe hypotension and tachycardia. Symptoms typically develop rapidly after the administration of the causative drug, which was most commonly identified as being a NMBA. Skin and respiratory symptoms often accompany circulatory signs in perioperative anaphylaxis in children. Further studies should aim to optimise the identification and management of perioperative anaphylaxis in children.

#### P125 Perioperative management in patients with history of anaphylaxis shock against anesthetic drugs

##### Deshinta Putri Mulya

###### Sardjito Hospital - Gadjah Mada University, Yogyakarta, Indonesia

**Correspondence:** Deshinta Putri Mulya - deshintamulya@yahoo.com

*Clinical and Translational Allergy* 2018, **8(Suppl 3)**:P125

**Background**

Anaphylactic reactions to anesthetic agents vary between 3% and 9%. The heaviest morbidity is in patients with cerebral hypoxia. The identification of appropriate causative agents is often not easy and not always done. Lack of an adequate diagnosis can lead to potentially fatal re-exposure.

**Case report**

A 28-year-old woman with primary infertility and multiple giant myoma uteri is planned for laparotomy of myomectomy with general anesthesia. Three months earlier, when undergoing myomectomy, the patient experienced an anaphylactic allergic reaction with shock at the beginning of anesthesia. At that time the patient received propofol, bupivacaine and fentanyl and cefotaxime prophylactic antibiotics. Due to myoma size and excessive bleeding at the time of menstruation, the patient again planned to do myomektomi. After passing the discussion with anesthesiologist and gynecologist for the surgery strategy, the patient then performed a drug allergy test in the form of skin prick test and intradermal test. From skin prick test and intradermal test it is found out patient has allergic to ketamine and bupivacaine. The patient then underwent surgery 2 days later with fentanyl, propofol, midazolam, rocuronium and received cefotaxime as an empiric antibiotic. The surgery was successfully performed without any allergic reactions or side effects.

**Conclusion**

Proper management of anaphylactic history of anesthesia requires a good multidisciplinary approach between an operator, anesthesiologist and an allergy doctor so that careful preparation of the drug will be used, through appropriate allergy testing.

**Consent to publish**

Consent to publish was obtained from the patients involved in this study.

#### P126 Iodinated contrast media; an approach to cross reactivity

##### Verónica P. López Couso, Alfredo Iglesias Cadarso, Miriam Barrios Albajar, Mar Reaño Martos, Patricia Gonzalez López, Marta Isabel Rodriguez Cabreros, María Blazquez Fernandez, Angelica Gutierrez-Maturana Jimenez

###### Hospital Universitario Puerta de Hierro, Madrid, Spain

**Correspondence:** Verónica P López Couso - vplcouso@gmail.com

*Clinical and Translational Allergy* 2018, **8(Suppl 3)**:P126

**Background**

Hypersensitivity to iodinated contrast media (ICM) it’s rare among drugs; we aim to study cross reactivity among ICM and a safety procedure to manage this patients.

**Methods**

This is a prospective study in which we included 117patients which had an adverse reaction during a computerized tomography (CT) with ICM.

We performed skin prick test (SPT) and intradermal reaction test (IDT). In those with positive IDT we performed CICT (Controlled intravenous challenge test) with one of the negatives in the IDT.

We followed up the patients, to check if they had tolerated a new administration of the ICM we offered as an alternative.

**Results**

From a total of 117 patients 21 had a positive IDT to iomeprol. A CICT with iohexol was tolerated in 20 patients. 19 out of 20 tolerated a new administration. One had an allergic reaction during the CT.

3 had positive IDT to iohexol, we did CICT with iomeprol being positive in one of them. When the two other patients underwent a new CT; one had an allergic reaction.

2 patients had a positive IDT to iohexol and iodixanol. A CICT was positive to iomeprol and negative to iopramide.

**Conclusion**

Regarding our results we can conclude that in patients allergic to iomeprol, iohexol (p < 0.081) can be a safe alternative.

By contrast patients allergic to iohexol, iomeprol doesn’t seem to be an alternative.

Patients allergic to several ICM tolerate iopramide.

Our diagnostic procedure seems to be safe because we didn’t had any severe reaction and useful to find an alternative ICM for each patient.

#### P127 Mastocytosis: NSAIDs are safer than previously thought

##### Tiago Rama^1^, José Mário Morgado^2^, Luis Escribano^3^, Ivan Alvarez-Twose^2^, Laura Sanchez-Muñoz^2^, André Moreira^4^, Alberto Órfão^3^, José Romão^5^, Almudena Matito^2^

###### ^1^Serviço de Imunoalergologia, Centro Hospitalar São João; Faculdade de Medicina da Universidade do Porto, Porto, Portugal; ^2^Instituto de Estudios de Mastocitosis de Castilla La Mancha, Hospital Virgen del Valle; Spanish Network on Mastocytosis (REMA), Toledo And Salamanca, Spain; ^3^Servicio General de Citometría, Centro de Investigación del Cáncer (IBMCC-CSIC/USAL and IBSAL) and Departamento de Medicina, Universidad de Salamanca; Spanish Network on Mastocytosis (REMA), Toledo And Salamanca, Spain; ^4^Faculdade de Medicina da Universidade do Porto; EPIUnit - Instituto de Saúde Pública, Universidade do Porto; Faculdade de Ciências da Nutrição e Alimentação da Universidade do Porto, Porto, Portugal; ^5^Serviço de Anestesiologia, Centro Hospitalar do Porto; Instituto de Ciências Biomédicas de Abel Salazar, Porto, Portugal

**Correspondence:** Tiago Rama - tiagorama@gmail.com

*Clinical and Translational Allergy* 2018, **8(Suppl 3)**:P127

**Background**

Mastocytosis are a group of diseases characterized by an accumulation of clonal mast cells (MC). Nonsteroidal anti-inflammatory drugs (NSAIDs) are frequently avoided in mastocytosis patients, due to safety concerns, as they may elicit mast cell mediated symptoms. In the general population, hypersensitivity reactions to nonsteroidal anti-inflammatory drugs are thought to derive from the depletion of Prostaglandin E2 and resulting Leukotriene release. This study aims to show the prevalence of MC mediator release symptoms triggered by NSAIDs and the associated clinical findings in mastocytosis patients.

**Methods**

Medical records of 554 patiens (417 adults and 137 pediatric) were reviewed. Groups defined by the tolerance patterns to NSAIDs and other cyclooxygenase inhibitors were compared for epidemiological, clinical, laboratorial and imagiological variables.

**Results**

Tolerance patterns to NSAIDs and other cyclooxygenase inhibitors were: 88% of the patients tolerated, 5% were intolerant to all administered drugs, 4% had mast cell-related symptoms caused by one drug, while tolerating other drugs and 3% presented mast cell-related symptoms caused by one drug and avoided others. In the adult sample, intolerance was found to be more frequent in patients with aggressive systemic mastocytosis (p = 0.019), prior anaphylaxis (p = <0.001), multilineage KIT mutation (p = 0.002), diffuse osteosclerosis (p = < 0.001), serum basal tryptase (sBT) levels ≥ 48 ng/mL (p = 0.013), and bone marrow (BM) MC burden ≥ 0.012% (p = 0.006). Positive drug challenges were present in 3/51 and 0/21 of adult and pediatric patients, respectively.

**Conclusion**

The prevalence of hypersensitivity reactions to cyclooxygenase inhibitors (12%) in mastocytosis seems to be higher than in general population, however lower than previously thought. Such reactions are more frequent in patients with forms of the disease with worse prognosis. Furthermore, sBT levels ≥ 48 ng/mL and BM MC burden ≥ 0.012% may be useful surrogate markers to predict for cyclooxygenase inhibitor intolerance.

Mastocytosis should not be considered as a contraindication for the administration of these drugs, although careful evaluation and individualized recommendations are strongly recommended.

#### P128 NSAIDs and anaphylaxis- a case report

##### González-Mendiola Rosario^1^, Jiménez Blanco Aranzazu^1^, Ramonaite Agne^2^, Boteanu Cosmin^1^, Dionicio Elera Javier^1^, Chiarella Privette Gian Marco^3^, Santos Álvarez Ana^3^, Laguna Martínez Julio Jose^1^

###### ^1^Allergy Unit, Hospital Central de la Cruz Roja, Faculty of Medicine, Madrid, Spain; ^2^Allergy Unit, Republic Klaipeda Hospital, Klaipeda, Lithuania; ^3^Allergy Unit, Hospital Central de la Cruz Roja, Madrid, Spain

**Correspondence:** Ramonaite Agne - a.ramonaite@gmail.com

*Clinical and Translational Allergy* 2018, **8(Suppl 3)**:P128

**Background**

Non-steroidal anti-inflammatory drugs (NSAIDs) are well-known augmenting factors in anaphylaxis. In the Mediterranean area, NSAIDs are involved up to 58% of cofactor-induced food-related anaphylaxis episodes. A 49-year-old white female without personal or family history of atopy was referred to our outpatient clinic. She had experienced several anaphylactic reactions to NSAIDS (ibuprofen, diclofenac, paracetamol, metamizole) in the previous 5 years. She had been diagnosed of non-allergic hyperreactivity to NSAIDs in another center.

**Case report**

After obtaining informed consent, we performed single blind oral challenge tests up to the total cumulative doses with: ASA (1000 mg), diclofenac (87.5 mg), paracetamol (2000 mg) and metamizole (2012 mg). The patient rejected performing oral challenge tests with ibuprofen.

Oral provocation tests were negative. We asked the patient if anaphylactic reactions were related to physical effort (walking, running…), alcohol intake, estrogens or specific foods. The patient now related the episodes with exercise and eating wheat-containing food. We performed skin prick tests (SPT) with commercial extracts of common aeroallergens, fruits, nuts, cereals, latex, Anisakis, profilin (Pho d 2), polcalcin-enriched date palm, peach nsLTP (Pru p 3) and determination of total and specific IgE (sIgE) to pollens, cereals, fruits, nuts, latex and O-5-gliadin. The patient rejected performing exercise challenge test. SPT showed positive results to wheat, Anisakis, D. Pteronyssinus, D. Farinae, Olea europaea pollen, Cynodon and nsLTP. sIgE was positive (> 0.35 KU/L) for: Anisakis, peach, hazelnut, rye and omega-5-gliadine. Serum tryptase: 3.58 mcg/L, total IgE: 123 KU/l.

**Conclusion**

We present a case clinically compatible with WDEIA with NSAIDs intake as augmenting factor. This case emphasizes that a carefully and thoroughly taken medical history is of crucial importance, otherwise WDEIA can easily be unrecognized. As a result, non-allergic hyperreactivity to NSAIDs could be excluded and the diagnose of selective allergy to arylpropionic acids was made. Exercise challenge test could not be done in our case.

**Consent to publish**

Consent to publish was obtained from the patients involved in this study.

#### P130 Non-steroidal anti-inflammatory drugs (NSAIDs) hypersensitivity phenotypes and their common triggering medications

##### Mohammed Faizal Bakhtiar^1,2^, Chun Lai Too^3^, Min Moon Tang^4^, Lay Kim Tan^3^, Salsabil Sulaiman^3^, Nurul Aain Ahmad Fauzi^3^, Anastasia Ria Nagum^1^, Giriyappanavar Chandrashekar Rayappa^2^

###### ^1^Allergy Unit, Allergy & Immunology Research Center, Institute for Medical Research, Kuala Lumpur, Malaysia; ^2^Faculty of Medicine, Universiti Kuala Lumpur Royal College of Medicine Perak, Ipoh, Perak, Malaysia; ^3^Immunogenetic Unit, Allergy & Immunology Research Center, Institute for Medical Research, Kuala Lumpur, Malaysia; ^4^Dermatology Department, Kuala Lumpur Hospital, Kuala Lumpur, Malaysia

**Correspondence:** Mohammed Faizal Bakhtiar - drfuzzy73@gmail.com

*Clinical and Translational Allergy* 2018, **8(Suppl 3)**:P130

**Background**

Non-steroidal anti-inflammatory drugs (NSAIDs) are the most consumed drugs worldwide as the first line medication in many diseases, which also causes drug hypersensitivity in susceptible individuals. We investigated the clinical phenotypes of NSAIDs hypersensitivity and their common triggering medication(s) in a local setting.

**Methods**

Sixty-two Malay patients referred for NSAIDs hypersensitivity from 2016 to 2017 were included. Details of NSAIDs hypersensitivity including atopic status, symptoms manifestation, diagnosis and the NSAID(s) causing the reaction(s) were recorded and analyzed. Selective cyclo-oxygenase (COX) 2 inhibitor drug provocation (DP) was performed for known cross intolerant patients, while for unknown cross intolerance, DP to a strong non-selective COX inhibitor was performed.

**Results**

We observed 4 major phenotypes of NSAIDs hypersensitivity namely, NSAIDs induced urticaria/angioedema (NIUA) [n = 39, 63.9% %] followed by NSAIDs exacerbated cutaneous disease (NECD) [n = 6, 9.8%], single NSAIDs induced urticaria/angioedema/anaphylaxis (SNIUAA) [n = 4, 6.6%] and NSAIDs exacerbated respiratory disease (NERD) [n = 1, 1.6%]. Interestingly, 19.7% (n = 12) of these patients were diagnosed with overlapping symptoms which did not fall into the 2013 European Network of Drug Allergy (ENDA) classification. The five most common triggering NSAID was sodium diclofenac (55.7%) followed by mefenamic acid (52.4%), paracetamol (47.5%), ibuprofen (32.8%) and aspirin (29.5%). Of these five medications, sodium diclofenac (58.9%) and paracetamol (51.3%) were the commonly eliciting drugs in NIUA patients. Two third (66.7%) of the NECD patients were triggered by mefenamic acid and paracetamol. while 75% of SNIUAA was induced by mefenamic acid. A total of 63.6% of the overlapping symptoms was induced by sodium diclofenac and mefenamic acid.

**Conclusion**

The current study demonstrates that NIUA is the commonest phenotype of NSAIDs hypersensitivity and was mainly triggered by sodium diclofenac. Additionally, it remains challenging to classify the overlapping symptoms and it warrants consideration to be included as one of the phenotype for NSAIDs hypersensitivity.

## Friday 20 April 2018

### Clinical cases - Poster Walk 14

#### P131 Hypersensitivity reactions to nonsteroidal anti-inflammatory drugs by inhibition of the cyclooxygenase-1–12 years experience

##### Catarina Coutinho^1^, Marta Neto^1^, Manuel Pereira Barbosa^2^

###### ^1^Immunoallergology Department, Hospital de Santa Maria, Centro Hospitalar Lisboa Norte E.P.E., Lisbon, Portugal; ^2^Immunoallergology Department, Hospital de Santa Maria, Centro Hospitalar Lisboa Norte E.P.E.; University Clinic of Immunoallergology, Faculdade de Medicina, Universidade de Lisboa, Lisbon, Portugal

**Correspondence:** Catarina Coutinho - catarinapc@hotmail.com

*Clinical and Translational Allergy* 2018, **8(Suppl 3)**:P131

**Background**

Hypersensitivity reactions to nonsteroidal anti-inflammatory drugs (NSAIDs) were recently reclassified, in order to make more practical and uniform the used nomenclature to define the clinical presentation and to permit a better diagnostic accuracy. Non-immunologically mediated (cross-reactive) reactions were divided in 3 types: NSAIDs exacerbated respiratory disease (NERD), NSAIDs exacerbated cutaneous disease (NECD) and NSAIDs induced urticaria/angioedema (NIUA). In these entities, patients have hypersensitivity to NSAIDS not belonging to the same chemical group, which physiopathology settles in the similarity of the Cyclooxygenase-1 (COX-1) inhibition pattern.

**Methods**

Between the 379 patients referred to the drug outpatient visit with history of hypersensitivity to NSAIDs, from January 2006 to December 2017, we retrospectively analysed 180 patients with confirmed NSAIDs hypersensitivity. The NSAIDs hypersensitivity diagnosis was established by a suggestive clinical history and by skin test (only with metamizole) and/or oral challenge with the culprit NSAID, with acetylsalicylic acid (ASA) or with the alternative NSAID if multiple episodes with reproducible symptoms or anaphylaxis. The patients with selective hypersensitivity to NSAIDs were excluded.

**Results**

In a total of 133 patients, 92 were female, middle age 47 ± 14.98. The most frequently suspect NSAID was ASA, followed by ibuprofen (75/72). Hypersensitivity reactions were immediate in 69 (52%) and late in 64 patients (48%). We performed 97 alternative challenges and 36 diagnostic challenges. We identified 93 (70%) patients with NIUA, 18 (14%) with NERD and 21 (16%) with NECD. In the NIUA type of reaction, besides tolerating selective COX-2 inhibitors, 79 (85%) patients also tolerated weak COX-1 inhibitor—paracetamol. Also in NIUA, 49 (53%) patients had immediate reaction, 26 (53%) of them with anaphylaxis as clinical presentation.

**Conclusion**

The majority of the patients were women. The global number of alternative oral challenges was almost threefold of the diagnostic ones. NIUA was the principal type of reaction found, with more than a half of the patients with immediate reaction. We also emphasize the percentage of anaphylaxis in NIUA and the fact of only a small percentage (15%) of patients in this group didn’t tolerate weak COX-1 inhibitor (paracetamol). We discuss the relevance of the diagnostic tools in the correct diagnosis of hypersensitivity to NSAIDs.

#### P132 Selective hypersensitivity to codeine

##### María Del Valle Campanón-Toro, Esther Moreno-Rodilla, Francisco Javier Muñoz-Bellido, Elena Laffond-Yges, Eva Macías-Iglesias, Miriam Sobrino-García, Ignacio Dávila-González, Maria Teresa Gracia-Bara, Sonia De Arriba-Méndez, Milagros Lázaro-Sastre

###### Complejo Asistencial Universitario de Salamanca, Salamanca, Spain

**Correspondence:** María Del Valle Campanón-Toro - mvallect@gmail.com

*Clinical and Translational Allergy* 2018, **8(Suppl 3)**:P132

**Background**

Codeine is an opioid agonist that is usually prescribed for its analgesic and antitussive effects. Hypersensitivity reactions to codeine are rarely described; most of them are induced by non-immunological induction of histamine release. Delayed reactions, which include generalized maculopapular eruptions, fixed drug eruptions, or toxic epidermal necrolysis are even more infrequent. Cross-sensitization among structurally related opium alkaloids has been described, which involve avoiding useful drugs in surgery and in the treatment of pain.

**Methods**

We evaluated 5 patients with suggestive reactions of hypersensitivity to codeine. Four patients had immediate reactions and 1 patient had a delayed reaction (Table 1).

**Table 1:** Characteristic of patient and test performed

	Patient 1	Patient 2	Patient 3	Patient 4	Patient 5
**Symptoms**	Urticaria (x2)	Urticaria, angioedema and wheezing (x2)	Urticaria (x2)	Urticaria	Exanthema
**Latency**	Immediate	Immediate	Immediate	Immediate	Delayed
**SPTs codeine**	Positive	Positive	Positive	Negative	ND
**SPTs & IDTs fentanyl**	Negative	Negative	Negative	Negative	ND
**PT codeine**	ND	ND	ND	ND	++
**PT morphine**	ND	ND	ND	ND	Negative
**SBPCC codeine**	ND	ND	ND	Urticaria (x2)	ND
**SBPCC morphine**	Negative	Negative	Negative	Negative	Negative
**SBPCC fentanyl**	Negative	Negative	Negative	Negative	Negative

ND: not done

Skin prick tests (SPT) with codeine and SPTs and intradermal tests (IDT) to fentanyl were performed. Patch tests (PTs) were performed in the delayed reaction. In addition, single blind placebo controlled challenges (SBPCC) with fentanyl and morphine were performed. As codeine has histamine release properties, SPTs with codeine were performed in 14 healthy controls.

**Results**

SPTs with codeine were positive in three patients, but also in 64% of the control subjects. The 3 patients that had had more than one reaction suggestive of hypersensitivity to codeine were considered positive. The patient who had only one immediate reaction underwent a SBPCC to confirm the diagnosis of codeine hypersensitivity. The patient with the delayed reaction had a positive PT with codeine. SBPCCs with subcutaneous fentanyl and oral morphine were negative in all patients. All patients were advised to avoid codeine, but they were allowed to receive morphine, fentanyl and other non-structurally related opioids.

**Conclusion**

We present 5 patients with a confirmed diagnosis of codeine hypersensitivity with good tolerance to morphine and fentanyl.

SPTs were not helpful in the diagnosis of codeine hypersensitivity, due to a high rate of false positive results. A negative skin test with fentanyl was associated with good tolerance.

Further studies are necessary to investigate the pathophysiology of these reactions.

#### P133 Is oral aspirin desensitization safe? Regular clinical practice experience during oral aspirin desensitization

##### Marina Lluncor^1^, Marta Sanchez-Jareño^1^, Irina Bobolea^2^, Ana Fiandor^1^, Pilar Barranco^1^, Javier Domínguez-Ortega^1^, Santiago Quirce^1^, Rosario Cabañas^1^

###### ^1^Allergy Department, Hospital La Paz Health Research Institute (IdiPaz), Madrid, Spain; ^2^Pulmonology Department, Hospital Clinic Barcelona, Barcelona, Spain

**Correspondence:** Rosario Cabañas - charo.cabanas@gmail.com

*Clinical and Translational Allergy* 2018, **8(Suppl 3)**:P133

**Background**

Aspirin desensitization is often effective in aspirin-exacerbated respiratory disease (AERD), reducing the need of corticosteroids and nasal surgery. Objective: to describe the adverse reactions during the oral aspirin desensitization (OAD) and to compare them with those of the diagnostic aspirin challenge.

**Methods**

Clinical data of adverse reactions occurred during diagnostic bronchial/oral aspirin challenge (BAC/OAC) and subsequent OAD performed in the Allergy department in AERD patients were retrospectively collected and compared. A 4-day desensitization protocol adapted after the original proposed by Stevenson was used. It started at 25 and 50 mg of aspirin and proceeded to 75 mg and 100 mg on day 2; 150 and 320 mg on day 3 and 650 mg the 4^th^ day. The two daily doses were separated by 120 min. The reactive dose was repeated until tolerance. All patients received during the desensitization LABA/inhaled corticosteroids and montelukast, and if needed, oral corticosteroids.

**Results**

A total of 14 reactions during 11 OAD in 9 AERD patients were collected. Mean age was 46 yr, 5 males, 4 females. AERD had been previously diagnosed by BAC in 4 patients and by OAC in 5 patients. The reaction during the OAC occurred 30–165 min after an accumulated aspirin dose of 150–175 mg in 3 patients (2 nasal symptoms, 1 ocular symptom, and 2 dyspnea) and 1 patient, 60 min after an accumulated dose of paracetamol 1 g had a 27% FEV_1_ decrease. The treatment used was antihistamines (3), nasal vasoconstrictors (2), beta2-adrenergic agonists (4), oxygen (2) and intravenous corticosteroids (4). The first reaction on OAD occurred 90 min (range 60–120 min) after the desensitization had started, after an accumulated dose of 75 mg in all patients. Seven patients had a 7% (mean) decrease in FEV_1_ (range 5–14%), 9 naso-ocular symptoms, and 4 cough. Three patients had a second reaction during desensitization, after an aspirin accumulated dose of 175 mg in 2 patients (one had a 30% decrease in peak flow and the other only nasal symptoms) and one patient after 120 mg AAS presented nasal symptoms. Treatments used were: antihistamines(9), beta2-adrenergic agonists(6), nasal vasoconstrictors(7) and intravenous corticosteroids(2). After the second reaction, 2 patients required treatment, one nasal vasoconstrictors, and the other one also antihistamines and inhaled corticosteroids.

**Conclusion**

We describe 14 reactions during 11 aspirin desensitizations in 9 AERD patients. These reactions were mild and less severe than during diagnosis procedure. Aspirin desensitization is a safe technique. Baseline treatment seems to prevent severe reactions.

#### P134 Hypersensitivity to NSAIDs in different age ranges

##### Natalia Blanca-Lopez, Diana Pérez-Alzate, Maria Luisa Somoza, M. Antonia Rojas, Francisco Javier Ruano, Elisa Haroun, Maria Vazquez De La Torre, Maria Garcimartin, Miguel Blanca, Gabriela Canto

###### Infanta Leonor - University Hospital, Madrid, Spain

**Correspondence:** Natalia Blanca-Lopez - natalia.blanca@gmail.com

*Clinical and Translational Allergy* 2018, **8(Suppl 3)**:P134

**Background**

Hypersensitivity (HS) reactions to NSAIDs are the most common cause of drug hypersensitivity in most countries at all age ranges. So far five different phenotypes have been defined, with most of the data published in adults, very few in children and none focused specifically in adolescents. Our aim was to define the type of reactions in a population of adolescents in a large allergic center.

**Methods**

Subjects from 15 to 25 y.o. with suspicion of NSAIDs HS were evaluated in a 6 years period time (2013–2017). Diagnosis was established by a detailed clinical history and oral provocation test (OPT) with ASA. If negative, we challenged with the culprit drug If ASA was the culprit, the OPT was done with ibuprofen. Atopic status was assessed with skin testing to a panel of inhalant allergens. Clinical entities were classified in three categories: cutaneous symptoms, cutaneous plus respiratory symptoms and respiratory symptoms. Statistical Analysis was made by X^2^ and T student.

**Results**

A total of 100 subjects were included, being 60% females (mean age 19.8, median 19.0). From the total, 79% referred cutaneous symptoms, 5% respiratory symptoms, and the remaining 16% cutaneous plus respiratory symptoms. The NSAIDs involved were: ibuprofen (57%), dypirone (11%), dexketoprofen (10%), paracetamol (10%), ASA (8%), naproxen (3%) and diclofenac (1%).

After the allergological evaluation, in 44% of cases the diagnosis of HS was confirmed, having 34 patients cross intolerance and 10 subjects a selective response (6 = dipyrone, 3 = ibuprofen and 4 = paracetamol.

When comparing HS vs tolerants we found statistical differences in the clinical entity with more anaphylaxis and respiratory symptoms in the cases and more cutaneous symptoms in the **control** group (p < 0.001), and in atopic status, being the 65% of cases positive to at least one aeroallergen (p = 0.01). We also found differences when we analysed time interval between drug intake and symptoms, although it was not significant (p = 0.05), being this time interval in cases shorter (< 1 h) than in controls.

We did not found differences in gender age, drug involved indication of treatment and number of episodes.

**Conclusion**

In our study almost the 50% were confirmed as having HS to NSAIDs. Patients finally diagnosed of HS had more anaphylaxis and respiratory symptoms, and were more atopic than subjects having good tolerance, being significant these differences. Although the time interval between drug intake and appearance of symptoms was shorter in allergic, this these differences did not reached significance.

#### P135 Comparison of NSAID hypersensitivity between children and young adults including adolescents

##### Diana Perez-Alzate, Natalia Blanca-Lopez, Maria Luisa Somoza, Montserrat Onieva, Elisa Haroun, Francisco Javier Ruano, Maria Garcimartin, Maria Vazquez De La Torre, Miguel Blanca, Gabriela Canto

###### Infanta Leonor - University Hospital, Madrid, Spain

**Correspondence:** Diana Perez-Alzate - dianavictoria.perez@salud.madrid.org

*Clinical and Translational Allergy* 2018, **8(Suppl 3)**:P135

**Background**

Although hypersensitivity reactions to NSAIDs are the first cause of these type of reactions few studies have been carried out focusing on populations stratified by age. Previous studies indicate that manifestations like facial angioedema are more frequently observed in children with asthma induced by NSAIDs. Our aim was to compare a group of children versus a group of young population evaluated in our Allergy Unit with confirmed NSAIDs hypersensitivity.

**Methods**

Two group of patients with suspicion of NSAIDs were evaluated in a 6 years period (2013–2017). GroupA) Children from 2 to 14 y.o and groupB) Those from 15 to 25 y.o. In all cases diagnosis was established by a clinical history and oral provocation test (OPT) with ASA. If negative, we challenged with the culprit NSAID. In those cases were ASA was the responsible, OPT was done with ibuprofen. Atopic status was established by skin testing to a panel of inhalant allergens. Clinical entities were classified in three categories: cutaneous symptoms, cutaneous plus respiratory symptoms and respiratory symptoms. Statistical Analysis was made by X2 and T student.

**Results**

A total of 216 patients were included: GroupA: 116 children and GroupB: 100 adolescents. We observed significant differences between males/females, with more male in childrens (53.8%) (p = 0.01). In Group A 60% referred cutaneous symptoms, 25% respiratory symptoms, and the remaining 20% cutaneous and respiratory symptoms. In Group B 59% referred cutaneous symptoms, 11.3% respiratory symptoms, and the remaining 29.5% cutaneous plus respiratory symptom. (p = 0.6). Significance was observed in the indication of previous treatment with NSAIDs: infections in children and pain in adolescents (p < 0.0001). After the allergological work-up, in 25.8% children and 44% of adolescents, the diagnosis of HS was confirmed, being 83% cross intolerance and 16.6% selective reactions in Group A, and 77% of cross intolerance and 22.7% in Group B. The NSAIDs involved in group A were: ibuprofen: (46%), ASA: 10%, dypirone: 6.7%, dexketoprofen: 3.3%, paracetamol: 3.3%; Group B: ibuprofen: 54%, dypirone: 18.2%, ASA: 9%, dexketoprofen: 6.8% and paracetamol: 4.5%.

**Conclusion**

Significant sex differences between hypersensitivity reactions to NSAID in children(A) versus adolescents/young adults(B) occurs with predominance of males in the first group.

Although there was a predominance of anaphylaxis in adolescents and respiratory symptoms in children, we did not found significant differences further studies are in progress for analysing in more details this differences including an increase in our study group number.

There were significant differences in the indication of treatment with NSAIDs, because de are more prescribed for pain in adolescents and for infections in children.

#### P136 Factors influencing Aspirin treatment response in AERD patients

##### Elina Jerschow^1^, Matthew Edin^2^, Fred Lih^2^, Artiom Gruzdev^2^, J Alyce Bradbury^2^, Teresa Pelletier^1^, Simon Spivack^1^, Victor Schuster^1^, David Rosenstreich^1^, Darryl Zeldin^2^

###### ^1^Montefiore Medical Center/Albert Einstein College of Medicine, Bronx, NY, United States; ^2^NIEHS, Research Triangle Park, NC, United States

**Correspondence:** Elina Jerschow - ejerschow@yahoo.com

*Clinical and Translational Allergy* 2018, **8(Suppl 3)**:P136

**Background**

Instead of the expected improvement, some patients with aspirin exacerbated respiratory disease (AERD) develop worsening of respiratory symptom during the standard of care treatment for AERD—aspirin desensitization followed by a high-dose aspirin treatment.

**Methods**

Aspirin treatment (650 mg orally twice daily for 4 weeks) response was defined by a ≥ 3-point change from baseline in Asthma Control Test (ACT) or ≥ 0.5 point change in Rhinoconjunctivitis Quality of Life Questionnaire (RQLQ) score. We compared aspirin-induced changes in three groups of participants: 1. AERD patients who benefited from aspirin treatment for 4 weeks (“responders”, N = 9); 2. AERD patients whose respiratory symptoms worsened on aspirin (“non-responders”, N = 8); 3. Healthy volunteers (N = 10) who were treated with aspirin the same way as the AERD patients. We analyzed the association between aspirin responsiveness and the following markers: total serum immunoglobulin E (IgE), peripheral eosinophilia, plasma 15-hydroxyeicosatetraenoic acid (15-HETE), 15-HETE chirality, and atopy (defined by either positive skin test to environmental allergens or increased serum specific IgE levels).

**Results**

The non-responders had higher baseline serum IgE levels than the responders (310 kU/L, IQR 102–937, vs. 107 kU/L, IQR 69–284, *p *= 0.02) and IgE levels significantly increased during aspirin treatment only in the non-responders (from 310 kU/L, IQR 102–937 to 472 kU/L, IQR 237–1310, *p *= 0.01). The non-responders were more likely to be atopic than the responders (*p *= 0.03). After 4 weeks of aspirin treatment, there was a significant correlation between blood eosinophil count and 15-HETE in AERD responders to aspirin treatment, *r *= 0.92, *p *= 0.008 while there was no significant correlation between 15-HETE and eosinophil count in non-responders, *r *= 0.1, *p *= 0.7. Peripheral blood eosinophil counts correlated with 15-hydroxyeicosatetraenoic acid (15-HETE) plasma levels after taking aspirin for 4 weeks only in healthy volunteers, *r *= 0.79, *p *= 0.006, but not in AERD patients. In all groups, the predominant 15-HETE isomer at baseline was 15S-HETE. After 4 weeks of aspirin treatment, the non-responders tended to have significantly higher levels of both 15S- and 15R-HETE (p < 0.001 for both) but there were no significant changes in either chirality or the plasma levels of 15-HETE in AERD responders or healthy volunteers.

**Conclusion**

AERD patients who are atopic and have higher baseline IgE levels tend to have worsening in respiratory symptoms during aspirin treatment and develop a simultaneous increase in total IgE levels and in plasma 15-HETE. This study indicates that the presence of atopy and higher serum IgE levels may contribute to the aspirin treatment outcomes.

#### P137 Paracetamol hypersensitivity – single vs multiple NSAID reactors

##### Maria João Vasconcelos, Leonor Carneiro-Leão, Fabrícia Carolino, Eunice Dias-Castro, Josefina Cernadas

###### Allergy and Clinical Immunology Department, Centro Hospitalar de São João, EPE, Porto, Portugal

**Correspondence:** Maria João Vasconcelos - mariajoaosvasconcelos@gmail.com

*Clinical and Translational Allergy* 2018, **8(Suppl 3)**:P137

**Background**

Hypersensitivity (HS) to paracetamol is rare despite its widespread use. Most cases occur in multiple nonsteroidal anti-inflammatory drugs (NSAIDs) reactors. Cases of selective HS to paracetamol can occasionally be found but the underlying mechanism is poorly understood. Objective: To assess the clinical features and management of patients with suspected HS to paracetamol, comparing single vs multiple-reactors.

**Methods**

Clinical records of all patients referred to our Drug Allergy Unit for suspected paracetamol HS (2010–2017) were reviewed.

**Results**

Sixty-five patients were included (55% female); median age at first reaction [interquartile range] was 36 [23–51] years; 14% were < 18 years. The time elapsed between the reaction and diagnostic work-up ranged from 1 month to 25 years. Pain was the main reason for paracetamol’s administration (29%). All patients reported prior tolerance, except an infant with no previous exposure. The reaction was immediate in 46% of cases. Urticarial exanthema occurred in 54% of patients and isolated angioedema in 71% of cases. Twelve cases of anaphylaxis were reported.

Paracetamol was considered to be implicated in 41 (63%) of cases (7 as single and 34 as NSAID multiple reactor).

Both groups were similar regarding gender distribution (p = 0.233), atopy (p = 0.101), reaction time (p = 0.659), age of onset (p = 0.119) and severity of the reactions—anaphylaxis vs mucocutaneous signs (p > 0.999).

**Conclusion**

We found single reactors to paracetamol to be clinically similar to those also reacting to multiple NSAIDs. However these results need to be prospectively validated in larger samples.

#### P138 Hypersensitivity to paracetamol: placebo effect or overdiagnosis?

##### Rebeca Mussi Brugnolli, Cláudia Castilho Mouco, Andressa Zanandréa, Rafaella Gaia Duarte, Marcelo Vivolo Aun

###### University of São Paulo, São Paulo, Brazil

**Correspondence:** Marcelo Vivolo Aun - marcelovivoloaun@gmail.com

*Clinical and Translational Allergy* 2018, **8(Suppl 3)**:P138

**Background**

Paracetamol is considered safe in patients with urticaria or angioedema triggered by nonsteroidal anti-inflammatory drugs (NSAIDs). However, there are patients who also report a reaction to this drug and they end up with no therapeutic option. We aimed to evaluate the frequency of reactions to acetaminophen in patients intolerant to NSAIDs who also reported reactions to paracetamol.

**Methods**

We retrospectively analyzed patients’ medical records from 2005 to June 2017. Patients with chronic urticaria exacerbated by NSAIDs (NECD) or urticaria/angioedema induced by NSAIDs (NIUA) who reported intolerance to paracetamol were submitted to single blind placebo controlled drug provocation test (DPT) with paracetamol (dose 1 drop/kg body weight, up to 500 mg). Individuals with NSAIDs exacerbated respiratory disease or suspicion of reaction to a single group of NSAIDs (immediate or delayed) were excluded. We evaluated demographic characteristics, clinical features triggered by the drugs in the referred reactions and the results of the DPT with paracetamol.

**Results**

A total of 122 patients (77% women) were evaluated, with a mean age of 38 years, 28 (23.9%) with NECD and 94 with NIUA. Thirteen patients presented a reaction during DPTs (10.6%), including 9 (7.4%) due to paracetamol and 4 (3.2%) reactions to placebo. Of the patients with objective reactions to acetaminophen, 6/9 presented urticaria, 2/9 with angioedema and 1/9 had urticaria and angioedema. The placebo reactors presented itchiness without skin lesions (2/4) and urticaria (2/4). There was no anaphylaxis.

**Conclusion**

Paracetamol has been shown to be safe in patients with urticaria/angioedema induced or exacerbated by NSAIDs, even when they reported a previous reaction to it. Among the positive challenges, 1/3 were reactions to placebo, showing the relevance of psychologic factor and the need to use placebo before the active drug during DPTs.

#### P139 Piroxicam-induced fixed drug eruption: cross-reactivity to Meloxicam

##### Haifa Ben Romdhane, Najah Ben Fadhel, Helmi Ammar, Zohra Chadli, Nadia Ben Fredj, Amel Chaabane, Naceur Abdelsattar Boughattas, Karim Aouam

###### Laboratory of Pharmacology, Faculty of Medicine of Monastir, University Hospital, University of Monastir, Monastir, Tunisia

**Correspondence:** Karim Aouam - aouam_k@yahoo.fr

*Clinical and Translational Allergy* 2018, **8(Suppl 3)**:P139

**Background**

Fixed drug eruption (FDE) is a common cutaneous adverse reaction, characterized by the recurrence of single or multiple round erythematous plaques that typically regress leaving residual hyperpigmentation. Non-steroidal anti-inflammatory drugs are one of principal cause of FDE.A few cases of piroxicam-induced FDE have been reported, however the cross-reactivity among oxicams has been rarely evaluated. Aim: We report three cases of piroxicam-induced FDE with cross-reactivity to meloxicam.

**Methods**

We included all cases of piroxicam-induced FDE with a cross reactivity to meloxicam diagnosed in our department of pharmacovigilance of Monastir. The drug imputability of FDE was established according to Begaud and *al* method. Patch tests for piroxicam and meloxicam were performed in the involved skin according to the ENDA recommendations: 10% in petrolatum for both piroxicam and meloxicam. Rechallenge test was performed for patients with negative skin tests, by administering 15 mg of meloxicam for three consecutive days.

**Results**

Three patients (one man and two women) aged between of 26 and 44 years were enrolled. The FDE was multiple for two patients and solitary for one. No bullous presentation was noticed in these patients. The most common sites were arms. The time between drug intake and eruption onset was 48 h in all cases. Patch test for piroxicam performed on the involved skin was positive in all patients. To assess cross-reactivity to meloxicam in our patients, we have performed a patch test for this drug. The test was positive in only one patient. An oral provocation test with meloxicam was positive in the two patients with negative patch test.

**Conclusion**

We have demonstrated a potential cross reactivity to meloxicam in patients with piroxicam-induced FDE. Clinicians should be aware that meloxicam is not a safe alternative and avoid its prescribing in that case.

## Friday 20 April 2018

### Clinical cases - Poster Walk 15

#### P140 Assessment of quality of life by means of specific questionnaire, in patients with suspected allergic drug reaction. Comparison before and after allergological study

##### Gabriel Gastaminza^1^, Miguel Ruiz-Canela^2^, Mª José Barasona-Villarejo^3^, Rosario Cabañas^4^, Ignacio García-Núñez^5^, José Julio Laguna^6^, Teófio Lobera^7^, Marta López-San Martín^8^, Joaquín Martín-Lázaro^9^, Ruth Mielgo^10^, Esther Moreno^11^, M Carmen Moya^12^, Nancy R. Ortega-Rodríguez^13^, Patricia Rojas-Perez-Ezquerra^14^, Ana Rosado^15^, María Salas^16^, Leticia Sánchez-Morillas^17^, Concepción Vila^18^, Mercè Corominas^19^

###### ^1^Clinica Universidad de Navarra, Pamplona, Spain; ^2^Dpto. Medicina Preventiva y Salud Pública, Universidad de Navarra, Pamplona, Spain; ^3^Hospital Universitario Reina Sofía, Córdoba, Spain; ^4^Hospital La Paz Health Research Institute (IdiPAZ), Madrid, Spain; ^5^Hospital Quironsalud Campo de Gibraltar, Cádiz, Spain; ^6^Hospital Central Cruz Roja, Madrid, Spain; ^7^Hospital de San Pedro, Logroño, Spain; ^8^Hospital Universitario Puerta de Hierro-Majadahonda, Madrid, Spain; ^9^Hospital Universitario Lucus Augusti, Lugo, Spain; ^10^Hospital Universitario 12 de Octubre, Madrid, Spain; ^11^Complejo Asistencial Universitario de Salamanca, Salamanca, Spain; ^12^Complejo Hospitalario Torrecárdenas, Almería, Spain; ^13^Hospital Universitario de Gran Canaria Dr. Negrín, Las Palmas De Gran Canaria, Spain; ^14^Hospital General Universitario Gregorio Marañon, Madrid, Spain; ^15^Unidad de Alergia, Hospital Universitario Fundación Alcorcón, Madrid, Spain; ^16^Hospital Regional Universitario de Málaga, Málaga, Spain; ^17^Hospital Clínico San Carlos, Madrid, Spain; ^18^Hospital Universitario Severo Ochoa, Madrid, Spain; ^19^Hospital Universitari de Bellvitge, L’hospitalet De Llobregat, Spain

**Correspondence:** Gabriel Gastaminza - gastaminza@unav.es

*Clinical and Translational Allergy* 2018, **8(Suppl 3)**:P140

**Background**

Suspicion of allergic drug reaction (ADR) can cause important disturbances in the patient’s life. The aim of this study, was to evaluate the quality of life (QoL) of the patients who suffered a possible ADR, using a specific questionnaire (HDRQoL, Baiardini et al. 2011), validated in Spanish.

**Methods**

We carried out a prospective multicenter study, with 19 participating allergy centers from Spain. The inclusion criteria were: patients who consulted on suspicion of an allergic reaction to a drug, age > 18 years old and have a signed informed consent. The HDRQoL was administered twice: before the allergic study began (Q0), and one month after it was completed (Q1). A questionnaire to measure the Psychological General Well-Being Index (PGWBI) was also applied before the allergic study. We described the main results of QoL of the patients. Logistic regression was used to describe associated variables to a lower QoL score. We also used linear regression to assess those variables associated with the change of QoL after the allergic study was performed.

**Results**

A total of 367 patients were recruited and 360 of them (240, 66.6% female; mean age 45.4 y, SD 15.6 y) correctly completed the questionnaire Q0. 346 patients completed the allergic study: in 150 patients (43.4%) an allergic reaction was confirmed (115/150 immediate; 35/150 delayed); in 196 patients (56.6%) an allergic reaction was ruled out after a challenge test with the suspicious drugs.

Analysing Q0 HDRQoL results, mean value was 32.14 (SD 11.84). The risk to have worse QoL was higher in patients that suffered an anaphylaxis (OR 2.37; 95% CI 1.17–4.81; p = 0.017), had a musculoskeletal chronic disease (OR 3.07; 95% CI 1.33–7.09; p = 0.008) or had had more than one ADR (OR 1.88; 95% CI 1.14–3.12; p = 0.014). We observed a mild correlation between the results of HDRQoL and PGWBI.

After completing allergological study, 335 patients answered the Q1 HDRQoL, showing a global improvement of QoL compared to Q0, with a mean value of 27.27 (SD 9.96). Patients with a true allergic reaction, musculoskeletal chronic disease or who had had more than one ADR had higher risk to keep a worse QoL after the diagnosis.

**Conclusion**

Having suffered an anaphylaxis, more than one allergic drug reaction or presenting a musculoskeletal disease are factors that worsen the quality of life of the patients. After the allergic study was completed, the QoL improved significantly in patients in whom allergic reaction was ruled out.

#### P141 Drug-induced anaphylactic reactions in children: a retrospective analysis of 159 validated spontaneous reports submitted to the adverse drug reaction database of the Federal Institute for Drugs and Medical Devices

##### Bernhardt Sachs^1^, Diana Dubrall^1^, Wilma Fischer-Barth^1^, Matthias Schmid^2^, Julia Stingl^1^

###### ^1^Federal Institute for Drugs and Medical Devices (BfArM), Bonn, Germany; ^2^Institute for Medical Biometry, Informatics and Epidemiology, (IMBIE), Bonn, Germany

**Correspondence:** Bernhardt Sachs - bernhardt.sachs@bfarm.de

*Clinical and Translational Allergy* 2018, **8(Suppl 3)**:P141

**Background**

Few data are available concerning drug-induced anaphylactic reactions in children. Beta-lactams and non-steroidal anti-inflammatory drugs are reported as frequent inducers based on a limited number of cases in published studies. The paucity of data concerning incriminated drugs, demographic data and associated factors prompted us to further investigate this issue on a larger scale by exploring the adverse drug reaction (ADR) database of the German Federal Institute for Drugs and Medical Devices. The overarching aim was to identify factors which are frequently associated with drug-induced anaphylaxis in children.

**Methods**

By means of an internationally standardized MedDRA query (SMQ) we identified 505 spontaneous reports of anaphylactic reactions in the ADR database which were registered between 01/2000 and 12/2016, originated from Germany, and referred to children (0–17 years). In order to focus on the most frequently reported drugs only drugs with more than three reports were considered for full analysis resulting in 242 reports. These cases were assessed with regard to the causal relationship with the suspected drug and the correctness of diagnosis according to standardized published criteria resulting in 159 validated cases. The validated cases were analysed concerning incriminated drugs, grade of severity, reported symptoms, demographic parameters, mode of application and associated factors like allergy/atopy, if reported. Since different drug classes may cause anaphylactic reactions by different pathomechanisms results were stratified by drug class.

**Results**

Among the 159 validated cases antibiotics (30.2%), analgesics/antipyretics (22.0%), and MRI-contrast media (11.9%) were most frequently incriminated. Intravenous application was noted for 39.6% of the cases. In 13.8% of the cases the drug had not been tolerated before (54/159 cases reporting previous use). Differences concerning the grade of severity, the reported symptoms and associated factors (allergy/atopy/gender/mode of application) were noted between different drug classes. Allergy/atopy was noted in 42.9% of analgesic/antipyretic cases (40/159 cases providing respective information).

**Conclusion**

No uniform pattern of drug-induced anaphylactic reactions in children was found. Observed differences between drug classes concerning allergy/atopy may reflect different pathomechanisms or result from method-immanent limitations. Intravenous application seems to confer a striking higher risk than oral application. As an easy measure to take a careful history could help to reduce the occurrence of drug-induced anaphylactic reactions in children.

#### P142 An assessment of allergy and adverse reaction history and documentation in patients on a Medical Emergency Admissions Unit (MEAU)

##### Daniel Law, Manjeet Singh, James Coulson, Laurence Gray

###### University Hospital of Llandough, Cardiff, United Kingdom

**Correspondence:** Daniel Law - daniel.law@doctors.org.uk

*Clinical and Translational Allergy* 2018, **8(Suppl 3)**:P142

**Background**

Approximately 15% of UK hospital in-patients are estimated to have their admission prolonged by an adverse drug reaction. This study aimed to assess if allergies and adverse drug reactions are correctly identified and documented in new patients admitted to University Hospital Llandough (UHL) in Cardiff.

**Methods**

Patients admitted to UHL via the MEAU were assessed for their drug allergy and adverse reaction status. To assess this, the patient’s allergy history was elicited by an investigator and this was compared to the documentation in the prescription chart. Medications administered during admission were compared to the allergy information obtained. The study took place over a five-day period from Monday 8th to Friday 12th January 2018. Patients who were unable to provide a verbal history of their allergies or who declined to participate were excluded.

**Results**

Thirty-nine patients were assessed during the duration of the audit. The median age was 75 (age range 25–94) and male–female ratio was 17:22. Allergies and adverse drug reactions were positively identified in 33% (13/39) of patients. 23% (9/39) of drug charts assessed had incomplete and/or incorrect documentation of allergies and adverse reactions. One patient was documented as having an allergy without information of the medication or reaction. This patient was administered medication regardless. 5% (2/39) of patients had allergies or adverse reactions identified by our investigator’s history which were not documented. 2% (1/39) of drug charts had an allergy section without a signature. One serious case was identified where a patient documented as having a trimethoprim allergy was administered co-trimoxazole and subsequently developed angioedema and had to be closely monitored.

**Conclusion**

This study looked at allergy and adverse reaction history taking, documentation and medication administration. The drug allergy history was not completely documented in 23% of cases. One patient had a history of an adverse reaction not elicited by the clerking doctor which could have led to patient harm. The administration of co-trimoxazole to a patient with trimethoprim allergy highlights the need for education of both medical and nursing staff. We will use these findings to develop an educational intervention in the MEAU to improve local practice in allergy and adverse reaction pharmacology, history taking and documentation.

#### P143 Allergic contact dermatitis to fentanyl patch with good tolerance to sistematic fentanyl

##### Blanca Noguerado-Mellado, Inés Torrado-Español, Vicente Albéndiz-Gutiérrez, Maria Carmen Lillo-Ordóñez, Patricia Rojas-Pérez-Ezquerra

###### Allergy Department, Madrid, Spain

**Correspondence:** Blanca Noguerado-Mellado - blancanoguerado@gmail.com

*Clinical and Translational Allergy* 2018, **8(Suppl 3)**:P143

**Background**

Fentanyl is a primarily opioid agonist. It is frequently used in general anesthetic and as a potent analgesic. It can be administered either oral, transdermal or systemically. Adverse effects due to opium alkaloids are usually because of a non-specifically histamine release. Only in a few cases, a true allergy mechanism could be involved. Immediate reactions to opioids are most frequent than delayed reactions. However, in the past years, delayed reactions have increased in frequency because of the wide use of transdermal therapeutic system (TTS) with several opioids for its potent analgesic properties.

**Case report**

A 52-year-old man with diagnosis of pancreatic cancer whom began treatment for a bone metastases pain with fentanyl TTS, at a dose of 50 micrograms per hour (mcg/h). After 10–15 days of treatment he developed an itchy papulovesicular rash in the application site of the fentanyl TTS. Afterwards, eczema and a superficial desquamation just in the application site of the patch were observed. He changed several times the site of application, but always developing the same symptoms in every single application. Later on, he tolerated other opioids such oral morphine or tramadol.

An allergy workout was performed. Patch tests (PT) in his forearm (due to the widely extension of the lesions in his back), with fentanyl at concentration of 10% in aqua (aq) and with buprenorphine 10% aq., in order to investigate probable cross-reactivity among other topic opioids. Readings were made at day 2 (D2) and day 4 (D4), with positive PT only with fentanyl at D2 (+++) and D4 (+++).

We decided to perform a single blind challenge test with buprenorphine 35 mcg/h in TTS, with a negative result. At this moment, fentanyl TTS was replaced by buprenorphine TTS, with good tolerance.

**Conclusion**

We present the case of allergic contact dermatitis (ACD) due to hypersensitivity to fentanyl with good tolerance to buprenorphine.

Positive PT in this patient suggests a type IV hypersensitivity mechanism.

Allergic reactions to opioids are frequently immediate, but delayed reactions could appear, especially when the drug is administered topically.

Cross reactivity among opioids of the same group could exist, so we think that is very important to perform an allergy workout in other to give alternative therapeutic drugs to our patients.

**Consent to publish**

Consent to publish was obtained from the patients involved in this study.

#### P144 Autoimmune progesterone dermatitis (clinical case)

##### Marta Lomikovska, Valentyna Chopyak, Halyna Potomkina

###### Lviv National Medical University, Lviv, Ukraine

**Correspondence:** Marta Lomikovska - ydmarta79@gmail.com

*Clinical and Translational Allergy* 2018, **8(Suppl 3)**:P144

**Background**

Autoimmune progesterone dermatitis is a rare disease with a hypersensitivity response to exogenous or endogenous progesterone, characterized by a recurrent rash on the skin and mucous membranes in the luteal phase of the menstrual cycle.

**Case report**

Complaints, medical background data from the patient card and results of laboratory examination, including immunological one.

A 33-year-old woman has been suffering from a disease for about 13 years. About 2 years before the first symptoms appeared she was using contraceptives. The first signs of the disease were observed in 2004, when against the background of the first pregnancy, allergic erythematous rashes throughout the body accompanied by angioedema suddenly appeared, spontaneous miscarriage occurred at the 6th week of pregnancy. In 2009, in the early stages of the second pregnancy, there were a pronounced allergic dermatitis, angioedema, pain and swelling of small joints, weight loss of 8 kg per week. At the 5th week, a recurrent spontaneous miscarriage occurred after which the symptoms of allergy stopped. In 2012, against the background of the third pregnancy, skin rashes and inflammation of the small joints of the hands and feet were observed. The prescription of progesterone to preserve pregnancy has led to a significant increase in muscle pain with a sharp muscle contractions and the appearance of asphyxia. At the 5th week of treatment there was a repeated spontaneous miscarriage, after which the symptoms disappeared. There are complaints of pain and swelling of the joints, erythematous skin rash, muscle pain, general fatigue, which began on the 10th day of the menstrual cycle and decreased from the 2nd day of menstruation. According to the results of the examinations: complete blood count within normal limits, total IgE 47kU/L. An intradermal test using progesterone was conducted, which turned out to be extremely positive. Desensitization with increasing dosage of progesterone in the form of intravaginal suppositories from 0.05 to 100 mg every 20 min on the 22nd day of the menstrual cycle has been performed. On the 25th day of the menstrual cycle 100 mg of progesterone was administered three times a day. After 3 days the complaints gradually disappeared. She has been feeling much better for 9 months after desensitization.

**Conclusion**

Desensitization expands the treatment options for women with autoimmune progesterone dermatitis and is perhaps the only option for treatment that retains the patient’s fertility.

**Consent to publish**

Consent to publish was obtained from the patients involved in this study.

#### P145 Allergy to nickel - paradoxes in dentistry

##### Lyudmila L. Lazarenko

###### North-Western State Medical University named after I.I. Mechnikov, Saint-Petersburg, Russia

**Correspondence:** Lyudmila L Lazarenko - lazarenko@list.ru

*Clinical and Translational Allergy* 2018, **8(Suppl 3)**:P145

**Background**

Introduction: Allergy to nickel is a frequent phenomenon not just in dentistry, (treatment with nickel salts in oncology, professional allergy in metallurgy, contact allergy of people who wear jewelry.) The fact is that nickel stimulates the T cells of the immune system, in a special way—it forms complex with two sections of the histocompatibility molecule, and two fragments of the T-cell receptor (Weltsien NU). Purpose: Study features of nickel allergy in patients with prosthetic.

**Methods**

124 patients of the St. Petersburg dental clinic aged 22–82 years old were examined. Of these, 78 women and 46 men. The patients underwent allergological testing in vivo- patch tests and in vitro-determination of IgE and IgG ELISA using the reagent D-.R FOOKE.

**Results**

in patients with the syndrome of “burning mouth” patch tests to nickel are positive in 60 people (in 48% of cases), IgE to nickel—in 43 people (35%), IgG—in 27 people (22%). Study features of nickel allergy in patients with prosthetic.

**Conclusion**

1. 71% of the people (88 people) are women (cooks and housewives and 29% (36 people) are male smokers and workers of the foundry.

2. In order to avoid the financial costs associated with re-prosthetics it is mandatory to pre-test allergology (patch tests and IgE and IgG antibodies in serum to nickel) in individuals at risk—cooks, confectioners, housewives, smokers, bijouterie lovers and workers in the metallurgical industry.

#### P146 Characteristics of drug hypersensitivity reaction reporting at a major tertiary hospital in Melbourne, Australia

##### Ar Kar Aung^1^, Hui Wen Tee^1^, Mei Jie Tang^1^, Nikki Rae Adler^2^, Sara Lee De Menezes^2^, Jason Anthony Trubiano^3^, Linda Velta Graudins^1^

###### ^1^Alfred Health, Melbourne, Australia; ^2^Monash University, Melbourne, Australia; ^3^Austin Health, Melbourne, Australia

**Correspondence:** Hui Wen Tee - wen.tee@gmail.com

*Clinical and Translational Allergy* 2018, **8(Suppl 3)**:P146

**Background**

We aimed to evaluate the characteristics of immediate and delayed drug hypersensitivity reaction reporting at a tertiary hospital in metropolitan Melbourne, Australia.

**Methods**

A retrospective study of all reports assessed by a centralised Adverse Drug Reaction (ADR) Review Committee, a multidisciplinary team including a senior pharmacist and specialist clinicians from dermatology, immunology, clinical pharmacology, infectious diseases and general medicine, over a 2-year period (2015–2016). Descriptive and univariate models were used to analyse outcomes, using standardised ADR definitions. Drug hypersensitivity reactions were classified according to previously published modified Gell and Coombs criteria, and time to reporting was used as a surrogate marker for the length of diagnostic and evaluation process.

**Results**

555 ADR episodes were captured over the study period. Twenty-nine percent were classified as severe or life-threatening reactions, with a total of 71.1% being of at least moderate severity. Immune-mediated hypersensitivity reactions were the most commonly reported ADRs (409, 73.7%), and of these, 38.6% were immediate (including anaphylaxis) and 61.4% were delayed reactions. Of the delayed hypersensitivity reactions (n = 251), 16.7% were severe cutaneous adverse reactions (SCAR), 53.4% non-SCAR and 21.5% single organ reactions. The median time to ADR reporting was 3 (1–10) days. The median time to ADR report submission was longer when multiple agents were implicated, [3 (1–10) vs. 5 (1–13) days for single vs. multiple implicated agents, p = 0.01] and for delayed hypersensitivity reactions [1 (0–3) vs. 6 (2–13) days for immediate vs. delayed, p < 0.0001]. The majority of adverse reactions (84.8%) were reported by pharmacists, with only 9.4% reported by medical staff. Pharmacists were noted to have a longer median time to ADR report submission when compared with medical staff [4 (1–11) days for pharmacists vs. 1 (0–6) day for medical staff, p = 0.01]. A total of 650 medications were implicated, with multiple agents in 165/555 (29.7%) episodes. Antimicrobials were by far the most commonly implicated agents in immunologically mediated ADRs (257/475, 54.1%).

**Conclusion**

Pharmacists are an integral part of ADR reporting and management. The majority of reported ADRs were delayed type hypersensitivity with a large proportion being mild non-SCAR reactions. All delayed hypersensitivity reactions were associated with a longer time to report, especially SCARs, suggesting that they required the most time for diagnosis and evaluation. Targeted education programs to promote the understanding of ADR principles, particularly for delayed immune-mediated reactions, is important for safer patient outcomes.

#### P147 A case of glatiramer acetate-induced eosiophilic pleuritis

##### Jan Walter Schroeder^1^, Olivia Leoni^2^, Alessandra Cernuschi^1^, Alessandra Protti^1^, Michel Chevalard^1^, Lea Caron^1^, Elide Anna Pastorello^1^

###### ^1^Niguarda Hospital, Milan, Italy; ^2^Regional Pharmacovigilance Centre of Lombardy, Milan, Italy

**Correspondence:** Jan Walter Schroeder - janwaltervolk.schroeder@ospedaleniguarda.it

*Clinical and Translational Allergy* 2018, **8(Suppl 3)**:P147

**Background**

Eosinophilia is sometimes related to many drugs. Often we see the first signs after some weeks of taking the drug. In our case the patient took the drug for 8 weeks. There were only some small description of system side effects of Glatiramer acetate, a very important drug for multiple sclerosis.

**Case report**

A 40-year-old Caucasian male presented to the Emergency Room (ER) of our hospital with progressive dyspnea and chest pain in November 2016. A bilateral exudative pleural effusion was confirmed on chest X-ray. The white blood cell count revealed 15,000 cells/mm^3^ and 34% eosinophils. His past medical history included quadriplegia resulting from a road accident, spondylosis and multiple sclerosis diagnosed in May 2016. Two months before ED admission treatment with Glatiramer Acetate (40 mg subcutaneously three times a week) had been commenced.

During hospital stay a thoracentesis of 800 ml fluid was undertaken and cytology revealed an excess of eosinophils (50% of leucocytes).

A diagnostic work-up of parasitic infection, other infections and autoimmunity was normal. Computed Tomography of thorax, abdomen and pelvis was negative.

A diagnosis of Glatiramer Acetate-induced eosinophilic pleuritis was made with subsequent withdrawal of the medication and starting treatment with high-dose methylprednisolone, leading to clinical improvement and peripheral eosinophilia normalization.

After discharge the patient continued prednisone therapy, but 2 months later steroids were stopped and he went back to the ER with dyspnea, right pleural effusion and eosinophilia. High-dose prednisone was re-established with subsequent clinical resolution.

**Conclusion**

In our knowledge, this is the second report of Glatiramer Acetate-induced hypereosinophilia and organ involvement. Hypereosinophilia is frequently related to many drugs and is often associated with a skin involvement like in DRESS (drug reaction with eosinophilia and systemic symptoms). Our case is related to Glatiramer as resolution occurred with discontinuation of the medication.

**Consent to publish**

I confirm that I have received written consent for online publication from all patients described (or their parents/guardians), where there are images, videos, or 3 more indirect identifiers to be published.

#### P148 Rapid drug desensitization with chemotherapeutics (platins, taxanes, etoposide, doxorubicin, and irinotecan): a single-center experience

##### Sevim Bavbek, Resat Kendirlinan, Reyhan Gümüsburun, Pamir Çerçi, Emre Özbek, Seda Altiner, Zeynep Çelebi Sözener, Sadan Soyyigit, Ömür Aydin

###### Ankara University School of Medicine, Division of Immunology and Allergy, Department of Chest Disease, Ankara, Turkey

**Correspondence:** Sevim Bavbek - bavbek@medicine.ankara.edu.tr

*Clinical and Translational Allergy* 2018, **8(Suppl 3)**:P148

**Background**

Rapid drug desensitization (RDD) induces a temporary tolerance to chemotherapeutics inducing hypersensitivity reactions (HSRs), but data are limited regarding its use outside of the United States of America. We aimed to report our experience with RDD to Chemotherapeutics; Platins, Taxanes, Etoposide, Doxorubicin, and Irinotecan.

**Methods**

The study was conducted as a retrospective chart review of patients with symptoms of HSRs to chemotherapeutics. RDD with chemotherapeutics was evaluated between 2012 and 2017. HSRs were classified as Grade I, II, and III based on their severity. Prick/intradermal tests were performed with culprit chemotherapeutics. The 12-step RDD protocol was used.

**Results**

The study consisted of 38 women and three men with a mean age of 53.3 ± 11.6 years old. A total of 122 RDD were performed. Patients had ovarian cancer (n:13, 31.8%), breast cancer (n:10, 24.4%), colon cancer (n:7, 17%), lung cancer (n: 4 9.8%), and the others [n: 7 (Endometrial sarcoma:1, Testicular cancer:1, Uterine cancer:1, Ampulla water tumor:1, Choledochal tumor:1, Peritonitis carcinomatosa:1, Merkel cell carcinoma:1)]. 22 patients experienced HSRs to Platins; 12 Carboplatin, 3 Cisplatin, 7 Oxaliplatin along with 31, 6 and 10 RDD to the implicated chemotherapeutics respectively. Fifty-two RDD were performed in 15 cases with Taxanes (Paclitaxel: n: 7, 23 RDD; Docetaxel: n: 8; 29 RDD). Twenty-three RDD were performed with the other chemotherapeutics (Doxorubicin n: 2, 11 RDD; Irinotecan n: 1, 10 RDD; Etoposide n: 1, 2 RDD). In 25 (61%) cases, no reactions occurred during RDD. Breakthrough reactions during RDD developed in 16 cases (39%, Platins: 11, Taxanes: 3, Doxsorubicin:1 Irinotecan: 1). Among 11 cases with HSR to Platins; two Grade I, three Grade II, and three Grade III rx developed to carboplatin, and one RDD procedure could not completed. Grade II rx developed in two cases with Oxaliplatin, one Grade III RDD procedure could not be completed. One Grade II rx was seen with Cisplatin. In Taxanes group; a total of three Grade II reactions were observed with one to Paclitaxel, and two Docetaxel. Four Grade II reactions were observed with Doxorubicin (n:1) and Irinotecan (n:1). Generally, Grade I reactions were seen at the early steps and Grade II, and III reactions were seen usually at the last steps.

**Conclusion**

In our hands, 98.3% of 122 RDD were completed. We found RDD is safe and effective in this largest series of RDD with chemotherapeutics in our country.

#### P149 ‘Prick’ testing - safe or not?

##### Matea Kolacevic^1^, Maja Ilijanic Samoscanec^2^, Robert Likic^3^, Iva Kraljickovic^3^, Iveta Mercep^3^

###### ^1^University Hospital Centre Sisters of Charity, Zagreb, Croatia; ^2^General Hospital Karlovac, Karlovac, Croatia; ^3^University Hospital Centre Zagreb, Zagreb, Croatia

**Correspondence:** Maja Ilijanic Samoscanec - maja.ilijanic.samoscanec@gmail.com

*Clinical and Translational Allergy* 2018, **8(Suppl 3)**:P149

**Background**

Skin testing is considered a safe diagnostic method for the diagnosis of IgE-mediated allergies, but systemic reactions have been reported. According to Bagg et al. (2009) and their prospective observational study, a systemic reaction occurred in 3.6% of patients of which only 0.4% underwent skin prick testing. Another study (Co Minh HB et al., 2006) confirmed that systemic reactions during skin testing are more common in patients with a history of anaphylaxis or anaphylactic shock.

Here, we present a case of a 73-year-old male who developed a generalised allergic reaction to ‘prick’ testing with β-lactams.

**Case report**

A 73-year-old man was hospitalised for drug hypersensitivity testing to antibiotics. His medical history revealed that he had Quincke’s oedema to amoxicillin–clavulanate and an anaphylactic reaction to the combination of cefuroxime and azitromycin. CAST ELISA testing for penicillin G, penicillin V, MDM, amoxicillin, cefalosporine and cefuroxime performed prior to hospitalisation was negative. After routine laboratory testing and physical examination, including vital parameters, the patient was exposed to ‘prick’ skin testing for penicillin G, amoxicillin–clavulanate, cefazoline and cefuroxime with positive and negative controls. Ten minutes later, the patient reported numbness of palms and soles and stomach pain, which was followed by signs of a systemic allergic reaction: face and neck flushing and peak expiratory flow (PEF) drop of 19%, with only the ‘prick’ test to cefuroxime markedly positive. The patient was given an antihistamine (*chloropyramine*) intravenously and was closely monitored for a 24-h period in which he did not experience any new hypersensitivity related signs or symptoms.

**Conclusion**

Although rare, a possibility of developing a systemic allergic reaction during ‘prick’ testing to β-lactams is something that should be considered before even starting skin testing. Special consideration should be given to patients with a history of severe allergic reactions, as they might have a higher risk for developing signs and symptoms of systemic hypersensitivity.

**Consent to publish**

I confirm that I have received written consent for online publication from all patients described (or their parents/guardians), where there are images, videos, or 3 more indirect identifiers to be published.

## Friday 20 April 2018

### Clinical cases - Poster Walk 16

#### P150 Generalized lichen nitidus following anti-programmed cell death 1 antibody treatment

##### Saeko Nakajima, Maria Cho, Atsushi Otsuka, Takashi Nomura, Kenji Kabashima

###### Kyoto University, Kyoto, Japan

**Correspondence:** Saeko Nakajima - saenakajimakuhp@gmail.com

*Clinical and Translational Allergy* 2018, **8(Suppl 3)**:P150

**Background**

Lichen nitidus (LN) is an uncommon skin disease characterized by minute flesh-colored papules on the abdomen, limbs, and genitalia. Generalized LN is a rare form of LN that is more often seen in children and young adults. Anti-programmed cell death 1 (PD-1) antibodies, such as nivolumab, are immune checkpoint inhibitors that prevent the binding of PD-1 to its ligands, thereby facilitating the activation of T-lymphocytes in patients with cancers such as melanoma and non-small cell lung carcinoma.

**Case report**

A man in his 40s was referred to our clinic after developing multiple skin lesions. He had been diagnosed with metastatic lung adenocarcinoma the previous year and received two courses of radiotherapy to his head and leg and four cycles of carboplatin, pemetrexed, and bevacizumab followed by nivolumab, which is one of the anti-PD1 antibody, administered every 2 weeks. After eight cycles of nivolumab over 5 months, he developed 1–2 mm shiny papules scattered on the upper limbs. Skin biopsy of the papular lesions showed typical histological features of LN. He subsequently developed pruritus, and a “very strong” class topical steroid was started on his right arm while nivolumab was continued. One month later, the skin lesions on his right arm completely resolved whereas those on his untreated arm and body remained.

**Conclusion**

We report a case of generalized LN that developed during anti-PD-1 therapy, and its atypical transformation into LP-like lesions and responsiveness to topical steroids. Such clinical progression in addition to the histopathological features distinguish the lesions seen in our case from other immune related adverse effects and further contributes to ongoing studies on the cutaneous reactions of nivolumab, suggesting a disease progression of LN that may occur in relation to immune checkpoint inhibitors.

**Consent to publish**

Consent to publish was obtained from the patient involved in this study.

#### P151 Suspected adverse reactions to vaccines in children referred to a general hospital

##### Sonja Posega Devetak^1^, Tina Vesel Tajnšek^2^, Nika Morgan^1^, Anja Koren Jeverica^2^, Nataša Toplak^2^, Štefan Blazina^2^, Gašper Markelj^2^, Tadej Avcin^2^

###### ^1^General hospital Izola, Izola, Slovenia; ^2^University Children’s Hospital Ljubljana, Ljubljana, Slovenia

**Correspondence:** Sonja Posega Devetak - sonja.posega@gmail.com

*Clinical and Translational Allergy* 2018, **8(Suppl 3)**:P151

**Background**

Diagnosis of suspected adverse reactions (SAR) to vaccines is important to prevent serious reactions at re-vaccination and to avoid unnecessary vaccine restriction. The aim of our study was to evaluate the management of children referred to a general hospital due to SAR following immunization.

**Methods**

We retrospectively analysed medical documentation of 117 children (56 girls, 61 boys, median age 2 years, IQR 1–6) who have been referred to the General hospital Izola due to SAR to vaccines from 2010 to 2017. Thirty five (30%) children were also sent for further diagnostic workup to the tertiary pediatric unit.

**Results**

Ninety-five percents of referrals were due to previous SAR associated to vaccine (64% to combined vaccine against diphtheria, tetanus, pertussis, poliomyelitis and *Haemophilus influenzae* type b (DTPPH), 17% to measles-mumps-rubella vaccine (MMR), 14% to others), 2% due to potential allergens contained in vaccines and 3% due to other reasons (hemophilia, neurological disorders). Among children with SAR associated to vaccine, 40 (36%) had local reactions, 36 (32%) systemic skin reactions (8 immediate, 28 delayed), 16 (14%) fever with irritability, 9 (8%) isolated neurological disorders and 11 (10%) other adverse reactions.

Hypersensitivity to DTPPH was confirmed with skin tests (ST) in four children—one immediate to whom the vaccine was later given in graded doses and three delayed (two of them later developed negative ST and were re-vaccinated without adverse reaction, the third got subcutaneous nodule once again). In one child with positive immediate ST to MMR, re-vaccination was withheld due to high protective antibodies. One child with positive delayed ST to hepatitis B vaccine is waiting for the opinion of infectious disease consultant at the Ministry of health and has not been re-vaccinated yet.

Forty-two (36%) children received booster dose with no adverse reactions, 3 (2%) have fulfilled the immunization programme and need no further doses. Forty-two (36%) haven’t been re-vaccinated yet because of adequate amount of protective antibodies, two due to medical contraindications (active neurological and rheumatologic disease), 24 (21%) haven’t completed diagnostic workup yet, 4 (3%) parents declined re-vaccinations.

**Conclusion**

Vaccination has been continued in 36% of children without adverse reactions and has been postponed in 36% because of adequate amount of protective antibodies. In 28% of children vaccination hasn’t been continued yet, mostly due to uncompleted diagnostic workup. Hypersensitivity to vaccines was confirmed in only 6 (5%) children, none of them had anaphylactic or serious delayed reaction.

#### P152 Contact allergy to topical ophthalmic medications

##### Liesbeth Gilissen^1^, Toon Hulshagen^2^, Lana De Decker^2^, An Goossens^1^

###### ^1^Department of Dermatology, University Hospitals KU Leuven, Leuven, Belgium; ^2^Faculty of Pharmaceutical Science KU Leuven, Leuven, Belgium

**Correspondence:** Liesbeth Gilissen - liesbeth.gilissen@uzleuven.be

*Clinical and Translational Allergy* 2018, **8(Suppl 3)**:P152

**Background**

Allergic contact dermatitis (ACD) from topical ophthalmic medications is often overlooked, and is most often characterized by eyelid eczema, although only conjunctivitis may also occur. To identify the most common allergenic culprits in them, results of patch tests, performed in a tertiary patch-test clinic between 1990 and 2016, were retrospectively analyzed.

**Methods**

Patch tests were performed with the pharmaceutical products used by the patients, and whenever possible, also the ingredients.

**Results**

117 out of 16 065 patch-tested patients (0.7%) had reacted positively to at least one ingredient of topical ophthalmic medications: 70 of them to an active principle, 34 to a vehicle component, and 14 to both. The most common allergenic active principles were antibiotics, i.e. tobramycin (n = 27), neomycin (n = 18) that often cross-reacts with the former, and polymyxin B (n = 12), and as vehicle components wool alcohols, an excipient (n = 19), and thiomersal (n = 12) and benzalkonium chloride, both preservatives (n = 10). The actual culprit could not be identified in 8 cases who only reacted positively to products used, particularly beta-blockers: 4 by patch testing, 3 only in a Repeated Open Application Test (or ROAT, application of a small amount of the product on the same spot of the patient’s forearm twice daily up to 2 weeks), while one patient experienced a flare-up of the symptoms following a provocative use test (reintroduction of the ophthalmic product).

**Conclusion**

In agreement with the literature, contact allergy to ophthalmic medications is most frequently caused by the active principles, particularly antibiotics. Most of these drugs are, however, not available as commercial patch-test preparations, which hampers their identification. With regard to the other ingredients, wool alcohols are a frequent cause of periocular dermatitis, but few reports focus on their use in eye medication. Moreover, given their wide use, preservatives cannot be regarded as common allergens, the more since the use of preservative-free single-dose eye drops is increasing. Last but not least, patch-test results may be false—negative, as in the case of β-blockers, such as timolol. The skin of the patch-test site, i.e. the upper back is less sensitive than eyelid skin, through which chemicals more easily penetrate. In those cases, ROATs or provocative use tests are helpful.

#### P153 Vemurafenib acts as an aryl hydrocarbon receptor antagonist

##### Heike C. Hawerkamp^1^, Andreas Kislat^1^, Peter Arne Gerber^1^, Marius Pollet^2^, Anatoly A. Soshilov^3^, Michael S. Denison^3^, Afaque A. Momin^4^, Stefan T. Arold^4^, Angeliki Datsi^1^, Stephan A. Braun^1^, Mario E. Lacouture^5^, Thomas Haarmann-Stemmann^2^, Bernhard Homey^1^, Stephan Meller^1^

###### ^1^Department of Dermatology, Medical Faculty, Heinrich-Heine-University, Düsseldorf, Germany; ^2^Leibniz-Research Institute for Environmental Medicine, Düsseldorf, Germany; ^3^Department of Environmental Toxicology, University of California, Davis, CA, United States; ^4^King Abdullah University of Science and Technology (KAUST), Computational Bioscience Research Center (CBRC), Division of Biological and Environmental Sciences and Engineering (BESE), Thuwal, Saudi Arabia; ^5^Dermatology Service, Department of Medicine, Memorial Sloan-Kettering Cancer Center, New York, NY, United States

**Correspondence:** Heike C. Hawerkamp - heike.hawerkamp@hhu.de

*Clinical and Translational Allergy* 2018, **8(Suppl 3)**:P153

**Background**

In recent years, the BRAF-inhibitor vemurafenib has been successfully established in the therapy of advanced melanoma. Despite its superior efficacy, the use of vemurafenib is limited by frequent inflammatory cutaneous adverse events that affect patients’ quality of life and may lead to dose reduction or even cessation of anti-tumor therapy. To date, the molecular and cellular mechanisms of vemurafenib-induced rashes have remained largely elusive.

**Methods**

We treated skin explants, keratinocytes and T cells with vemurafenib and analyzed the cellular response via RT-qPCR, flow cytometry and lymphocyte activation tests. Additionally we deployed immunohistochemistry, different cell-free protein-interaction assays and computer simulation.

**Results**

We here demonstrate that vemurafenib inhibits the downstream signaling of the canonical pathway of aryl hydrocarbon receptor (AhR) in vitro, thereby inducing the expression of proinflammatory cytokines (e.g. *IL1B*, *TNF*) and chemokines (e.g. *CCL5*). In line with these results we observed an impaired expression of AhR regulated genes (e.g. *CYP1A1*) and an upregulation of the corresponding proinflammatory genes in vivo. Moreover, results of lymphocyte activation tests showed the absence of drug-specific T cells in respective patients.

**Conclusion**

Taken together, we obtained no hint of an underlying sensitization against vemurafenib but found evidence suggesting that vemurafenib enhances proinflammatory responses by inhibition of AhR signaling. Our findings contribute to our understanding of the central role of the AhR in skin inflammation and may point towards a potential role for topical AhR agonists in supportive cancer care.

#### P154 “Treating through’’ - clopidogrel hypersensitivity treatment

##### Armine Hakobyan, Mariam Movsisyan, Tatev Aloyan, Alexandra Zakaryan, Spartak Gambarov

###### Yerevan State Medical University, Yerevan, Armenia

**Correspondence:** Mariam Movsisyan - mariamrmovsisyan@gmail.com

*Clinical and Translational Allergy* 2018, **8(Suppl 3)**:P154

**Background**

Clopidogrel is the most frequently prescribed antiplatelet medication. Clopidogrel hypersensitivity occurs in 4–6% of patients, who receive this drug. There are several types of hypersensitivity reactions to clopidogrel—erythematous, macular or morbilliform skin rash with or without pruritus, urticaria and angioedema [1–4]. In 1.5% of patients symptoms are severe enough to result in drug discontinuation. But discontinuation of clopidogrel is extremely undesirable after percutaneous coronary intervention because of the risk of stent thrombosis and death. “Treating through’’ therapy is suggested for treatment of clopidogrel hypersensitivity and is more preferable than a desensitization procedure or changing to a different antiplatelet agent.

**Case report**

A 46-year-old patient was admitted to the Allergology outpatient clinic on 03/02/2017 with complaints of painful swelling and itching of both hands and arthralgia of wrists. The rest of the skin was without changes. Lab results were normal. The patient had undergone cardiac catheterization with PCI (percutaneous coronary intervention) of DESmRCA and DESdRCA on 20/01/2017. The patient was taking the following medications: Clopidogrel 75 mg, atorvastatin 40 mg, aspirin 100 mg, amlodipine 5 mg, ramipril 2.5 mg. Among all mentioned medications only clopidogrel and aspirin were newly prescribed for the patient.

Prednisone 30 mg twice a day (with tapering of the dose over a week) was prescribed along with diphenhydramine 25–50 mg to be taken every 6 h for 1 week. After Prednisone was discontinued, symptoms returned and the Prednisone prescription was renewed for 30 days, which brought to the complete resolution of the symptoms.

**Conclusion**

Hypersensitivity reactions to clopidogrel are not well understood and there is no optimal treatment regimen in management. In this report we demonstrate that patients with clopidogrel hypersensitivity can continue the treatment without drug interruption. Our experience with clopidogrel provides proof of concept that development of drug hypersensitivity does not always necessitate the discontinuation of the medication.

The relative ease and effectiveness of this approach could allow for potential application to the management of hypersensitivity reactions to various, other vital medications when there is a lack of alternative treatment methods.

**Consent to publish**

Consent to publish was obtained from the patient involved in this study.

#### P155 Anaphylaxis to verapamil: an unexpected culprit

##### Bárbara Kong Cardoso, Sofia Martins Farinha, Elza Tomaz, Filipe Inácio, Sara Correia

###### Centro Hospitalar de Setúbal, Setúbal, Portugal

**Correspondence:** Bárbara Kong Cardoso - barbarakc@gmail.com

*Clinical and Translational Allergy* 2018, **8(Suppl 3)**:P155

**Background**

Invasive procedures (diagnostic/therapeutic) have been increasing in frequency and sometimes associated to allergic reactions. The improvement of the pharmacological protocols resulted in the increase of possible implicated drugs. As procedures may have to be repeated identifying the culprit drug is crucial for avoiding further allergic accidents.

Hypertension is one of the most common worldwide diseases. Verapamil, a calcium channel blocker, is used to treat hypertension, angina and certain heart rhythm disorders. Verapamil reactions have been reported previously, but a very limited number of cases have been reported.

On the other hand iodinated contrasts are often responsible for anaphylactic reactions, making them main suspects whenever they are involved.

Diagnostic efficacy of tests used in drug allergy depends on the focused drug and procedures also demand standardization for each drug. A lot of investigation is lacking in this field.

**Case report**

A sixty-nine years old male patient was referred to our clinic by his cardiologist due to an anaphylactic reaction during a coronary angiography performed 2 months earlier stating the possible need of repeating the procedure. Since the exam was performed with iopromide, the reaction was attributed to this contrast agent. The other drugs administered during the procedure were: heparin, lidocaine, verapamil, isosorbide mononitrate, clopidogrel and acetylsalicylic acid.

The patient current medications were: acetylsalicylic acid, isosorbide mononitrate, tamsulosin and bisoprolol. The patient also reported a positive history of allergy to penicillin (sudden generalized erythema and face angioedema after a penicillin injection) and mentioned no reactions with any anti-inflammatory agents.

Allergic study workup to penicillin, contrast agents (iopramide and iopamidol), verapamil, heparin, lidocaine and latex were performed. The penicillin study revealed a positive intradermal test to the minor determinant mixture 1/100 and a positive IgE to Penicilloyl V and G. The skin test to verapamil 1/100 was positive (and negative in five patients using verapamil without reaction) as well the BAT (1 mcg/ml) with a 5.8% activation and a 6.1 stimulation index). The results of the other drugs studied were negative. The final diagnosis was allergy to penicillin and to verapamil.

**Conclusion**

This multiple drug allergy case shows us that although more commonly described, the contrast agent was not culprit drug for this case of anaphylaxis. In spite of the very few reports, Verapamil was the trigger. The skin intradermal test and the Basophil Activation Test (BAT) proved useful in the diagnosis.

**Consent to publish**

Consent to publish was obtained from the patient involved in this study.

#### P156 Is fondaparinux always an alternative in allergy to low molecular weight Heparins?

##### Cosmin Boteanu^1^, Javier Arsenio Dionicio Elera^1^, Rosario Gonzalez-Mendiola^1^, Maria Aranzazu Jimenez Blanco^1^, Gian Marco Chiarella Privette^2^, Agne Ramonaite^3^, Antonio Alvarez Perez^2^, Jose Julio Laguna Martinez^1^

###### ^1^Hospital Central de la Cruz Roja, Allergy Unit, Faculty of Medicine, Alfonso X el Sabio University, Madrid, Spain; ^2^Hospital Central de la Cruz Roja, Allergy Unit, Madrid, Spain; ^3^Republic Klaipeda Hospital, Klaipeda, Lithuania

**Correspondence:** Cosmin Boteanu - cosbote@yahoo.com

**Background**

Heparins are potent anticoagulants that have been widely prescribed in clinical practice.

Hypersensitivity reactions to heparins are not very frequent. Delayed skin lesions after subcutaneous heparin are the most common type of hypersensitivity reactions. Some studies confirm that up to 7.5% of the patients receiving heparins develop lesions at the injection site. Management of hypersensitivity reactions includes finding an alternative. Fondaparinux and novel oral anticoagulants may be safe alternatives.

We present two cases of patients that presented itching, erythema around the site of the administration after receiving prophylaxis of the thromboembolic disease with enoxaparin: patient 1 (61 years old woman) who refers two episodes, 48 h and respectively 24 h after starting the prophylaxis, and patient 2 (47 years old woman) who refers the symptoms 1 month after starting the drug.

**Case report**

We performed a skin allergy test (skin prick and intradermal tests) with heparin sodium, dalteparin, nadroparin, bemiparin, enoxaparin, fondaparinux. Readings were taken after 20 min, 24 h, 48 h, 96 h and 1 week. After obtaining the patient´s informed consent, subcutaneous challenge test was performed, depending of the results.

Patient 1: The results of the skin allergy test were negative at 20 min, 24 h, 48 h. At 96 h and 1 week readings intradermal test for dalteparin, nadroparin, bemiparin and enoxaparin were positive. At 1 week there was also positive reading of the intradermal test for heparin sodium.

The subcutaneous challenge test with fondaparinux was positive: 6 h after the first doses the patient refers itching, erythematous plaque around the site of administration.

Patient 2: The results of the skin allergy test were negative at 20 min, 24 h. At 48 h, 96 h and one week readings intradermal test for dalteparin, nadroparin, bemiparin and enoxaparin were positive.

The subcutaneous challenge test with fondaparinux was negative.

**Conclusion**

We present two cases of cutaneous delayed-type hypersensitivity reactions to enoxaparin, with cross reactivity with low molecular weight heparins.

One of the patients was sensitized to Fondaparinux too, although it is usually a safe alternative in patients sensitized to heparins. The other patient tolerated fondaparinux.

Different low molecular weight heparins, heparin sodium and fondaparinux should be included in the tests because of the high rate of cross-reactivity between heparins, low molecular weight heparins and in some cases fondaparinux too.

**Consent to publish**

Consent to publish was obtained from the patients involved in this study.

#### P159 When to administer antibiotic prophylaxis in a surgery – a case report

##### Cosmin Boteanu^1^, Joaquin Archilla Esteban^2^, Javier Arsenio Dionicio Elera^1^, Rosario Gonzalez-Mendiola^1^, Maria Aranzazu Jimenez Blanco^1^, Gian Marco Chiarella Privette^3^, María Del Mar Medina Santos^3^, Jose Julio Laguna Martinez^1^

###### ^1^Hospital Central de la Cruz Roja, Allergy Unit, Faculty of Medicine, Alfonso X el Sabio University, Madrid, Spain; ^2^Hospital Central de la Cruz Roja, Anesthesiology Unit, Madrid, Spain; ^3^Hospital Central de la Cruz Roja, Allergy Unit, Madrid, Spain

**Correspondence:** Cosmin Boteanu - cosbote@yahoo.com

*Clinical and Translational Allergy* 2018, **8(Suppl 3)**:P159

**Background**

During the perioperative period patients are exposed to numerous agents, mostly drugs (anesthetic drugs, antibiotics), latex, antiseptics, etc., being all of them able to induce adverse reactions.

The diagnosis of perioperative hypersensitivity reactions is complex, and it is a combined effort between allergists and anesthesiologists in order to identify the cause of the reaction.

We present the case of a 47 years old woman that presented facial itching, hives in the thorax region in the perioperative area. The surgery was canceled.

**Case report**

Detailed clinical history with the anesthesiologist shows that amoxicillin-clavulanic acid was administered as prophylaxis before the surgery, 30 min before the scheduled time to perform the anesthesia procedure and 15 min after the administration the patient developed the reaction.

We performed skin tests (skin prick and intradermal test) with PPL, MDM, penicillin G, amoxicillin, clavulanic acid, latex, chlorhexidine. Specific IgE to penicillin G, penicillin V, amoxicillin, ampicillin, latex, chlorhexidine and tryptase were determined. After obtaining the patient´s informed consent, single blind challenge test with penicillin, amoxicillin-clavulanic acid, and handling test with latex gloves were performed.

The skin tests (skin prick and intradermal test) with PPL, MDM, penicillin G, amoxicillin, clavulanic acid, and chlorhexidine were negative. Skin prick test with latex was negative. The specific IgE to penicillin G, penicillin V, amoxicillin, ampicillin, latex and chlorhexidine were negative (< 0.35 ku/L). The level of tryptase was 3.55 mcg/L. The handling test with latex gloves was negative.

The single blind challenge test to penicillin was negative. The single blind challenge test to amoxicillin-clavulanic acid was positive: 1 h after the last doses administered the patient presented itching, hives in the thorax region.

**Conclusion**

The antibiotics are one of the most common causes of perioperative reactions. They are usually administered during the induction phase of anesthesia. An increase in the period of time between the administration of the antibiotic and the anesthetic induction should be recommended, could identify the reaction in an early moment before administration of other drugs and this will simplify the allergologic study of a perioperative hypersensitivity reactions. Latex and chlorhexidine should be tested as routine in perioperatory reactions because they are common antigens in surgical area.

**Consent to publish**

Consent to publish was obtained from the patients involved in this study.

## Friday 20 April 2018

### Clinical cases - Poster Walk 17

#### P160 Performance of a specialized day-hospital regarding drug hypersensitivity

##### Sofia Campina, João Gaspar Marques

###### Centro Hospitalar Lisboa Ocidental, Unidade de Imunoalergologia, Lisboa, Portugal

**Correspondence:** Sofia Campina - sofiacampinac@gmail.com

*Clinical and Translational Allergy* 2018, **8(Suppl 3)**:P160

**Background**

The investigation of drug hypersensitivity (DH) is a requisite in a Immunoallergy Department and represents an important characteristic for a tertiary referral hospital. In our Immunoallergy Unit this investigations takes place in a specialized Day-Hospital where we perform drug skin and provocation tests.

**Methods**

Retrospective analysis of the clinical records of Day-Hospital sessions performed in order to investigate DH, between Nov 2010 and May 2017.

**Results**

301 sessions were performed (± 3 sessions/week), 114 drug skin tests (ST), (prick, intradermal and epicutaneous) and 187 drug provocation tests (PT). ST were performed: 66.67% to antibiotics (mainly betalactams, 63.16%), 8.77% to metamizol, 7.89% to general anaesthetics, 3.51% to corticosteroids and 13.16% to other drugs. Seventeen ST (14.91%) were considered positive, 9 to betalactams, 1 to quinolone, 4 to metamizol, 1 to general anaesthetic and 2 to other drugs. Regarding the 187 PT, 43.85% were to NSAID (54.88% to an alternative NSAID like paracetamol, nimesulide or selective COX-2 inhibitor; and 45.12% with AAS or the culprit drug), 41.18% to antibiotics (80.52% to betalactams, 12.99% to macrolides, 6.49% to quinolones), and 14.97% to other drugs. We had 9 positive PT (4.81%), all of them with mild mucocutaneous symptoms: 5 to NSAID, 2 to betalactams and 2 to other drugs.

**Conclusion**

In a 6 year period, from three hundred Day-Hospital sessions only concerning the investigation of DH, we had 8.63% (n = 26) positive results confirming the diagnosis of drug allergy. Betalactams and NSAID represent the majority of the culprit drugs, but our diagnosis of NSAID hypersensitivity could be undervalued because PT with an alternative NSAID was more usually perfumed than with AAS. On the other hand is important to notice that 65.38% of drug allergy was confirmed by ST and 34.61% by PT, which means that a well designed investigation of DH requires not only an expert team but also the continuous development of an immunoallergy specialized Day-Hospital.

#### P163 Systemic reaction to oral immunotherapy against house dust mite

##### Peter Valentin Tomazic

###### Medical University of Graz, Graz, Austria

**Correspondence:** Peter Valentin Tomazic - peter.tomazic@medunigraz.at

*Clinical and Translational Allergy* 2018, **8(Suppl 3)**:P163

**Background**

Oral immunotherapy is a promising alternative to subcutaneous therapeutic strategies. A relatively new development is oral immunotherapy against house dust mites. Here we report our first experience with a potential systemic and local adverse reaction to that therapy.

**Case report**

A 20 years old female patient was referred to our out patient clinic for allergic rhinitis. In skin prick test and blood test (ImmunoCAP) sensitization to grass, trees and house dust mite (HDM) was diagnosed. Since HDM showed highest specific IgE levels and symptom calender correlated primarily to HDM related problems an oral immunotherapy (IT) against HDM was administered.

However after 3 months of immunotherapy the patient complained about bowel pain, dysphagia, nausea and swelling of the tongue as well as burning of the oral mucosa. Due to the timely correlation to IT administration an adverse reaction to the IT was suggested and the therapy was stopped and replaced by symptomatic therapy with oral antihistamines and topical steroids. During the follow-up no further systemic (or local) reactions as described above occured.

**Conclusion**

Adverse reaction to oral IT against HDM are potentially possible. If symptomatic therapy in this case does not achieve sufficient relief subcutaneous therapy will be tried.

**Consent to publish**

I confirm that I have received written consent for online publication from all patients described (or their parents/guardians), where there are images, videos, or 3 more indirect identifiers to be published.

#### P164 From acute sinusitis to a hypertensive reaction: a case report

##### Maja Ilijanic Samoscanec^1^, Matea Kolacevic^2^, Annemarie Balasko^3^, Radovan Vrhovac^3^, Robert Likic^3^

###### ^1^General Hospital Karlovac, Karlovac, Croati; ^2^University Hospital Centre Sisters of Charity, Zagreb, Croatia; ^3^University Hospital Centre Zagreb, Zagreb, Croatia

**Correspondence:** Maja Ilijanic Samoscanec - maja.ilijanic.samoscanec@gmail.com

*Clinical and Translational Allergy* 2018, **8(Suppl 3)**:P164

**Background**

Ephedrine and pseudoephedrine are sympathomimetic drugs often used as decongestants. Both drugs can increase blood pressure (BP), however, according to loratadine + pseudoephedrine SmPC, this side effect is very rare. Furthermore, it is well known that ephedrine has a more powerful effect on BP then pseudoephedrine (Salerno et al., 2005; Drew et al., 1978).

Here, we present a case of a 50-year-old male who developed hypertensive reaction while using oral pseudoephedrine and nasal ephedrine.

**Case report**

A 50-year-old man was set up with 24-h ambulatory BP monitor due to borderline BP values (140/90 mmHg) registered recently on a regular check up. In his chronic therapy, he was taking rosuvastatin 10 mg and for an acute sinusitis and nasal congestion at the time, he also started on amoxicillin with clavulanic acid, oral loratadine + pseudoephedrine 5/120 mg 2 times daily and nasal ephedrine 4–5 times daily. On the 3rd day of treatment, he woke up with tinnitus. Before noon, his BP was 150/125 mmHg, so he took perindopril + amlodipine 10/10 mg. Shortly after, his BP was 170/120 mmHg, so he took bisoprolol 5 mg and alprazolam 0.5 mg. In the evening, he developed headache, so he took bisoprolol 5 mg, diclofenac 50 mg, oksazepam 10 mg and went to the Emergency department (ED) where the BP was 190/120 mmHg. He was monitored there overnight, did not receive any more medicines and his BP decreased to 135/90 mmHg in the morning. He was discharged from the ED with perindopril + amlodipine 5/5 mg once daily.

**Conclusion**

In our view, sympathomimetic decongestants should be carefully prescribed and taken in patients with prehypertensive BP levels. We propose using pseudoephedrine alone rather than ephedrine in patients with prehypertensive BP values, as according to a meta-analysis by Salerno et al. (2005), pseudoephedrine causes a small increase in systolic BP (0.99 mm Hg) with no effect on diastolic BP.

**Consent to publish**

I confirm that I have received written consent for online publication from all patients described (or their parents/guardians), where there are images, videos, or 3 more indirect identifiers to be published.

#### P165 Treatment of nasal polyps with low dose aspirin for asipirin sensitive patients

##### Agne Savonyte^1^, Justas Arasimavicius^1^, Darius Rauba^2^, Violeta Kvedariene^3^

###### ^1^Faculty of Medicine, Vilnius university, Vilnius, Lithuania; ^2^Clinic of Ear, Nose, Throat and Eye Diseases, Faculty of Medicine, Vilnius University, Vilnius, Lithuania. Center of Ear, Nose and Throat Diseases, Vilnius University Hospital Santariskiu Clinics, Vilnius, Lithuania; ^3^Clinic of Infectious, Chest diseases, Dermatology and Allergology, Institute of Clinical Medicine, Vilnius, Lithuania and Institute of Biomedical Sciences, Department of Pathology, Faculty of Medicine, Vilnius University, Vilnius, Lithuania

**Correspondence:** Agne Savonyte - agne.sav.95@gmail.com

*Clinical and Translational Allergy* 2018, **8(Suppl 3)**:P165

**Background**

Aspirin-exacerbated respiratory disease (AERD) represents a severe form of chronic rhinosinusitis (CRS) characterized by nasal polyposis, bronchial asthma, and aspirin intolerance. This syndrome is difficult to manage, due to several recurrences that require multiple revision endoscopic sinus surgery. One of possibility is treatment with acetylsalicylic acid (AsA) for aspirin sensitive patients. The aim: To present a few clinical cases that prove that desensitization is one of the effective treatment methods for patients who have nasal polyps and aspirin-induced asthma.

**Case report**

We present two patients 54 years old man and 56 years old woman with hypersensitivity to AsA and bronchial asthma with nasal polyps. Several years of disease and many revision polypectomies were made. Nasal eosinophilia in the nasal secretion were observed. One of patient was allergic with positive skin prick tests, another—non allergic. Both patients had anaphylaxis with bronchospasm induced by AsA in the clinical history. Hypersensitivity to AsA was proved by oral provocation test. Oral provocation tests were positive with 880 mg for woman and 100 mg for man. Desensitization with aspirin was made and 300 mg acetylsalicylic acid continued permanently once a day. Influence of treatment with aspirin was positive, nasal eosinophilia was reduced. Nasal polyps disappeared and smell recovered for man. Consultation of allergist and ENT was repeated after two years. Nasal polyps do not recurred and weren‘t operated. Now he has a sense of smell. The woman ended treatment by aspirin after 2 weeks because of gastralgia.

**Conclusion**

Desensitization for aspirin is one of the alternative methods of treatment for nasal polyps and aspirin-induced asthma to people who have hypersensitivity to aspirin.

**Consent to publish**

Consent to publish was obtained from the patient involved in this study.

#### P167 Case of allergy to nickel against the background of its reception in food

##### Marina Peredelskaya

###### RMAPE, Moscow, Russia

**Correspondence:** Marina Peredelskaya - concy1984@gmail.com

*Clinical and Translational Allergy* 2018, **8(Suppl 3)**:P167

**Background**

Nickel is one of the most commonly used metals; it is used for the manufacture of jewelry, plates and dishes, and medical products.

**Case report**

A patient N, 32 years old, female, complains of pruritic rash on the body skin with the itch intensity up to 8–9 points and the number of lesions more than 50.

Allergic background: for quite some time now the patient noted occasional eruptions on her skin after a contact with jewelry made of non-precious metals. Previously patch skin tests with nickel showed a positive reaction.

The patient sought emergency medical care with complaints of a number of itchy lesions erupted on her whole body during the last 24 h.

On admittance: state of moderate severity, the patient was emotionally labile, focused on her body sensations, tearful. On the skin of face, upper and lower extremities and torso a punctuate purpura with lesions up to 0.5 cm diameter, prone to confluent. A physical status was within normal limits.

In order to control the itching, as well as to sedate the patient, antihistamines of the first generation were administrated parenterally; but the eruptions kept to progress and to intensify; lesions were spread throughout the whole body, merged in gigantic areas. System glucocorticosteroids therapy was administrated, with 120 mg of prednisolone, but then new lesions kept appearing in a large number, including after-meal rash. Water, tea, bakery products, thin yoghurts did not impact the skin condition, whereas the intake of pasta, cereals, and similar products provoked intensifying of eruptions. The patient observation revealed a sharp increase in the rash after such manipulations as intravenous injections or blood sampling from the vein, the process spreading from the injection site to the entire arm. A detailed anamnesis of the disease: on the eve of the start of hives, the patient purchased a coffee machine (with metal nickel-plated parts) and started to use it. Diagnosis: A systemic contact dermatitis. An allergy to nickel.

The injection treatment was discontinued and a therapy with per oral GCS and antihistamines of the second generation was administrated. A recommendation was given to cook and to eat food using ceramic or wooden utensils. Three days later marked positive dynamics of the skin process has been noted.

**Conclusion**

The episode of systemic contact dermatitis has developed due to exposure to nickel from ingestion in food, as well as during the parenteral treatment.

**Consent to publish**

Consent to publish was obtained from the patient involved in this study.

#### P168 The role of serum and local IgE concentration in quality of life of patients with allergic rhinosinusitis

##### Atanas Vlaykov, Dimitar Mihaylow, Valentin Stoyanov

###### Medical University, Stara Zagora, Bulgaria

**Correspondence:** Atanas Vlaykov - at.vlaykov@gmail.com

*Clinical and Translational Allergy* 2018, **8(Suppl 3)**:P168

**Background**

Allergic rhinosinusitis (AR) is a symptomatic disorder of the nose as a result of IgE-mediated inflammation and provoked by exposure of nasal mucosa to allergens.

The task we set was to determine the concentration of IgE molecules in blood serum and nasal lavage in patients with intermittent and persistent AR and to compare it with quality of life questionnaire results of their subjective feeling and potential health damage.

**Methods**

Sino-Nasal Outcome Test-22 (SNOT-22) is one of the most frequently used quality of life instruments for sinonasal diseases, including 22 symptoms, with possibility of self-assessment in 5 point scale.

Measurement of concentration of IgE-total was performed with IgE-Sandwich ELISA-kits (Nova Tec Immunodiagnostica Gmbh). Statistical data processing was carried out using IBM SPSS Statistics v.21.0/2012.

**Results**

It was performed comparison between serum and nasal lavage IgE concentration in 50 patients—40 cases and 10 healthy controls (21 males and 29 females) with mean age of 40.86 ± 17.37 (ranging from 19 to 79) years. Through statistical and comparative analysis of the results of SNOT-22 in patients with intermittent and persistent allergic rhinosinusitis, the authors make inferences and conclusions about the extent of quality of life impairment among patients with the two main forms of hay fever.

**Conclusion**

Allergic rhinitis is characterized by a constellation of symptoms of the nose, eyes, ears, mouth and palate. A special place among them take itching, sneezing, nasal congestion, rhinorrhea, cough and post nasal drip into the pharyngeal cavity. It is believed that IgE antibodies have a key role in atopic response and the clinical manifestations of these symptoms.

In our study we did not find statistically significant correlation between IgE antibodies concentration in serum or nasal lavage in patients with intermittent and persistent allergic rhinitis and SNOT-22 results in both groups.

Because IgE are unique to each allergen, checking for specific variants in blood or nasal secretion could be more sensitive than total IgE antibodies.

#### P169 Dual sensitization of latex and chlorhexidine causing severe anaphylaxis after pelvic examination

##### Mongkhon Sompornrattanaphan^1^, Yuttana Srinoulprasert^2^, Duangjit Kanistanon^2^, Torpong Thongngarm^1^

###### ^1^Division of Allergy and Clinical Immunology, Department of Medicine, Siriraj Hospital, Mahidol University, Bangkok, Thailand; ^2^Department of Immunology, Siriraj Hospital, Mahidol University, Bangkok, Thailand

**Correspondence:** Mongkhon Sompornrattanaphan - mongkhon.som@mahidol.ac.th

*Clinical and Translational Allergy* 2018, **8(Suppl 3)**:P169

**Background**

Natural rubber latex (NRL) and chlorhexidine (CHX) are not uncommon culprit causing anaphylaxis during perioperative period. Mucosal contact in highly sensitized individuals can also lead to severe allergic reaction. Herein, we reported a case of severe anaphylaxis after pelvic examination with dual sensitization to NRL and CHX.

**Case report**

A 54-year-old woman presented with syncope shortly after the pelvic examination. She was previously diagnosed with spinal cord tumor which was surgically removed 4 years ago led to spastic paraplegia and bed-bounded status. She underwent pelvic examination because of vaginal spotting. CHX solution was used to prep the vulvar area, and the gynecologist used NRL gloves and lubricating jelly for the pelvic examination. No pre-procedural medication was administered. The patient complained of perivaginal pruritus shortly after the end of examination. Thirty minutes later, she developed generalized urticaria, swollen eyelids and hypotensive syncope (Fig. 1). After the treatment of severe anaphylaxis. All symptoms improved without biphasic reaction.

**Fig. 1:**
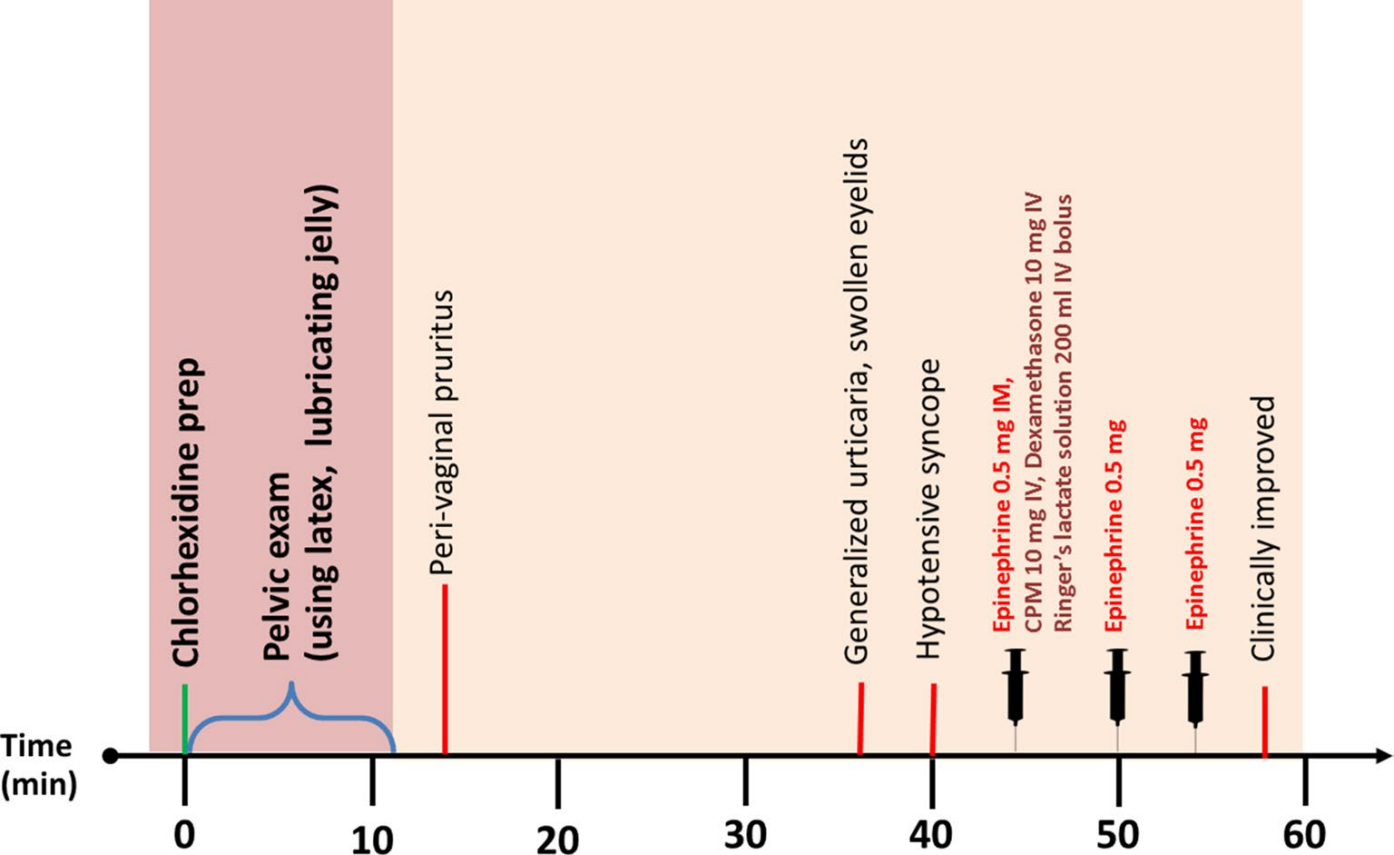
Case report

Investigations for the culprits were evaluated 6 weeks after the episode. A skin prick test to 0.002 mg/ml CHX solution demonstrated wheal formation (7 × 6 mm) with surrounding flare (29 × 25 mm). Latex-specific IgE was elevated at 45.9 kUA/L. Basophil activation test (BAT) for CHX was performed and positive results were obtained. She also had a history of fruits allergy including banana, kiwi, longans and jackfruits. Thus, we performed skin prick to prick testing which showed positive results for all aforementioned fruits. Written informed consent was obtained from the patient for publication of this case report and accompanying images.

**Conclusion**

Concomitant mucosal exposure of NRL and CHX in sensitized patients during pelvic examination can lead to severe anaphylaxis. Screening for drug and NRL allergy should be expanded to non-operative procedures that involve with mucosal contact. Preventive approaches in high risk individuals may improve patient safety.

**Consent to publish**

I confirm that I have received written consent for online publication from all patients described (or their parents/guardians), where there are images, videos, or 3 more indirect identifiers to be published.

## Friday 20 April 2018

### Clinical cases - Poster Walk 18

#### P170 Prospective study of the allergological exploration of hypersensitivity reactions to iodinated contrast media

##### Nathalie Masson^1^, Amandine Vial-Dupuy^2^, Jean-Eric Autegarden^1^, Hafida Gaouar^1^, Emmanuelle Amsler^1^, Annick Barbaud^1^, Sylvie Chollet-Martin^3^, Pascale Nicaise-Roland^3^, Angèle Soria^4^

###### ^1^Dermatology and Allergy Department, Tenon hospital, APHP, Paris, France; ^2^Allergy Department, Saint Joseph Hospital, Paris, France; ^3^Immunology Department Auto-Immunity and Hypersensitivity, Bichat Hospital, APHP, Paris, France; ^4^Dermatology and Allergy Department, Tenon Hospital, APHP and CIMI-Paris INSERM 1135, Paris, France

**Correspondence:** Annick Barbaud - annick.barbaud@aphp.fr

*Clinical and Translational Allergy* 2018, **8(Suppl 3)**:P170

**Background**

The diagnosis of hypersensitivity reactions (HS) to iodinated contrast media (ICM) is challenging; based on clinical history and skin tests. ICM challenge is the gold standard. The objective of this prospective study was to determine the negative predictive value (NPV) of skin tests and intravenous provocation tests (IPT) at half-dose in patients suspected of HS to ICM; basophil activation tests (BAT) were performed in some patients.

**Methods**

All the 118 patients included had culprit ICM skin tests (patch tests and/or prick tests, intradermal tests) when it was known and 5 other ICM. 110 of them had a single-blind IPT, with culprit ICM if the skin tests were negative and the reaction was not severe or with a substitutive ICM in the opposite case. Eight BAT were performed with 6 different ICM, their results were known only once the allergological work-up (skin tests and IPT with ICM) was achieved.

**Results**

The allergological work-up was positive in 23 patients; 14 with skin tests (1 prick test, 3 intradermal tests, 3 patch tests) and 9 with IPT. Three BAT were negative in patients with negative allergological work-up (skin tests and IPT). In the 5 patients with positive allergogical work up (3 with skin tests and 2 with IPT), only 1 patient (positive skin tests for Ioversol and positive IPT with Iodixanol) had a positive BAT for 3 different ICM; Iomeprol Iodixanol Iobitridol (Ioversol was not tested with BAT).

The NPV for skin tests and IPT was 90.5% in immediate HS and 87.5% in delayed HS.

**Conclusion**

The interest of IPT in HS to ICM is well-documented in addition to skin tests, however the challenge methodology is not codified. Challenge ICM doses ranging from 10 ml to 120 ml according to publications (Table 1). In the largest study published, the NPV of skin tests and IPT (10th of the full dose) was 94% among 340 patients (1). One study found BAT positivity to ICM in 62.5% of confirmed cases of immediate HS (2).

**Table 1:** Conclusion

References	HS type/number of patients	Dose for challenge
*Trcka J* et al. *AJR Am J Roentgenol 2008*	Immediate HS/2	49.05 mL
*Salas M,* et al. *Allergy 2013*	Immediate HS/8 (and 14 challenges with various ICM)	100 mL
*Prieto*-*Garcia* et al. *J Investig Allergol Clin Immunol 2013*	Immediate HS/5	120 mL (n = 3), 24 mL (n = 1), 14 mL (n = 1)
*Sese* et al. *Clin Exp Allergy 2016*	Immediate HS/37	10 mL
*Vernassiere C* et al. *Contact Dermatitis 2004*	Delayed HS/5	10 mL
*Seitz CS,* et al. *Eur J Radiol 2009*	Delayed HS/4	53.5 mL
*Torres MJ,* et al. *Allergy 2012*	Delayed HS/44	100 mL (cumulative dose)
*Lerondeau B,* et al. *J Allergy Clin Immunol 2016*	Delayed and immediate HS/340	10 mL (10th of the full dose)

The NPV for skin tests and IPT in this study was 90.5% in immediate HS and 87.5% in delayed HS. The value of BAT in the exploration of HS to PCI is to be confirmed on a larger series.

(1) Lerondeau B, et al. J Allergy Clin Immunol 2016

(2) Salas M, et al. Allergy 2013

#### P171 Delayed allergic reactions to iodinated contrast media in Vilnius University Hospital Santaros Klinikos

##### Justina Rudyte^1^, Laura Malinauskiene^2^, Algirdas Tamosiunas^1^, Violeta Kvedariene^3^

###### ^1^Vilnius University Faculty of Medicine, Vilnius, Lithuania; ^2^Vilnius University Faculty of Medicine; Clinic of Infectious, Chest diseases, Dermatology and Allergology, Institute of Clinical Medicine, Vilnius, Lithuania; ^3^Vilnius University Faculty of Medicine; Institute of Biomedical Sciences, Department of Pathology, Faculty of Medicine, Vilnius University, Vilnius, Lithuania

**Correspondence:** Justina Rudyte - r.justina08@gmail.com

*Clinical and Translational Allergy* 2018, **8(Suppl 3)**:P171

**Background**

Iodinated contrast media (ICM) is widely used in radiology as a diagnostic method. Adverse reactions to ICM may occur. Allergic reactions, especially delayed type, are rare and mostly manifest as mild and moderate skin reactions. However, delayed reaction and late diagnosis can lead to life-threatening conditions.

**Methods**

The study included 38 patients with suspected allergic reactions to ICM in Vilnius university hospital Santaros klinikos during 2014–2017. Allergy tests were performed according to ENDA rules. Skin tests were performed with iohexol, iopromide, diatrizoate, iodixanol. Reactions to ICM were evaluated after 15 min, 1, 24, 48 h. Reevaluation after ICM readmission was performed.

**Results**

The average age of patients was 54 (SD ± 12) years. 26.3% (n = 10) were male and 73.7%. (n = 28) were female. Culprit drugs revealed from the clinical history were diatrizoate 18.4 proc. (n = 7), iohexol 13.25% (n = 5), iodixanol 13.2% (n = 5), iopromide 7.9% (n = 3), unknown iodine containing drug 44.7% (n = 17). Allergy testing was performed in 94 months (SD ± 156) on average after previous exposure. The rate of positive allergy tests was 13.2% (n = 5) in all tested patients: iohexol 75% (n = 3), diatrizoate 50% (n = 2), iodixanol 25% (n = 1), iopromide 25% (n = 1). One 33 years old man with erythema fix induce by iopromide and proven by skin biopsy, had true delayed allergy to ICM. Provocation skin tests with iopromide were negative, but the reexposure with the same ICM was positive after 21 h.

**Conclusion**

True delayed allergy to iodinated contrast media is rare. Patients with late reactions to iodinate contrast media may need to be observed for up 2 days after the procedure.

#### P172 Immediate-hypersensitivity to proton pump inhibitors – a case series

##### Joana Pita, Rosa-Anita Fernandes, Carlos Loureiro, Ana Todo-Bom, Emilia Faria

###### Coimbra Universitary Hospital Centre, Coimbra, Portugal

**Correspondence:** Joana Pita - joana.s.pita@gmail.com

*Clinical and Translational Allergy* 2018, **8(Suppl 3)**:P172

**Background**

Proton pump inhibitors (PPIs) are widely used in the Portuguese population. Treatment with PPIs is generally well tolerated, but hypersensitivity reactions (HSR) have been described. In these cases, it is essential to investigate the existence of a true allergy, identify the culprit PPI and provide an alternative therapy.

**Methods**

The authors performed a retrospective analysis of patients medical records. Data related to skin prick tests (SPT), intradermal tests (ID) and drug provocation tests (DPT) performed in our Allergy and Clinical Immunology Department from January 2014 to December 2017, were collected. We selected 9 patients with suspected PPI allergy studied in that period of time.

**Results**

The population was constituted mainly by female patients (66.7%). The youngest patient was 40 years old and the oldest 69 years old, with a mean of 51 years ± 8.4 years. Pantoprazole (5) was the PPI most commonly involved in the suspected allergic reaction, followed by omeprazole (3) and esomeprazole (1). Hypersensitivity reactions were: maculopapular rash in 5 cases, followed by anaphylaxis in 3 cases, and palpebral angioedema in 1 case. Only one patient with anaphylaxis was treated with adrenaline.

Five patients were submitted to skin tests (1 patient missed the tests appointment and 3 patients were submitted directly do DPT). All 5 patients had negative SPTs. In what concerns the IDT, 3 patients had positive results. Six patients were submitted to DPT, 2 of those diagnostic and 4 alternative challenges, all negative. One patient was submitted to SPT and IDT with pantoprazole, omeprazole, lansoprazole, esomeprazole and rabeprazole, with positive results to all the PPI tested. Consequently, she was treated with ranitidine, since the previous reaction (anaphylaxis) contraindicated a drug provocation test. As all drug provocation tests were negative, in 6/9 patients it was possible to maintain a therapy with a PPI with good clinical tolerance.

**Conclusion**

SPT and IDP are important for the diagnosis of PPI allergy, as well as for the identification of the suspected PPI and identification of a safe alternative. In our population, IDT seem to have higher sensitivity than SPT in identifying the culprit drug. Based on the IDT results, we performed an alternative and safe DPT, managing to keep the PPI therapy in the majority of patients.

#### P173 Hypersensitivity reaction to lenalidomide: two different clinical patterns in the same patient

##### Blanca Noguerado-Mellado^1^, Patricia Rojas-Perez-Ezquerra^1^, Vicente Albéndiz-Gutierrez^1^, Maria Carmen Lillo-Ordóñez^1^, Monica Ballesteros-Andres^2^

###### ^1^Allergy Department, Madrid, Spain; ^2^Hematology Department, Madrid, Spain

**Correspondence:** Blanca Noguerado-Mellado - blancanoguerado@gmail.com

*Clinical and Translational Allergy* 2018, **8(Suppl 3)**:P173

**Background**

Lenalidomide is an immunomodulatory agent (analogue of thalidomide) that is approved for use in multiple myeloma, myelodysplastic syndrome (MS) and mantle cell lymphoma. This drug is generally well tolerated, but skin toxicity due to high doses or real allergic reactions can occur. Hypersensitivity reactions ranging from urticaria, purpura, morbilliform exanthema or even Stevens–Johnson Syndrome or Toxic Epidermal necrolysis has been described in the literature. We report a patient with MS with a delayed adverse skin reaction to Lenalidomide.

**Case report**

A 78-years-old male with a history of penis epidermoid carcinoma (2011), prostatic cancer (2012) and MS, presented with a generalized exanthema, with intense erythema and pruritus, with no mucosae affection, after 2–3 days of the second cycle of Lenalidomide. Corticoids and antihistamines were administered and the reaction resolved in 15–20 days without desquamation or residual lesions. Lenalidomide was discontinued at that time and an allergy workout was carried out.

Patch tests (PT) were performed with lenalidomide at 10% in DMSO (dimethylsulfoxide) with negative readings at Day 2 (D2) and Day 4 (D4). An oral challenge test was then performed reaching 25 mg with good tolerance.

Afterwards, three more cycles of lenalidomide were given without reactions but in the forth, a generalized erythema appeared again and antihistamines were given, and the rash disappeared in 7 days.

Another allergy study was performed then. PT were now positive with lenalidomide at 10% in DMSO at D2 and D4. Since the use of lenalidomide for treating his MS was indispensible, he underwent slow lenalidomide desensitization. We started with 1 mg daily for 7 days, 2, 5 mg daily for 7 days, 5 mg another 5 days, but at the sixth day the patient developed an erythematous rash on back, arms and thorax. He was treated with oral antihistamines and corticosteroids. We reduced dose of lenalidomide to 2.5 mg, and given daily for another 6 days, but at the seventh day again a generalized exanthema appeared. He was treated with corticosteroid and desensitization was then discontinued.

**Conclusion**

Lenalidomide skin toxicity due to high doses of the drug is frequent, but real allergic reactions may occur. In our patients, both clinical patterns were observed.

There is little described in the literature about positive skin test and successful desensitization to lenalidomide with these kinds of type IV hypersensitivity reactions.

The protocol used in this patient was not appropriated, but maybe rapid desensitization could have been a safe alternative.

**Consent to publish**

Consent to publish was obtained from the patients involved in this study.

#### P175 Mastocytosis and drugs: a retrospective study

##### Jan Walter Schroeder, Michel Chevallard, Laura Michelina Losappio, Lea Caron, Daniela Macellaio, Corrado Mirone, Elide Anna Pastorello

###### Ospedale Niguarda, Milan, Italy

**Correspondence:** Jan Walter Schroeder - janwaltervolk.schroeder@ospedaleniguarda.it

*Clinical and Translational Allergy* 2018, **8(Suppl 3)**:P175

**Background**

Mastocytosis is an afinalistic mast cell proliferation. Skin is the most frequent organ involved. Mast cell infiltration of extra-cutaneous site is defined systemic mastocytosis (SM). Patients with mastocytosis often develop hypersensitivity reactions to various triggers, including drugs.

**Methods**

We retrospectively described our cohort of mastocytosis patients. We compared patient with Hypersensitivity Drug Reaction (HDR) versus patients without HDR.

**Results**

In a population of 34 patients with mastocytosis, 10 (29.4%) experienced HDR to NSAIDs, antibiotics, bisphosphonates, steroids. Skin involvement was significantly more common in HDR patients than in subjects without HDR (89% versus 54%, p = 0.05). Serological tryptase was higher in HDR patients than in subjects without HDR (91.36 ng/ml versus 26.84 ng/ml, p = 0.06). The prevalence of osteoporosis (22% versus 29%, p = 0.62) and splenomegaly (22% versus 29%, p = 0.62) was similar between the two groups. 8/10 (80%) patients with HDR, in addition to typical cutaneous hypersensitivity symptoms such as urticaria and angioedema, suffered non-specific symptoms (flushing, diarrhea, itching without skin lesions, tachycardia). The totality of HDR patients sustained tolerance tests with alternative drugs (acetaminophen, COX2 selective NSAIDs, clarithromycin) without development of adverse reactions.

**Conclusion**

Cutaneous mastocytosis can be exacerbated by several drugs. In mastocytosis patients with HDR, urticaria and angioedema are frequently associated with other non-specific symptoms and signs. For mastocytosis patients with higher tryptase level, it is preferable to prescribe drugs with low hypersensitivity risk (acetaminophen, COX2 selective NSAIDs, clarithromycin).

#### P176 Cetuximab desensitisation in patient with colon rectal cancer

##### Deshinta Putri Mulya

###### Sardjito Hospital - Gadjah Mada University, Yogyakarta, Indonesia

**Correspondence:** Deshinta Putri Mulya - deshintamulya@yahoo.com

*Clinical and Translational Allergy* 2018, **8(Suppl 3)**:P176

**Background**

Cetuximab is one of the targeting therapy for colon cancer. Cetuximab is known to be well tolerated by patients. Skin reactions such as acne form rash are the most common side effects. Cetuximab is associated with severe allergic reactions in 3–5% of patients. We present a case of Cetuximab allergy which is then been desensitised.

**Case report**

A 36-year-old female patient, diagnosed with stage IV sigmoid colon cancer and pulmonary subpleural metastasis, has undergone first-line chemotherapy with the Folfox-Avastin regimen. Post-chemotherapy, evaluation shows progressive disease condition and this patient is planned to be given chemotherapy with Folfiri-Cetuximab regimen for 12 cycles with 2 weeks interval. In the first cetuximab administration the patient undergoes rapid type hypersensitivity reactions with widespread urticarial manifestations. We desensitize the patient according to the protocol used by Saif MW et al. (2009), with appropriate dose adjustment as required. We still find allergic reactions to chemotherapy when the desensitisation is done, from second until fifth cycle but the reaction that arises was more rare and mild. In the administration of the 6th chemotherapy we stop the desensitisation and start to give chemotherapy as original protocol. The chemotherapy can be safely given from sixth cycle until the end of the cycle.

**Conclusion**

Desensitisation was developed to meet the need for safe drug delivery in patients who are known to have an allergic reaction to the drug. Following the published and proven successful drug desensitisation protocol, can be used as a guide for desensitisation in our clinical practice with minor modifications in respect of the dosage used.

**Consent to publish**

Consent to publish was obtained from the patients involved in this study.

#### P179 Anaphylaxis to methylprednisolone sodium succinate in a patient with large-vessel vasculitis

##### Ivan Markovic, Silva Pukšic, Joško Mitrovic, Jadranka Morovic-Vergles

###### Division of Clinical Immunology, Allergy and Rheumatology, Department of Internal Medicine, School of Medicine, University of Zagreb, Dubrava University Hospital, Zagreb, Croatia

**Correspondence:** Ivan Markovic - imarkoviczg@gmail.com

*Clinical and Translational Allergy* 2018, **8(Suppl 3)**:P179

**Background**

Immediate hypersensitivity reactions to systemic glucocorticoids are rare, and their exact incidence is unknown. Glucocorticoids are essential in the initial treatment of large-vessel vasculitis.

**Case report**

A 69-year-old woman was diagnosed with large-vessel vasculitis involving thoracic and abdominal aorta along with the major branches, and admitted to the hospital for treatment. The patient had a history of arterial hypertension, euthyroid goiter, osteoporosis and appendectomy revealing localized adenocarcinoma. She reported no hypersensitivity reactions to drugs or food, and had not received systemic glucocorticoids previously.

Two minutes after the patient had received the first dose of methylprednisolone sodium succinate 80 mg intravenously, she experienced itching and redness of the palms, followed by generalized pruritus, erythema, nausea and vomiting. Her blood pressure was 170/90 mmHg and heart rate 110 beats per minute. The physical examination revealed also a few wheals on abdomen and lower back, and there was no wheezing. The patient was treated with loratadine 10 mg orally, chloropyramine 20 mg and metoclopramide 5 mg intravenously, followed by a complete withdrawal of symptoms and signs of hypersensitivity reaction. Adrenaline was not given. No other medications were administered concomitantly with or in the 5 h prior to methylprednisolone. More than 4 h elapsed since the patient’s last food intake.

Two days later we performed an open graded challenge with oral prednisone. The initial dose was 1.25 mg, and further incremental doses of 2.5 mg, 5 mg, 10 mg and 30 mg were given at hourly intervals. The patient tolerated well the highest administered oral dose of prednisone. The treatment of vasculitis was continued with 60 mg of prednisone daily.

**Conclusion**

Systemic glucocortocoids have been associated with hypersensitivity reactions including anaphylaxis. The patterns of cross-reactivity among different drugs are not defined. Taking into account their widespread use, allergy evaluation is necessary to identify “safe” alternative glucocorticoids.

We report a patient with large-vessel vasculitis who developed anaphylaxis on first exposure to methylprednisolone sodium succinate. Besides the glucocorticoid, other possible allergens were succinate esters and cow’s milk proteins, that can be found in traces in the specific lactose-containing preparation. However, the latter were highly unlikely to cause the reaction considering the patient’s tolerance to ingested milk. The patient tolerated oral prednisone in a graded challenge, that was thereafter given in the treatment of vasculitis.

**Consent to publish**

I confirm that I have received written consent for online publication from all patients described (or their parents/guardians), where there are images, videos, or 3 more indirect identifiers to be published.

## Friday 20 April 2018

### Drug reactions in malignancy treatment

#### O05 COBIOPHAD: progresses towards in vitro diagnosis of drug allergies by a point-of-care device

##### Luis A. Tortajada-Genaro, Estrella Fernández, Teresa Molina, Sergi Morais, Angel Maquieira

###### Universitat Politecnica de Valencia, Valencia, Spain

**Correspondence:** Luis A Tortajada-Genaro - luitorge@qim.upv.es

*Clinical and Translational Allergy* 2018, **8(Suppl 3)**:O05

**Background**

Advances on molecular diagnostic techniques are revolutionizing the diagnosis and treatment of diseases. The use of in vitro diagnostic (IVD) tests for the determination of specific IgEs associated to drug hypersensitivity is a highly demanded solution to substitute the invasive and risky in vivo tests. The current IVD assays show low sensitivity (lower than 40%; detection limits > 0.2 IUA/mL), analyse few BLCs with high rates of false positives and negatives. Furthermore, the employed instruments are benchtop, expensive instruments, only available for well-resource hospitals. The potential of a connected point-of-care (POC) device, providing rapid reliable results should aid the disease diagnosis in any decentralized setting.

COBIOPHAD (Compact biophotonic platform for drug allergy diagnosis) is a three-year European project within the frame of H2020 program to build a highly innovative compact disc-based system for improved diagnosis of allergy to beta-lactam antibiotics (BLCs). The aim is the development of a competitive multiplexed diagnostic device to provide sensitive, selective, rapid and cost-effective IVD results.

**Methods**

The COBIOPHAD test integrates multiple key enabling technologies, including photonics, microfluidics, opto-electronics, bio-analytical tools, and advanced manufacturing technologies. Also, a sophisticated cloud-based data networking and management system will eventually provide allergy decision support to medical staff in clinics and hospitals worldwide. More experimental details are described in the project webpage [http://www.cobiophad.eu].

**Results**

The up-to-date advances of the project will be presented in detail, showing the critical aspects already implemented, together with the challenges faced and the solutions found. The model BLC targets are the derivatives of the subgroups (1) penams such as benzylpenicillin, benzylpenilloate, amoxicillin, and piperacillin; (2) cephems, including cefuroxime, ceftriaxone and cefotaxime; (3) carbapenems as meropenem; (4) monobactam as aztreonam, and (5) oxapenam as clavulanic acid. Nevertheless, the developed technologies could be extended to other allergens following the sensing principle. Furthermore, their scalability will help to cover a broad range of medical scenarios, i.e. doctor office, emergency and critical care Units to Allergy Departments. Serum samples will be tested to either drug or to the complete family within a single run.

**Conclusion**

The COBIOPHAD consortium works to support the antibiotic prescriptions which in turn will contribute to the sustainability of healthcare systems. Therefore, this improved diagnostic tool will contribute to advance the health status and quality of life of millions of citizens worldwide that suffer with allergies.

Acknowledgments: H2020 program (Project COBIOPHAD, Grant Agreement No. 688448), being an initiative of the Photonics Public Private Partnership (www.photonics21.org).

#### O06 Characterization of penicillin-specific naïve CD8+ T cells: from extracellular to intracellular protein haptenation

##### Rami Bechara^1^, Marie-Eliane Azouri^1^, Luc De Chaisemartin^1^, Sylvie Chollet-Martin^1^, Delphine Joseph^2^, Richard Weaver^3^, Bernard Maillère^4^, Marc Pallardy^1^

###### ^1^INSERM UMR 996, Univ Paris-Sud, Université Paris-Saclay, 92290, Châtenay-Malabry, France; ^2^UMR CNRS 8076, Univ Paris-Sud, Université Paris-Saclay, 92290, Châtenay-Malabry, France; ^3^Institut de Recherches Internationales Servier, 92284, Suresnes, France; ^4^SIMOPRO, IBiTecS, CEA, Saclay, France

**Correspondence:** Rami Bechara - rami.bechara@u-psud.fr

*Clinical and Translational Allergy* 2018, **8(Suppl 3)**:O06

**Background**

According to the hapten hypothesis, protein haptenation by covalent conjugation of drugs such as benzylpenicillin (BP) is considered a key process for the allergic response. Our group has recently shown the existence of naïve CD4+ T cells recognizing BP in healthy donors through an extracellular human serum albumin (HSA) haptenation by BP (HSA-BP). Moreover, BP-haptenated peptides from HSA, participating in the immunization of allergic patients, were also identified. However, less is known about the naïve CD8+ T cells repertoire. The purpose of this work was to identify naïve CD8+ T cells specific for BP and to explore mechanism dictating the activation of BP-specific CD8+ T cells.

**Methods**

Co-cultures were established with naïve CD8+ T cells and autologous dendritic cells (DCs) loaded with HSA-BP or free BP. The specific CD8+ T cell response was measured using an IFN-γ ELISpot assay. In order to explore the requirement for MHC class I molecule and proteasome, DCs were pre-treated with an anti-MHC class I antibody or MG132 respectively, then loaded with the antigen.

**Results**

HSA-BP was produced at basic pH and analyzed by mass spectrometry MALDI-TOF analysis showing a covalent binding of an average of 15 molecules of BP per HSA molecule. In contrast to naïve CD4+ T cells, HSA-BP was recognized by naïve CD8+ T cells in only one donor out of five tested healthy donors. Since MHC class I molecules are classically thought to present peptides derived from endogenous protein, we checked whether free BP can prime naïve CD8+ T cells through direct intracellular protein haptenation in DCs.

**Fig. 1:**
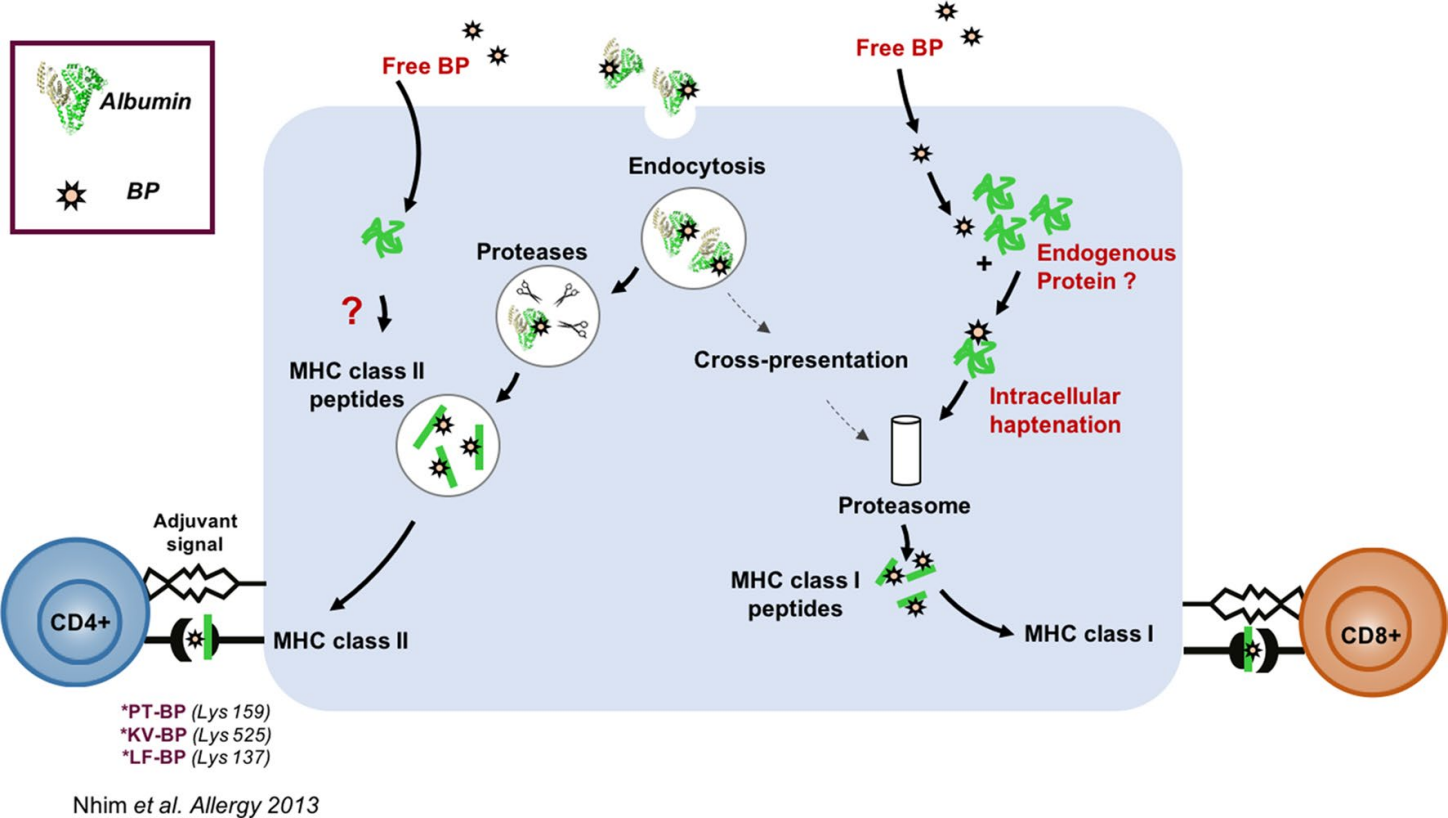
Results

We were able to detect a naïve CD8+ T cells repertoire for BP in the tested healthy donors. Moreover, the BP-specific CD8+ T cell response was MHC class I and proteasome-dependent (Fig. 1).

**Conclusion**

Our work showed the existence of a naïve CD8+ T cell repertoire for BP when DCs were treated with free BP suggesting that patients could be immunized by haptenated peptides from intracellular proteins generated in antigen-presenting cells.

## Saturday 21 April 2018

### Drug hypersensitivity pathomechanisms

#### O07 Characterization of drug-treated hepatocyte-derived exosomes: implications for the mechanistic basis of idiosyncratic drug-induced liver injury

##### Monday O. Ogese^1^, Catherine J. Betts^1^, Lee Faulkner^2^, Rosalind Jenkins^2^, B. Kevin Park^2^, Dean J. Naisbitt^2^

###### ^1^Pathology Sciences, Drug Safety and Metabolism, IMED Biotech Unit, AstraZeneca, Cambridge Science Park, Milton Road, Cambridge, United Kingdom; ^2^MRC Centre for Drug Safety Science, Department of Molecular and Clinical Pharmacology, University of Liverpool, Ashton Street, Liverpool, United Kingdom

**Correspondence:** Monday O. Ogese - m.o.ogese@liverpool.ac.uk

*Clinical and Translational Allergy* 2018, **8(Suppl 3)**:O07

**Background**

Idiosyncratic drug-induced liver injury (iDILI) is a rare and unpredictable adverse reaction with complex pathomechanisms. iDILI can be fatal and represents a major reason for drug attrition and the withdrawal of already licenced drugs because it is difficult to predict preclinically. iDILI has a delayed onset, up to several months after the administration of the drug(s) implicated. Multiple genetic (HLA alleles) and non-genetic factors may predispose individuals to iDILI. It is now evident that iDILI is immune-mediated and may involve the activation of drug antigen-specific T cells. Since the interaction between hepatocytes and immune cells is likely essential in determining the outcome of drug exposure, we sought to investigate the role of hepatocyte-derived exosomes (HDE) in the transfer of drug-derived signals to the immune system. Exosomes are cell-derived vesicles secreted into most biological fluids including blood, urine, saliva and cell culture media. These vesicles ferry RNA, lipids and protein cargo from their cell of origin to other cells, and may play a key role in immune activation. The aims of this study were to: 1. Isolate and characterise drug-induced changes in protein expression of HDE. 2. To evaluate the uptake of HDE into dendritic cells.

**Methods**

Freshly isolated or commercially sourced primary human hepatocytes (PHH) were treated with three drugs associated with T cell-mediated DILI (flucloxacillin, amoxicillin and isoniazid), a reactive metabolite nitroso-sulfamethoxazole (SMX-NO) and a non-DILI causing drug (piperacillin) for 24 h. Supernatant from hepatocyte cultures was collected for exosome isolation using Exoquick tissue culture solution. HDE were first characterised using electron microscopy (EM). Subsequently, immunoblotting was performed to identify typical exosomal protein markers. Immunofluorescence was used to explore the uptake of HDE by human monocyte-derived dendritic cells. Lastly, LC–MS was utilised to investigate drug-induced changes in exosomal protein expression.

**Results**

HDE measured between 50 and 200 nm and expressed two typical exosomal protein markers, CD63 and Hsp70. Uptake of exosomes by monocyte-derived dendritic cells occurred mainly via phagocytosis. LC–MS analysis identified over 1900 hepatocyte-derived exosomal proteins. Drug treatment of hepatocytes results in changes in exosomal protein expression. Furthermore, amoxicillin, flucloxacillin and SMX-NO formed drug-protein adducts with specific exosomal proteins.

**Conclusion**

Collectively, this study has identified drug-modified proteins that are transported to immune cells within HDE. In on-going studies, we are exploring whether the hepatocyte protein adducts might act as antigens and activate drug-specific T cells from patients with iDILI.

## Saturday 21 April 2018

### Risk stratification as a tool in drug allergy investigation

#### O08 Direct graded amoxicillin challenges in adults with low risk beta-lactam allergy labels: the West Australian Experience

##### Brittany Knezevic^1^, Elizabeth Klinken^1^, Grace Thompson^1^, Naoko Horimoto^2^, Charlotta Ekstrom^3^, Michaela Lucas^3^

###### ^1^Department of Immunology, Pathwest, Perth, Australia; ^2^Department of Immunology, Fiona Stanley Hospital, Perth, Australia; ^3^Department of Immunology, Sir Charles Gairdner Hospital, Perth, Australia

**Correspondence:** Michaela Lucas - michaela.lucas@health.wa.gov.au

*Clinical and Translational Allergy* 2018, **8(Suppl 3)**:O08

**Background**

Self reported antibiotic allergy to predominantly beta-lactam derivatives labels approximately 18% of hospitalised patients in Australia, however the majority of these labels can be removed following drug allergy assessment. In February 2016, the Australian Society of Clinical Immunology & Allergy (ASCIA) introduced consensus guidelines for the assessment of patients with penicillin allergy history according to risk of reaction to provocation testing.

**Methods**

All beta-lactam challenges performed at two West Australian tertiary hospitals between February 2016 and September 2017 were audited. Patients were stratified into low risk and high risk groups based on their allergy history. Low risk allergy history was defined as (1) a remote reaction without systemic allergic features, or (2) symptom suggestive of non-allergic intolerance, and (3) absence of significant cardiac or respiratory comorbidities. We reviewed outcomes for low risk patients who underwent direct amoxicillin challenges (1/10th, followed by full dose). Low risk patients who underwent skin testing, not in accordance to ASCIA guidelines, were excluded from this analysis. Patients that did not fulfil low risk criteria were categorised as high risk and underwent skin testing and selected beta-lactam challenges according to usual clinical practice.

**Results**

We analysed 209 adult patients with beta-lactam allergies. Of these, 73 patients had a low risk allergy history: 66 reported a distant rash, and 7 patients reported fevers, hallucinations or probable side-effects. Thirty two low risk patients were excluded because they underwent skin testing. The remaining 41 patients underwent direct amoxicillin challenge according to the ASCIA guidelines. Of these, 37 (90%) patients tolerated the challenge and their beta-lactam allergy labels were removed. Four (10%) patients had mild reactions. There were no episodes of anaphylaxis.

There were 136 high-risk patients, of which 91 had a history of grade 3 or 4 anaphylaxis to beta-lactams. Forty five (33%) high risk patients had positive skin prick or intradermal tests. A total of 115 high risk beta-lactam challenges were performed and 13 (11%) patients reacted. Adrenaline was required for 4 patients: all responded promptly to treatment. There was no statistical difference in the rates of oral provocation challenge reactions between low risk patients who underwent direct amoxicillin challenge, and high risk patients who underwent standard of care testing (p = 1.00, RR = 1.20, 95% CI = 0.42–3.47).

**Conclusion**

This preliminary data suggests that direct amoxicillin challenge following ASCIA guidelines is a safe and efficient approach to de-labelling patients with a low risk history of penicillin allergy.

## Saturday 21 April 2018

### Severe cutaneous adverse reactions to drugs

#### O09 MicroRNAs associated with T cell activation are up-regulated in plasma exosomes in severe cutaneous reactions

##### Alejandra Monroy^1^, Jose Luis Castrejon^1^, Noe Duran^1^, Judith Dominguez^2^, Silvia Mendez^2^, Nancy Pulido^3^, José Angel Baltazar^3^, Dean Naisbitt^4^

###### ^1^Instituto Politecnico Nacional, Mexico City, Mexico; ^2^Instituto Nacional de Ciencias Médicas y Nutrición Salvador Zubirán, Mexico City, Mexico; ^3^Centro Medico Nacional La Raza, Mexico City, Mexico; ^4^University of Liverpool, Liverpool, United Kingdom

**Correspondence:** Alejandra Monroy - alemonroya@hotmail.com

*Clinical and Translational Allergy* 2018, **8(Suppl 3)**:O09

**Background**

Drug hypersensitivity reactions (DHR) can affect almost 6% of the general population and can lead to deathly diseases such as Steven Johnson’s syndrome (SJS) and toxic epidermal necrolysis (TEN). Recently, miRNAs represent a new and exciting field of investigation due to their capacity to regulate gene expression. Previous investigations demonstrated the up-regulation of miR-18a in skin and plasma of patients with severe cutaneous manifestations such as toxic epidermal necrolysis. Nonetheless, miRNAs expression in microvesicles in these pathologies has not been addressed.

**Methods**

Exosomes from plasma of 5 newly diagnostic SJS/TEN patients were purified using Total exosome Isolation Reagent (*Invitrogen*) and surface markers were detected by Western Blot. Size distribution was determinated by Transmission Electron Microscopy. We performed a total RNA extraction, and miRNAs expression was assessed using GeneChip™ miRNA 4.0 array (*Thermo Fisher Scientific*). The individual expression of miRNAs in allergic and healthy donors was quantified using miR-18a and -155 proofs (*Exiqon*) by real time PCR.

**Results**

Proteins of membrane CD9 and CD81 were detected in purified exosomes; moreover, all the macrovesicles had a size distribution from 30 to 120 nm diameter characteristic of exosomes. We performed a microarray analysis of allergic donors, and a change in the expression of several miRNAs was seen across samples. Bioinformatic analysis are currently under development to determine the regulated genes and the biological pathways related with the up-regulation of the miRNAs. Finally, we evaluated the expression of miR-18a and -155, since previous experiments identified them to play a role in the pathogenesis of the diseases and in the T-cell activation process. We detected an up-regulation in the expression of miR-18a in plasma´s exosomes from SJS/TEN patients, although its role in the apoptosis process needs to be assessed. Similar to the expression of miR-18a, we found miR-155 highly expressed in exosomes. This miRNA could have an impact in the activation of distant cells, also, further investigations related to the target gene of miR-155 and its mechanism in the activation of T cell needs to be carried out.

**Conclusion**

MiRNAs are consider important players in pathologies because their capacity to regulate gene expression. Here, we demonstrated for the first time the up-regulation of miRNAs previously involved in apoptosis and T-cell activation in exosomes. These findings could open the pathway to find biomarkers for these deathly diseases.

